# Discovery and SAR analysis of 5-chloro-4-((substituted phenyl)amino)pyrimidine bearing histone deacetylase inhibitors

**DOI:** 10.1080/14756366.2022.2097446

**Published:** 2022-07-14

**Authors:** Lin Zhang, Yiming Chen, Fahui Li, Lihui Zhang, Jinhong Feng, Lei Zhang

**Affiliations:** a Department of Medicinal Chemistry, School of Pharmacy, Weifang Medical University, Weifang, China; b School of Medicine and Pharmacy, Ocean University of China, Qingdao, China; c School of Stomatology, Weifang Medical University, Weifang, China; d Shandong Analysis and Test Center, Qilu University of Technology (Shandong Academy of sciences), Jinan, China

**Keywords:** Histone deacetylase, inhibitor, anticancer, solid tumour, drug design

## Abstract

Histone deacetylases (HDACs) are validated targets for the development of anticancer drugs in epigenetics. In the discovery of novel HDAC inhibitors with anticancer potency, the 5-chloro-4-((substituted phenyl)amino)pyrimidine fragment is assembled as a cap group into the structure of HDAC inhibitors. The SAR revealed that presence of small groups (such as methoxy substitution) is beneficial for the HDAC inhibitory activity. In the enzyme inhibitory selectivity test, compound **L20** exhibited class I selectivity with IC_50_ values of 0.684 µM (selectivity index of >1462), 2.548 µM (selectivity index of >392), and 0.217 µM (selectivity index of >4608) against HDAC1, HDAC2 and HDAC3 compared with potency against HDAC6 (IC_50_ value of >1000 µM), respectively. In the antiproliferative assay, compound **L20** showed both hematological and solid cancer inhibitory activities. In the flow cytometry, **L20** promoted G0/G1 phase cell cycle arrest and apoptosis of K562 cells.

## Introduction

Histone deacetylases (HDACs) are a family of enzymes that lead to the lysine residue deacetylation of histones and various non-histone proteins. The balance of the acetylation state of specific proteins is regulated by HDACs and the acetyl group transferring histone acetyltransferases (HATs)^1^^,^^2^. There are four classes of HDACs identified in human^3^^,^^4^. Class I HDACs (HDAC1, 2, 3 and 8) are mainly located in the nucleus. Class II HDACs are subdivided into IIa (HDAC4, 5, 7 and 9) an IIb (HDAC6 and 10). Class III HDACs, a group of NAD + dependent enzymes termed sirtuins (Sirt1-7), have been found in nucleus, cytoplasm and mitochondria. The class IV HDAC, HDAC11, is the latest member of HDAC family.

The acetylation level of histone and non-histone proteins modulated by HDACs and HATs participates in the regulation of cellular functional balances^5^. Overexpression and aberrant recruitment of HDACs are closely correlated with the occurrence and development of various diseases, especially cancer^6^^,^^7^. Inhibition of HDACs has been extensively studied in the development of anticancer drugs^8^. Vorinostat (SAHA)^9^ is the first US FDA-approved HDAC inhibitor utilised for the treatment of cutaneous T-cell lymphoma (CTCL). Romidepsin (FK-228), Belinostat (PXD101), Tucidinostat (Chidamide, approved by the Chinese FDA) and Panobinostat (LBH589) were successively approved for the treatment of CTCL, peripheral T-cell lymphoma (PTCL), and multiple myeloma^10–12^, respectively.

In general, a zinc-binding group (ZBG), a linker and a cap motif constitute the essential pharmacophores of a HDAC inhibitor^8^. ZBGs are used for the binding of zinc ion located in the active site of class I, II and IV HDACs. Cap region contributing to the hydrophobic interactions leads to the structural diversity of HDAC inhibitors. Fatty or aromatic linkers are utilised to connect the former two pharmacophores.

**Figure 1.**
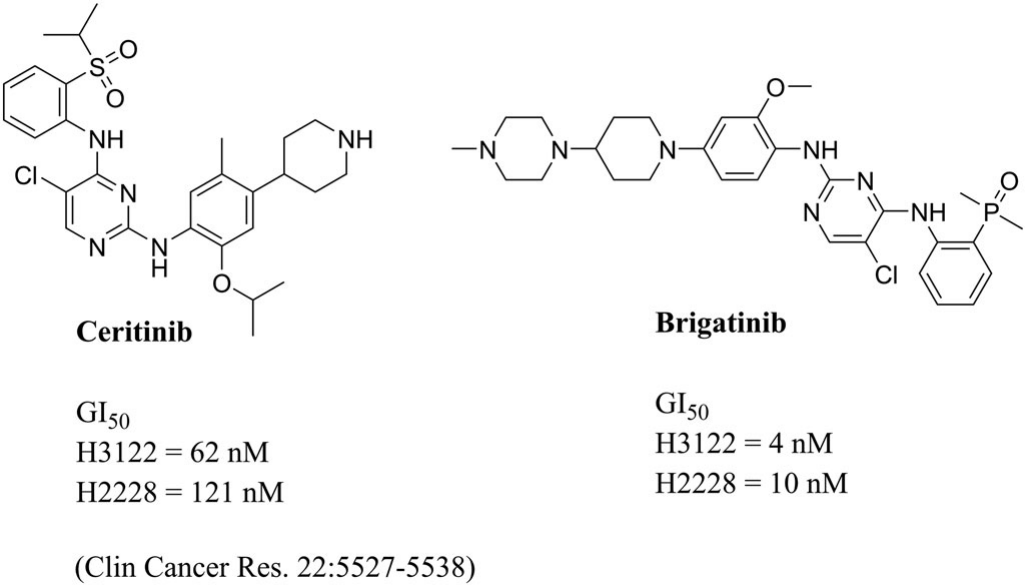
Representative ALK inhibitors and their antiproliferative potency.

**Figure 2.**
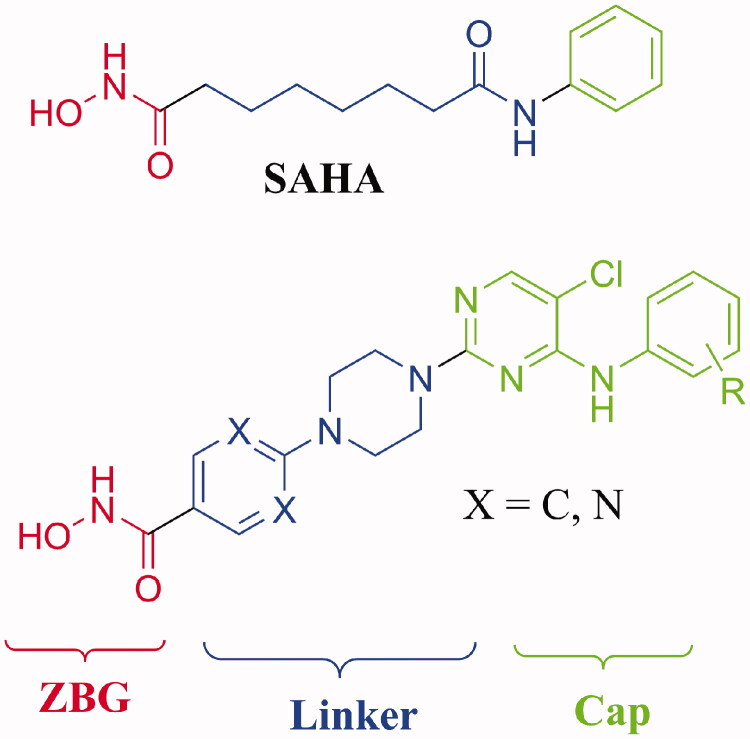
Design of 5-chloro-4-((substituted phenyl)amino)pyrimidine containing HDAC inhibitors.

The 5-chloro-4-((substituted phenyl)amino)pyrimidine structure is widely used in the design of anaplastic lymphoma kinase (ALK) inhibitors which have exhibited significant potency in the inhibition of solid tumours (Figure 1)^13^^,^^14^. To form strong hydrophobic interactions with residues in the opening of active site, the 5-chloro-4-((substituted phenyl)amino)pyrimidine group is integrated into the cap moiety of HDAC inhibitors (Figure 2). The pyrimidine motif is commonly utilised in the design of HDAC inhibitors, and the pyrimidine group plays an important role in improving the solubility of target molecules, enhancing the polar interactions between inhibitors and HDACs, and optimising pharmacokinetic parameters of HDAC inhibitors^15^^,^^16^. Currently, lack of efficacy against solid tumours in clinical trials restricted the application of HDAC inhibitors. The introduction of substituted pyrimidine group is expected to enhance potency of current HDAC inhibitors in the inhibition solid tumour cells. Hydroxamic acid group was utilised as ZBG; phenylpiperazine and pyrimidinylpiperazine were introduced as linkers. The synthesised target compounds were investigated in the enzyme inhibitory assay, *in vitro* antiproliferative screening, cell cycle and apoptosis test.

## Results and discussions

### HDAC enzyme inhibitory activity

**Figure 3.**
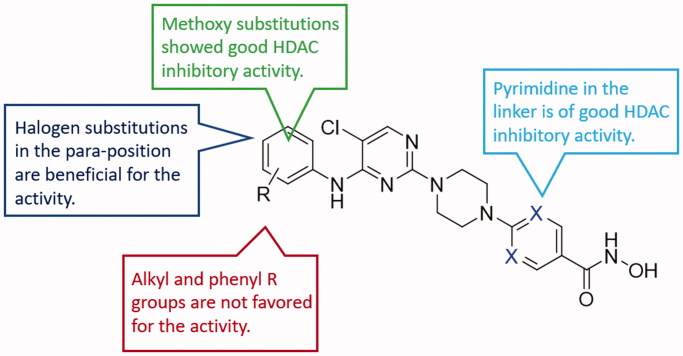
SAR analysis of the derived molecules.

The synthesised molecules were firstly screened against Hela nucleus extract containing a mixture of HDAC isoforms. Percentage inhibitory rate was calculated to determine the activity of tested compounds (Table 1), and Vorinostat (SAHA) was used as a positive control. Compared with the phenyl unsubstituted **L1**, the alkyl R groups did not increase the enzyme inhibitory activity of target molecules, such as **L2** and **L3** (Figure 3). Halogen substitutions in the para-position were revealed to be beneficial for the inhibitory activity, such as **L4**, **L11** and **L17**. Compounds with methoxy substitutions such as **L6**, **L7**, **L9**, **L15**, **L20** and **L21**, showed enhanced activities compared with **L1**. The phenyl substitution led to decreased inhibitory activity, such as **L14** and **L22**. It is indicated that small-sized R groups (such as methoxy group) are favourable for the enzyme inhibitory activity and substitutions in the para-position are of benefit to the potency.

**Table 1.** Structure and potency of the derived compounds in the activity screening. 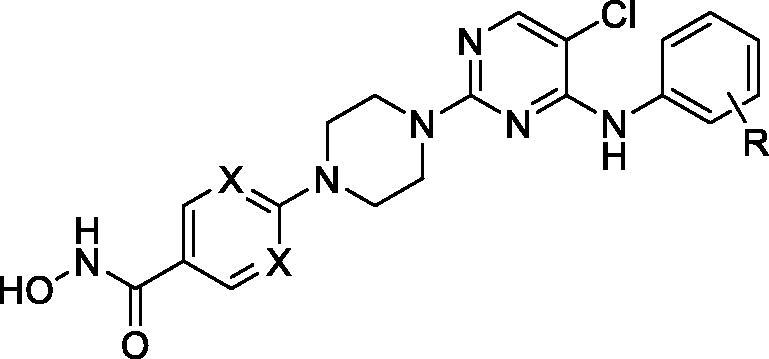

Compounds	R	X	HDACs^a^	K562^a^
L1	–H	C	50.54 ± 2.27	30.69 ± 0.32
L2	–CH_3_(p)	C	47.98 ± 1.54	43.89 ± 0.81
L3	– CH_2_CH_3_ (p)	C	50.54 ± 0.21	19.50 ± 0.06
L4	–F(p)	C	53.89 ± 3.35	58.20 ± 1.05
L5	–CN(p)	C	48.33 ± 0.64	31.51 ± 0.09
L6	–3,5–2OCH_3_	C	59.74 ± 0.03	56.60 ± 0.11
L7	−3,4–2OCH_3_	C	58.37 ± 1.55	63.91 ± 1.12
L8	–OCH_2_CH_3_ (p)	C	49.32 ± 0.89	37.15 ± 1.54
L9	–OCH_3_(m)	C	55.28 ± 0.15	50.78 ± 2.92
L10	–Br(m)	C	51.50 ± 2.03	31.64 ± 1.12
L11	–Br (p)	C	59.82 ± 4.01	60.90 ± 0.21
L12	–Cl(m)	C	32.74 ± 0.38	50.61 ± 1.86
L13	–Cl(p)	C	52.44 ± 1.44	54.14 ± 2.34
L14	–C_6_H_5_ (p)	C	46.80 ± 1.07	35.18 ± 1.21
L15	–3,4,5–3OCH_3_	C	58.15 ± 2.02	58.63 ± 2.01
L16	–I(m)	C	46.50 ± 4.74	37.15 ± 1.54
L17	–I(p)	C	54.51 ± 3.05	56.40 ± 2.16
L18	–3–Cl–4–F	C	52.92 ± 2.00	31.27 ± 0.92
L19	–SO_2_CH(CH_3_)_2_(o)	C	44.91 ± 4.97	21.34 ± 0.65
L20	–3,5–2OCH_3_	N	72.52 ± 1.26	74.18 ± 1.18
L21	–3,4–2OCH_3_	N	58.95 ± 0.74	62.62 ± 2.45
L22	–C_6_H_5_ (p)	N	40.93 ± 0.50	37.08 ± 2.11
SAHA	–	–	50.12 ± 0.74	52.16 ± 0.74

^a^Illustrated as percentage inhibitory rate at concentration of 1.0 µM, and each value is the mean of three experiments. R group substitutions, o: ortho-position; m: meta-position; p: para-position.

Among the derived compounds, **L20** with pyrimidine in the linker exhibited the highest inhibitory activity in the enzymatic screening. Therefore, molecule **L20** was evaluated in the HDAC enzyme inhibitory selectivity test against HDAC1, 2, 3 and 6 (Table 2). Comparing the inhibitory potency against HDAC6, compound **L20** showed an inhibitory pattern of class I selectivity. Among HDAC1, 2 and 3, molecule **L20** exhibited high HDAC3 inhibitory activity with IC_50_ values of 0.217 µM compared with the inhibitory activity against HDAC1 and HDAC2 (IC_50_ values of 0.684 and 2.548 µM, respectively).

**Table 2.** Enzyme inhibitory selectivity of **L20** comparing with SAHA (µM^b^).

	HDAC1	HDAC2	HDAC3	HDAC6
L20	0.684 ± 0.016	2.548 ± 0.079	0.217 ± 0.008	>1000
SAHA	0.0539 ± 0.002	0.152 ± 0.011	0.0397 ± 0.001	ND

^b^Each value is the mean of three experiments.

### Binding pattern analysis

**Figure 4.**
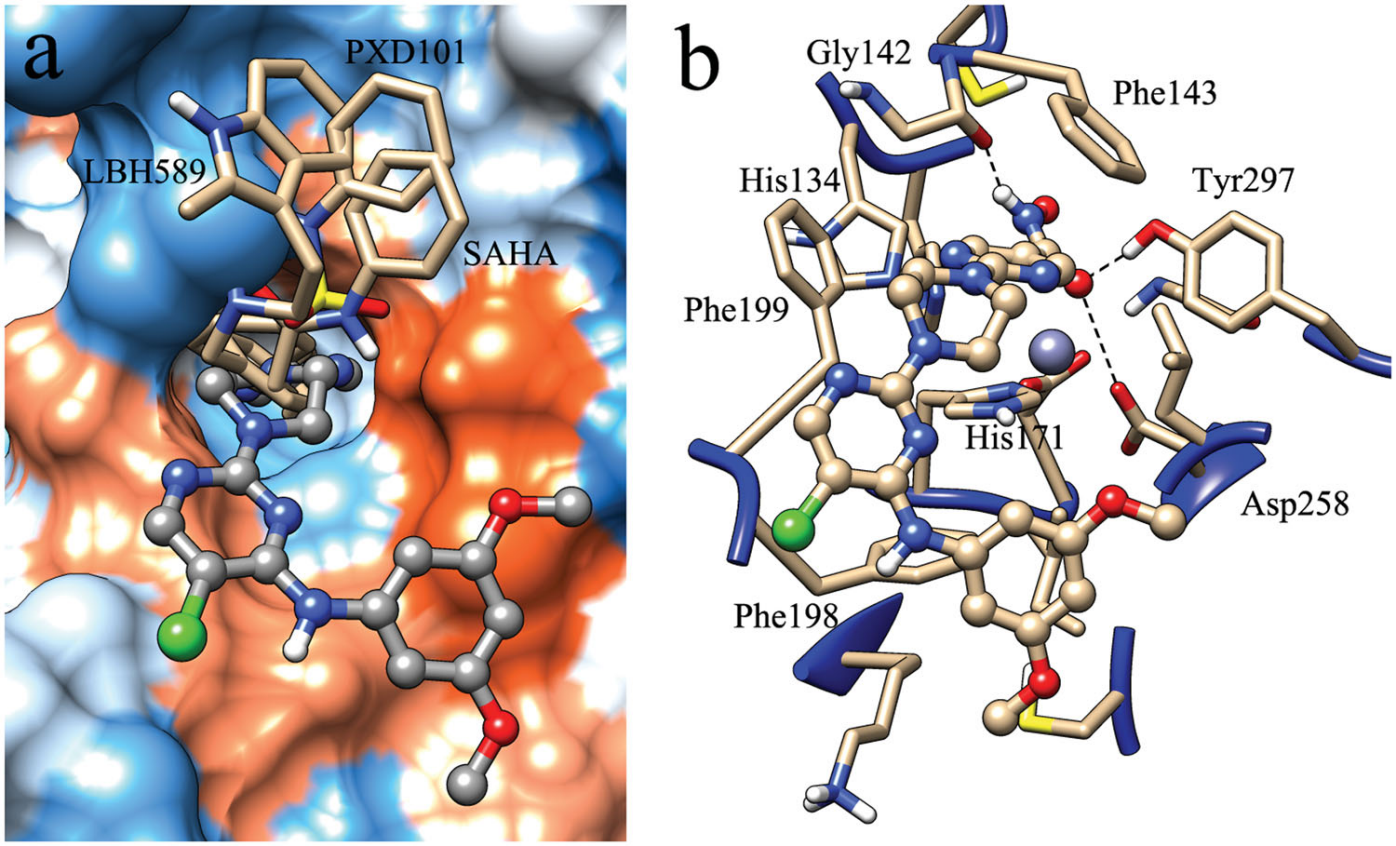
Binding pattern of molecule **L20** in the active site of HDAC3 (PDB entry: 4A69).

Molecular docking was performed to predict the binding pattern of molecule **L20** in the active site of HDAC3 using the FDA-approved SAHA, PXD101 and LBH589 as the control. The results showed that the linker and ZBG of **L20** get into the narrow tunnel in the active site (Figure 4(a)). The cap moiety binds to the opening of the catalytic site. Unlike the caps of control molecules (SAHA, PXD101 and LBH589) which locate to a small pocket in the opening of the active site, the cap of the HDAC3 selective molecule **L20** binds to the hydrophobic region in the surface of the catalytic site. The hydroxamic acid group which chelates to the zinc ion in the end of the binding pocket can also form hydrogen bond interactions with surrounding residues, such as Gly142, Asp258 and Tyr297 (Figure 4(b)). Hydrophobic interactions formed between the linker part of molecule **L20** and key residues (Phe143, His171, Phe199 and Leu265) plays a significant role in the ligand-receptor binding. The cap region of **L20** also makes contributions to the hydrophobic interactions by binding to hydrophobic residues, such as Phe198. The docking result provides structural information for further derivatisation of molecule **L20**.

### *In vitro* antiproliferative activity

All the synthesised compounds were screened against K562 cells (Table 1), and the methoxy substituted molecules exhibited high inhibitory rates compared with SAHA. Compound **L20** with good performances in both enzyme and K562 cell inhibitory tests were selected for further antiproliferative assay against various cell lines. K562, U266, U937, C6, Fadu, MDA-MB-231, MDA-MB-468, A549, A2780 and HepG2 cell lines were used in the cell-based anticancer evaluation of **L20**. As illustrated in Table 3, both **L20** and SAHA exhibited higher potency in the inhibition of hematological cancer cell lines (K562, U266 and U937) compared with the solid tumour cell inhibitory activity. It is remarkable that compound **L20** shows good solid tumour cell inhibitory activities with IC_50_ values of 1.98 µM, 1.42 µM, 1.63 µM, 2.23 µM, 1.06 µM, 0.97 µM and 3.17 µM against C6, Fadu, MDA-MB-231, MDA-MB-468, A549, A2780 and HepG2 cells comparing with SAHA (IC_50_ values of 3.22 µM, 4.12 µM, 3.59 µM, 4.85 µM, 4.22 µM, 2.54 µM and 3.97 µM, respectively). The result revealed the potential of molecule **L20-**based drug discovery in the treatment of both hematological malignancy and solid cancer.

**Table 3.** Antiproliferative activities of **L20** against various cancer cell lines (IC_50_, µM^a^).

	L20	SAHA
K562	0.34 ± 0.02	1.96 ± 0.07
U266	0.28 ± 0.01	0.19 ± 0.01
U937	0.76 ± 0.03	1.21 ± 0.08
C6	1.98 ± 0.10	3.22 ± 0.13
Fadu	1.42 ± 0.05	4.12 ± 0.22
MDA-MB-231	1.63 ± 0.06	3.59 ± 0.24
MDA-MB-468	2.23 ± 0.09	4.85 ± 0.23
A549	1.06 ± 0.03	4.22 ± 0.19
A2780	0.97 ± 0.02	2.54 ± 0.13
HepG2	3.17 ± 0.15	3.97 ± 0.21

^a^Each value is the mean of three experiments.

### Cell cycle analysis

**Figure 5.**
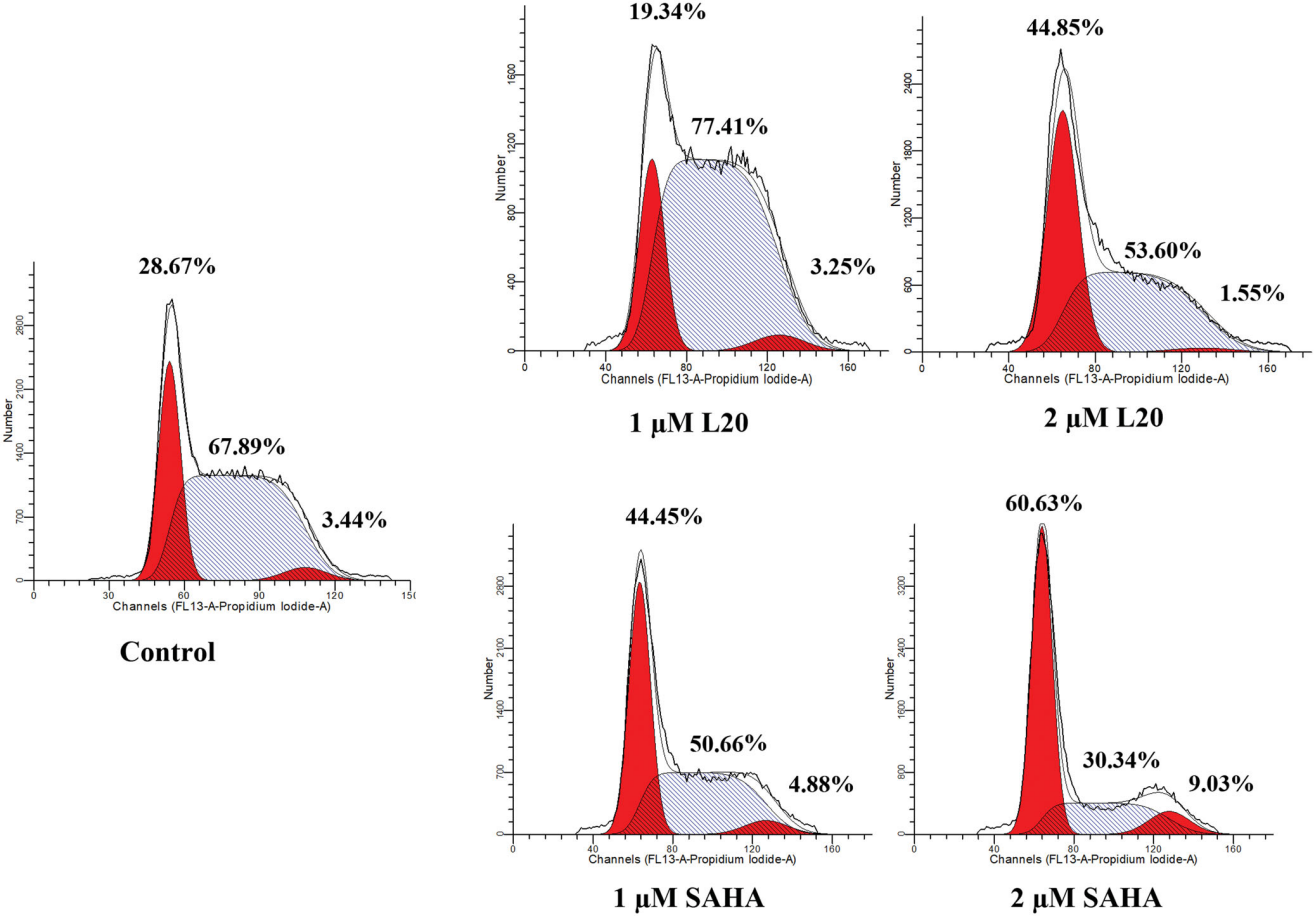
Molecule **L20** induces cell cycle arrest in K562 cells.

Analysis of cell cycle which is divided into G0/G1 phase, S phase, and G2/M phase is often utilised for the anticancer drug evaluation. In this study, the effects of molecule **L20** on cell cycle were investigated using the most sensitive K562 cell line. The cell cycle distribution was analysed by treating K562 cells with 1 and 2 µM of **L20** and SAHA for 24 h. As shown in Figure 5, both **L20** and SAHA increased cell proportion at G0/G1 phase with increasing concentrations, accompanied by decreased cell number at S phase. Compared with SAHA (44.45%, and 60.63% at the concentration of 1and 2 µM), molecule **L20** increased G0/G1 phase ratio of K562 cells from 19.34% at dose of 1 µM to 44.85% at dose of 2 µM. It is suggested that induction of G0/G1 phase arrest contributes to *in vitro* antiproliferative effects of molecule **L20**.

### Cell apoptosis study

**Figure 6.**
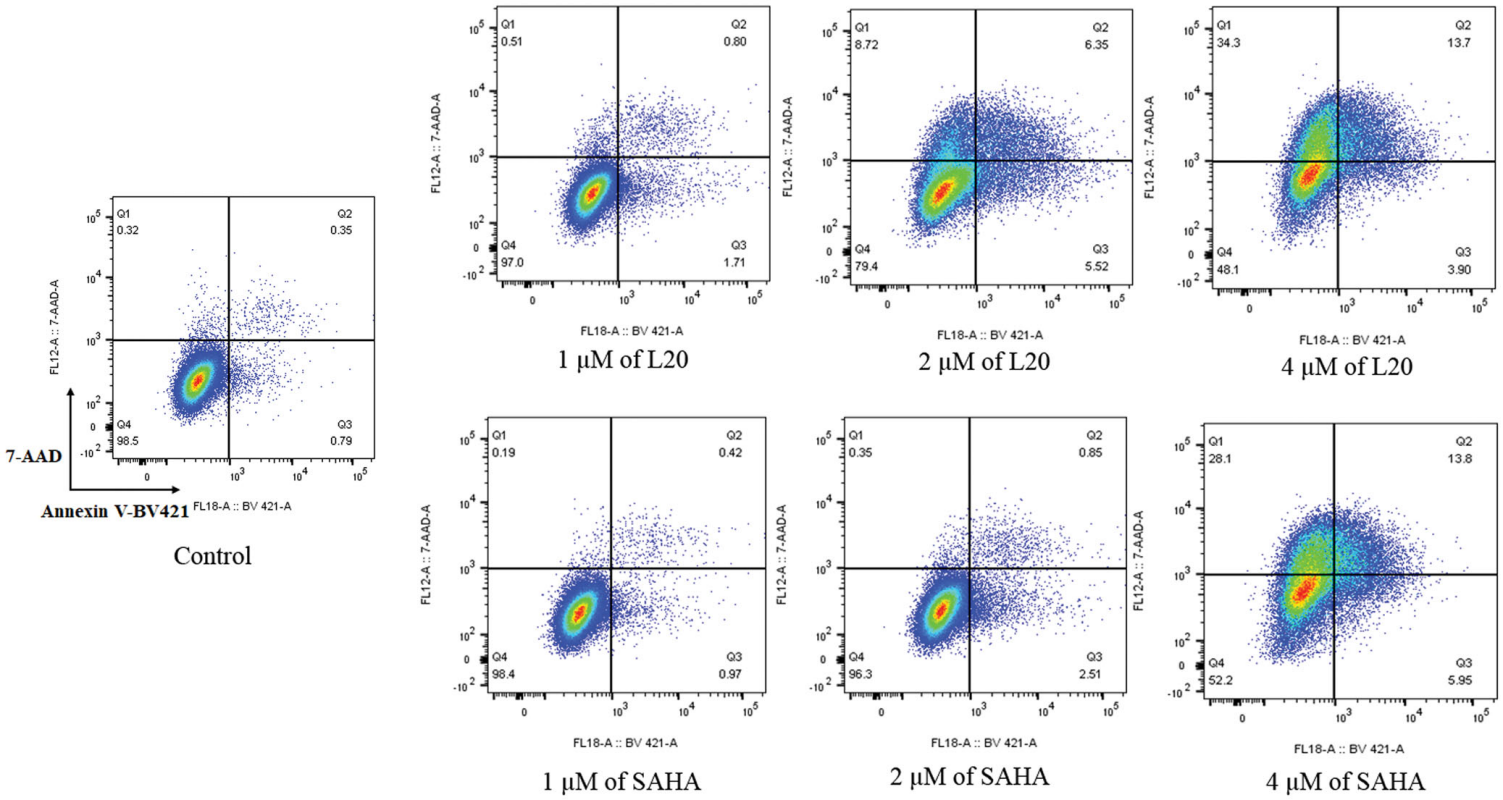
Molecule **L20** induces apoptosis in K562 cells.

Apoptosis, known as programmed cell death, is triggered by a series of effectors, including anticancer drugs. Induction of apoptosis is usually utilised to evaluate the anticancer effects of chemical molecules. In this study, the ability of compound **L20** in promoting K562 cell apoptosis was evaluated by utilising SAHA as the positive control. As illustrated in Figure 6, molecule **L20** increased the apoptotic rate of K562 cells in a dose-dependent manner. It is similar to SAHA (apoptotic rate of 1.39%, 3.36%, 19.75% at dose of 1 µM, 2 µM and 4 µM, respectively) that molecule **L20** induced apoptotic cell proportion from 1.14% of the control to 2.51%, 11.87%, 17.60% at the concentration of 1 µM, 2 µM and 4 µM, respectively. It is indicated that induction of cell apoptosis is involved in the anticancer effect of molecule **L20**.

## Chemistry

**Scheme 1.**
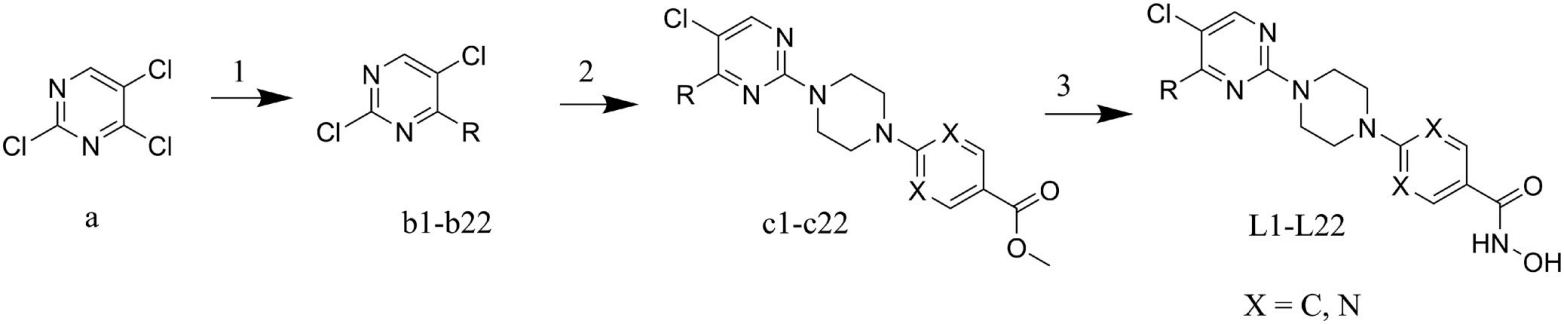
Reagents and conditions: (1) TEA, tetrabutylammonium iodide, DMSO, rt; (2) K_2_CO_3_, ACN, reflux; (3) NH_2_OK, MeOH, rt.

The target molecules were synthesised as illustrated in Scheme 1. Briefly, the commercially available 2,4,5-trichloropyrimidine was used as the starting material. At first, introduction of various benzenamines to the 4-position of pyrimidine ring was performed to afford intermediate **b1**-**b22**. For example, aniline was utilised to react with 2,4,5-trichloropyrimidine in the synthesis of **b1**. Then, intermediate **c1**-**c22** were synthesised by condensation of methyl 4-(piperazin-1-yl)benzoate or methyl 2-(piperazin-1-yl)pyrimidine-5-carboxylate to the 2-position of pyrimidine ring. Methyl 4-(piperazin-1-yl)benzoate was used in the synthesis of **c1**-**c19**; while **c20**-**c22** were synthesised by introduction of methyl 2-(piperazin-1-yl)pyrimidine-5-carboxylate to intermediate **b20**-**b22**. At last, the hydroxamic acid group was introduced by treatment of corresponding intermediates with NH_2_OK in methanol.

## Conclusion

In discovery of novel HDAC inhibitors for the treatment of cancer, the 5-chloro-4-((substituted phenyl)amino)pyrimidine structure often used in the ALK inhibitors was introduced in the design of HDAC inhibitors. A total of 22 compounds were synthesised for structure–activity relationship (SAR) analysis. Among the derived molecules, compound **L20** exhibited high activities in the *in vitro* assays. In the enzyme inhibitory test, compound **L20** showed class I HDACs (especially HDAC3) inhibitory selectivity compared with the potency against HDAC6. Molecule **L20** can also inhibit the proliferation of different kinds of cancer cells in the *in vitro* anticancer test. It is significant that molecule **L20** is effective in inhibition the growth of hematological cancer cells, as well as solid tumour cell lines. The results suggested the potential of molecule **L20** to be used as a lead compound for the treatment of solid cancers. Further K562 cell-based mechanistic study revealed that **L20** is effective in induction of G0/G1 phase arrest and promotion of apoptosis. Overall, a novel HDAC inhibitor with anticancer potency was a discovery for further development of anticancer drugs by inhibition of HDACs.

## Materials and methods

All chemicals were obtained from commercial suppliers and used without further refinement. All reactions were detected by TLC using 0.25 mm silica gel plate (60GF-254). UV light and ferric chloride were used to show TLC spots. ^1^H NMR and ^13 ^C NMR spectra were recorded on a Bruker DRX spectrometer at 500 MHz, using TMS as an internal standard. High-resolution mass spectra were recorded using a Thermo Scientific Q Exactive hybrid quadrupole-orbitrap mass spectrometer from Weifang Medical University.

### Preparation of b1 and its analogues: derivatives b2–b19 were prepared as described for b1 (see below)

2,5-dichloro-N-phenylpyrimidin-4-amine (**b1**). To a solution of compound **a** (0.50 g, 2.73 mmol) in DMSO (5 ml), Tetrabutylammonium iodide (0.10 g, 3.00 mmol) were sequentially added. After 5 min, TEA (0.30 g, 3.00 mmol) and Aniline (0.27 g, 3.00 mmol) were added. The reaction was stirred at room temperature for 3 h. After that, ice water (25 ml) was added and quenched. After the reaction, the solvent was taken up in EtOAc (3 × 30 ml). The EtOAc solution was washed with saturated NaCl (3 × 30 ml), dried over MgSO_4_ and concentrated by evaporation *in vacuo*. The desired compound **b1** (0.34 g, 52% yield) was derived by crystallisation in EtOAc as white powder. HRMS *m/z* [M + H]^+^ calcd for C_10_H_8_Cl_2_N_3_: 240.00953, found: 240.00841. ^1^H NMR (400 MHz, DMSO-d_6_) δ 9.53 (s, 1H), 8.38 (s, 1H), 7.57 (d, J = 8.0 Hz, 2H), 7.40 (t, J = 7.6 Hz, 2H), 7.21 (d, J = 7.3 Hz, 1H).

2,5-dichloro-N-(p-tolyl)pyrimidin-4-amine (**b2**). Crystallised from EtOAc to give a white powder (0.37 g, 60%); HRMS *m/z* [M + H]^+^ calcd for C_11_H_10_Cl_2_N_3_: 254.02518, found: 254.02426. ^1^H NMR (400 MHz, DMSO-d_6_) δ 9.47 (s, 1H), 8.35 (s, 1H), 7.43 (d, J = 7.8 Hz, 2H), 7.20 (d, J = 8.0 Hz, 2H), 2.31 (s, 3H).

2,5-dichloro-N-(4-ethylphenyl)pyrimidin-4-amine (**b3**). Crystallised from EtOAc to give a white powder (0.41 g, 62%); HRMS *m/z* [M + H]^+^ calcd for C_11_H_10_Cl_2_N_3_: 268.04083, found: 268.03946. ^1^H NMR (400 MHz, DMSO-d_6_) δ 9.48 (s, 1H), 8.35 (s, 1H), 7.45 (d, J = 8.0 Hz, 2H), 7.23 (d, J = 7.9 Hz, 2H), 2.61 (dd, J = 15.1, 7.5 Hz, 2H), 1.19 (t, J = 7.6 Hz, 3H).

2,5-dichloro-N-(4-fluorophenyl)pyrimidin-4-amine (**b4**). Crystallised from EtOAc to give a white powder (0.60 g, 85%); HRMS *m/z* [M + H]^+^ calcd for C_10_H_7_Cl_2_FN_3_: 258.00011, found: 257.99924. ^1^H NMR (400 MHz, DMSO-d_6_) δ 9.60 (s, 1H), 8.36 (s, 1H), 7.59 (dd, J = 7.7, 5.3 Hz, 2H), 7.24 (t, J = 8.4 Hz, 2H).

4-((2,5-dichloropyrimidin-4-yl)amino)benzonitrile (**b5**). Crystallised from EtOAc to give a white powder (0.38 g, 54%); HRMS *m/z* [M-H]^-^ calcd for C_11_H_5_Cl_2_N_4_: 262.98913, found: 262.98935. ^1^H NMR (400 MHz, DMSO-d_6_) δ 9.04 (s, 1H), 7.88 (d, J = 7.0 Hz, 1H), 7.38 (d, J = 8.1 Hz, 4H), 6.60 (d, J = 8.2 Hz, 4H).

2,5-dichloro-N-(3,5-dimethoxyphenyl)pyrimidin-4-amine (**b6**). Crystallised from EtOAc to give a white powder (0.71 g, 89%); HRMS *m/z* [M + H]^+^ calcd for C_12_H_12_Cl_2_N_3_O_2_: 300.03066, found: 300.02942. ^1^H NMR (400 MHz, DMSO-d_6_) δ 9.38 (s, 1H), 8.40 (s, 1H), 6.94 (s, 2H), 6.34 (s, 1H), 3.75 (s, 6H).

2,5-dichloro-N-(3,4-dimethoxyphenyl)pyrimidin-4-amine (**b7**). Crystallised from EtOAc to give a white powder (0.75 g, 90%); HRMS *m/z* [M + H]^+^ calcd for C_12_H_12_Cl_2_N_3_O_2_: 300.03066, found: 300.02945. ^1^H NMR (400 MHz, DMSO-d_6_) δ 9.41 (s, 1H), 8.33 (s, 1H), 7.22 (s, 1H), 7.14 (d, J = 8.8 Hz, 1H), 6.97 (d, J = 8.6 Hz, 1H), 3.76 (d, J = 6.7 Hz, 6H).

2,5-dichloro-N-(4-ethoxyphenyl)pyrimidin-4-amine (**b8**). Crystallised from EtOAc to give a white powder (0.62 g, 72%); HRMS *m/z* [M + H]^+^ calcd for C_12_H_12_Cl_2_N_3_O: 284.03574, found: 284.03418. ^1^H NMR (400 MHz, DMSO-d_6_) δ 9.43 (s, 1H), 8.32 (s, 1H), 7.42 (d, *J* = 8.6 Hz, 2H), 6.95 (d, *J* = 8.7 Hz, 2H), 4.03 (q, *J* = 6.9 Hz, 2H), 1.33 (t, *J* = 6.9 Hz, 3H).

2,5-dichloro-N-(3-methoxyphenyl)pyrimidin-4-amine (**b9**). Crystallised from EtOAc to give a white powder (0.35 g, 48%); HRMS *m/z* [M + H]^+^ calcd for C_11_H_10_Cl_2_N_3_O: 270.02009, found: 270.01865. ^1^H NMR (400 MHz, DMSO-d_6_) δ 9.46 (s, 1H), 8.39 (s, 1H), 7.35–7.14 (m, 3H), 6.77 (d, *J* = 8.0 Hz, 1H), 3.76 (s, 3H).

N-(3-bromophenyl)-2,5-dichloropyrimidin-4-amine (**b10**). Crystallised from EtOAc to give a white powder (0.25 g, 72%); HRMS *m/z* [M + H]^+^ calcd for C_10_H_7_BrCl_2_N_3_: 317.92004, found: 317.90173. ^1^H NMR (400 MHz, DMSO-d_6_) δ 9.60 (s, 1H), 8.43 (s, 1H), 7.89 (s, 1H), 7.66 (d, J = 6.3 Hz, 1H), 7.36 (d, J = 6.3 Hz, 2H).

N-(4-bromophenyl)-2,5-dichloropyrimidin-4-amine (**b11**). Crystallised from EtOAc to give a white powder (0.39 g, 50%); HRMS *m/z* [M + H]^+^ calcd for C_10_H_7_BrCl_2_N_3_: 317.92004, found: 317.90173. ^1^H NMR (400 MHz, DMSO-d_6_) δ 9.60 (s, 1H), 8.41 (s, 1H), 7.83–7.31 (m, 4H).

2,5-dichloro-N-(3-chlorophenyl)pyrimidin-4-amine (**b12**). Crystallised from EtOAc to give a white powder (0.38 g, 48%); HRMS *m/z* [M + H]^+^ calcd for C_10_H_5_Cl_3_N_3_: 271.95491, found: 271.95499. ^1^H NMR (400 MHz, DMSO-d_6_) δ 9.62 (s, 1H), 8.44 (s, 1H), 7.76 (s, 1H), 7.61 (d, J = 8.1 Hz, 1H), 7.42 (t, J = 8.1 Hz, 1H), 7.24 (d, J = 8.0 Hz, 1H).

2,5-dichloro-N-(4-chlorophenyl)pyrimidin-4-amine (**b13**). Crystallised from EtOAc to give a white powder (0.45 g, 68%); HRMS *m/z* [M + H]^+^ calcd for C_10_H_7_Cl_3_N_3_: 273.97056, found: 273.98939. ^1^H NMR (400 MHz, DMSO-d_6_) δ 9.61 (s, 1H), 8.41 (s, 1H), 7.63 (d, J = 8.4 Hz, 2H), 7.45 (d, J = 8.4 Hz, 2H).

N-([1,1′-biphenyl]-4-yl)-2,5-dichloropyrimidin-4-amine (**b14**). Crystallised from EtOAc to give a white powder (0.40 g, 64%); HRMS *m/z* [M + H]^+^ calcd for C_16_H_12_Cl_2_N_3_: 316.04083, found: 316.03967. ^1^H NMR (400 MHz, DMSO-d_6_) δ 9.61 (s, 1H), 8.41 (s, 1H), 7.70 (d, *J* = 6.5 Hz, 6H), 7.47 (t, *J* = 7.5 Hz, 2H), 7.36 (t, *J* = 7.3 Hz, 1H).

2,5-dichloro-N-(3,4,5-trimethoxyphenyl)pyrimidin-4-amine (**b15**). Crystallised from EtOAc to give a white powder (0.46 g, 62%); HRMS *m/z* [M + H]^+^ calcd for C_13_H_14_Cl_2_N_3_O_3_: 330.04122, found: 330.03976. ^1^H NMR (400 MHz, DMSO-d_6_) δ 9.41 (s, 1H), 8.38 (s, 1H), 7.06 (s, 2H), 3.77 (s, 6H), 3.67 (s, 3H).

2,5-dichloro-N-(3-iodophenyl)pyrimidin-4-amine (**b16**). Crystallised from EtOAc to give a white powder (0.73 g, 89%); HRMS *m/z* [M + H]^+^ calcd for C_10_H_7_Cl_2_IN_3_: 365.90617, found: 365.90475. ^1^H NMR (400 MHz, DMSO-d_6_) δ 9.55 (s, 1H), 8.41 (s, 1H), 8.03 (s, 1H), 7.68 (d, J = 8.1 Hz, 1H), 7.54 (d, J = 7.8 Hz, 1H), 7.20 (t, J = 8.0 Hz, 1H).

2,5-dichloro-N-(4-iodophenyl)pyrimidin-4-amine (**b17**). Crystallised from EtOAc to give a white powder (0.57 g, 74%); HRMS *m/z* [M + H]^+^ calcd for C_10_H_7_Cl_2_IN_3_: 365.90617, found: 365.90463. ^1^H NMR (400 MHz, DMSO-d_6_) δ 9.57 (s, 1H), 8.40 (s, 1H), 7.73 (d, J = 8.3 Hz, 2H), 7.43 (d, J = 8.3 Hz, 2H).

2,5-dichloro-N-(3-chloro-4-fluorophenyl)pyrimidin-4-amine (**b18**). Crystallised from EtOAc to give a white powder (0.49 g, 65%); HRMS *m/z* [M + H]^+^ calcd for C_10_H_6_Cl_3_FN_3_: 291.96113, found: 291.95831. ^1^H NMR (400 MHz, DMSO-d_6_) δ 9.64 (s, 1H), 8.42 (s, 1H), 7.86 (d, J = 6.7 Hz, 1H), 7.65–7.57 (m, 1H), 7.46 (t, J = 9.1 Hz, 1H).

2,5-dichloro-N-(2-(isopropylsulfonyl)phenyl)pyrimidin-4-amine (**b19**). Crystallised from EtOAc to give a white powder (0.51 g, 71%); HRMS *m/z* [M + H]^+^ calcd for C_13_H_14_Cl_2_N_3_O_2_S: 346.01838, found: 346.01138. ^1^H NMR (400 MHz, DMSO-d_6_) δ 9.81 (s, 1H), 8.56 (s, 1H), 8.33 (d, *J* = 8.3 Hz, 1H), 7.87 (dd, *J* = 18.3, 8.1 Hz, 2H), 7.48 (t, *J* = 7.6 Hz, 1H), 3.53 (dt, *J* = 13.4, 6.7 Hz, 1H), 1.16 (d, *J* = 6.7 Hz, 6H).

### Preparation of c1 and its analogues: derivatives c2–c22 were prepared as described for c1 (see below)

methyl-4–(4-(5-chloro-4-(phenylamino)pyrimidin-2-yl)piperazin-1-yl)benzoate (**c1**). Compound **b1** (0.24 g, 1.00 mmol) was dissolved in ACN. After addition of K_2_CO_3_ (0.28 g, 2.00 mmol), Methyl 4-(piperazin-1-yl)benzoate (0.27 g, 1.22 mmol), the solvent was stirred at 110 °C for 6 h. After that, the reagents were evaporated under vacuum and dissolved in EtOAc. The solvent was washed with saturated brine (3 × 30 ml), dried over MgSO_4_, and evaporated under vacuo. The desired compound **c1** (0.15 g, 35% yield) was derived by crystallisation in EtOAc as white powder. HRMS *m/z* [M + H]^+^ calcd for C_22_H_23_ClN_5_O_2_: 424.15403, found: 424.15341. ^1^H NMR (400 MHz, DMSO-d_6_) δ 8.75 (s, 1H), 8.09 (s, 1H), 7.73 (dd, J = 49.4, 7.9 Hz, 4H), 7.35 (s, 2H), 7.16–6.88 (m, 3H), 3.77 (s, 7H), 3.40 (s, 4H).

methyl-4–(4-(5-chloro-4-(p-tolylamino)pyrimidin-2-yl)piperazin-1-yl)benzoate (**c2**). Crystallised from EtOAc to give a white powder (0.15 g, 36%); HRMS *m/z* [M + H]^+^ calcd for C_23_H_25_ClN_5_O_2_: 438.16968, found: 438.16855. ^1^H NMR (400 MHz, DMSO-d_6_) δ 8.68 (s, 1H), 8.06 (s, 1H), 7.79 (d, J = 8.5 Hz, 2H), 7.53 (d, J = 8.0 Hz, 2H), 7.15 (d, J = 8.1 Hz, 2H), 7.01 (d, J = 8.7 Hz, 2H), 3.77 (s, 4H), 3.76 (s, 3H), 3.39 (s, 4H).

methyl-4–(4-(5-chloro-4-((4-ethylphenyl)amino)pyrimidin-2-yl)piperazin-1-yl)benzoate (**c3**). Crystallised from EtOAc to give a white powder (0.23 g, 47%); HRMS *m/z* [M + H]^+^ calcd for C_24_H_26_ClN_5_O_2_: 452.18533, found:.452.18143. ^1^H NMR (400 MHz, DMSO-d_6_) δ 7.83 (d, *J* = 8.3 Hz, 3H), 7.79 (s, 1H), 7.58 (d, *J* = 7.8 Hz, 1H), 7.19 (d, *J* = 8.0 Hz, 1H), 7.05 (d, *J* = 8.8 Hz, 3H), 7.01 (s, 1H), 3.79 (s, 4H), 3.17 (d, *J* = 4.3 Hz, 4H), 2.60 (dd, *J* = 14.9, 7.5 Hz, 2H), 1.20 (t, *J* = 7.5 Hz, 3H).

methyl-4–(4-(5-chloro-4-((4-fluorophenyl)amino)pyrimidin-2-yl)piperazin-1-yl)benzoate (**c4**). Crystallised from EtOAc to give a white powder (0.24 g, 50%); HRMS *m/z* [M + H]^+^ calcd for C_22_H_22_ClFN_5_O_2_: 442.15561, found: 442.14331. ^1^H NMR (400 MHz, DMSO-d_6_) δ 8.84 (s, 1H), 8.08 (s, 1H), 7.79 (d, J = 8.4 Hz, 2H), 7.65 (dd, J = 8.2, 5.3 Hz, 2H), 7.19 (t, J = 8.7 Hz, 2H), 7.00 (d, J = 8.6 Hz, 2H), 3.77 (s, 3H), 3.75 (s, 4H), 3.39 (s, 4H).

methyl-4–(4-(5-chloro-4-((4-cyanophenyl)amino)pyrimidin-2-yl)piperazin-1-yl)benzoate (**c5**). Crystallised from EtOAc to give a white powder (0.21 g, 42%); HRMS *m/z* [M + H]^+^ calcd for C_23_H_22_ClN_6_O_2_: 449.14928, found: 449.14114. ^1^H NMR (400 MHz, DMSO-d_6_) δ 8.99 (s, 1H), 8.12 (s, 1H), 7.94 (s, 1H), 7.80 (d, *J* = 8.1 Hz, 3H), 7.65 (s, 1H), 7.41 (d, *J* = 8.6 Hz, 1H), 7.01 (d, *J* = 7.9 Hz, 2H), 3.78 (s, 8H), 3.16 (s, 3H).

Methyl-4–(4-(5-chloro-4-((3,5-dimethoxyphenyl)amino)pyrimidin-2-yl)piperazin-1-yl)benzoate (**c6**). Crystallised from EtOAc to give a white powder (0.35 g, 55%); HRMS *m/z* [M + H]^+^ calcd for C_24_H_27_ClN_5_O_4_: 484.17516, found: 484.17340. ^1^H NMR (400 MHz, DMSO-d_6_) δ 8.62 (s, 1H), 8.05 (s, 1H), 7.80 (d, J = 8.6 Hz, 2H), 7.38 (s, 1H), 7.17 (d, J = 8.5 Hz, 1H), 7.01 (d, J = 8.8 Hz, 2H), 6.93 (d, J = 8.7 Hz, 1H), 3.78 (s, 3H), 3.76 (s, 4H), 3.76 (s, 3H), 3.40 (s, 4H).

methyl-4–(4-(5-chloro-4-((3,4-dimethoxyphenyl)amino)pyrimidin-2-yl)piperazin-1-yl)benzoate (**c7**). Crystallised from EtOAc to give a white powder (0.24 g, 46%); HRMS *m/z* [M + H]^+^ calcd for C_24_H_27_ClN_5_O_4_: 484.17516, found: 484.17294. ^1^H NMR (400 MHz, DMSO-d_6_) δ 8.62 (s, 1H), 8.05 (s, 1H), 7.80 (d, J = 8.6 Hz, 2H), 7.38 (s, 1H), 7.16 (t, J = 7.2 Hz, 1H), 7.03–6.98 (m, 2H), 6.93 (d, J = 8.7 Hz, 1H), 3.77 (d, J = 4.6 Hz, 8H), 3.76 (s, 4H), 3.40 (s, 3H).

methyl-4–(4-(5-chloro-4-((4-ethoxyphenyl)amino)pyrimidin-2-yl)piperazin-1-yl)benzoate (**c8**). Crystallised from EtOAc to give a white powder (0.29 g, 52%); HRMS *m/z* [M + H]^+^ calcd for C_24_H_27_ClN_5_O_3_: 468.18024, found: 468.17810. ^1^H NMR (400 MHz, DMSO-d_6_) δ 8.65 (s, 1H), 8.03 (s, 1H), 7.79 (d, *J* = 8.5 Hz, 2H), 7.51 (d, *J* = 8.6 Hz, 2H), 7.00 (d, *J* = 8.6 Hz, 2H), 6.91 (d, *J* = 8.6 Hz, 2H), 4.02 (q, *J* = 6.8 Hz, 2H), 3.76 (d, *J* = 14.3 Hz, 7H), 3.38 (s, 4H), 1.33 (t, *J* = 6.9 Hz, 3H).

methyl-4–(4-(5-chloro-4-((3-methoxyphenyl)amino)pyrimidin-2-yl)piperazin-1-yl)benzoate (**c9**). Crystallised from EtOAc to give a white powder (0.32 g, 56%); HRMS *m/z* [M + H]^+^ calcd for C_23_H_25_ClN_5_O_3_: 454.16459, found: 454.16248. ^1^H NMR (400 MHz, DMSO-d_6_) δ 8.65 (s, 1H), 8.03 (s, 1H), 7.79 (d, *J* = 8.5 Hz, 2H), 7.51 (d, *J* = 8.6 Hz, 2H), 7.00 (d, *J* = 8.6 Hz, 2H), 6.91 (d, *J* = 8.6 Hz, 2H), 4.02 (q, *J* = 6.8 Hz, 2H), 3.76 (d, *J* = 14.3 Hz, 7H), 3.38 (s, 4H), 1.33 (t, *J* = 6.9 Hz, 3H).

methyl-4–(4–(4-((3-bromophenyl)amino)-5-chloropyrimidin-2-yl)piperazin-1-yl)benzoate (**c10**). Crystallised from EtOAc to give a white powder (0.15 g, 37%); HRMS *m/z* [M + H]^+^ calcd for C_22_H_22_BrClN_5_O_2_: 504.06249, found: 504.06058. ^1^H NMR (400 MHz, DMSO-d_6_) δ 8.94 (s, 1H), 8.13 (s, 1H), 8.05 (s, 1H), 7.80 (d, *J* = 8.5 Hz, 2H), 7.70 (d, *J* = 7.8 Hz, 1H), 7.33–7.23 (m, 2H), 7.02 (d, *J* = 8.6 Hz, 2H), 3.80 (s, 3H), 3.78 (s, 4H), 3.42 (s, 4H).

methyl-4-(4-(4-((4-bromophenyl)amino)-5-chloropyrimidin-2-yl)piperazin-1-yl)benzoate (**c11**). Crystallised from EtOAc to give a white powder (0.10 g, 28%); HRMS *m/z* [M + H]^+^ calcd for C_22_H_22_BrClN_5_O_2_: 502.06454, found: 502.04648. ^1^H NMR (400 MHz, DMSO-d_6_) δ 8.90 (s, 1H), 8.11 (s, 1H), 7.80 (d, *J* = 8.6 Hz, 2H), 7.65 (d, *J* = 8.6 Hz, 2H), 7.54 (t, *J* = 12.9 Hz, 2H), 7.00 (d, *J* = 8.6 Hz, 2H), 3.78 (s, 6H), 3.61–3.49 (m, 1H), 3.41 (s, 3H), 3.20 (d, *J* = 4.8 Hz, 1H).

methyl4-(4–(5-chloro-4-((3-chlorophenyl)amino)pyrimidin-2-yl)piperazin-1-yl)benzoate (**c12**). Crystallised from EtOAc to give a white powder (0.12 g, 30%); HRMS *m/z* [M + H]^+^ calcd for C_22_H_22_Cl_2_N_5_O_2_: 458.11506, found: 458.11322. ^1^H NMR (400 MHz, DMSO-d_6_) δ 8.93 (s, 1H), 8.13 (s, 1H), 7.88 (s, 1H), 7.80 (d, J = 8.4 Hz, 2H), 7.67 (d, J = 8.2 Hz, 1H), 7.37 (s, 1H), 7.13 (d, J = 7.9 Hz, 1H), 7.01 (d, J = 8.6 Hz, 2H), 3.78 (d, J = 6.4 Hz, 7H), 3.42 (s, 4H).

methyl4-(4–(5-chloro-4-((4-chlorophenyl)amino)pyrimidin-2-yl)piperazin-1-yl)benzoate (**c13**). Crystallised from EtOAc to give a white powder (0.26 g, 42%); HRMS *m/z* [M-H]^-^ calcd for C_22_H_20_Cl_2_N_5_O_2_: 456.09941, found: 456.10065. ^1^H NMR (400 MHz, DMSO-d_6_) δ 8.91 (s, 1H), 8.11 (s, 1H), 7.80 (d, J = 8.4 Hz, 2H), 7.70 (d, J = 8.5 Hz, 2H), 7.40 (d, J = 8.5 Hz, 2H), 7.01 (d, J = 8.6 Hz, 2H), 3.78 (s, 7H), 3.41 (s, 4H).

methyl4–(4-(4-([1,1′-biphenyl]-4-ylamino)-5-chloropyrimidin-2-yl)piperazin-1-yl)benzoate (**c14**). Crystallised from EtOAc to give a white powder (0.16 g, 34%); HRMS *m/z* [M + H]^+^ calcd for C_28_H_27_ClN_5_O_2_: 500.18533, found: 500.15167. ^1^H NMR (400 MHz, DMSO-d_6_) δ 8.91 (s, 1H), 8.82 (s, 2H), 8.13 (s, 1H), 7.81 (d, J = 8.2 Hz, 2H), 7.71 (s, 3H), 7.70 (s, 2H), 7.56 (dd, J = 21.6, 7.8 Hz, 1H), 7.46 (t, J = 7.5 Hz, 2H), 7.35 (d, J = 7.1 Hz, 1H), 3.96 (s, 4H), 3.81 (s, 4H), 3.79 (s, 3H).

methyl4-(4–(5-chloro-4-((3,4,5-trimethoxyphenyl)amino)pyrimidin-2-yl)piperazin-1-yl)benzoate (**c15**). Crystallised from EtOAc to give a white powder (0.19 g, 41%); HRMS *m/z* [M + H]^+^ calcd for C_25_H_29_ClN_5_O_5_: 514.18572, found: 514.17664. ^1^H NMR (400 MHz, DMSO-d_6_) δ 8.60 (s, 1H), 8.09 (s, 1H), 7.80 (d, J = 8.6 Hz, 2H), 7.14 (d, J = 11.9 Hz, 2H), 6.99 (d, J = 9.3 Hz, 2H), 3.83 (s, 4H), 3.78 (s, 9H), 3.65 (s, 3H), 3.42 (s, 4H).

methyl4-(4–(5-chloro-4-((3-iodophenyl)amino)pyrimidin-2-yl)piperazin-1-yl)benzoate (**c16**). Crystallised from EtOAc to give a white powder (0.22 g, 47%); HRMS *m/z* [M + H]^+^ calcd for C_22_H_22_ClIN_5_O_2_: 550.05067, found: 550.04895. ^1^H NMR (400 MHz, DMSO-d_6_) δ 8.89 (s, 1H), 8.26 (s, 1H), 8.12 (s, 1H), 7.80 (d, J = 8.5 Hz, 2H), 7.68 (d, J = 8.1 Hz, 1H), 7.43 (d, J = 7.8 Hz, 1H), 7.14 (t, J = 8.0 Hz, 1H), 7.01 (d, J = 8.6 Hz, 2H), 3.80 (d, J = 5.1 Hz, 4H), 3.78 (s, 3H), 3.42 (s, 4H).

methyl4-(4–(5-chloro-4-((4-iodophenyl)amino)pyrimidin-2-yl)piperazin-1-yl)benzoate (**c17**). Crystallised from EtOAc to give a white powder (0.32 g, 72%); HRMS *m/z* [M + H]^+^ calcd for C_22_H_22_ClIN_5_O_2_: 550.05067, found: 550.04865. ^1^H NMR (400 MHz, DMSO-d_6_) δ 8.86 (s, 1H), 8.11 (s, 1H), 7.80 (d, J = 8.7 Hz, 2H), 7.68 (d, J = 8.5 Hz, 2H), 7.53 (d, J = 8.5 Hz, 2H), 7.01 (d, J = 8.8 Hz, 2H), 3.78 (s, 7H), 3.41 (s, 4H).

methyl4-(4–(5-chloro-4-((3-chloro-4-fluorophenyl)amino)pyrimidin-2-yl)piperazin-1-yl)benzoate (**c18**). Crystallised from EtOAc to give a white powder (0.21 g, 45%); HRMS *m/z* [M + H]^+^ calcd for C_22_H_21_Cl_2_FN_5_O_2_: 476.10563, found: 476.10107. ^1^H NMR (400 MHz, DMSO-d_6_) δ 7.87–7.75 (m, 4H), 7.66 (d, J = 8.2 Hz, 1H), 6.97 (d, J = 8.4 Hz, 4H), 3.80 (s, 4H), 3.44 (s, 4H), 3.40 (s, 3H).

methyl4-(4–(5-chloro-4-((2-(isopropylsulfonyl)phenyl)amino)pyrimidin-2-yl)piperazin-1-yl)benzoate (**c19**). Crystallised from EtOAc to give a white powder (0.38 g, 62%); HRMS *m/z* [M + H]^+^ calcd for C_25_H_29_ClN_5_O_4_S: 530.16288, found: 530.15332. ^1^H NMR (400 MHz, DMSO-d_6_) δ 9.49 (s, 1H), 8.56 (d, J = 8.3 Hz, 1H), 8.25 (s, 1H), 7.83 (dd, J = 16.8, 8.2 Hz, 4H), 7.39 (d, J = 7.6 Hz, 1H), 7.02 (d, J = 8.4 Hz, 2H), 3.82 (s, 4H), 3.78 (s, 3H), 3.45 (d, J = 4.2 Hz, 4H), 1.17 (d, J = 6.7 Hz, 6H).

methyl-2–(4-(5-chloro-4-((3,5-dimethoxyphenyl)amino)pyrimidin-2-yl)piperazin-1-yl)pyrimidine-5-carboxylate (**c20**). Crystallised from EtOAc to give a white powder (0.25 g, 52%); HRMS *m/z* [M + H]^+^ calcd for C_22_H_25_ClN_7_O_4_: 486.16566, found: 486.15619. ^1^H NMR (400 MHz, DMSO-d_6_) δ 8.77 (s, 1H), 8.66 (d, J = 33.5 Hz, 1H), 7.99 (d, J = 93.2 Hz, 1H), 7.06 (d, J = 12.6 Hz, 3H), 6.27 (d, J = 28.8 Hz, 1H), 3.89 (s, 3H), 3.79 (d, J = 3.9 Hz, 3H), 3.74 (d, J = 5.6 Hz, 8H), 2.60 (s, 3H).

methyl-2–(4-(5-chloro-4-((3,4-dimethoxyphenyl)amino)pyrimidin-2-yl)piperazin-1-yl)pyrimidine-5-carboxylate (**c21**). Crystallised from EtOAc to give a white powder (0.21 g, 48%); HRMS *m/z* [M + H]^+^ calcd for C_22_H_25_ClN_7_O_4_: 486.16566, found: 486.15631. ^1^H NMR (400 MHz, DMSO-d_6_) δ 8.82 (s, 2H), 8.62 (s, 1H), 8.05 (s, 1H), 7.37 (s, 1H), 7.20 (d, J = 10.0 Hz, 1H), 6.94 (d, J = 9.0 Hz, 1H), 3.93 (s, 3H), 3.81 (s, 3H), 3.75 (s, 11H).

methyl-2–(4–(4-([1,1′-biphenyl]-4-ylamino)-5-chloropyrimidin-2-yl)piperazin-1-yl)pyrimidine-5-carboxylate (**c22**). Crystallised from EtOAc to give a white powder (0.19 g, 38%); HRMS *m/z* [M + H]^+^ calcd for C_26_H_25_ClN_7_O_2_: 502.17583, found: 502.16711. ^1^H NMR (400 MHz, DMSO-d_6_) δ 8.87 (s, 1H), 8.11 (s, 1H), 7.80 (d, J = 8.4 Hz, 4H), 7.69 (t, J = 7.5 Hz, 4H), 7.47 (t, J = 7.5 Hz, 2H), 7.35 (d, J = 7.3 Hz, 1H), 7.01 (d, J = 8.7 Hz, 2H), 3.77 (s, 4H), 3.42 (s, 4H).

### Preparation of L1 and its analogues: derivatives L2–L22 were prepared as described for L1 (see below)

4–(4-(5-chloro-4-(phenylamino)pyrimidin-2-yl)piperazin-1-yl)-N-hydroxybenzamide (**L1**). Compound **c1** (0.20 g, 1.0 mmol) was dissolved in 14 ml of NH2OK methanol solution. After 2 h, the solvent was evaporated under vacuum. The residue was acidified with saturated citric acid, and then extracted with EtOAc (3 × 30 ml). The organic layers were combined, washed with brine (3 × 30 ml) and dried over MgSO_4_. The desired compound **L1** (0.11 g, 55% yield) was derived by crystallisation in EtOAc as white powder. HRMS *m/z* [M + H]^+^ calcd for C_21_H_22_ClN_6_O_2_: 425.14928, found: 425.14774.^1^H NMR (400 MHz, DMSO-d_6_) δ 12.39 (s, 1H), 10.95 (s, 1H), 8.76 (s, 1H), 8.09 (s, 1H), 7.66 (t, J = 7.8 Hz, 4H), 7.35 (t, J = 7.7 Hz, 2H), 7.10 (t, J = 7.3 Hz, 1H), 6.98 (d, J = 8.6 Hz, 2H), 3.77 (s, 4H), 3.37 (s, 4H). ^13 ^C NMR (101 MHz, DMSO) δ 175.01, 171.76, 159.57, 155.89, 155.01, 153.08, 139.21, 128.89–128.85 (m), 128.68 (d, J = 20.8 Hz), 123.90, 122.59 (d, J = 16.1 Hz), 114.4, 103.07, 72.92, 47.40, 43.84, 43.16.

4–(4-(5-chloro-4-(p-tolylamino)pyrimidin-2-yl)piperazin-1-yl)-N-hydroxybenzamide (**L2**). Crystallised from EtOAc to give a white powder (0.12 g, 56%); HRMS *m/z* [M + H]^+^ calcd for C_22_H_24_ClN_6_O_2_: 439.16493, found: 439.16321. ^1^H NMR (400 MHz, DMSO-d_6_) δ 11.05 (s, 1H), 8.71 (s, 1H), 8.05 (s, 1H), 7.66 (d, *J* = 8.6 Hz, 2H), 7.53 (d, *J* = 7.7 Hz, 3H), 7.16 (d, *J* = 8.1 Hz, 2H), 6.99 (d, *J* = 8.4 Hz, 2H), 3.31 (s, 8H), 2.30 (s, 3H). ^13 ^C NMR (101 MHz, DMSO) δ 166.59, 155.58 (d, *J* = 11.9 Hz), 154.19, 140.77, 131.97, 131.22, 130.80 (d, *J* = 9.6 Hz), 121.50, 118.73, 113.92, 103.18, 94.38, 51.95, 46.74, 43.79.

4–(4-(5-chloro-4-((4-ethylphenyl)amino)pyrimidin-2-yl)piperazin-1-yl)-N-hydroxybenzamide (**L3**). Crystallised from EtOAc to give a white powder (0.11 g, 54%); HRMS *m/z* [M + H]^+^ calcd for C_23_H_26_ClN_6_O_2_: 453.18058, found: 453.17609. ^1^H NMR (400 MHz, DMSO-d_6_) δ 8.69 (s, 1H), 8.06 (s, 1H), 7.82 (t, J = 10.1 Hz, 4H), 7.58 (d, J = 7.8 Hz, 1H), 7.19 (d, J = 8.0 Hz, 1H), 7.03 (t, J = 9.5 Hz, 4H), 3.78 (d, J = 4.2 Hz, 8H), 2.60 (d, J = 7.5 Hz, 2H), 1.20 (t, J = 7.5 Hz, 3H). ^13 ^C NMR (101 MHz, DMSO) δ 166.60, 159.52, 155.64, 155.37, 154.15, 139.20, 137.37, 131.21, 124.81, 118.61, 113.83, 103.18, 87.48, 51.94, 46.72, 43.77.

4–(4-(5-chloro-4-((4-fluorophenyl)amino)pyrimidin-2-yl)piperazin-1-yl)-N-hydroxybenzamide (**L4**). Crystallised from EtOAc to give a white powder (0.10 g, 50%); HRMS *m/z* [M + H]^+^ calcd for C_21_H_21_ClFN_6_O_2_: 443.13985, found: 443.13855. ^1^H NMR (400 MHz, DMSO-d_6_) δ 10.95 (s, 1H), 8.82 (d, J = 13.1 Hz, 2H), 8.08 (s, 1H), 7.66 (d, J = 8.5 Hz, 4H), 7.19 (t, J = 8.7 Hz, 2H), 6.98 (d, J = 8.6 Hz, 2H), 3.75 (s, 4H), 3.36 (s, 4H). ^13 ^C NMR (101 MHz, DMSO) δ 164.86, 159.57, 157.61, 155.97, 155.05, 153.07, 135.46, 128.58, 124.87 (d, *J* = 8.0 Hz), 122.51, 115.44, 115.22, 114.41, 102.91, 47.39, 43.82, 14.56.

4–(4-(5-chloro-4-((4-cyanophenyl)amino)pyrimidin-2-yl)piperazin-1-yl)-N-hydroxybenzamide (**L5**). Crystallised from EtOAc to give a white powder (0.12 g, 56%); HRMS *m/z* [M + H]^+^ calcd for C_22_H_24_ClN_6_O_2_: 450.14453, found: 450.14090. ^1^H NMR (400 MHz, DMSO-d_6_) δ 12.45 (s, 1H), 8.98 (s, 1H), 8.12 (s, 1H), 7.96 (d, *J* = 5.2 Hz, 1H), 7.80 (d, *J* = 7.0 Hz, 1H), 7.66 (d, *J* = 6.2 Hz, 2H), 7.40 (s, 1H), 7.00 (s, 2H), 3.78 (s, 6H), 3.41 (s, 2H). ^13 ^C NMR (101 MHz, DMSO) δ 175.00, 171.76, 166.59, 159.36, 156.18–155.75 (m), 155.54 (d, *J* = 39.7 Hz), 154.14, 136.50, 131.20, 128.58, 124.32, 123.03, 119.17, 118.68, 116.92, 116.70, 114.43, 113.86, 103.05, 72.92, 60.23, 51.93, 47.35, 46.66, 43.81 (d, *J* = 11.4 Hz), 43.15, 14.55.

4–(4-(5-chloro-4-((3,5-dimethoxyphenyl)amino)pyrimidin-2-yl)piperazin-1-yl)-N-hydroxybenzamide (**L6**). Crystallised from EtOAc to give a white powder (0.15 g, 75%); HRMS *m/z* [M + H]^+^ calcd for C_23_H_26_ClN_6_O_4_: 485.17041, found: 485.16885. ^1^H NMR (400 MHz, DMSO-d_6_) δ 11.19 (s, 1H), 10.95 (s, 1H), 8.78 (d, J = 9.5 Hz, 1H), 8.62 (s, 1H), 8.10 (s, 1H), 7.88 (s, 1H), 7.65 (d, J = 8.5 Hz, 1H), 7.08 (s, 1H), 7.04 (s, 1H), 7.00 (d, J = 8.7 Hz, 1H), 6.26 (d, J = 30.4 Hz, 1H), 3.82 (s, 3H), 3.78 (s, 1H), 3.75 (s, 4H), 3.73 (s, 3H), 3.40 (s, 1H), 3.32–3.29 (m, 1H). ^13 ^C NMR (101 MHz, DMSO) δ 160.59 (d, J = 4.7 Hz), 159.55, 158.70, 155.74, 155.19, 153.09, 141.11, 140.19, 128.56, 122.59, 114.49, 103.21, 101.76, 99.97, 96.59 (d, J = 7.0 Hz), 55.66 (d, J = 3.2 Hz), 47.38, 43.87.

4–(4-(5-chloro-4-((3,4-dimethoxyphenyl)amino)pyrimidin-2-yl)piperazin-1-yl)-N-hydroxybenzamide (**L7**). Crystallised from EtOAc to give a white powder (0.12 g, 55%); HRMS *m/z* [M + H]^+^ calcd for C_23_H_26_ClN_6_O_4_: 485.17041, found: 485.16885. ^1^H NMR (400 MHz, DMSO-d_6_) δ 10.95 (s, 1H), 8.79 (s, 1H), 8.61 (s, 1H), 8.05 (s, 1H), 7.65 (d, J = 8.3 Hz, 2H), 7.38 (s, 1H), 7.16 (d, J = 8.6 Hz, 1H), 6.95 (dd, J = 22.7, 8.5 Hz, 3H), 3.78 (s, 3H), 3.74 (t, J = 12.3 Hz, 8H), 3.31 (s, 3H). ^13 ^C NMR (101 MHz, DMSO) δ 159.63, 155.85, 154.71, 153.10, 148.65, 145.56, 132.52, 128.56, 122.55, 114.46, 112.02, 107.82, 102.90, 56.16, 55.88, 47.41, 43.81.

4–(4-(5-chloro-4-((4-ethoxyphenyl)amino)pyrimidin-2-yl)piperazin-1-yl)-N-hydroxybenzamide (**L8**). Crystallised from EtOAc to give a white powder (0.11 g, 53%); HRMS *m/z* [M + H]^+^ calcd for C_23_H_26_ClN_6_O_3_: 469.17549, found: 469.17401. ^1^H NMR (400 MHz, DMSO-d_6_) δ 11.01 (s, 1H), 8.98 (s, 1H), 8.06 (s, 1H), 7.72 (dd, J = 45.1, 8.6 Hz, 2H), 7.50 (d, J = 8.7 Hz, 2H), 6.98 (d, J = 8.5 Hz, 2H), 6.92 (d, J = 8.7 Hz, 2H), 3.76 (s, 4H), 3.47 (s, 4H), 3.38 (s, 1H), 2.72 (dd, J = 41.7, 15.4 Hz, 1H), 1.34 (t, J = 6.9 Hz, 3H). ^13 ^C NMR (101 MHz, DMSO) δ 166.59, 155.58 (d, J = 11.9 Hz), 154.19, 140.77, 131.97, 131.22, 130.80 (d, J = 9.6 Hz), 121.50, 118.73, 113.92, 103.18, 94.38, 51.95, 46.74, 43.79.

4–(4-(5-chloro-4-((3-methoxyphenyl)amino)pyrimidin-2-yl)piperazin-1-yl)-N-hydroxybenzamide (**L9**). Crystallised from EtOAc to give a white powder (0.13 g, 56%); HRMS *m/z* [M + H]^+^ calcd for C_22_H_24_ClN_6_O_3_: 455.15984, found: 455.15817. ^1^H NMR (400 MHz, DMSO-d_6_) δ 10.95 (s, 1H), 8.80 (s, 1H), 8.69 (s, 1H), 8.10 (s, 1H), 7.65 (d, *J* = 8.3 Hz, 2H), 7.39 (s, 1H), 7.26 (dt, *J* = 15.9, 7.9 Hz, 2H), 6.99 (d, *J* = 8.4 Hz, 2H), 6.66 (d, *J* = 7.8 Hz, 1H), 3.80 (s, 4H), 3.77 (s, 3H), 3.36 (s, 1H), 3.32 (s, 3H). ^13 ^C NMR (101 MHz, DMSO) δ 159.66 (d, J = 14.7 Hz), 155.80, 155.14, 153.09, 140.51, 129.49, 128.57, 122.56, 114.49 (d, J = 5.1 Hz), 109.94, 107.56, 103.15, 55.52, 47.40, 43.86.

4–(4–(4-((3-bromophenyl)amino)-5-chloropyrimidin-2-yl)piperazin-1-yl)-N-hydroxybenzamide (**L10**). Crystallised from EtOAc to give a white powder (0.16 g, 80%); HRMS *m/z* [M + H]^+^ calcd for C_21_H_21_BrClN_6_O_2_: 505.05774, found: 505.05588. ^1^H NMR (400 MHz, DMSO-d_6_) δ 10.96 (s, 1H), 8.93 (s, 1H), 8.81 (s, 1H), 8.09 (d, J = 30.9 Hz, 2H), 7.81–7.59 (m, 3H), 7.34–7.23 (m, 2H), 6.99 (d, J = 8.5 Hz, 2H), 3.80 (s, 4H), 3.34 (d, J = 4.6 Hz, 4H). ^13 ^C NMR (101 MHz, DMSO) δ 159.45, 155.61 (d, J = 11.8 Hz), 153.07, 141.00, 130.67, 128.59, 126.13, 124.86, 122.59, 121.47, 121.06, 114.47, 103.17, 47.38, 43.90.

4–(4–(4-((4-bromophenyl)amino)-5-chloropyrimidin-2-yl)piperazin-1-yl)-N-hydroxybenzamide (**L11**). Crystallised from EtOAc to give a white powder (0.14 g, 70%); HRMS *m/z* [M-H]^-^ calcd for C_21_H_21_BrClN_6_O_2_: 503.05979, found: 503.04236. ^1^H NMR (400 MHz, DMSO-d_6_) δ 10.96 (s, 1H), 8.93 (s, 2H), 8.09 (d, J = 30.9 Hz, 2H), 7.68 (dd, J = 14.8, 8.2 Hz, 3H), 7.34–7.24 (m, 2H), 6.99 (d, J = 8.5 Hz, 2H), 3.80 (s, 4H), 3.34 (d, J = 4.6 Hz, 4H). ^13 ^C NMR (101 MHz, DMSO) δ 159.45, 155.61 (d, *J* = 11.8 Hz), 153.07, 141.00, 130.67, 128.59, 126.13, 124.86, 122.59, 121.47, 121.06, 114.47, 103.17.

4–(4-(5-chloro-4-((3-chlorophenyl)amino)pyrimidin-2-yl)piperazin-1-yl)-N-hydroxybenzamide (**L12**). Crystallised from EtOAc to give a white powder (0.12 g, 60%); HRMS *m/z* [M-H]^-^ calcd for C_21_H_20_Cl_2_N_6_O_2_: 457.11030, found: 457.09189. ^1^H NMR (400 MHz, DMSO-d_6_) δ 10.98 (s, 1H), 8.88 (d, J = 21.6 Hz, 2H), 8.10 (s, 1H), 7.68 (dd, J = 16.5, 8.2 Hz, 4H), 7.40 (d, J = 8.0 Hz, 2H), 6.98 (d, J = 8.2 Hz, 2H), 3.76 (s, 4H), 3.32 (s, 4H). ^13 ^C NMR (101 MHz, DMSO) δ 175.21, 171.79, 159.53, 155.74, 155.29, 153.06, 138.19, 131.38, 128.65, 127.53, 124.27, 122.39, 114.39, 103.07, 72.85, 47.36, 43.84, 43.28.

4–(4-(5-chloro-4-((4-chlorophenyl)amino)pyrimidin-2-yl)piperazin-1-yl)-N-hydroxybenzamide (**L13**). Crystallised from EtOAc to give a white powder (0.11 g, 55%); HRMS *m/z* [M + H]^+^ calcd for C_21_H_20_Cl_2_N_6_O_2_: 459.11030, found: 459.10593. ^1^H NMR (400 MHz, chloroform-d) δ 10.95 (s, 1H), 8.90 (s, 1H), 8.80 (s, 1H), 8.10 (s, 1H), 7.68 (dd, J = 17.8, 8.5 Hz, 4H), 7.40 (d, J = 8.5 Hz, 2H), 6.98 (d, J = 8.5 Hz, 2H), 3.76 (s, 4H), 3.38 (d, J = 4.0 Hz, 4H).^13^C NMR (101 MHz, chloroform-d) δ 176.52, 164.30, 160.51, 160.09, 157.81, 142.99, 133.38, 132.25, 129.04, 127.25, 119.14, 107.82, 77.58, 52.14, 48.35 (d, J = 52.2 Hz).

4–(4–(4-([1,1′-biphenyl]-4-ylamino)-5-chloropyrimidin-2-yl)piperazin-1-yl)-N-hydroxybenzamide (**L14**). Crystallised from EtOAc to give a white powder (0.10 g, 50%); HRMS *m/z* [M + H]^+^ calcd for C_27_H_26_ClN_6_O_2_: 501.18058, found: 501.17673. ^1^H NMR (400 MHz, DMSO-d_6_) δ 10.95 (s, 1H), 8.87 (s, 1H), 8.79 (s, 1H), 8.11 (s, 1H), 7.80 (d, J = 8.0 Hz, 2H), 7.69 (t, J = 7.6 Hz, 5H), 7.64 (s, 1H), 7.46 (t, J = 7.5 Hz, 2H), 7.35 (d, J = 7.3 Hz, 1H), 6.98 (d, J = 8.6 Hz, 2H), 3.79 (d, J = 11.2 Hz, 6H), 3.42 (s, 2H). ^13 ^C NMR (101 MHz, DMSO) δ 159.64, 155.80, 155.21, 153.07, 140.17, 138.80, 135.33, 129.39, 128.59, 127.53, 126.82 (d, J = 18.6 Hz), 122.83, 122.51, 114.39, 103.15, 47.42, 43.90.

4–(4-(5-chloro-4-((3,4,5-trimethoxyphenyl)amino)pyrimidin-2-yl)piperazin-1-yl)-N-hydroxybenzamide (**L15**). Crystallised from EtOAc to give a white powder (0.13 g, 65%); HRMS *m/z* [M + H]^+^ calcd for C_24_H_28_ClN_6_O_5_: 515.18097, found: 515.13367. ^1^H NMR (400 MHz, DMSO-d_6_) δ 12.43 (d, J = 10.5 Hz, 1H), 8.60 (s, 1H), 8.09 (s, 1H), 7.80 (d, J = 8.6 Hz, 2H), 7.16 (s, 2H), 7.01 (d, J = 8.8 Hz, 2H), 3.83 (s, 3H), 3.78 (s, 8H), 3.65 (s, 3H), 3.42 (s, 3H), 2.71 (dd, J = 41.6, 15.4 Hz, 1H). ^13 ^C NMR (101 MHz, DMSO) δ 175.00, 171.75, 166.60, 159.49, 155.66, 154.87, 154.19, 152.87, 135.28, 133.84, 131.18, 118.70, 113.92, 103.10, 99.79, 72.92, 60.60, 56.24, 51.94, 46.68, 43.75, 43.15.

4-(4-(5-chloro-4-((3-iodophenyl)amino)pyrimidin-2-yl)piperazin-1-yl)-N-hydroxybenzamide (**L16**). Crystallised from EtOAc to give a white powder (0.12 g, 60%); HRMS *m/z* [M + H]^+^ calcd for C_21_H_21_ClIN_6_O_2_: 551.04592, found: 551.04193. ^1^H NMR (400 MHz, DMSO-d_6_) δ 10.96 (s, 1H), 8.89 (s, 1H), 8.77 (d, J = 23.6 Hz, 1H), 8.27 (s, 1H), 8.10 (d, J = 13.1 Hz, 1H), 7.66 (d, J = 8.1 Hz, 2H), 7.43 (d, J = 7.8 Hz, 1H), 7.15 (t, J = 8.0 Hz, 1H), 6.99 (d, J = 8.4 Hz, 2H), 3.80 (s, 4H), 3.33 (s, 4H). ^13 ^C NMR (101 MHz, DMSO) δ 159.43, 155.58 (d, J = 11.5 Hz), 153.09, 140.77, 131.97, 130.81 (d, J = 8.7 Hz), 128.69 (d, J = 18.8 Hz), 122.61, 121.50, 114.48, 103.15, 94.37, 47.43, 43.90.

4-(4-(5-chloro-4-((4-iodophenyl)amino)pyrimidin-2-yl)piperazin-1-yl)-N-hydroxybenzamide (**L17**). Crystallised from EtOAc to give a white powder (0.11 g, 55%); HRMS *m/z* [M + H]^+^ calcd for C_21_H_21_ClIN_6_O_2_: 551.04592, found: 551.04199. ^1^H NMR (400 MHz, DMSO-d_6_) δ 10.96 (s, 1H), 8.87 (s, 1H), 8.81 (s, 1H), 8.10 (s, 1H), 7.67 (t, J = 8.9 Hz, 4H), 7.53 (d, J = 8.4 Hz, 2H), 6.98 (d, J = 8.6 Hz, 2H), 3.77 (s, 4H), 3.32 (s, 4H). ^13 ^C NMR (101 MHz, DMSO) δ 159.53, 155.64, 155.35, 153.05, 139.19, 137.38, 128.59, 124.81, 122.47, 114.38, 103.15, 87.47, 47.38, 43.87.

4-(4-(5-chloro-4-((3-chloro-4-fluorophenyl)amino)pyrimidin-2-yl)piperazin-1-yl)-N-hydroxybenzamide (**L18**). Crystallised from EtOAc to give a white powder (0.09 g, 45%); HRMS *m/z* [M + H]^+^ calcd for C_21_H_20_Cl_2_FN_6_O_2_: 477.10088, found: 477.09766. ^1^H NMR (400 MHz, DMSO-d_6_) δ 10.94 (s, 1H), 8.97 (s, 1H), 8.12 (s, 1H), 7.95 (d, J = 5.3 Hz, 1H), 7.71 (d, J = 3.3 Hz, 1H), 7.65 (d, J = 8.6 Hz, 3H), 7.39 (d, J = 9.1 Hz, 1H), 6.98 (d, J = 8.6 Hz, 2H), 4.22 (t, J = 6.6 Hz, 1H), 3.77 (s, 4H), 3.38 (s, 3H). ^13 ^C NMR (101 MHz, DMSO) δ 170.81, 129.14, 114.44, 60.23, 21.24, 14.56.

4-(4-(5-chloro-4-((2-(isopropylsulfonyl)phenyl)amino)pyrimidin-2-yl)piperazin-1-yl)-N-hydroxybenzamide (**L19**). Crystallised from EtOAc to give a white powder (0.12 g, 60%); HRMS *m/z* [M + H]^+^ calcd for C_24_H_28_ClN_6_O_4_S: 531.15813, found: 531.10126. ^1^H NMR (400 MHz, DMSO-d_6_) δ 9.50 (s, 1H), 8.81 (s, 1H), 8.55 (d, J = 8.1 Hz, 1H), 8.25 (s, 1H), 7.83 (dd, J = 18.4, 8.3 Hz, 4H), 7.66 (d, J = 8.2 Hz, 1H), 7.38 (t, J = 7.7 Hz, 1H), 7.00 (t, J = 9.1 Hz, 2H), 3.88 (s, 4H), 3.45 (d, J = 4.7 Hz, 4H), 1.17 (d, J = 6.7 Hz, 6H). ^13 ^C NMR (101 MHz, DMSO) δ 175.01, 171.76, 148.65, 135.34, 130.89, 117.70, 116.54, 116.03, 72.92, 53.50, 43.15, 15.34.

2–(4-(5-chloro-4-((3,5-dimethoxyphenyl)amino)pyrimidin-2-yl)piperazin-1-yl)-N-hydroxypyrimidine-5-carboxamide (**L20**). Crystallised from EtOAc to give a white powder (0.10 g, 50%); HRMS *m/z* [M + H]^+^ calcd for C_21_H_24_ClN_8_O_4_: 487.16090, found: 487.15234. ^1^H NMR (400 MHz, DMSO-d_6_) δ 11.11 (s, 1H), 8.77 (s, 1H), 8.66 (d, J = 33.5 Hz, 2H), 7.99 (d, J = 93.2 Hz, 1H), 7.06 (d, J = 12.6 Hz, 3H), 6.27 (d, J = 28.8 Hz, 1H), 3.89 (s, 3H), 3.79 (d, J = 3.9 Hz, 3H), 3.74 (d, J = 5.6 Hz, 8H). ^13 ^C NMR (101 MHz, DMSO) δ 160.60 (d, J = 6.9 Hz), 155.17, 101.7, 99.97, 99.09, 96.63, 55.65 (d, J = 6.4 Hz), 43.88, 43.54.

2–(4-(5-chloro-4-((3,4-dimethoxyphenyl)amino)pyrimidin-2-yl)piperazin-1-yl)-N-hydroxypyrimidine-5-carboxamide (**L21**). Crystallised from EtOAc to give a white powder (0.08 g, 40%); HRMS *m/z* [M + H]^+^ calcd for C_21_H_24_ClN_8_O_4_: 487.16090, found: 487.15234.^1^H NMR (400 MHz, DMSO-d_6_) δ 11.09 (s, 1H), 9.01 (s, 1H), 8.70 (s, 2H), 8.61 (s, 1H), 8.05 (s, 1H), 7.38 (s, 1H), 7.20 (d, J = 8.8 Hz, 1H), 6.94 (d, J = 8.7 Hz, 1H), 3.87 (s, 4H), 3.76 (s, 10H). ^13 ^C NMR (101 MHz, DMSO) δ 159.65, 155.83, 154.69, 148.65, 145.54, 132.52, 114.46, 112.11, 107.69, 102.93, 56.16, 55.86, 43.85.

2–(4-(4-([1,1′-biphenyl]-4-ylamino)-5-chloropyrimidin-2-yl)piperazin-1-yl)-N-hydroxypyrimidine-5-carboxamide (**L22**). Crystallised from EtOAc to give a white powder (0.10 g, 50%); HRMS *m/z* [M + H]^+^ calcd for C_25_H_24_ClN_8_O_2_: 503.17107, found: 503.04236. ^1^H NMR (400 MHz, DMSO-d_6_) δ 9.09 (s, 1H), 7.89 (s, 1H), 7.81 (d, J = 8.0 Hz, 3H), 7.67 (t, J = 7.9 Hz, 6H), 7.47 (t, J = 7.3 Hz, 3H), 7.36 (d, J = 7.3 Hz, 1H), 3.90 (s, 2H), 3.76 (d, J = 4.6 Hz, 6H). ^13 ^C NMR (101 MHz, DMSO) δ 171.88, 158.85, 155.38, 142.38, 140.08, 137.88, 136.63, 129.42, 127.70, 126.84 (t, J = 9.3 Hz), 124.13, 122.68, 99.24, 43.63.

### *In vitro* HDACs inhibitory assay

All HDAC enzymes were purchased from BPS Bioscience. In short, 60 µL of recombinant HDAC enzyme solution was mixed with various concentrations of test compound (40 µL), and then incubated at 37 °C for 30 minutes^17^. The reaction was terminated by adding 100 µL of imaging agent containing trypsin and trichostatin A (TSA). After standing for 20 min, the fluorescence intensity was measured at the excitation and emission wavelengths of 360 and 460 nm with a microplate reader. The inhibition rate was calculated from the fluorescence intensity readings of the test wells relative to the control wells, and the IC_50_ curve and value were determined by GraphPad Prism 6.0 software.

### Molecular docking

Molecular docking was performed using Glide in Schrodinger Suites 2018. Crystal structure of HDAC3 (PDB Entry: 4A69) was derived from RCSB protein data bank (www.rcsb.org). Structural modifications were performed by Protein Preparation Wizard. The embedded water and metals molecules in the protein structure were removed. OPLS 2005 force field was assigned to the refined protein. The structure of SAHA, PXD101, LBH589 and molecule **L20** was sketched by maestro and prepared by LigPrep. The docked ligand was confined to an enclosing box with centroid of zinc ion. Extra precision was applied in the docking process, and other parameters were set as default.

### *In vitro* antiproliferative assay

The proliferation of cancer cells was tested by CCK-8 assay. Briefly, cells were seeded in 96-well plate with about 5 × 10^3^ cells in each well. The cells were treated with tested compounds after 24 h of incubation. CCK-8 reagent (10 ml) was added to each well after 72 h of incubation, and cells were incubated at 37 °C for 4 h. The light absorbance at 450 nm was measured by using an Opsys microplate reader (Dynex Technologies, Chantilly, VA, USA). Results are illustrated as percent of cell viability normalised to DMSO-treated control cells.

### Cell cycle analysis

K562 cells were incubated with different doses of molecule **L20** and SAHA for 24 h. After treatment, cells were collected and fixed with 70% pre-cold ethanol in PBS and stored at −20 °C overnight. Then washed the cells with PBS twice, and incubated with 100 µg/mL RNase I (Solarbio, China) at 37 °C for 1 h, stained with propidium iodide (PI, 10 µg/mL, Solarbio, China) for 30 min avoid light at room temperature. Finally, DNA content was measured by flow cytometry (FACSAriaIII, Becton Dickinson, USA). The data were analysed and fitted by ModFit software.

### Cell apoptosis analysis

K562 cells were treated with various concentrations of molecule **L20** and SAHA for 24 h, cells were harvested and PBS washed twice, then resuspended with binding buffer (Becton Dickinson, USA). Cells were incubated with Annexin V-BV421 (Becton Dickinson, USA) and 7-AAD (Becton Dickinson, USA) double labelling for 30 min in the dark at room temperature and measured by flow cytometry (FACSAriaIII, Becton Dickinson, USA). The data was analysed using Flowjo-V10 software.

### Statistical analysis

All experiments were repeated at least three times unless otherwise stated. The data were represented as mean ± SD. Statistical analyses were performed with Student’s *t* test for two group comparisons and using one-way ANOVA with Tukey’s *post hoc* test for multigroup comparisons.

## Supplementary Material

Supplemental Material
