# Supplementary material for: Discovery and SAR analysis of 5-chloro-4-((substituted phenyl)amino)pyrimidine bearing histone deacetylase inhibitors
Source: J Enzyme Inhib Med Chem. 2022 Jul 14;37(1):1918–27. doi: 10.1080/14756366.2022.2097446 (PMC9291665; doi:10.1080/14756366.2022.2097446)
Supplement: Supplemental Material [file IENZ_A_2097446_SM1775.pdf]

# Discovery and SAR Analysis of 5-chloro-4-((substituted phenyl)amino)pyrimidine Bearing Histone Deacetylase inhibitors

Lin Zhang [a†], Yiming Chen[b†], Lihui Zhang[c], Jinhong Feng[d] and Lei Zhang[a]*

[a] *Department of Medicinal Chemistry, School of Pharmacy, Weifang Medical University, Weifang, Shandong, China;*

[b]*School of Medicine and Pharmacy, Ocean University of China, Qingdao, Shandong, China;*

[c] *School of Stomatology, Weifang Medical University, Weifang, Shandong, China;*

[d] *Shandong Analysis and Test Center, Qilu University of Technology (Shandong Academy of sciences), Jinan, Shandong, China.*

[†]*These authors have contributed equally to this work.*

Author for correspondence: Tel./fax: +86-536-8462014, E-mail: leizhangchemical@gmail.com.

# Contents

HRMS and $^{1}$H-NMR spectrum of **b1** ........ 1

HRMS and $^{1}$H-NMR spectrum of **b2** ........ 2

HRMS and $^{1}$H-NMR spectrum of **b3** ........ 3

HRMS and $^{1}$H-NMR spectrum of **b4** ........ 4

HRMS and $^{1}$H-NMR spectrum of **b5** ........ 5

HRMS and $^{1}$H-NMR spectrum of **b6** ........ 6

HRMS and $^{1}$H-NMR spectrum of **b7** ........ 7

HRMS and $^{1}$H-NMR spectrum of **b8** ........ 8

HRMS and $^{1}$H-NMR spectrum of **b9** ........ 9

HRMS and $^{1}$H-NMR spectrum of **b10** ........ 10

HRMS and $^{1}$H-NMR spectrum of **b11** ........ 11

HRMS and $^{1}$H-NMR spectrum of **b12** ........ 12

HRMS and $^{1}$H-NMR spectrum of **b13** ........ 13

HRMS and $^{1}$H-NMR spectrum of **b14** ........ 14

HRMS and $^{1}$H-NMR spectrum of **b15** ........ 15

HRMS and $^{1}$H-NMR spectrum of **b16** ........ 16

HRMS and $^{1}$H-NMR spectrum of **b17** ........ 17

HRMS and $^{1}$H-NMR spectrum of **b18** ........ 18

HRMS and $^{1}$H-NMR spectrum of **b19** ........ 19

HRMS and $^{1}$H-NMR spectrum of **c1** ........ 20

HRMS and $^{1}$H-NMR spectrum of **c2** ........ 21

HRMS and $^{1}$H-NMR spectrum of **c3** ........ 22

HRMS and $^{1}$H-NMR spectrum of **c4** ........ 23

HRMS and $^{1}$H-NMR spectrum of **c5**........24
HRMS and $^{1}$H-NMR spectrum of **c6**........25
HRMS and $^{1}$H-NMR spectrum of **c7**........26
HRMS and $^{1}$H-NMR spectrum of **c8**........27
HRMS and $^{1}$H-NMR spectrum of **c9**........28
HRMS and $^{1}$H-NMR spectrum of **c10**........29
HRMS and $^{1}$H-NMR spectrum of **c11**........30
HRMS and $^{1}$H-NMR spectrum of **c12**........31
HRMS and $^{1}$H-NMR spectrum of **c13**........32
HRMS and $^{1}$H-NMR spectrum of **c14**........33
HRMS and $^{1}$H-NMR spectrum of **c15**........34
HRMS and $^{1}$H-NMR spectrum of **c16**........35
HRMS and $^{1}$H-NMR spectrum of **c17**........36
HRMS and $^{1}$H-NMR spectrum of **c18**........37
HRMS and $^{1}$H-NMR spectrum of **c19**........38
HRMS and $^{1}$H-NMR spectrum of **c20**........39
HRMS and $^{1}$H-NMR spectrum of **c21**........40
HRMS and $^{1}$H-NMR spectrum of **c22**........41
HRMS and $^{1}$H-NMR spectrum of **L1**........42
$^{13}$C-NMR spectrum of **L1** and HRMS spectrum of **L2**........43
$^{1}$H-NMR spectrum and $^{13}$C-NMR of **L2**........44
HRMS and $^{1}$H-NMR spectrum of **L3**........45
$^{13}$C-NMR spectrum of **L3** and HRMS spectrum of **L4**........46
$^{1}$H-NMR spectrum and $^{13}$C-NMR of **L4**........47

HRMS and $^{1}$H-NMR spectrum of **L5**........................................................................48

$^{13}$C-NMR spectrum of **L5** and HRMS spectrum of **L6**............................................................49

$^{1}$H-NMR spectrum and $^{13}$C-NMR of **L6**.....................................................................50

HRMS and $^{1}$H-NMR spectrum of **L7**........................................................................51

$^{13}$C-NMR spectrum of **L7** and HRMS spectrum of **L8**............................................................52

$^{1}$H-NMR spectrum and $^{13}$C-NMR of **L8**.....................................................................53

HRMS and $^{1}$H-NMR spectrum of **L9**........................................................................54

$^{13}$C-NMR spectrum of **L9** and HRMS spectrum of **L10**..........................................................55

$^{1}$H-NMR spectrum and $^{13}$C-NMR of **L10**...................................................................56

HRMS and $^{1}$H-NMR spectrum of **L11**......................................................................57

$^{13}$C-NMR spectrum of **L11** and HRMS spectrum of **L12**........................................................58

$^{1}$H-NMR spectrum and $^{13}$C-NMR of **L12**...................................................................59

HRMS and $^{1}$H-NMR spectrum of **L13**......................................................................60

$^{13}$C-NMR spectrum of **L13** and HRMS spectrum of **L14**........................................................61

$^{1}$H-NMR spectrum and $^{13}$C-NMR of **L14**...................................................................62

HRMS and $^{1}$H-NMR spectrum of **L15**......................................................................63

$^{13}$C-NMR spectrum of **L15** and HRMS spectrum of **L16**........................................................64

$^{1}$H-NMR spectrum and $^{13}$C-NMR of **L16**...................................................................65

HRMS and $^{1}$H-NMR spectrum of **L17**......................................................................66

$^{13}$C-NMR spectrum of **L17** and HRMS spectrum of **L18**........................................................67

$^{1}$H-NMR spectrum and $^{13}$C-NMR of **L18**...................................................................68

HRMS and $^{1}$H-NMR spectrum of **L19**......................................................................69

$^{13}$C-NMR spectrum of **L19** and HRMS spectrum of **L20**........................................................70

$^{1}$H-NMR spectrum and $^{13}$C-NMR of **L20**...................................................................71

HRMS and $^{1}$H-NMR spectrum of **L21**........................................................................72

$^{13}$C-NMR spectrum of **L21** and HRMS spectrum of **L22**........................................................73

$^{1}$H-NMR spectrum and $^{13}$C-NMR of **L22**.................................................................74

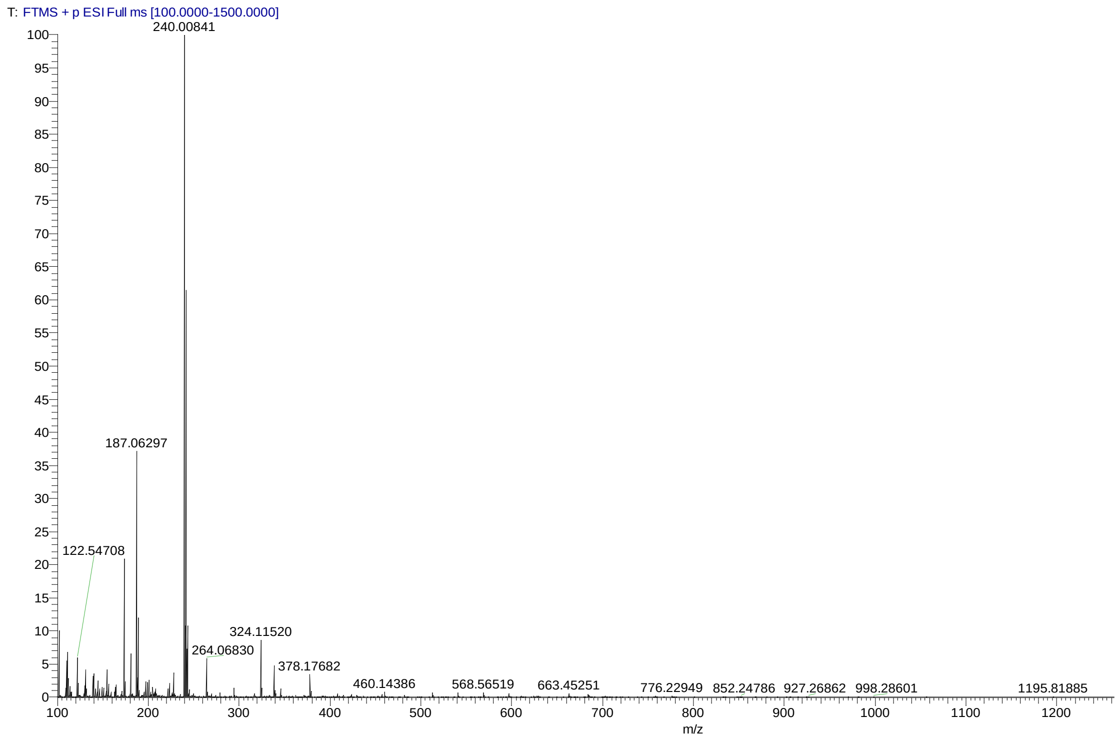


HRMS spectrum of b1

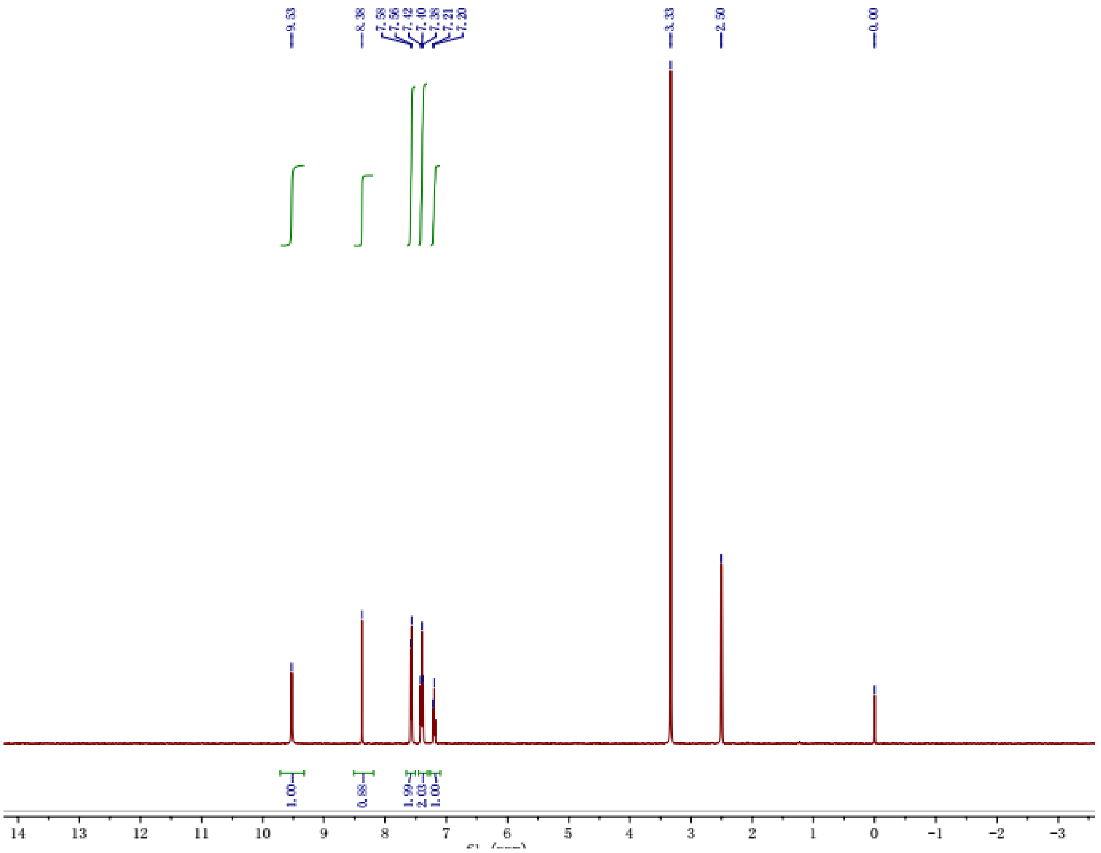


$^{1}$H-NMR spectrum of b1

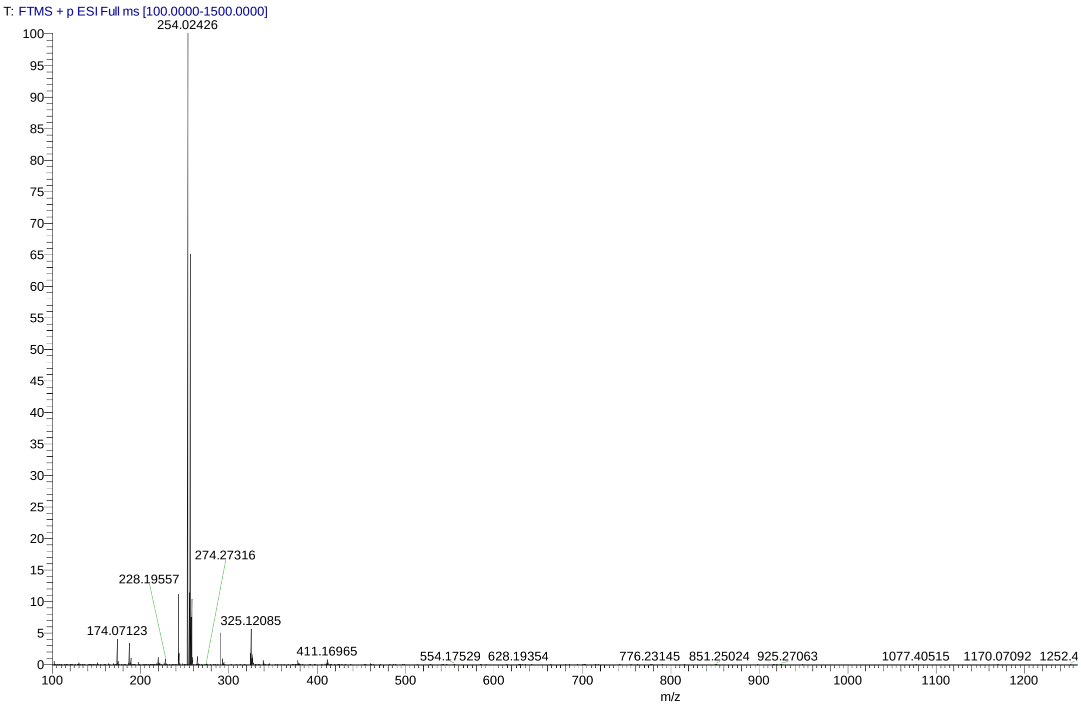


HRMS spectrum of b2

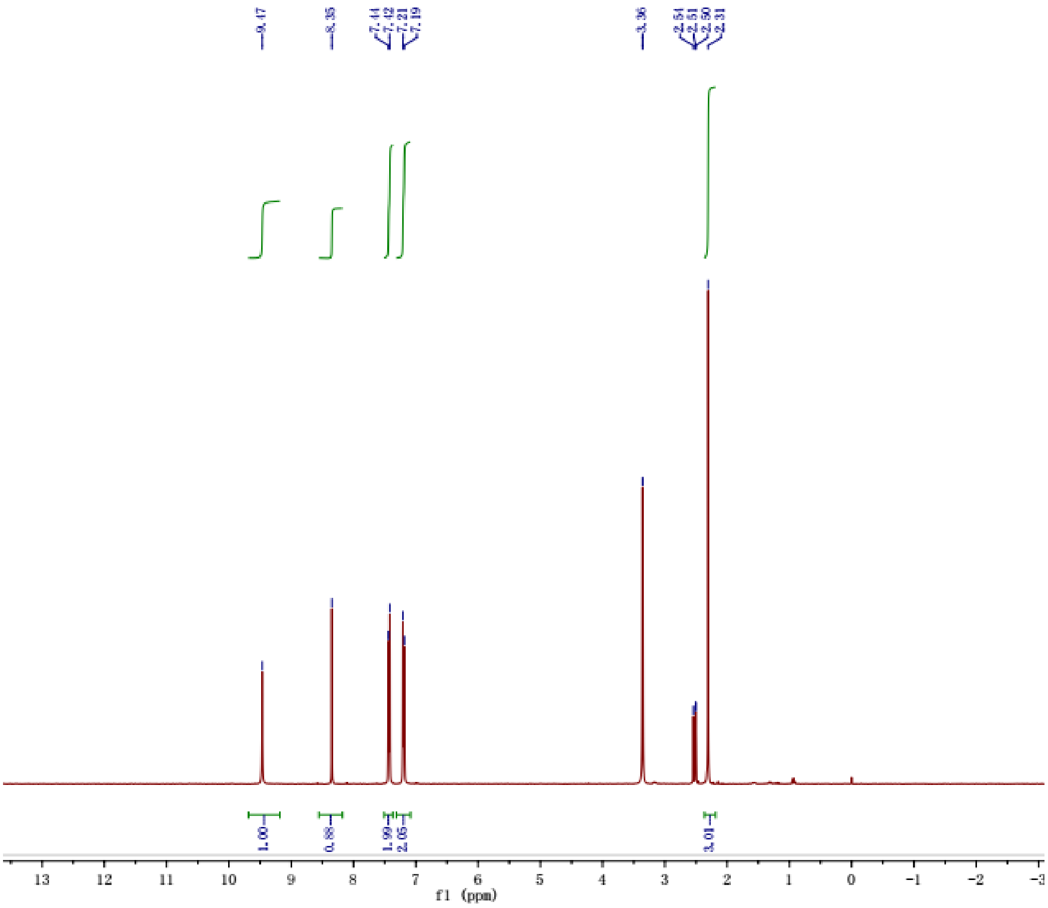


$^1$H-NMR spectrum of b2

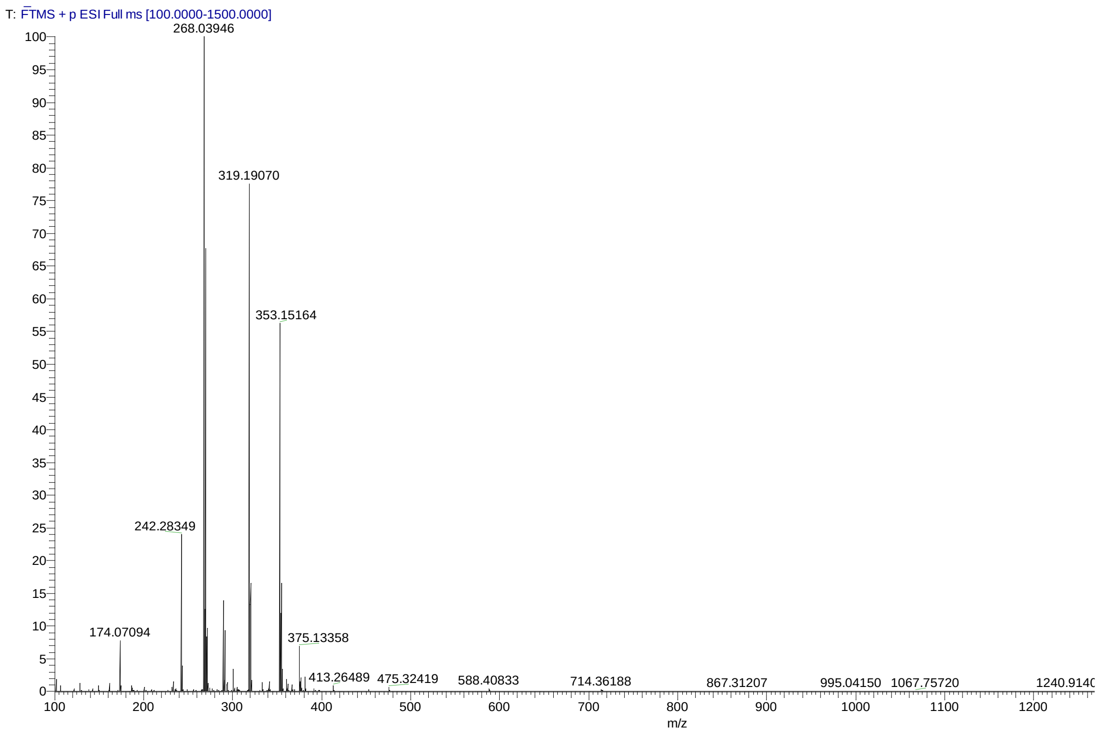


HRMS spectrum of b3

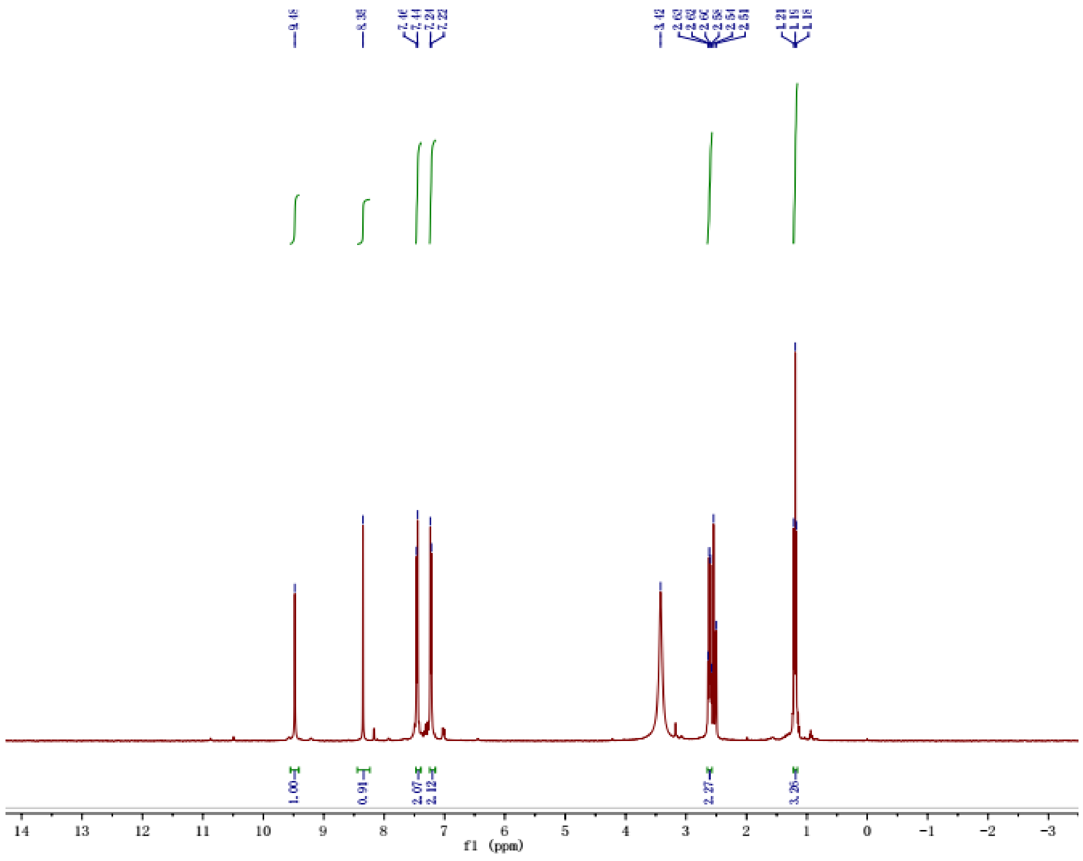


$^{1}$H-NMR spectrum of b3

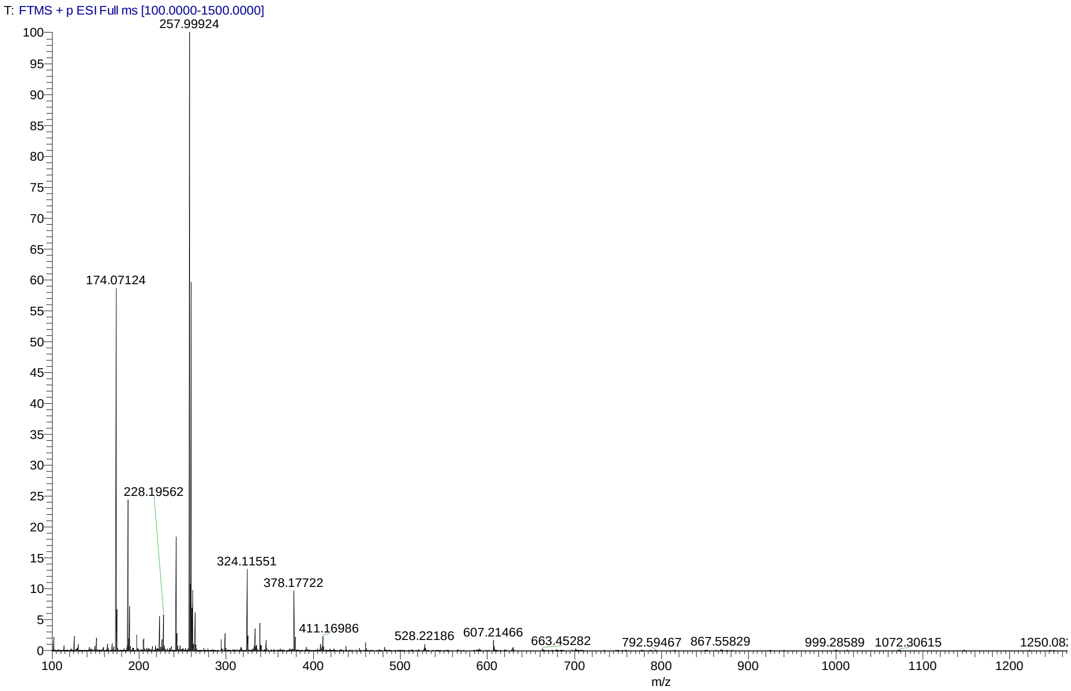


HRMS spectrum of b4

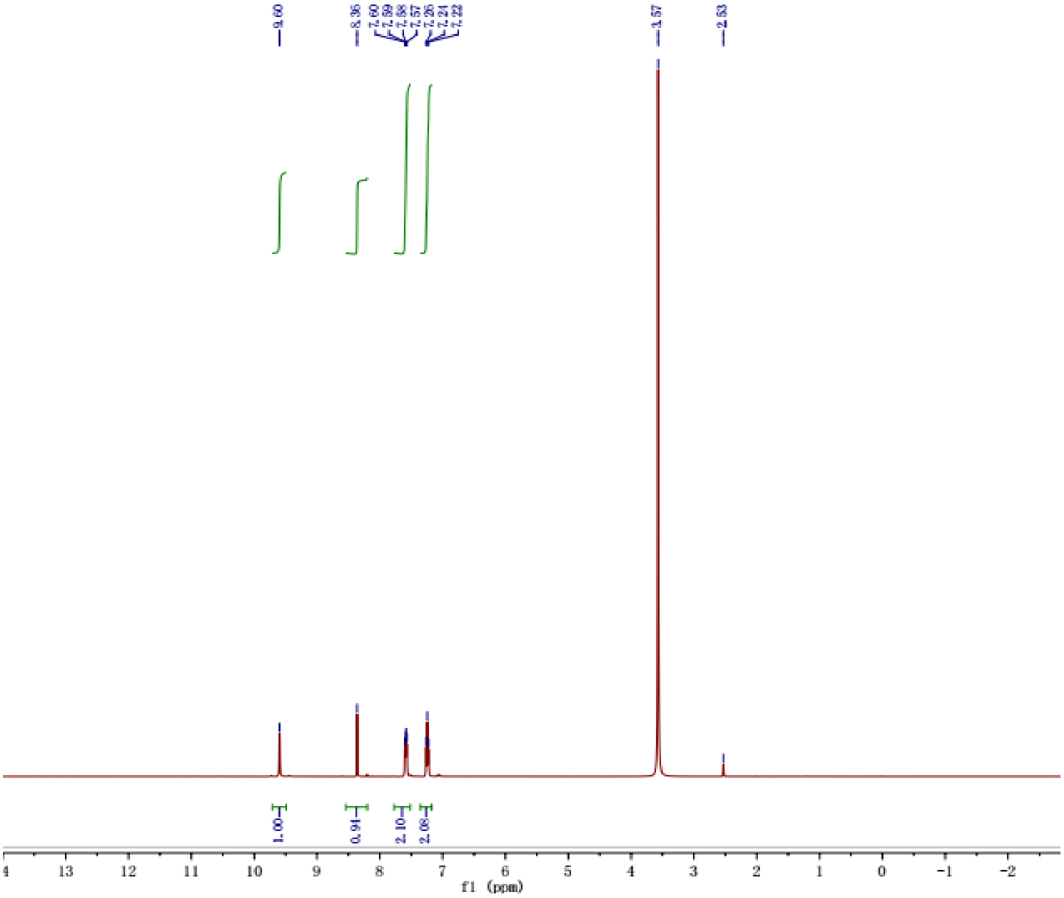


¹H-NMR spectrum of b4

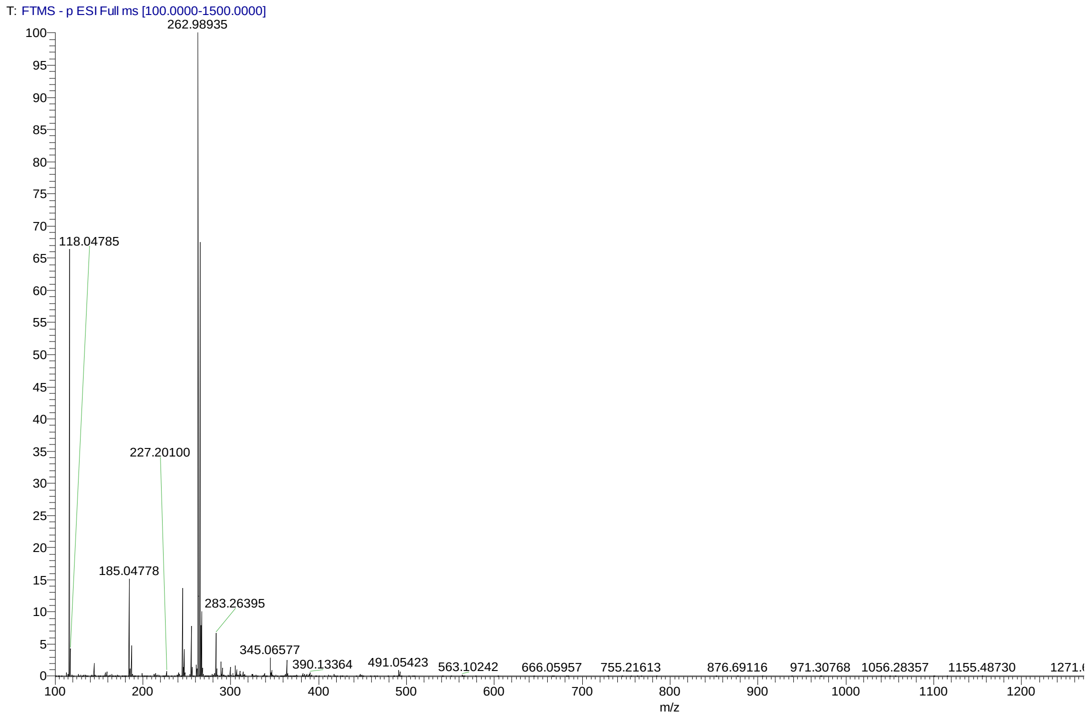


HRMS spectrum of b5

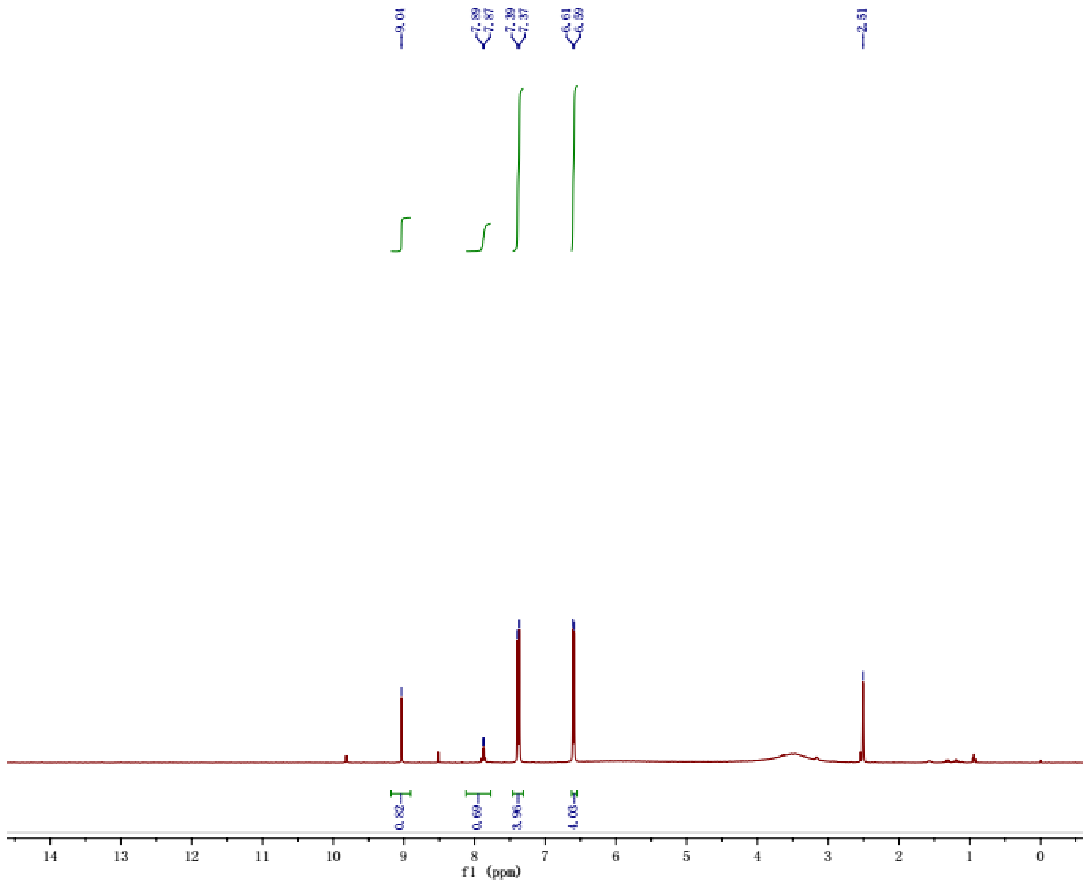


$^{1}$H-NMR spectrum of b5

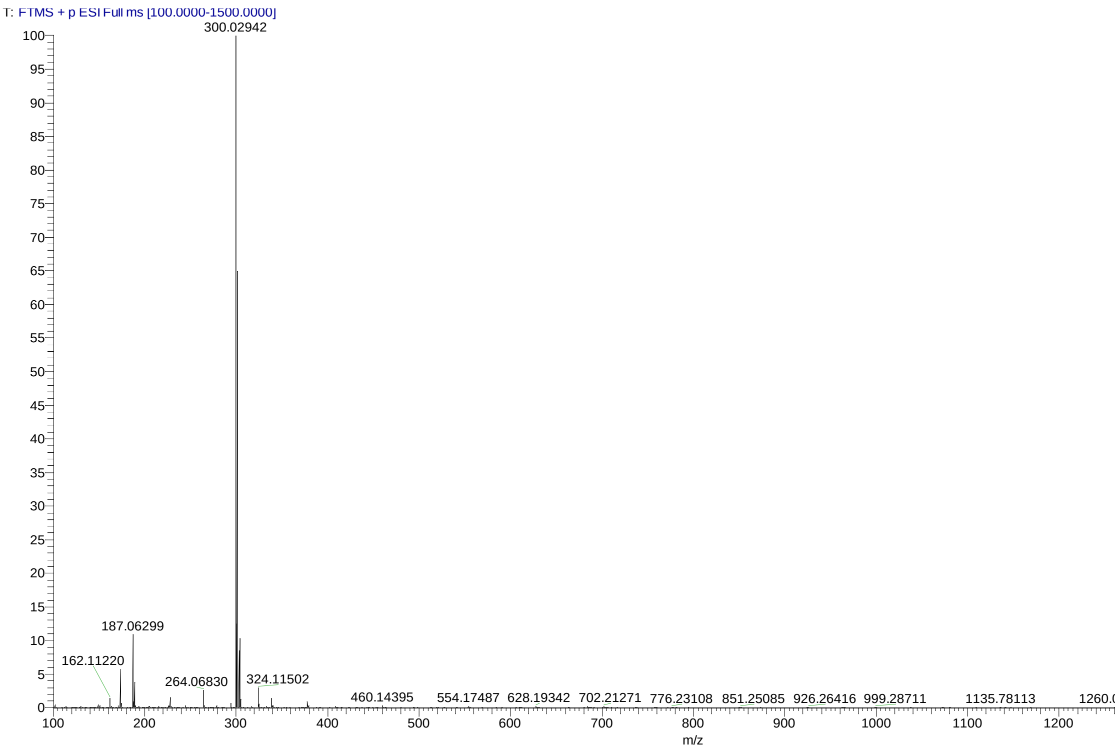


HRMS spectrum of b6

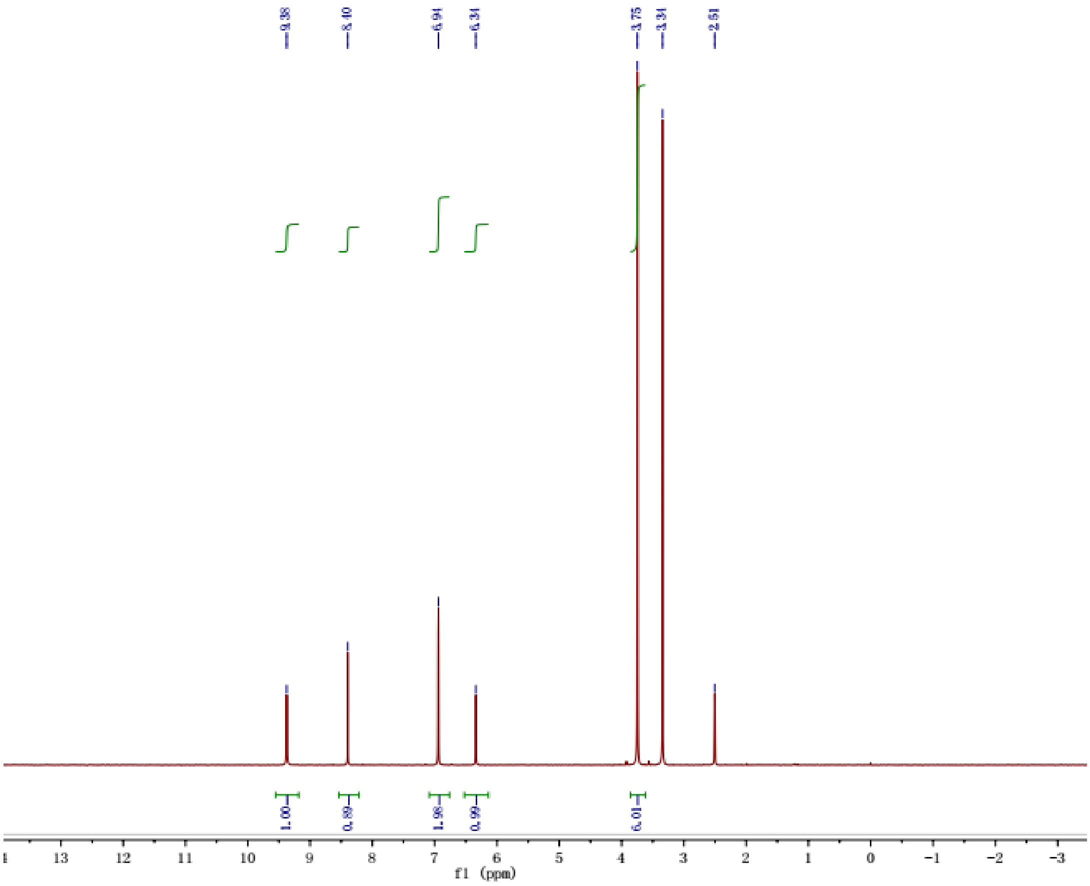


$^{1}$H-NMR spectrum of b6

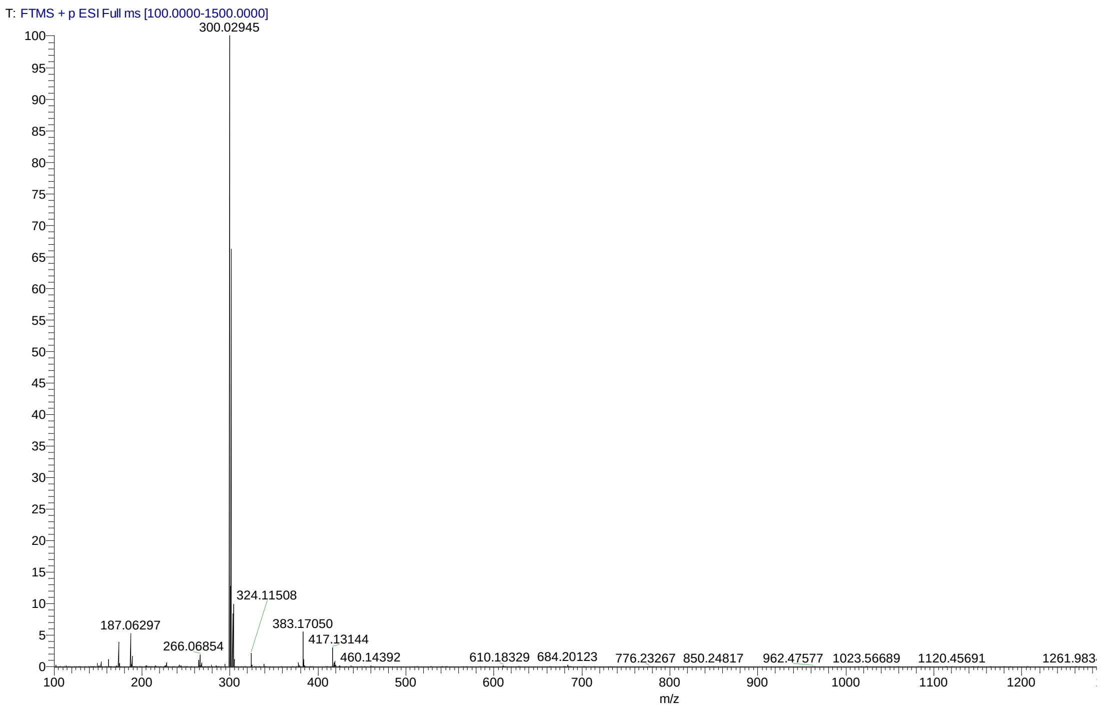


HRMS spectrum of b7

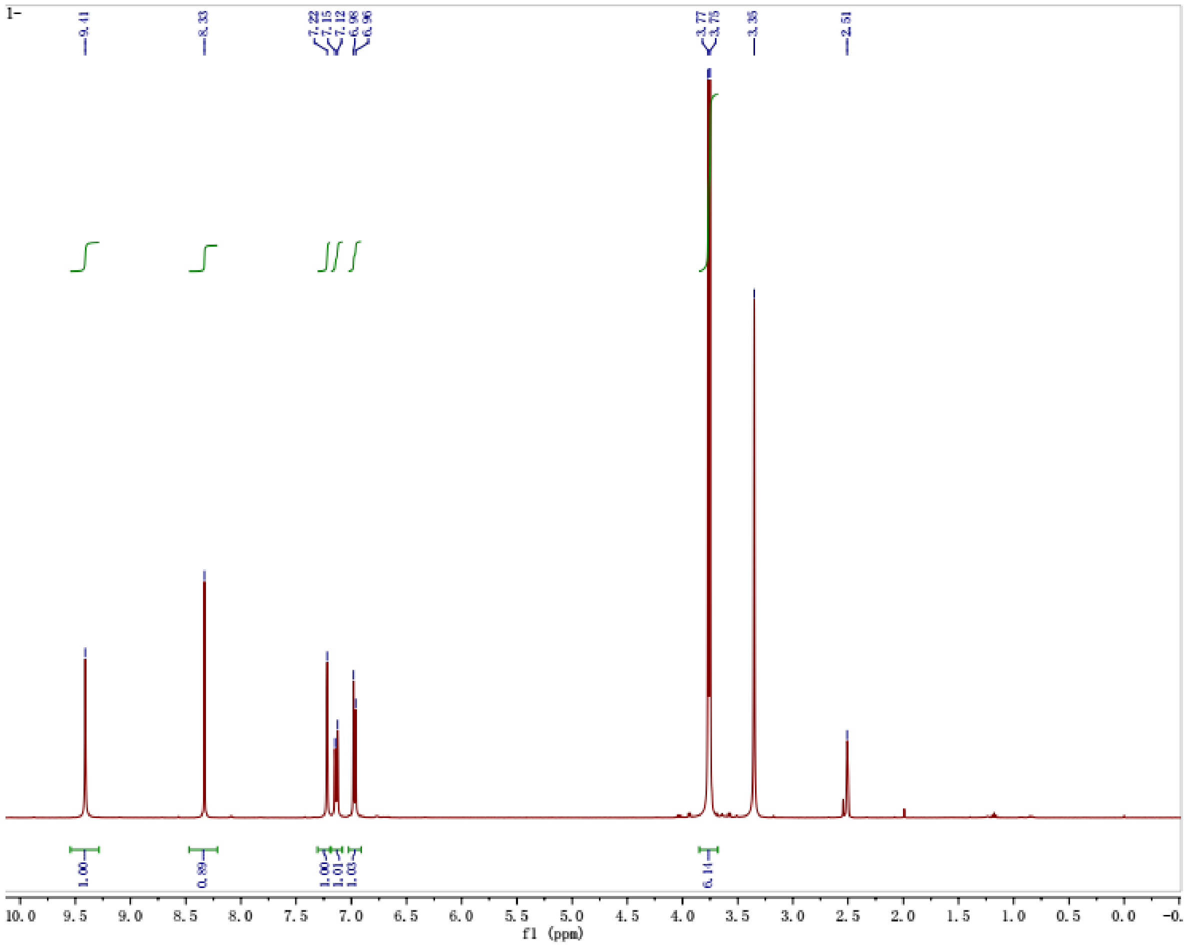


$^{1}$H-NMR spectrum of b7

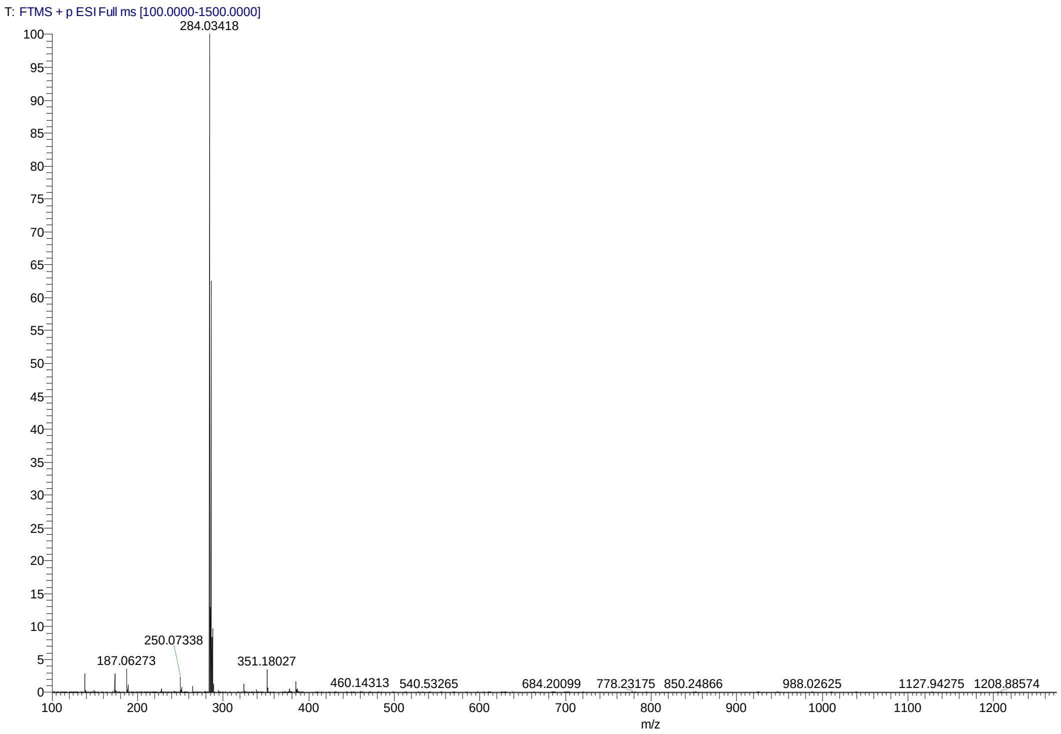


HRMS spectrum of b8

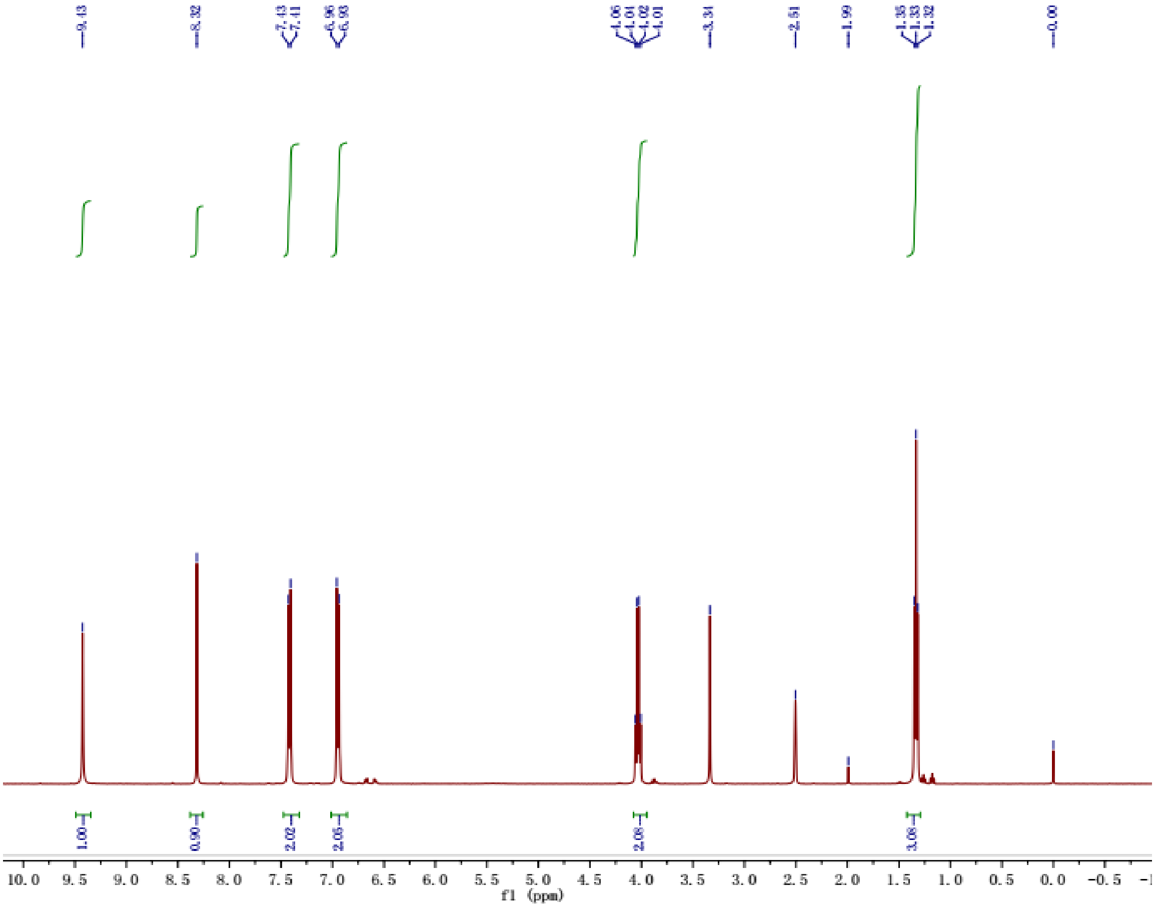


HRMS $^{1}$H-NMR spectrum of b8

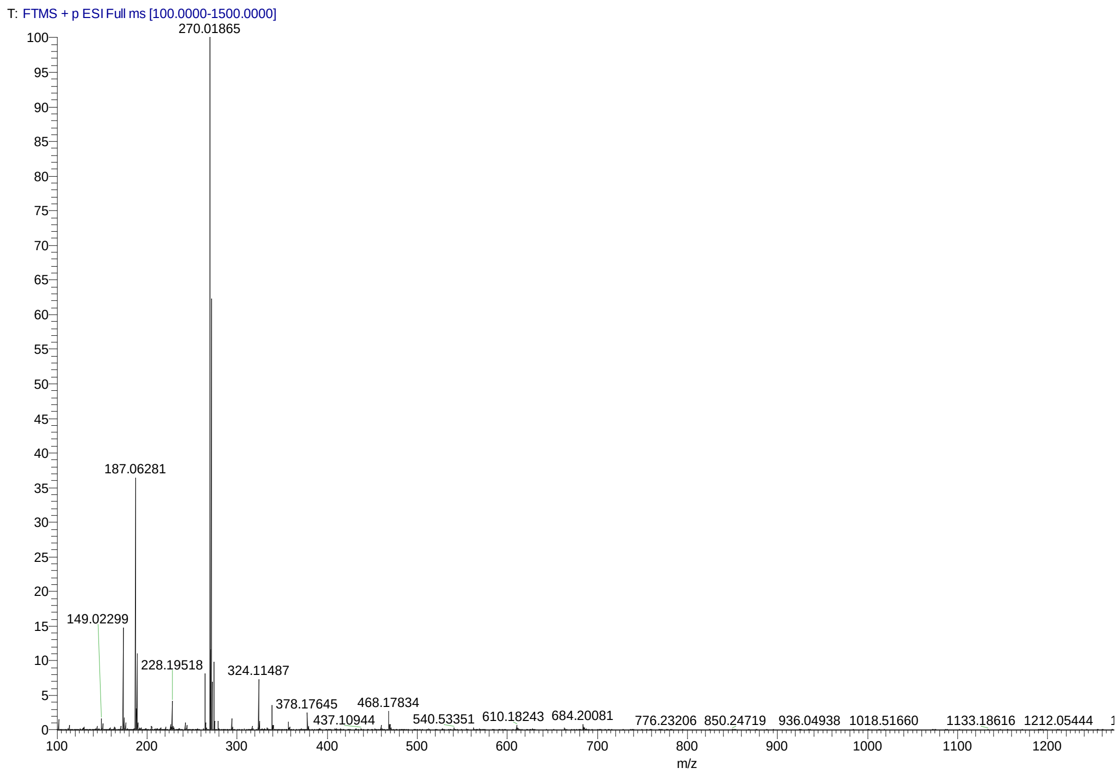


HRMS spectrum of b9

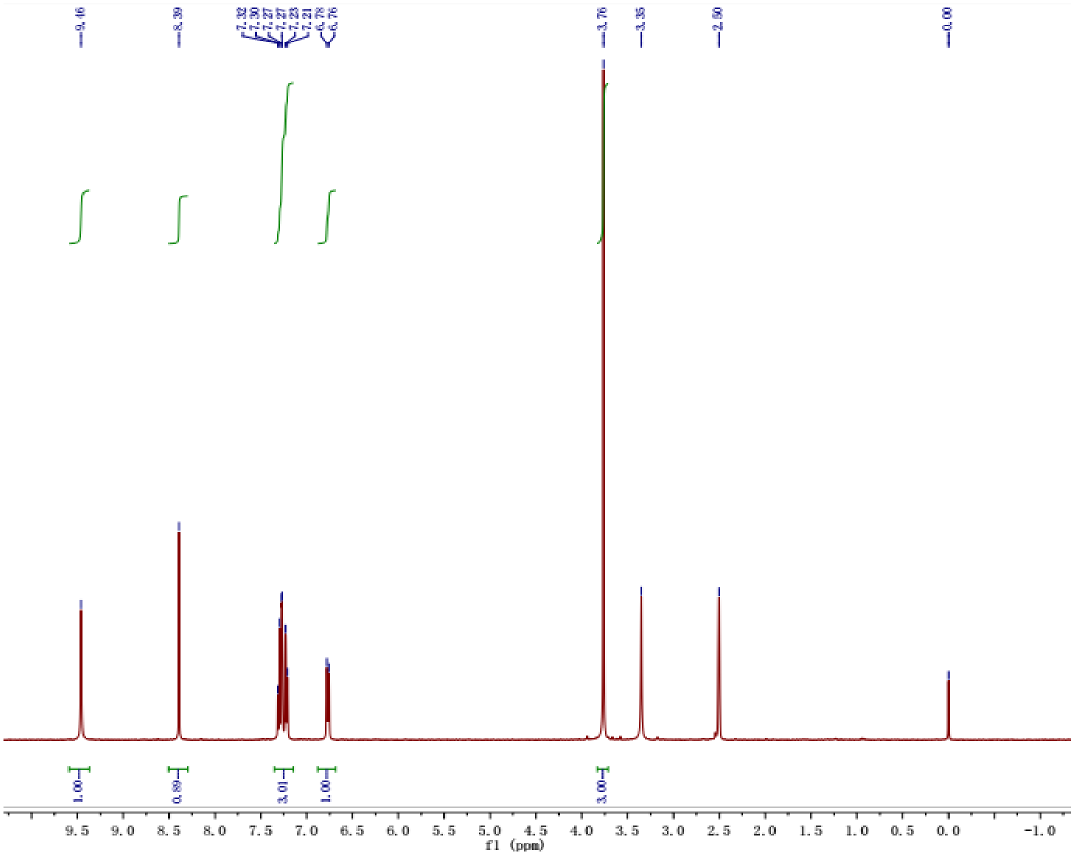


HRMS $^{1}$H-NMR spectrum of b9

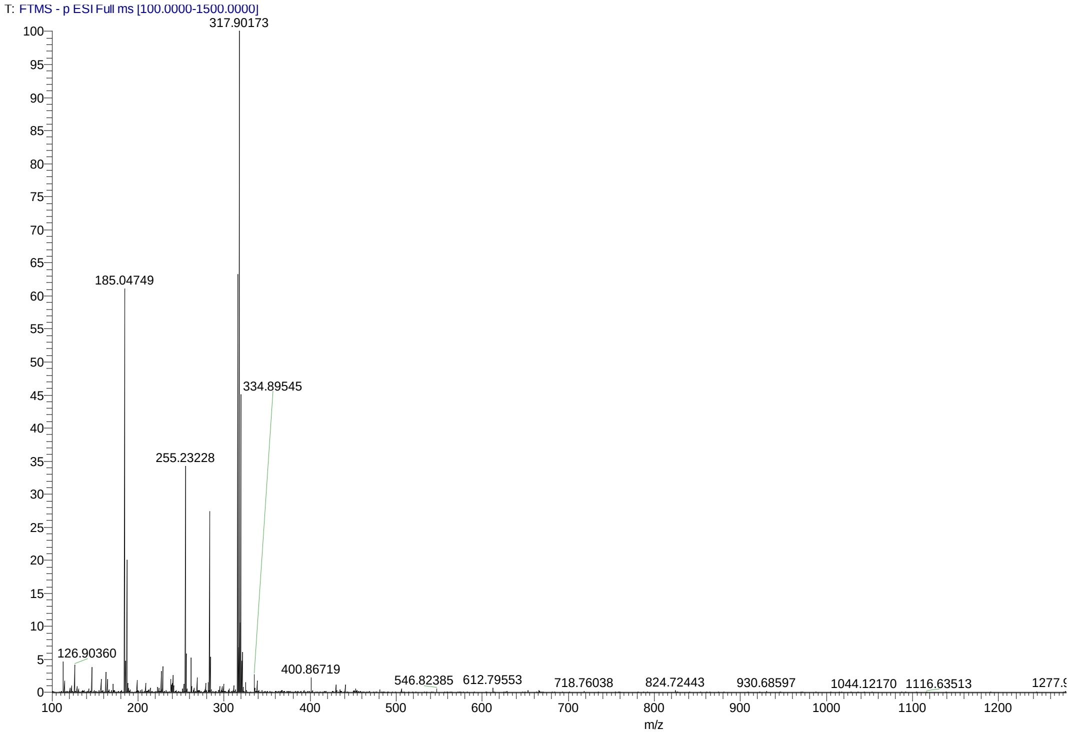


HRMS spectrum of b10

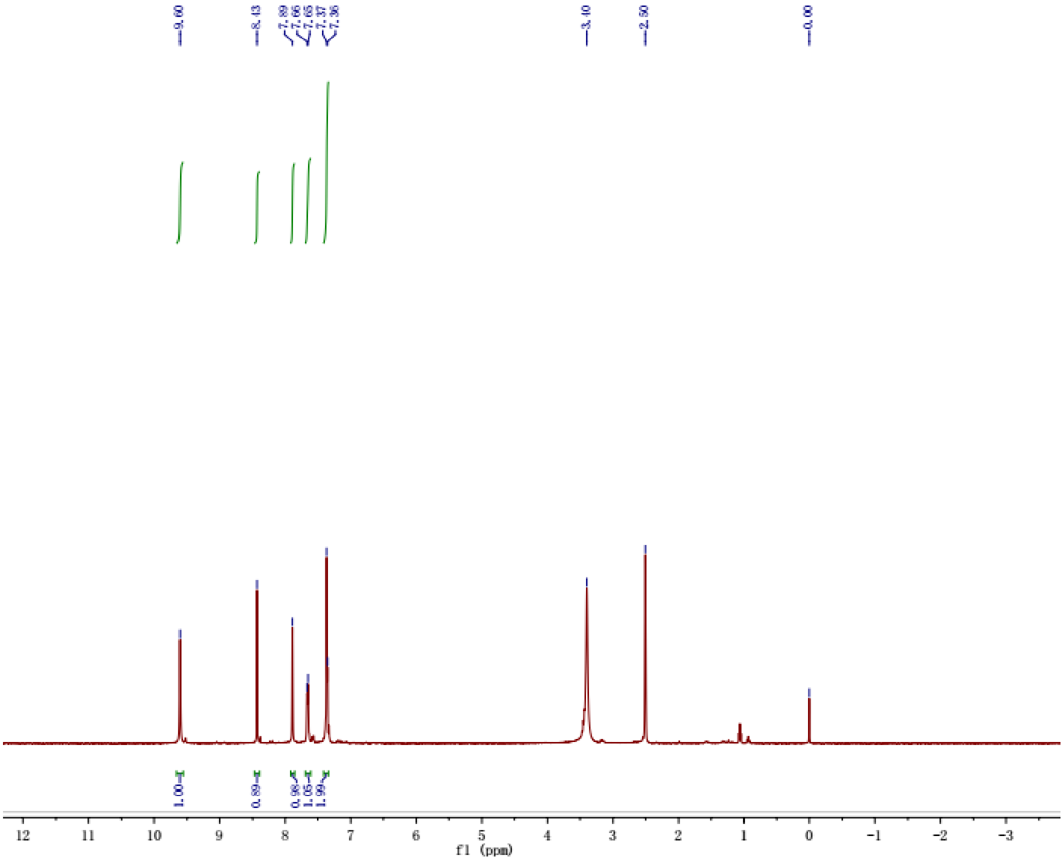


$^{1}$H-NMR spectrum of b10

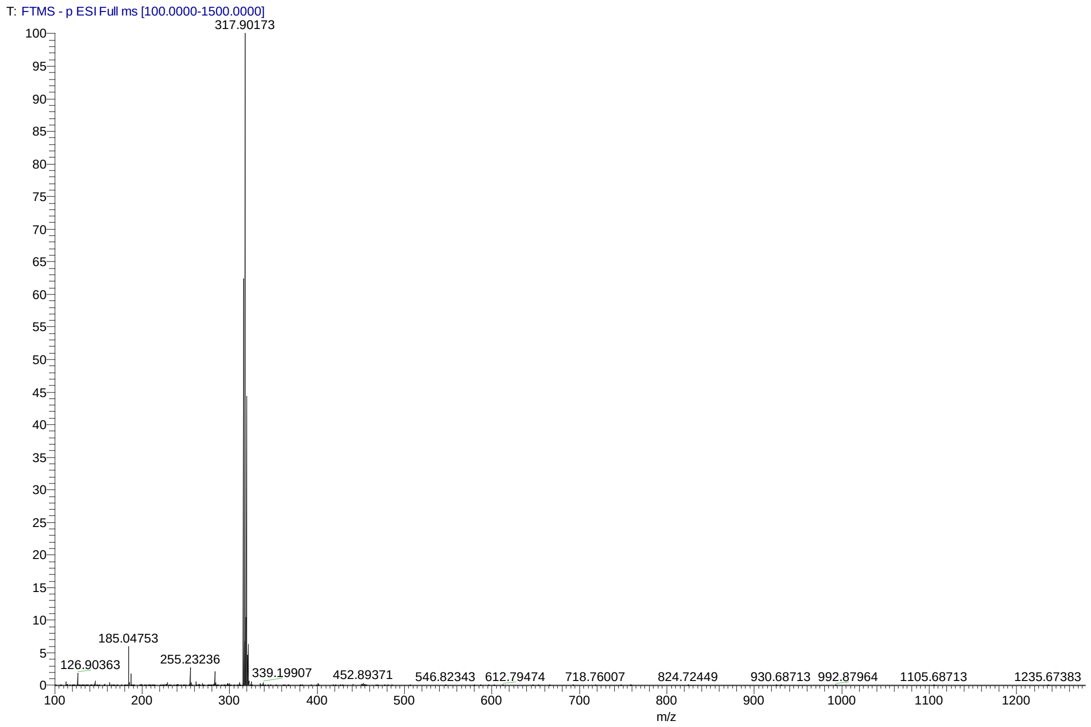


HRMS spectrum of b11

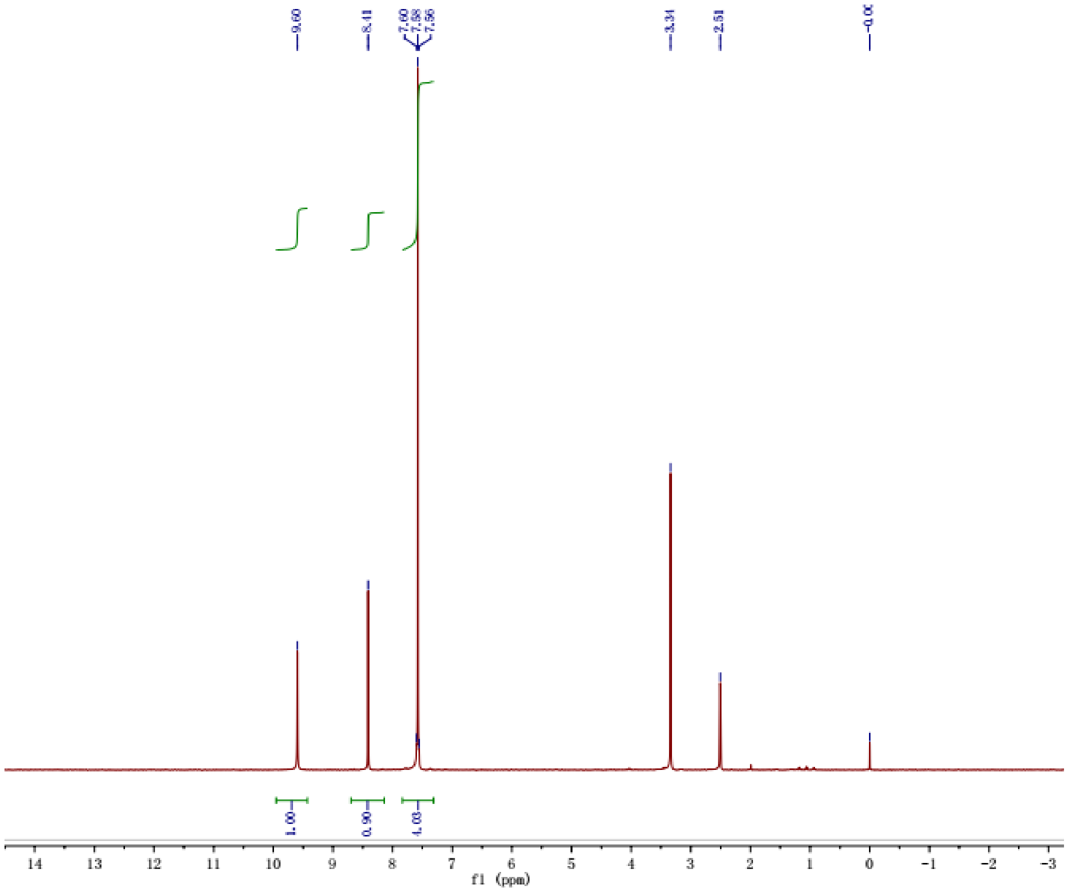


$^{1}$H-NMR spectrum of b11

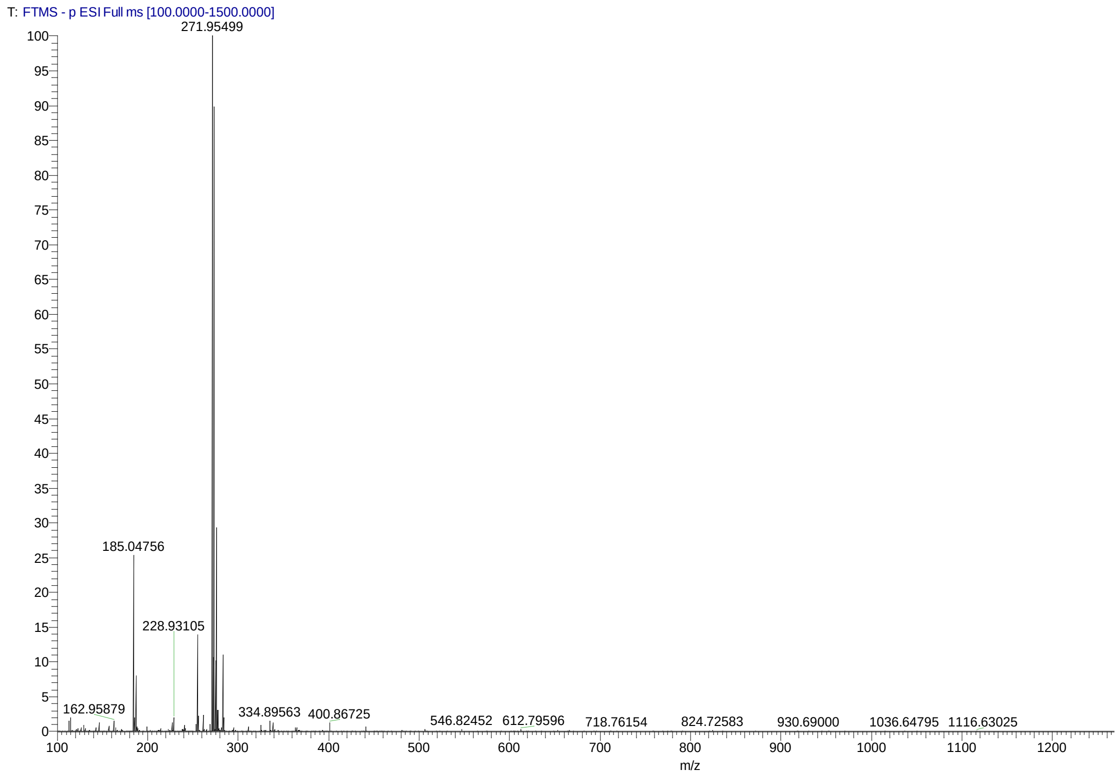


HRMS spectrum of b12

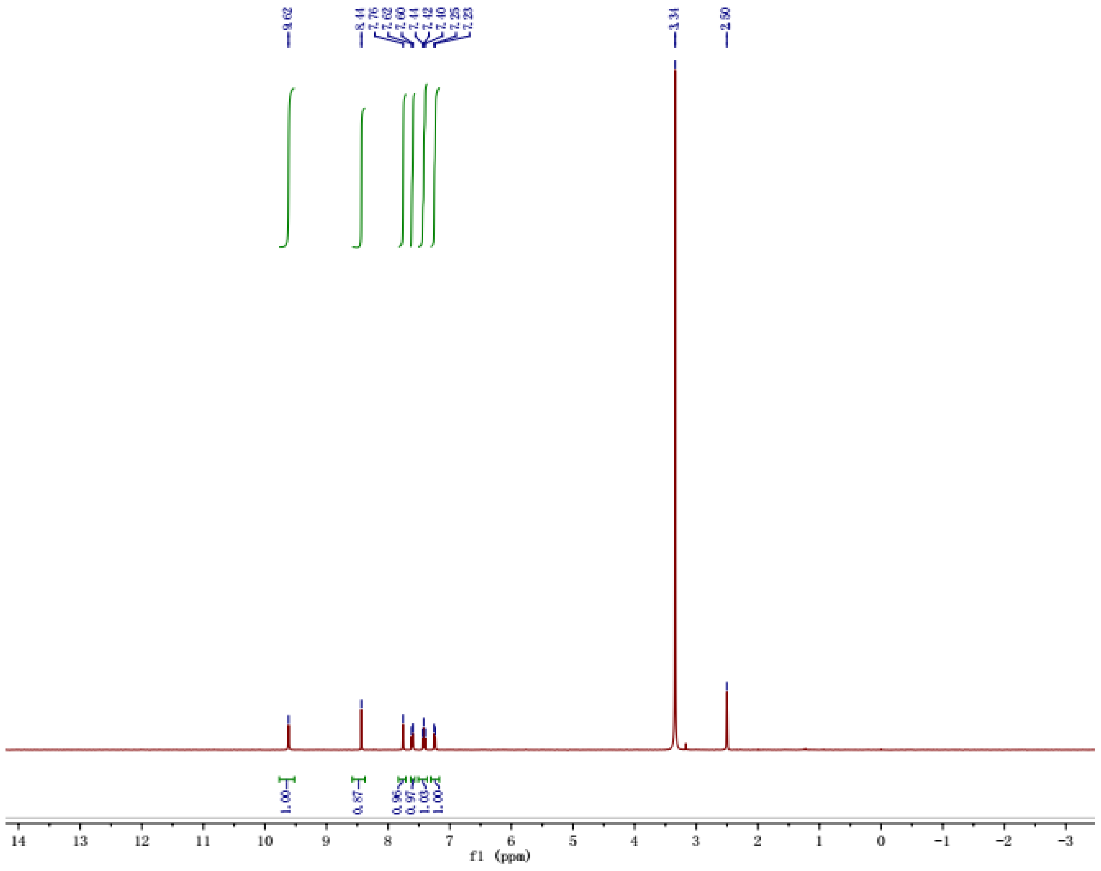


$^{1}$H-NMR spectrum of b12

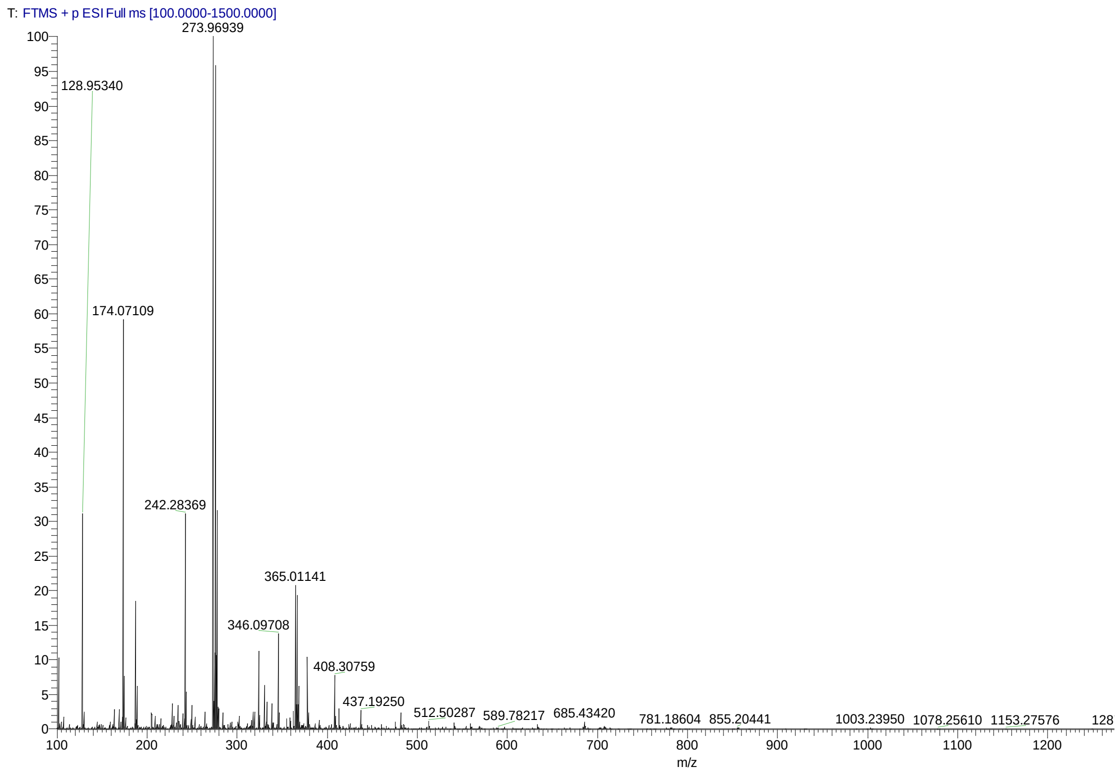


HRMS spectrum of b13

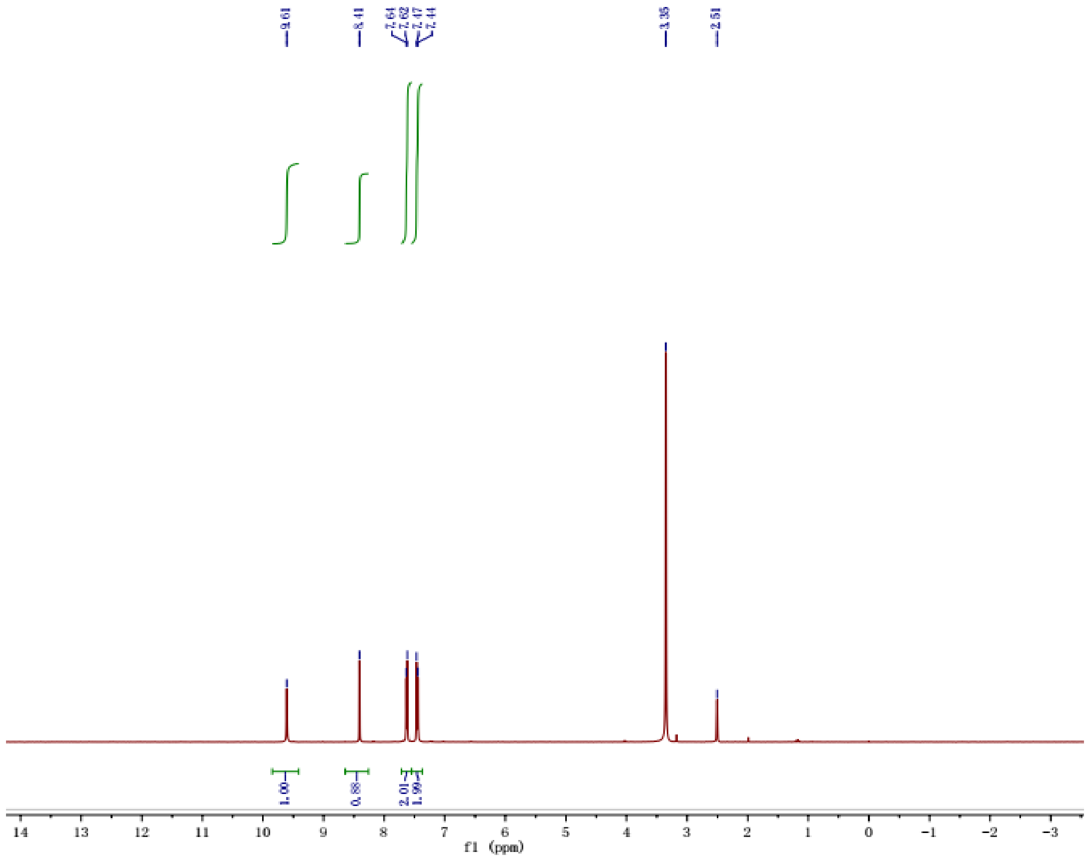


¹H-NMR spectrum of b13

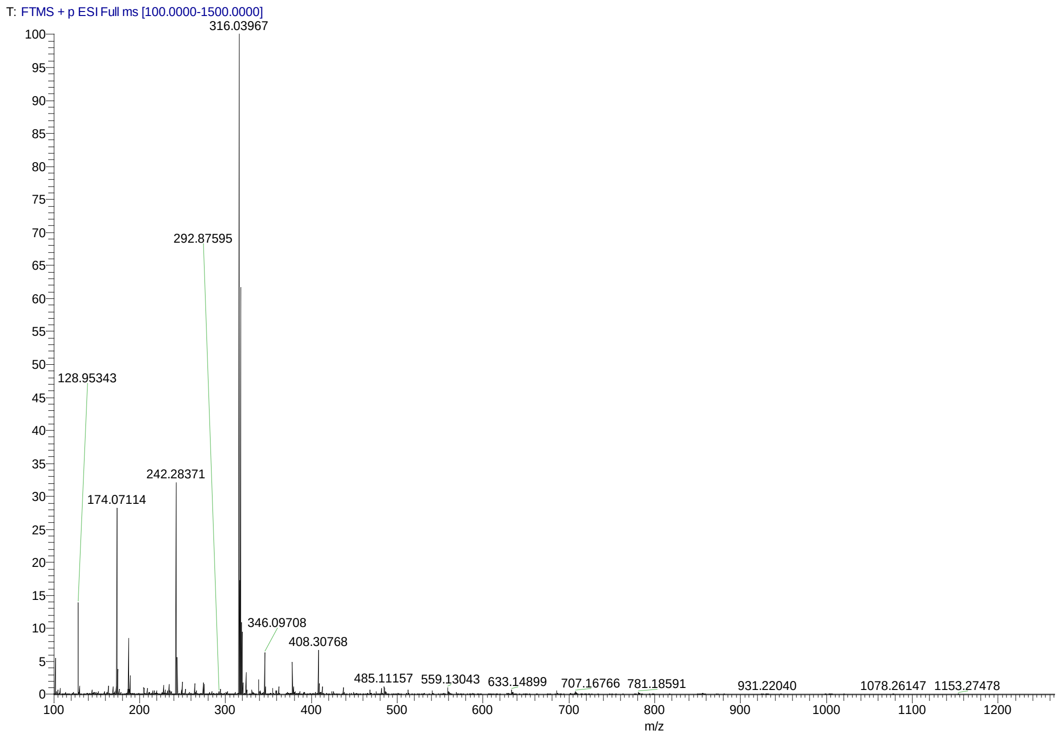


HRMS $^{1}$H-NMR spectrum of b14

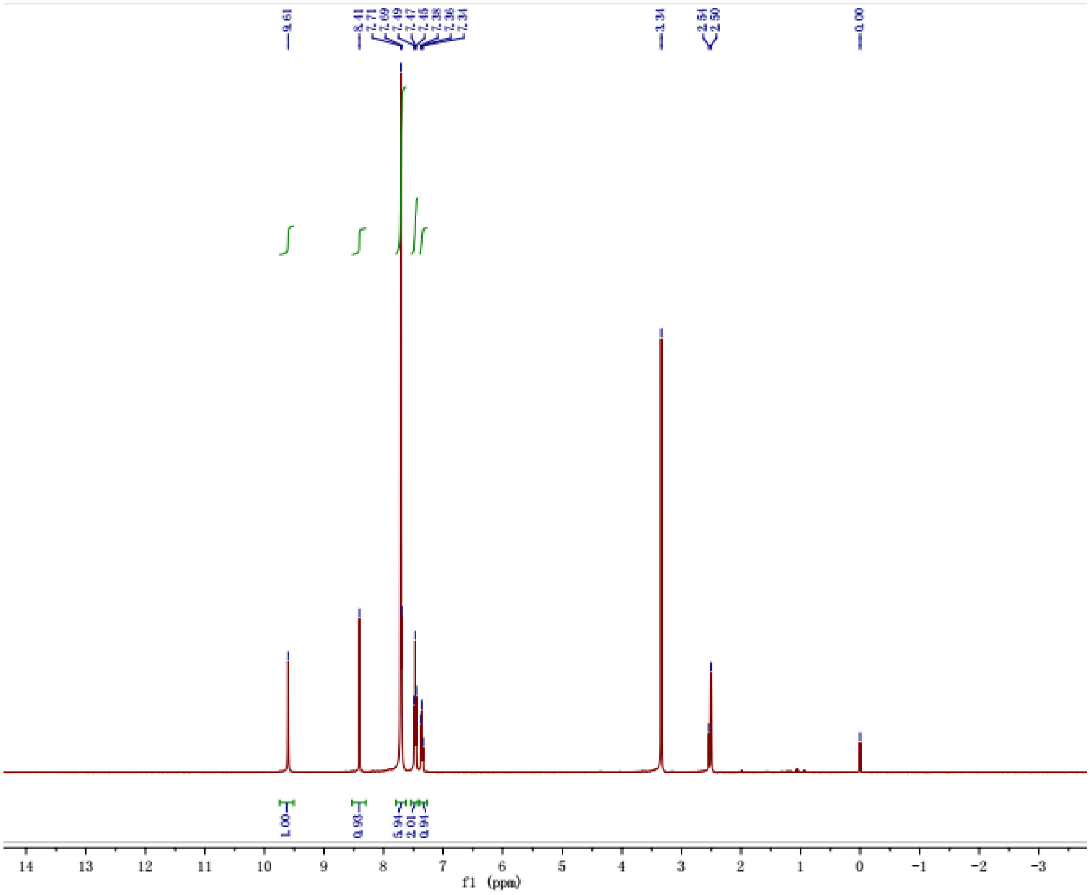


HRMS $^{1}$H-NMR spectrum of b14

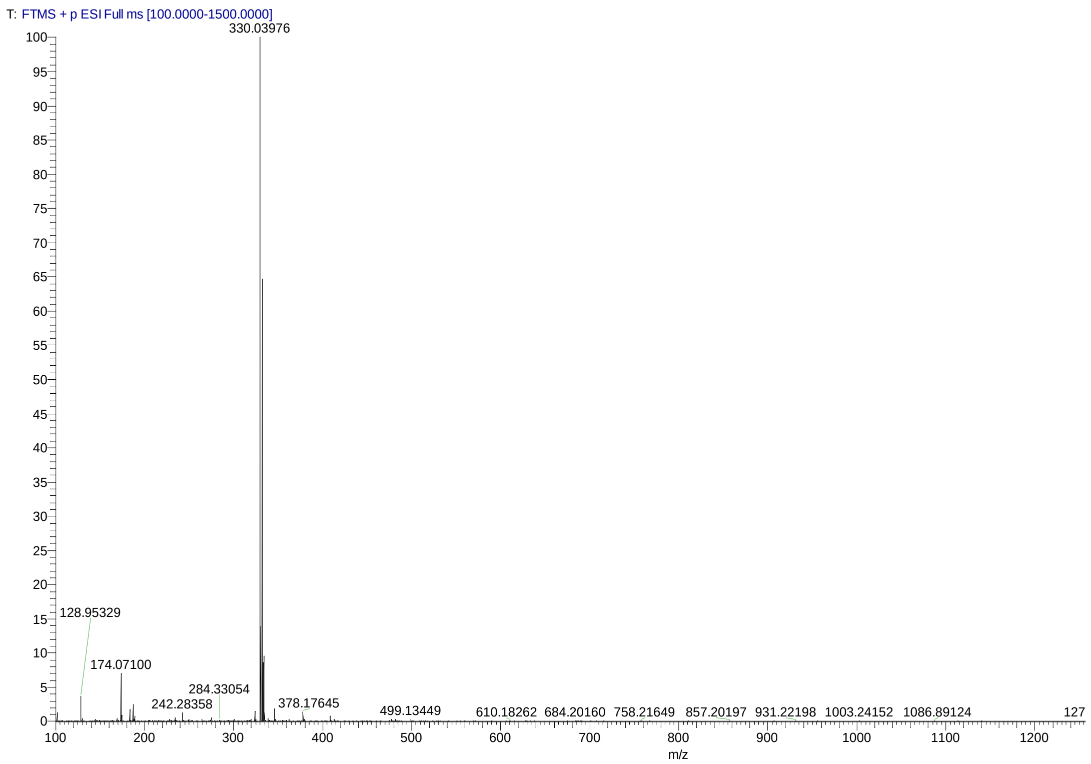


HRMS spectrum of b15

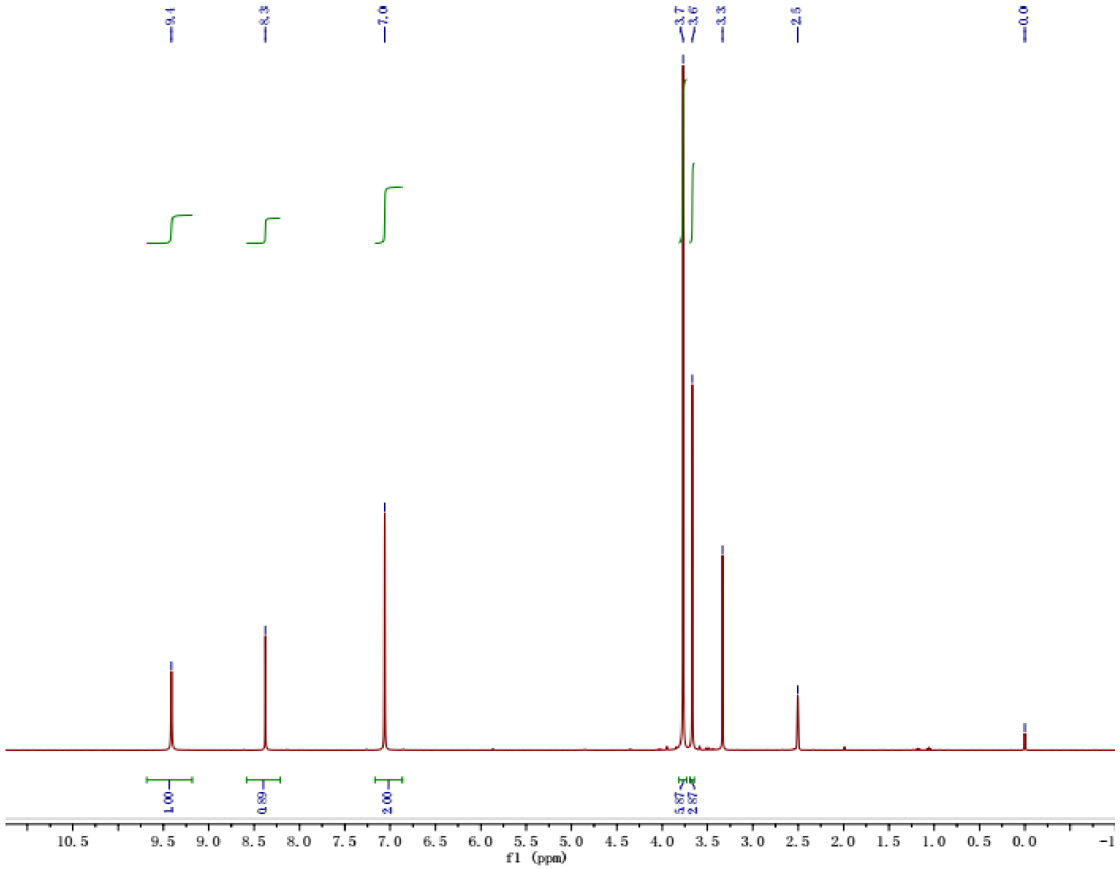


$^{1}$H-NMR spectrum of b15

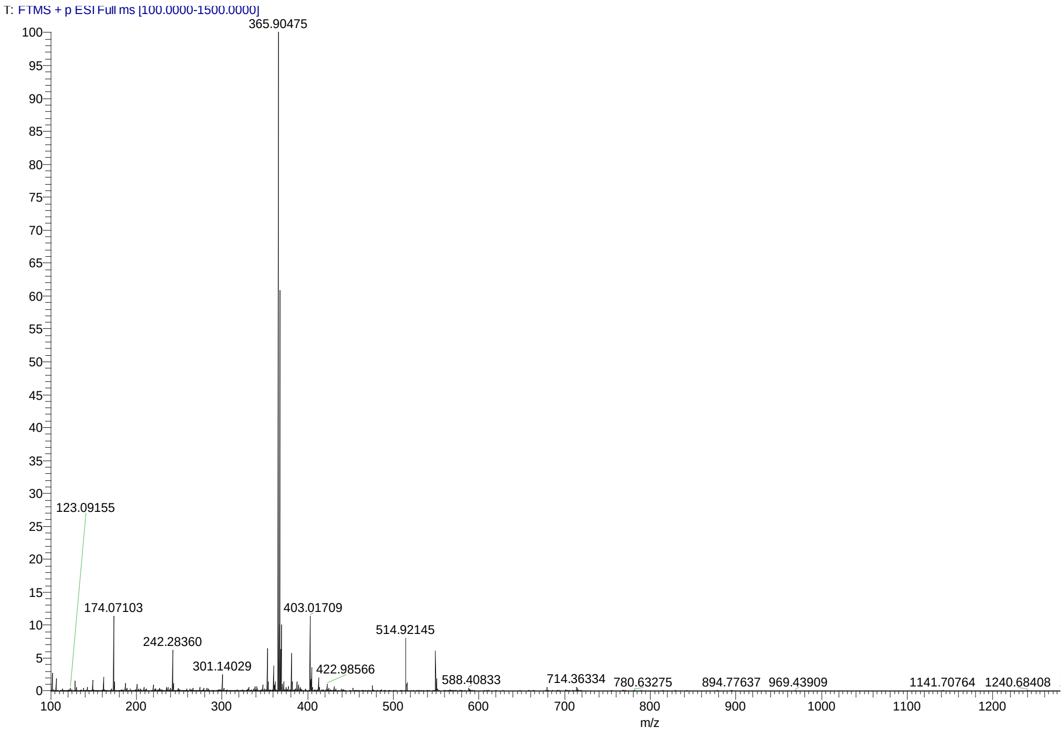


HRMS spectrum of b16

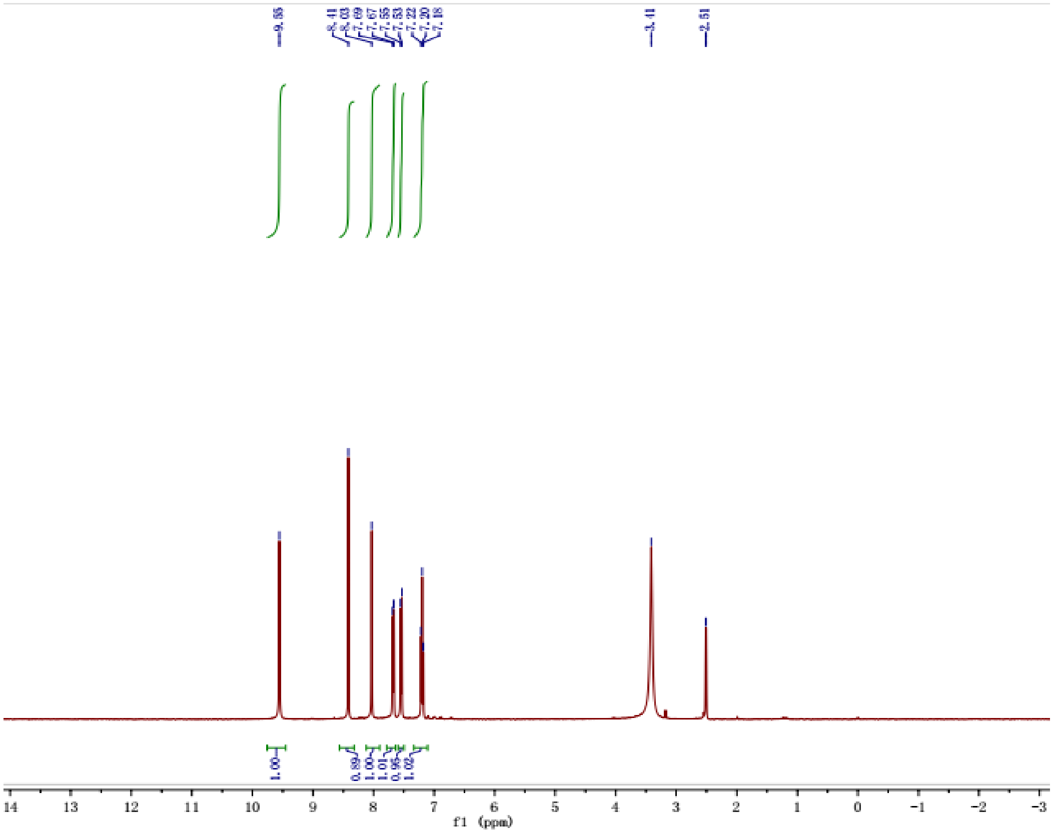


$^{1}$H-NMR spectrum of b16

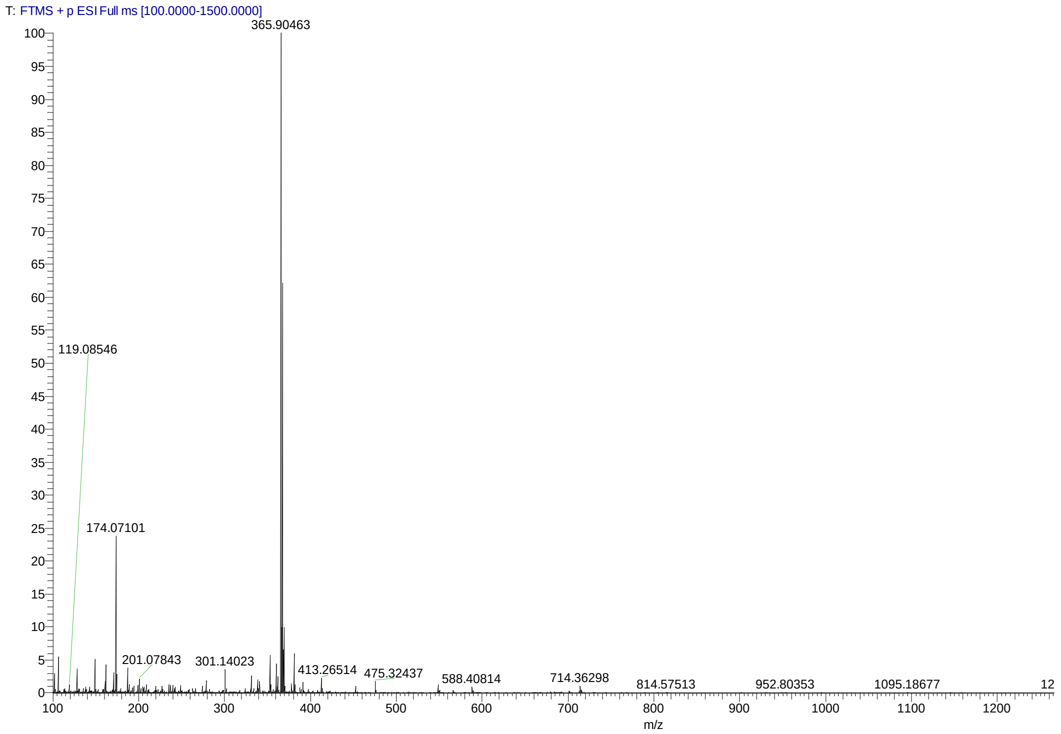


HRMS spectrum of b17

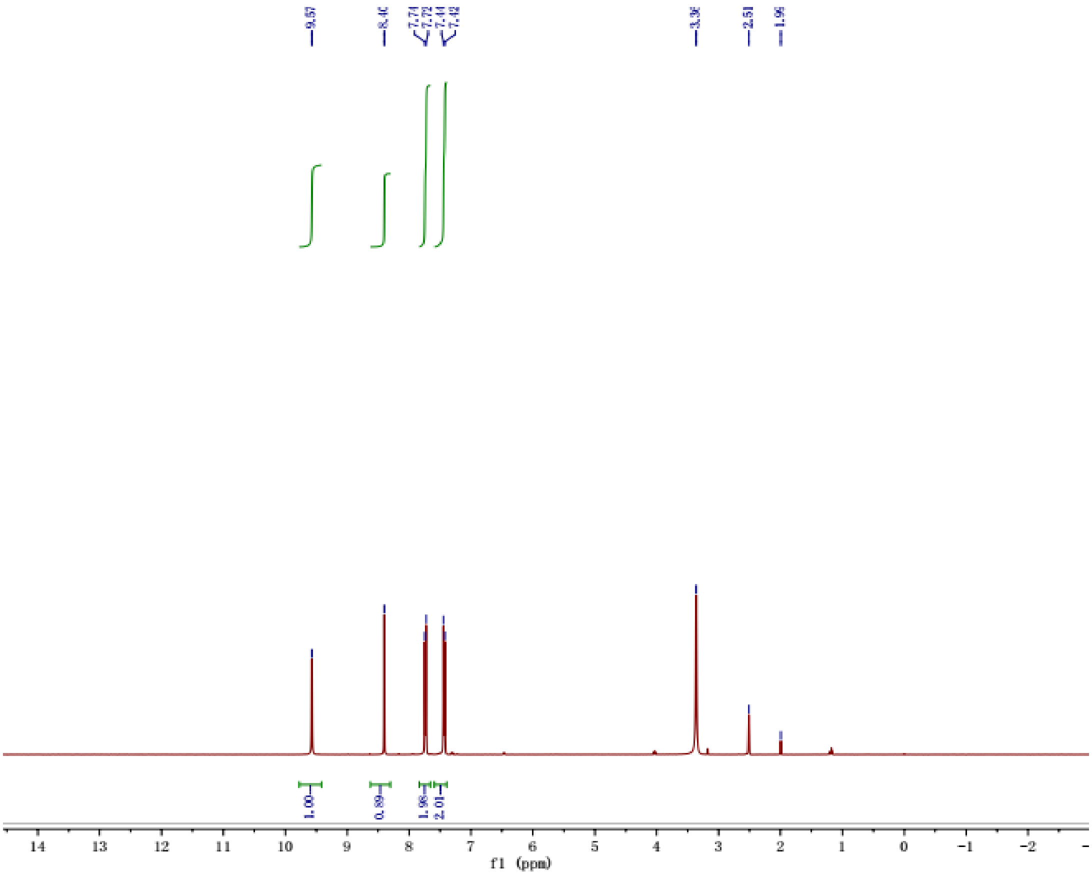


$^{1}$H-NMR spectrum of b17

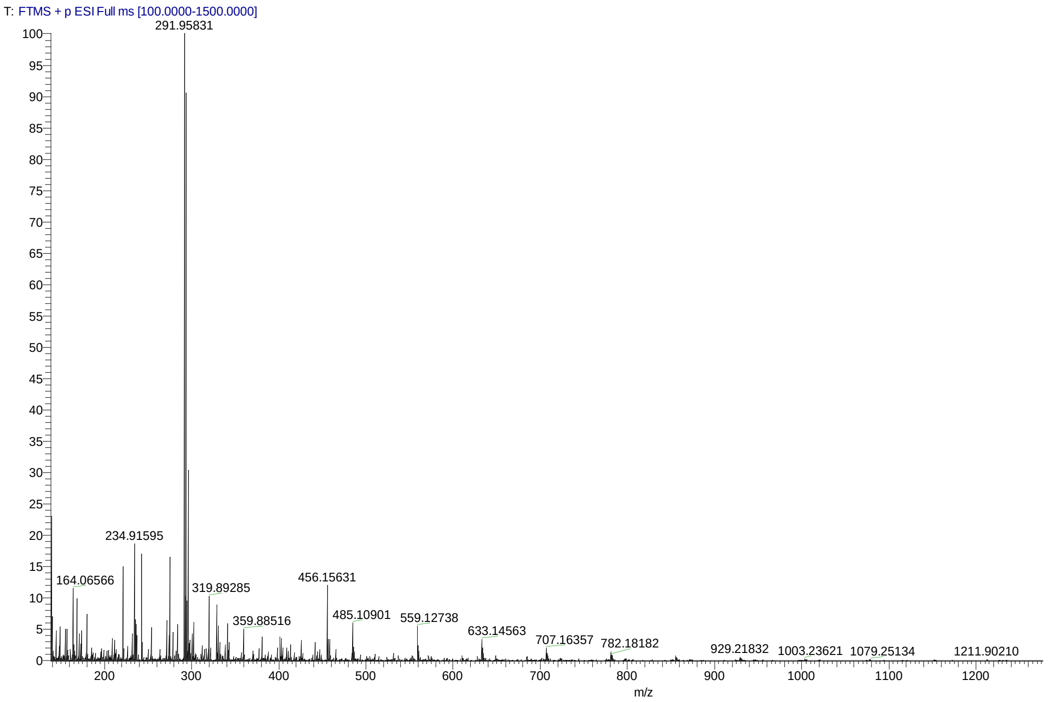


HRMS spectrum of b18

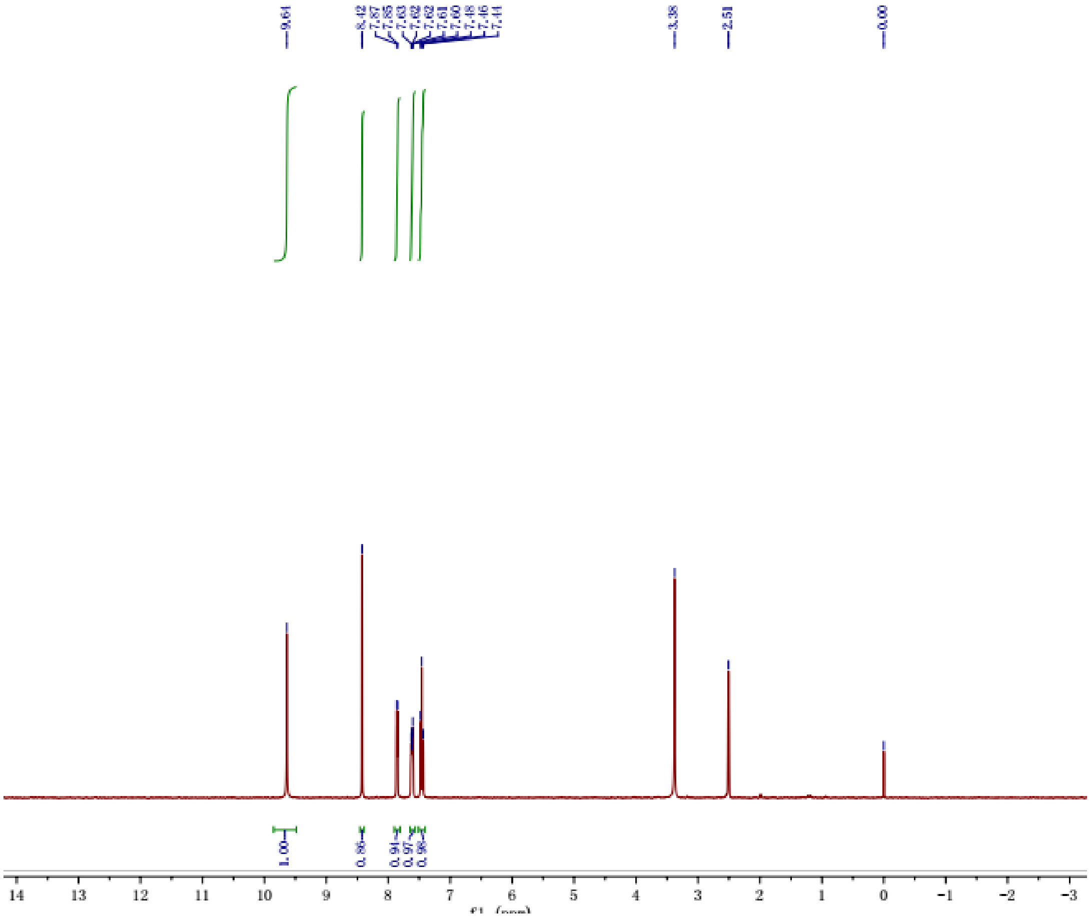


$^{1}$H-NMR spectrum of b18

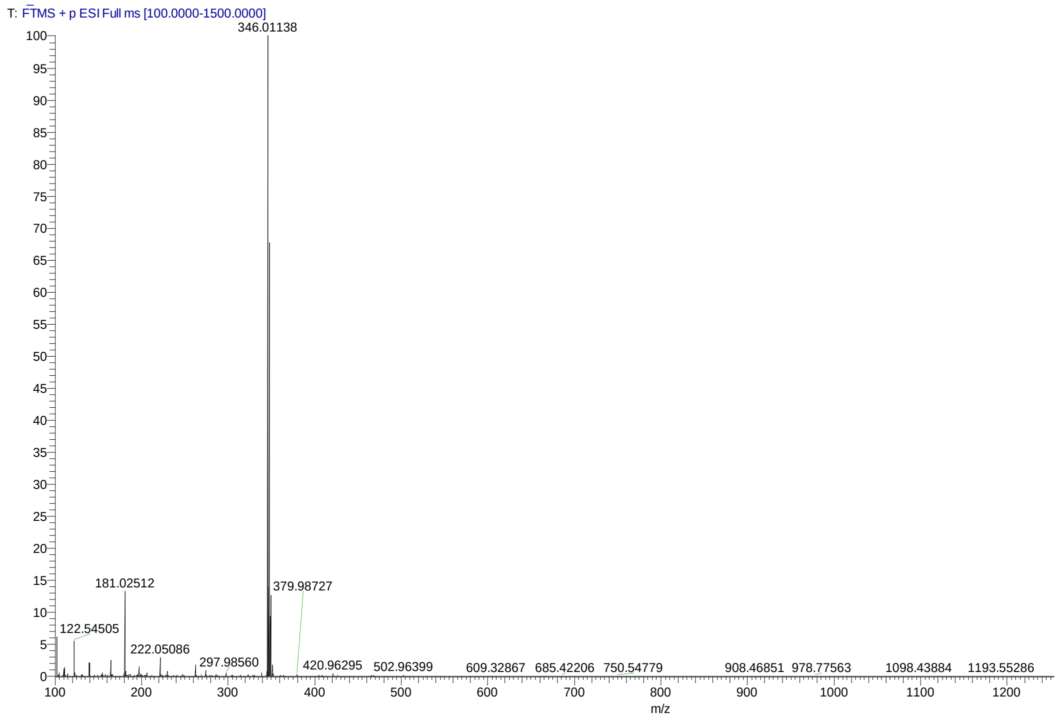


HRMS spectrum of b19

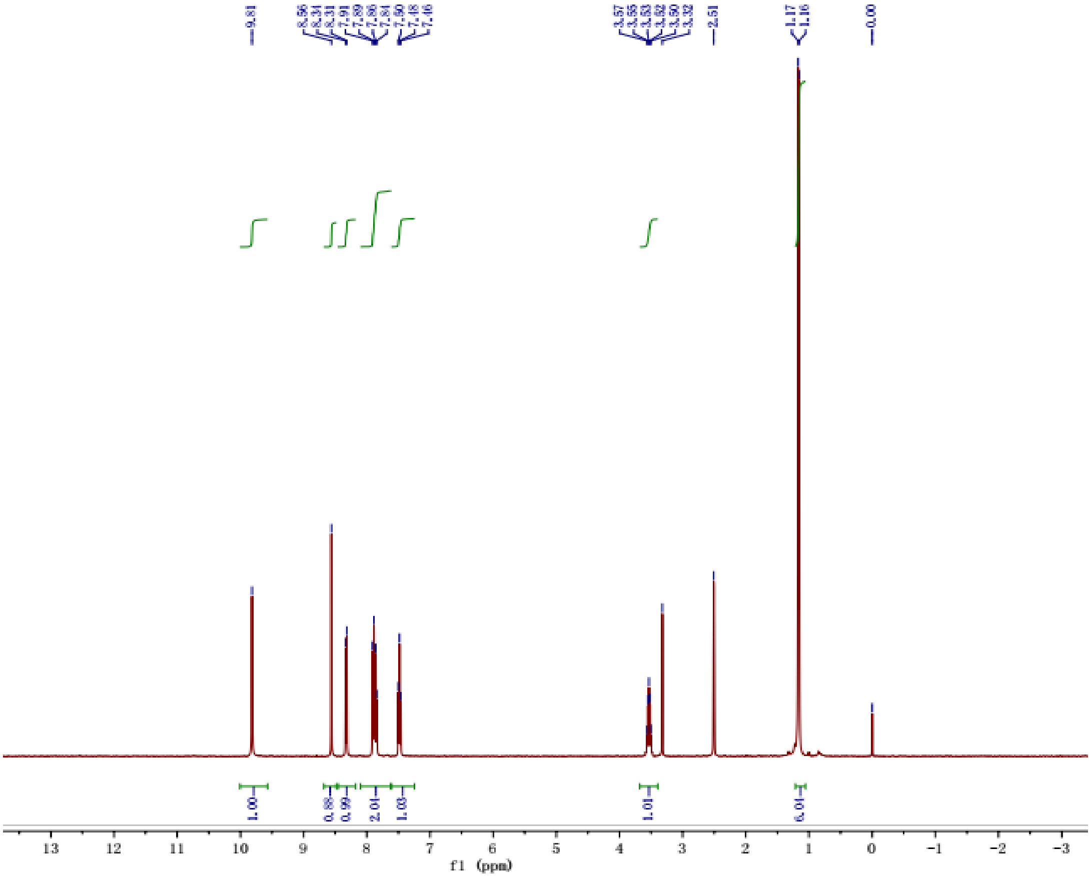


$^{1}$H-NMR spectrum of b19

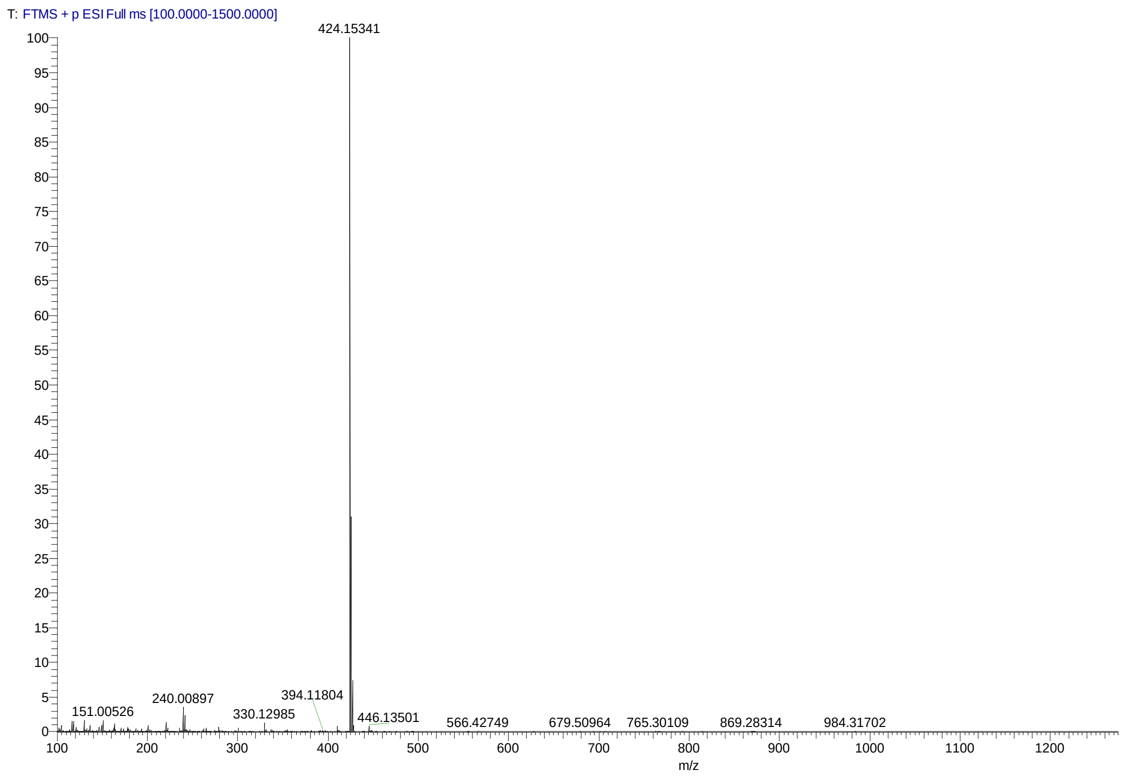


HRMS spectrum of c1

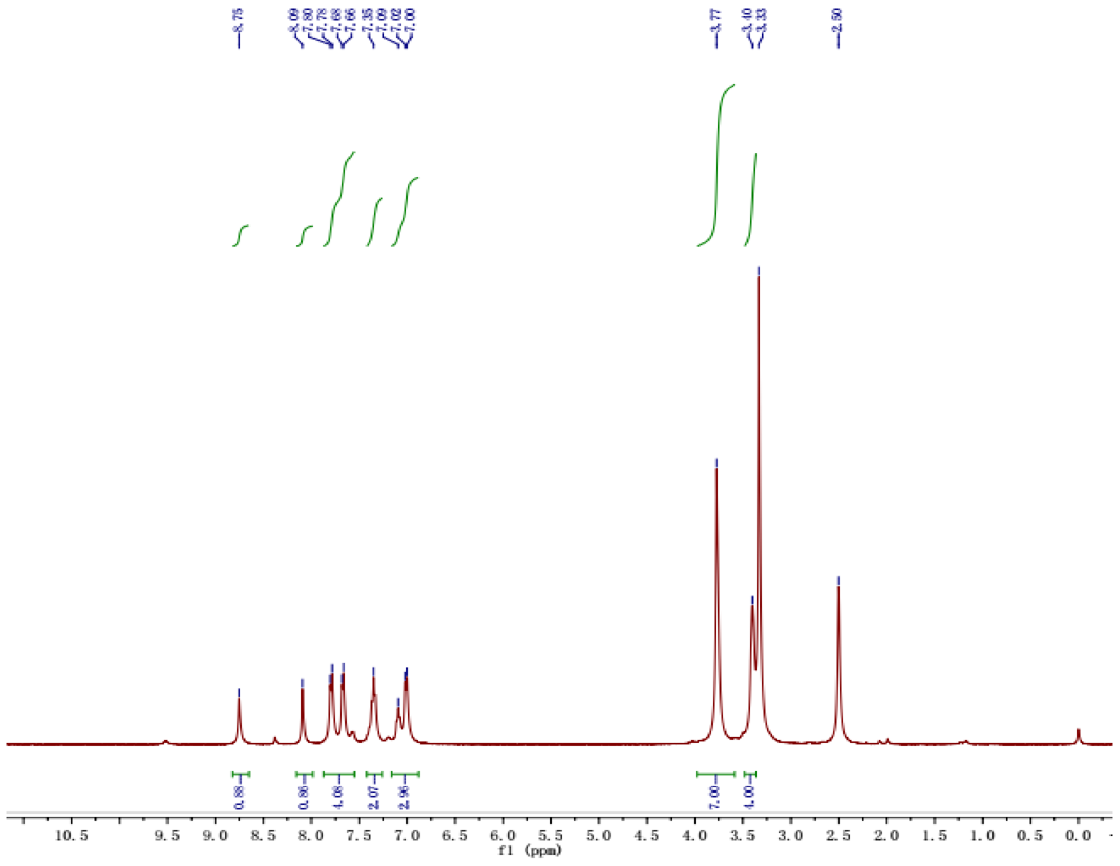


$^{1}$H-NMR spectrum of c1

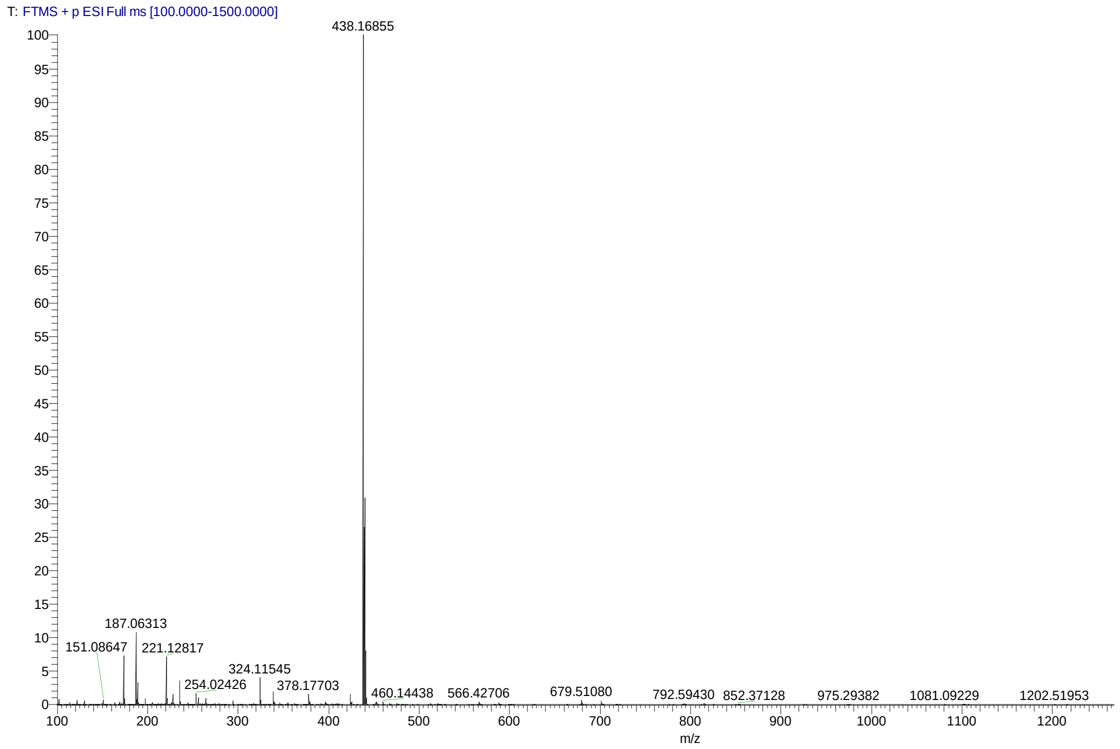


HRMS spectrum of c2

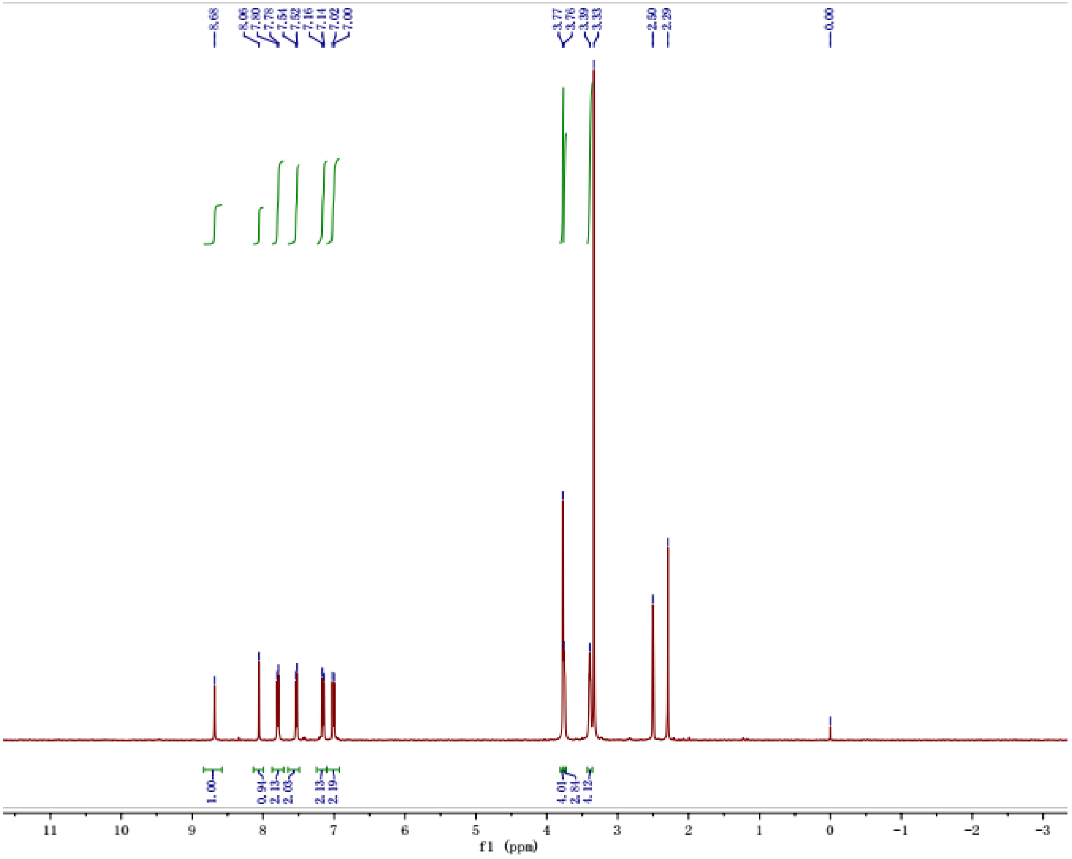


$^{1}$H-NMR spectrum of c2

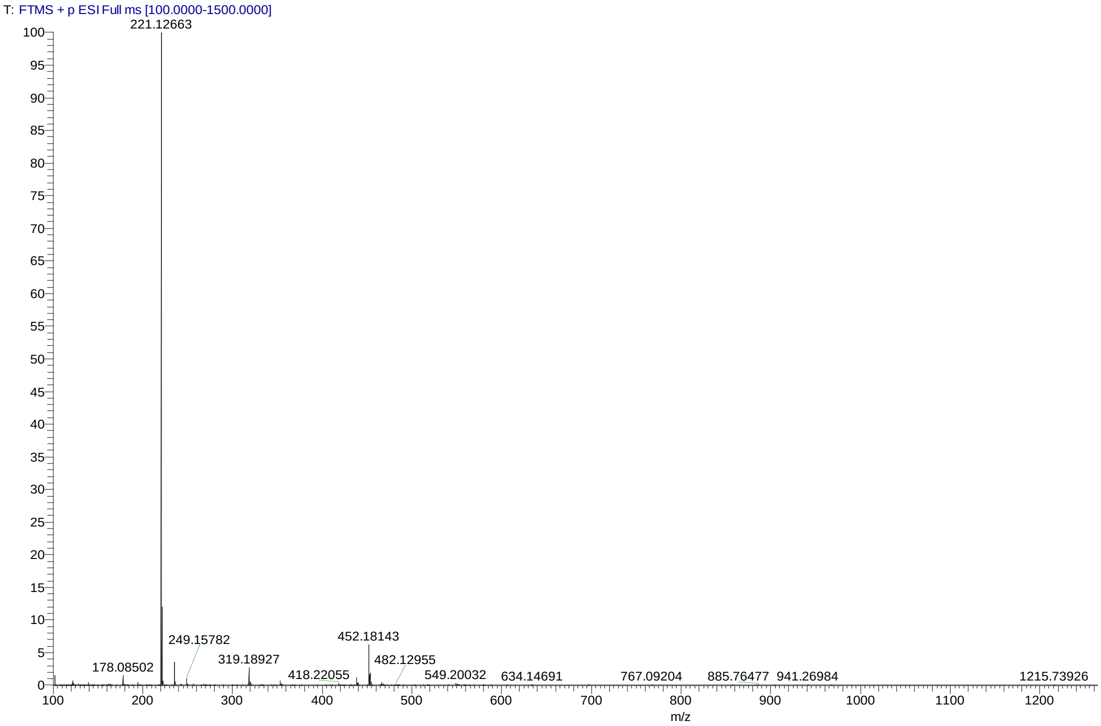


HRMS spectrum of c3

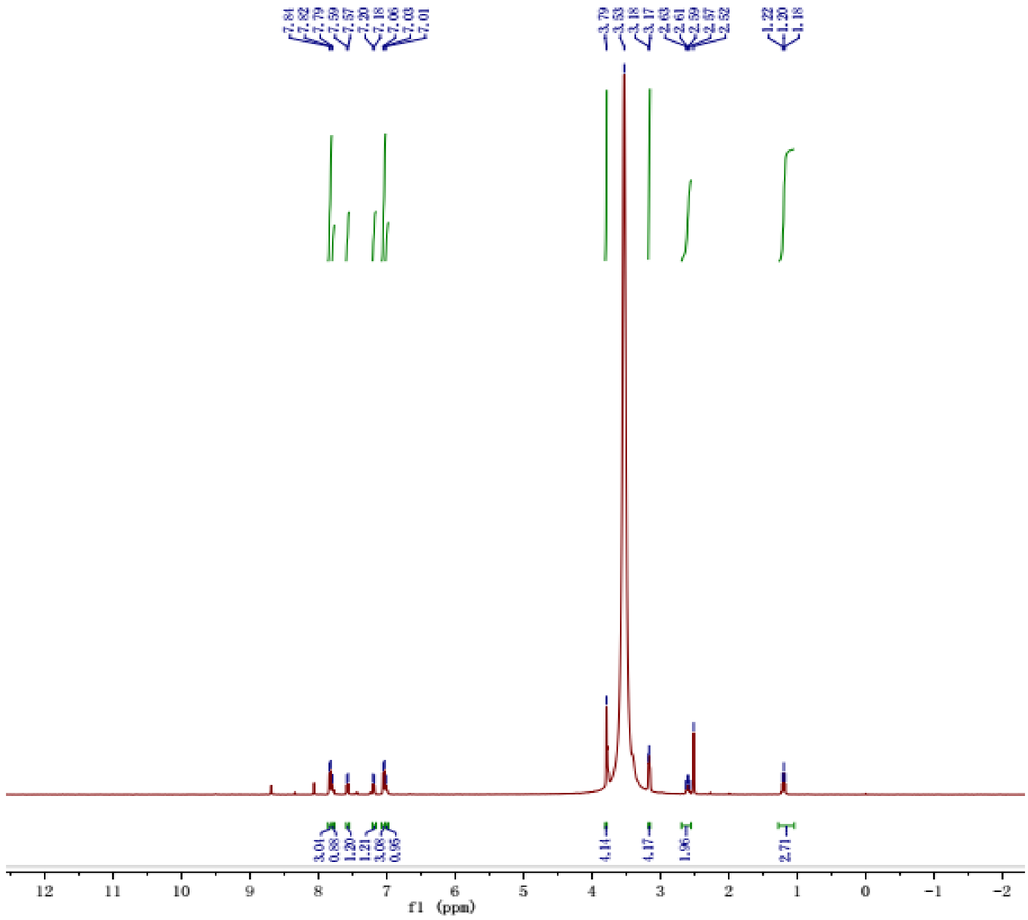


$^1$H-NMR spectrum of c3

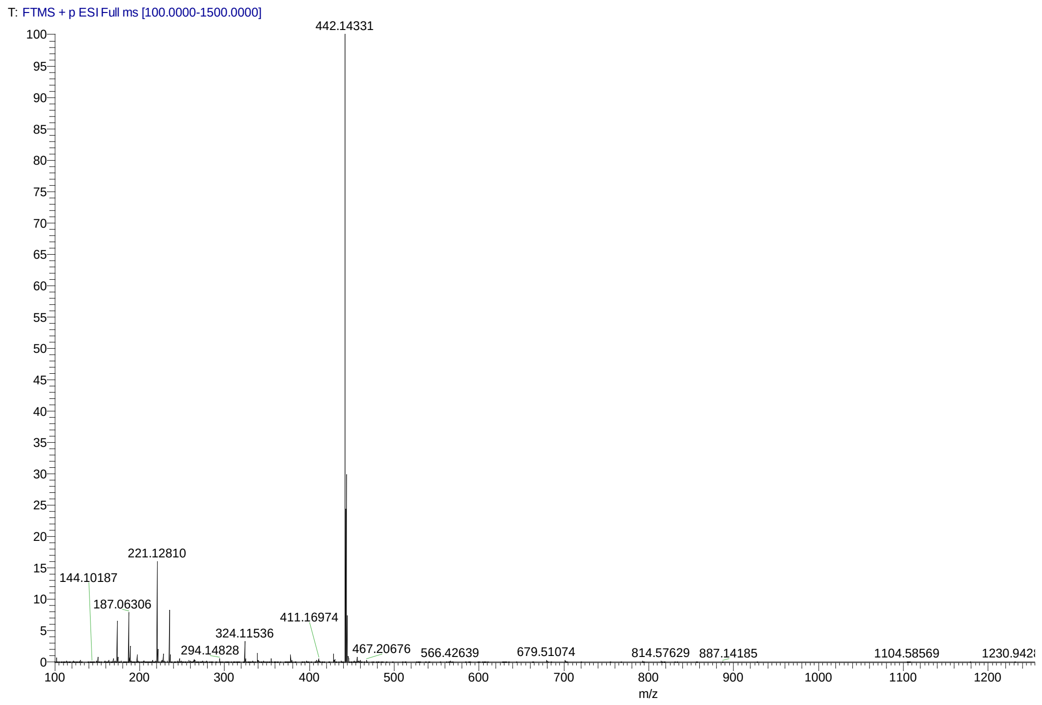


HRMS spectrum of c4

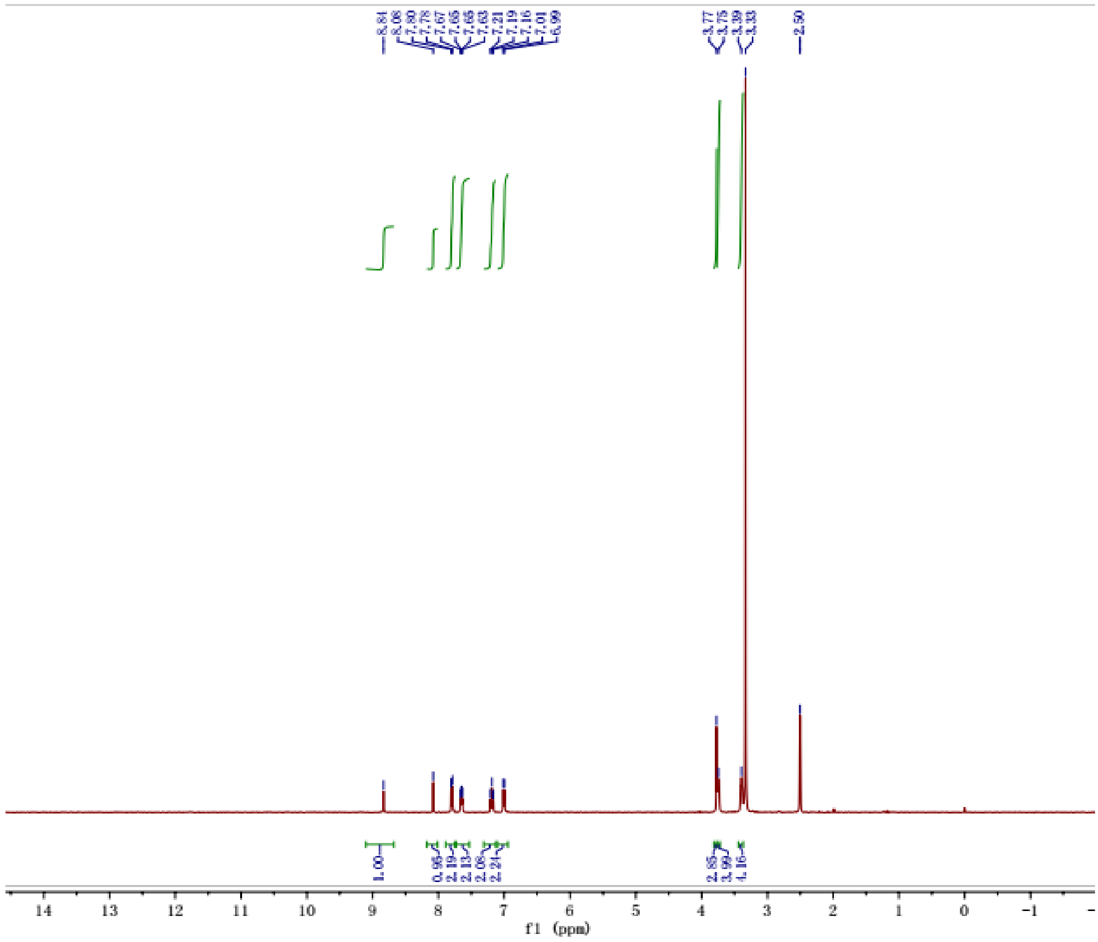


$^1$H-NMR spectrum of c4

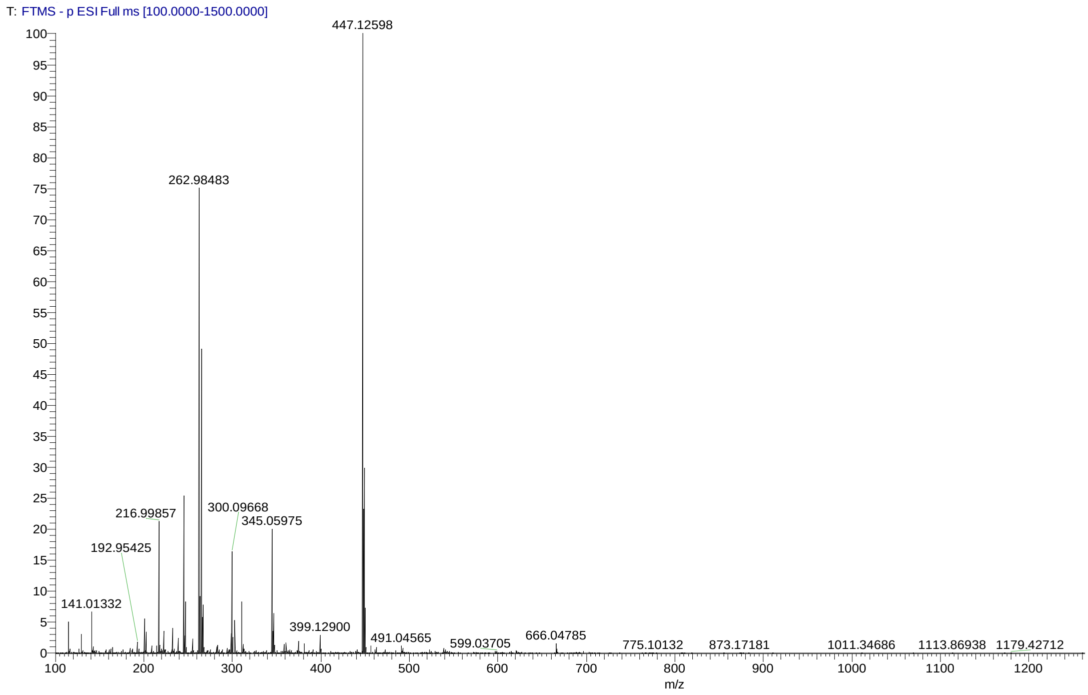


HRMS spectrum of c5

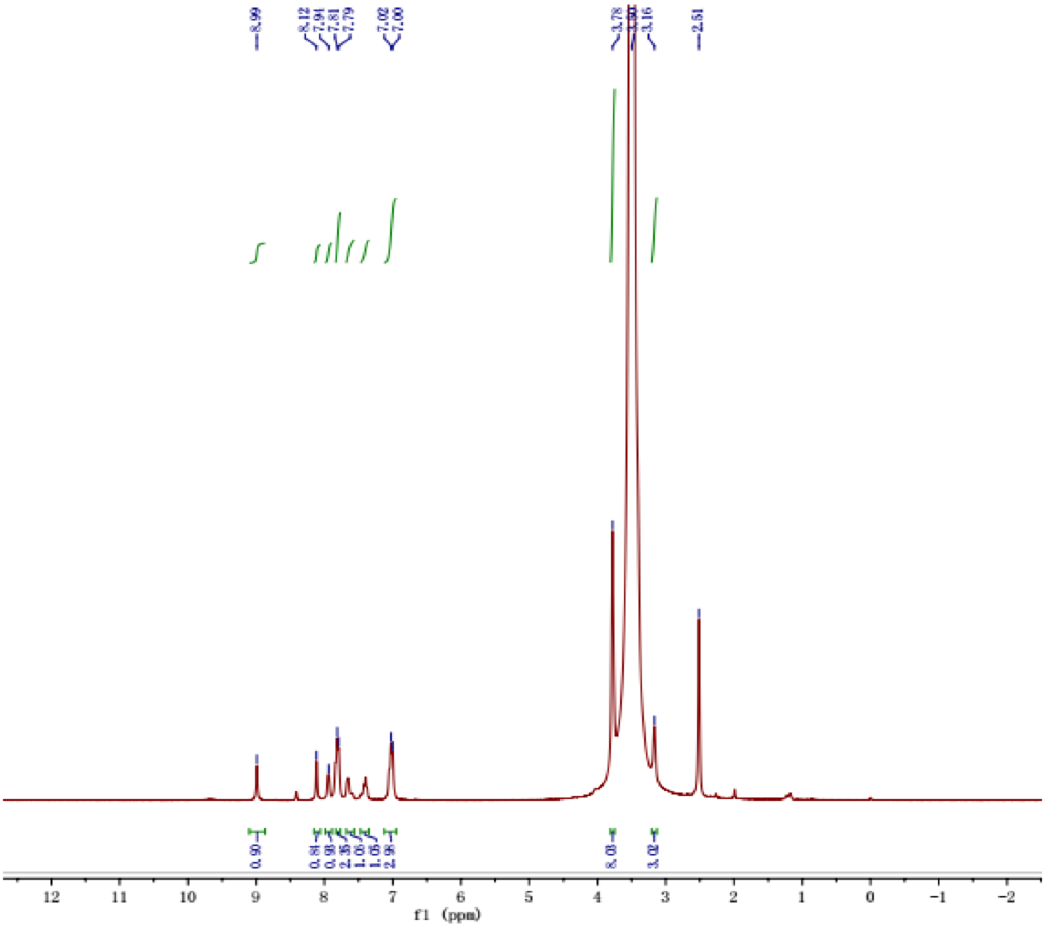


$^1$H-NMR spectrum of c5

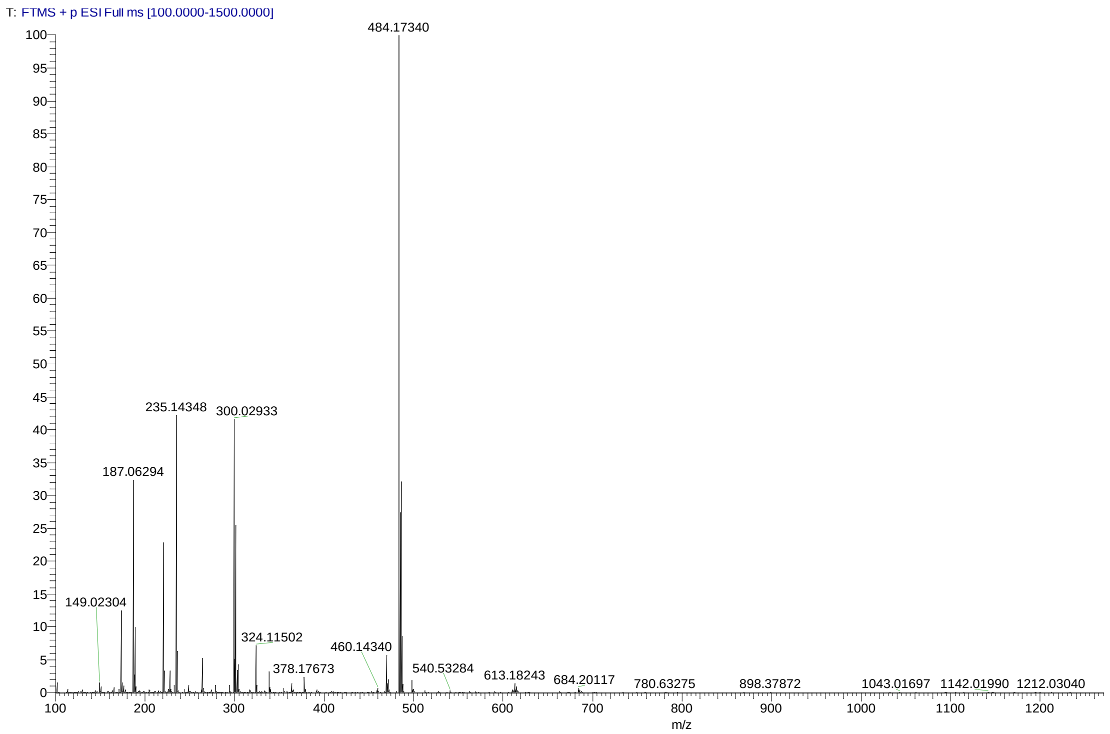


HRMS spectrum of c6

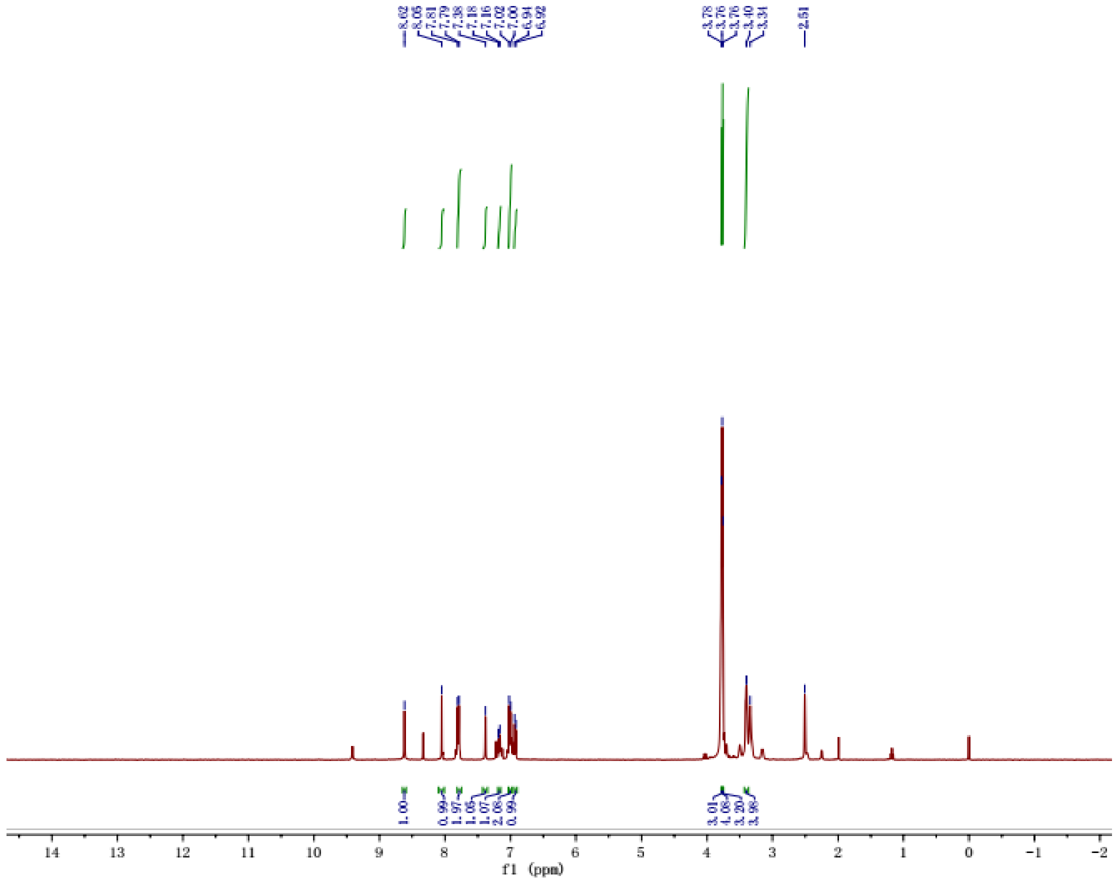


$^1$H-NMR spectrum of c6

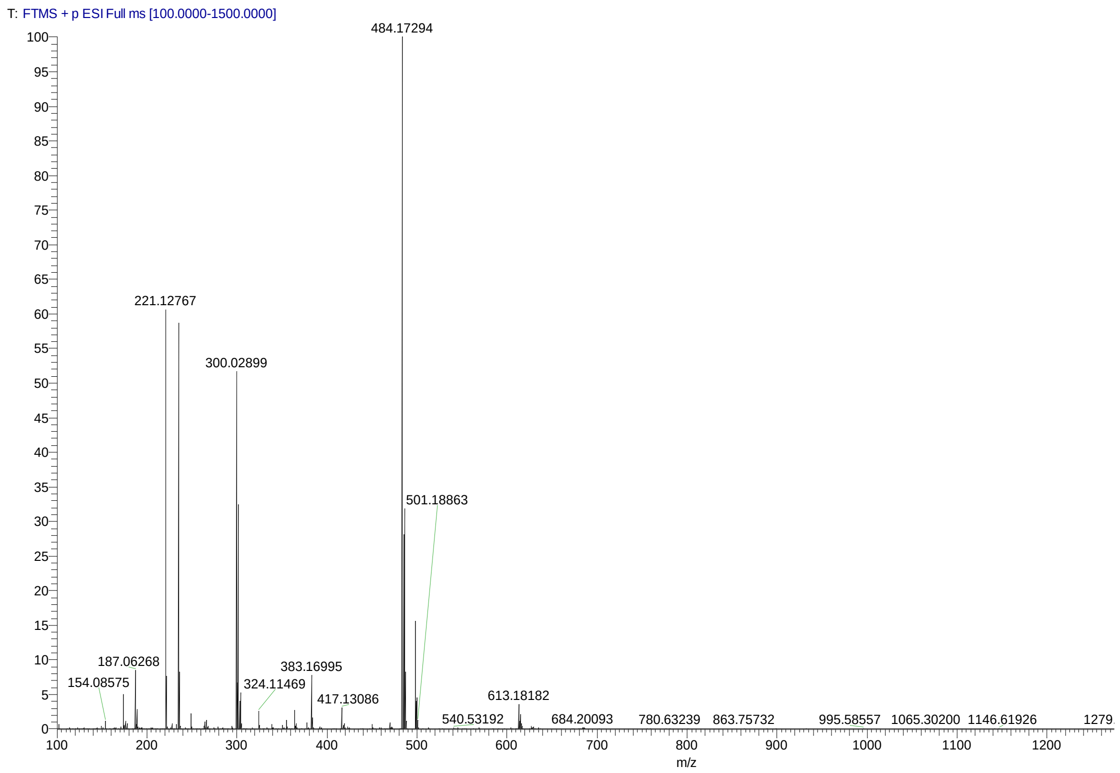


HRMS spectrum of c7

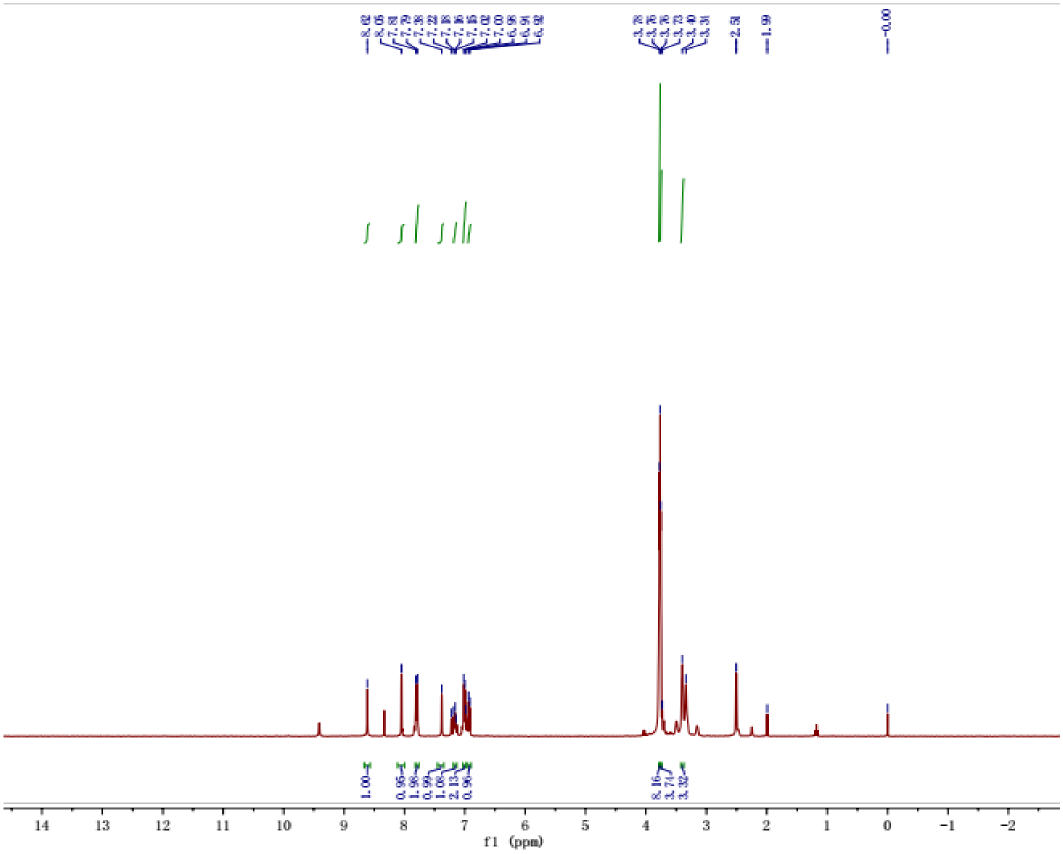


$^{1}$H-NMR spectrum of c7

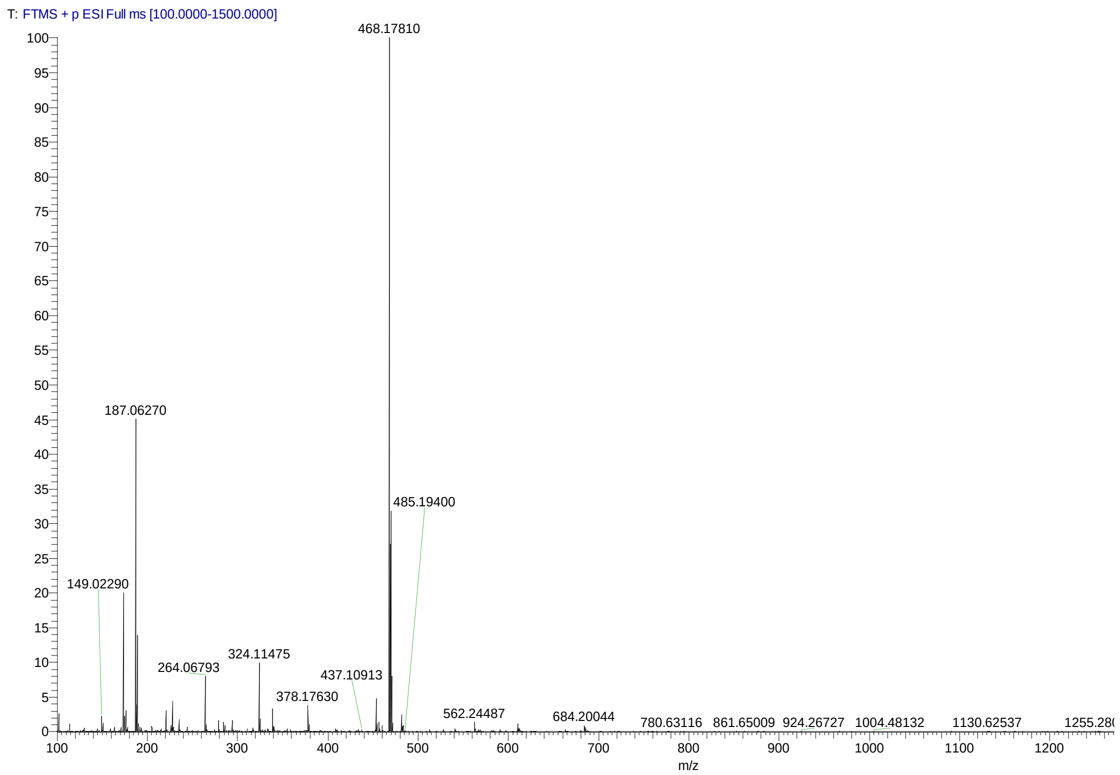


HRMS $^{1}$H-NMR spectrum of c8

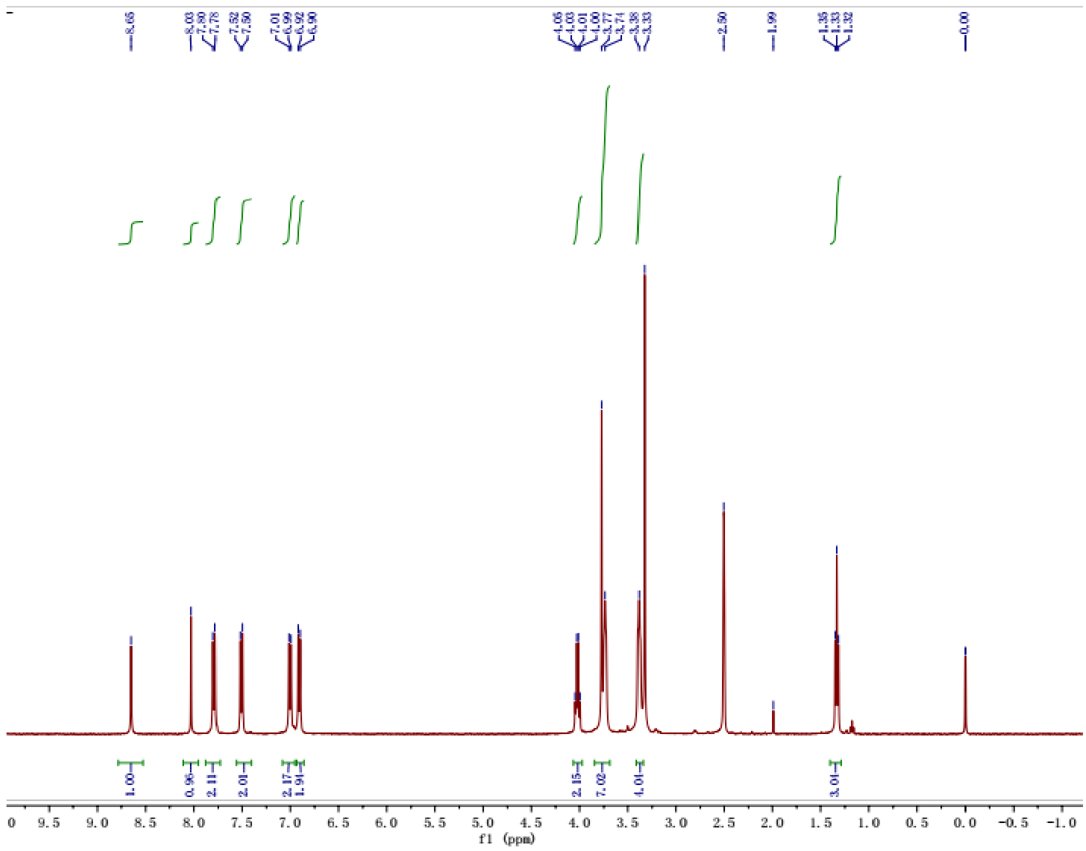


$^{1}$H-NMR spectrum of c8

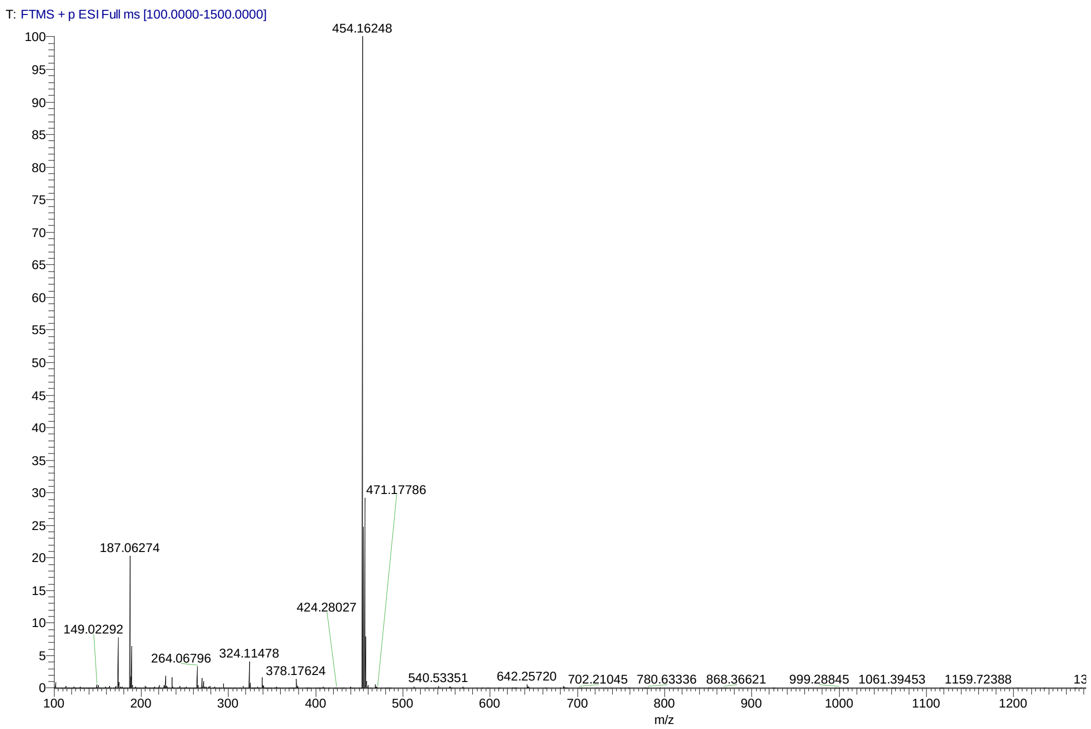


HRMS spectrum of c9

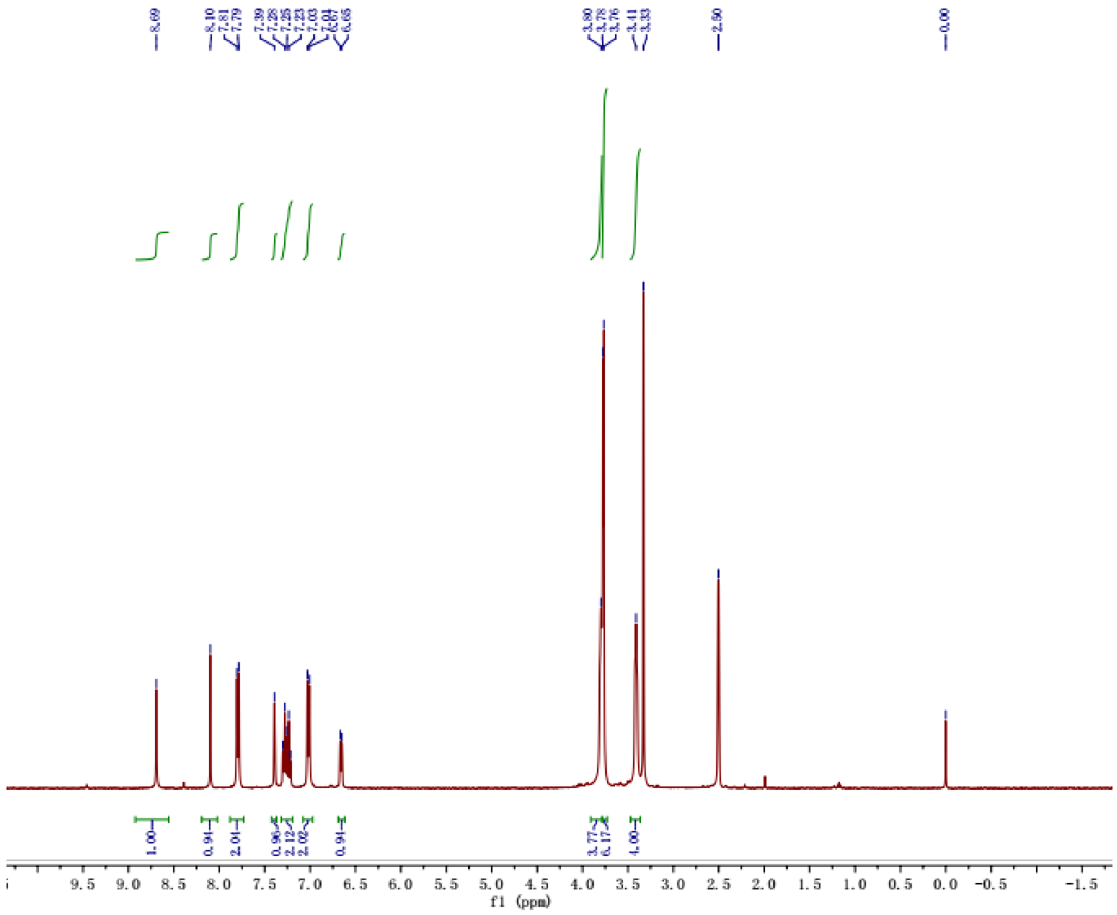


$^1$H-NMR spectrum of c9

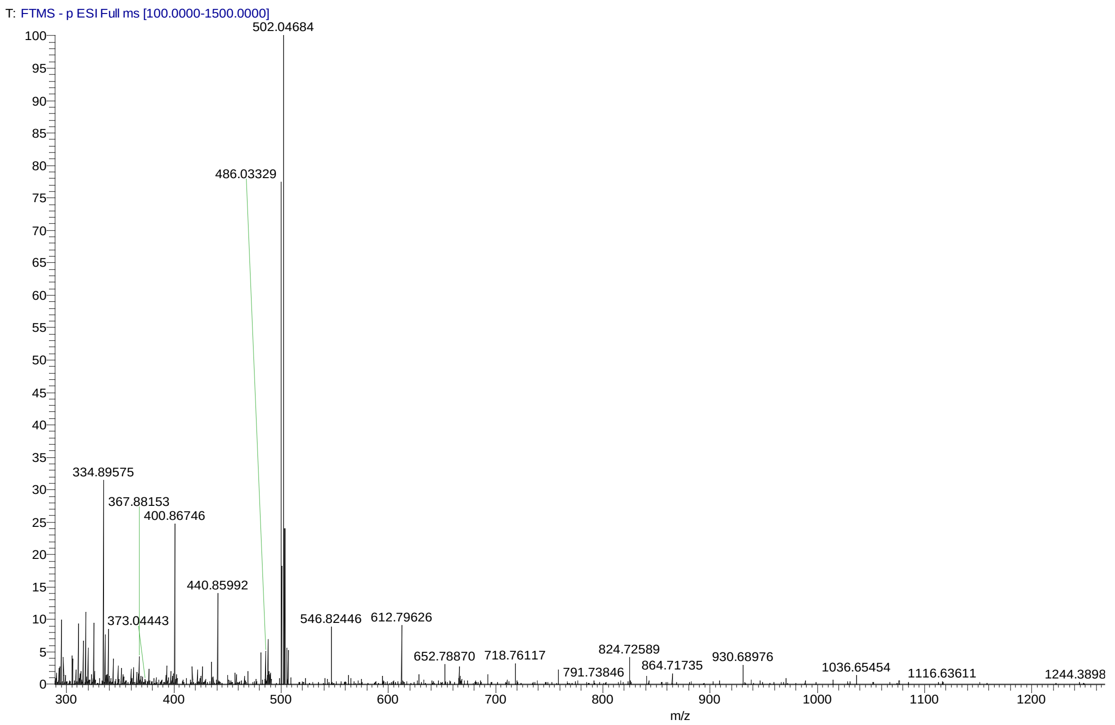


HRMS spectrum of c10

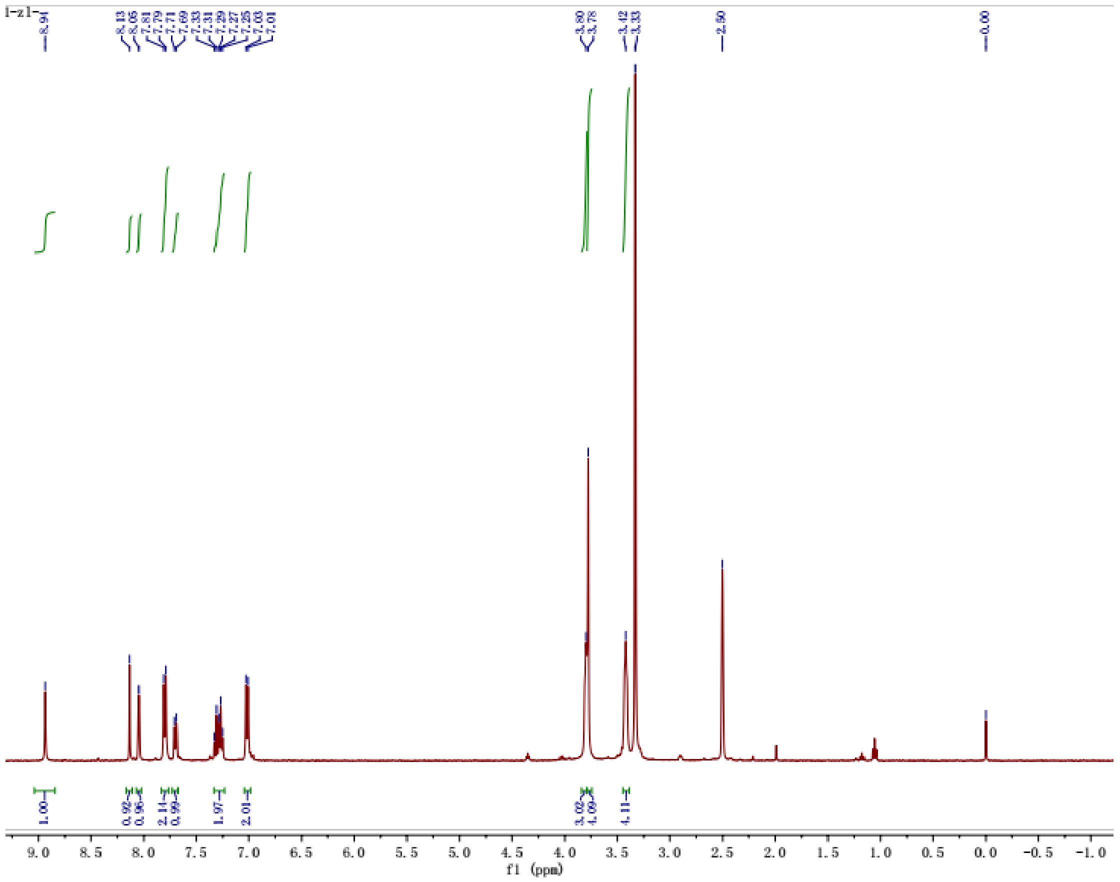


$^{1}$H-NMR spectrum of c10

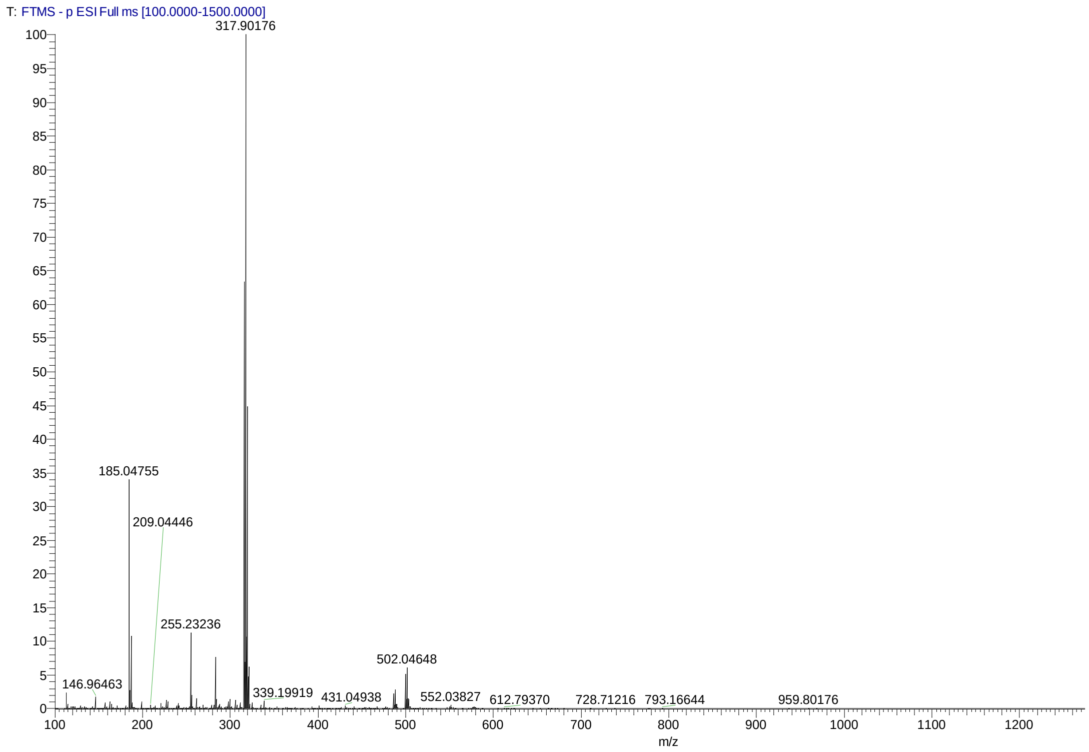


HRMS spectrum of c11

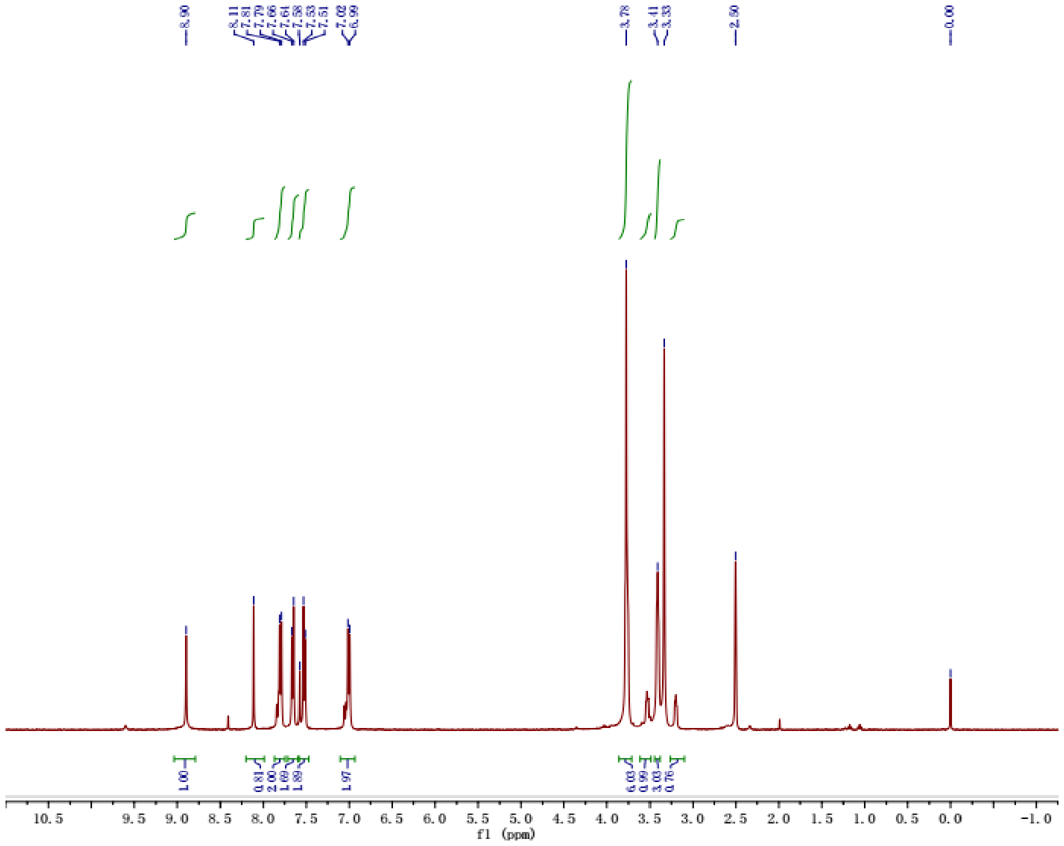


$^{1}$H-NMR spectrum of c11

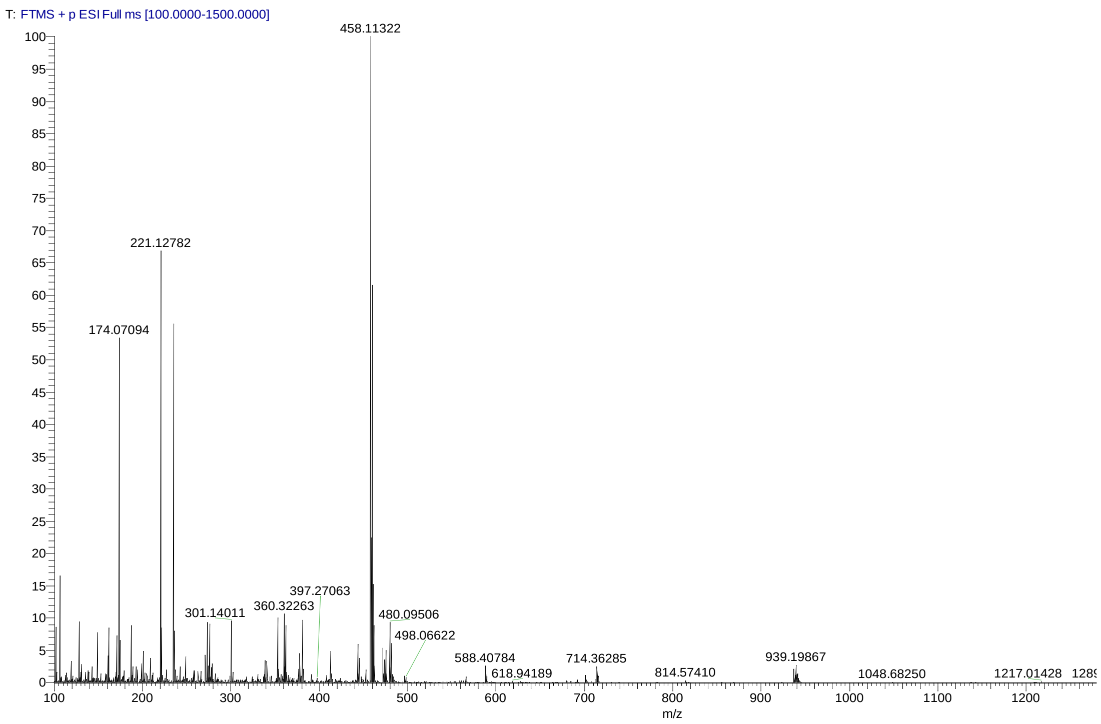


HRMS spectrum of c12

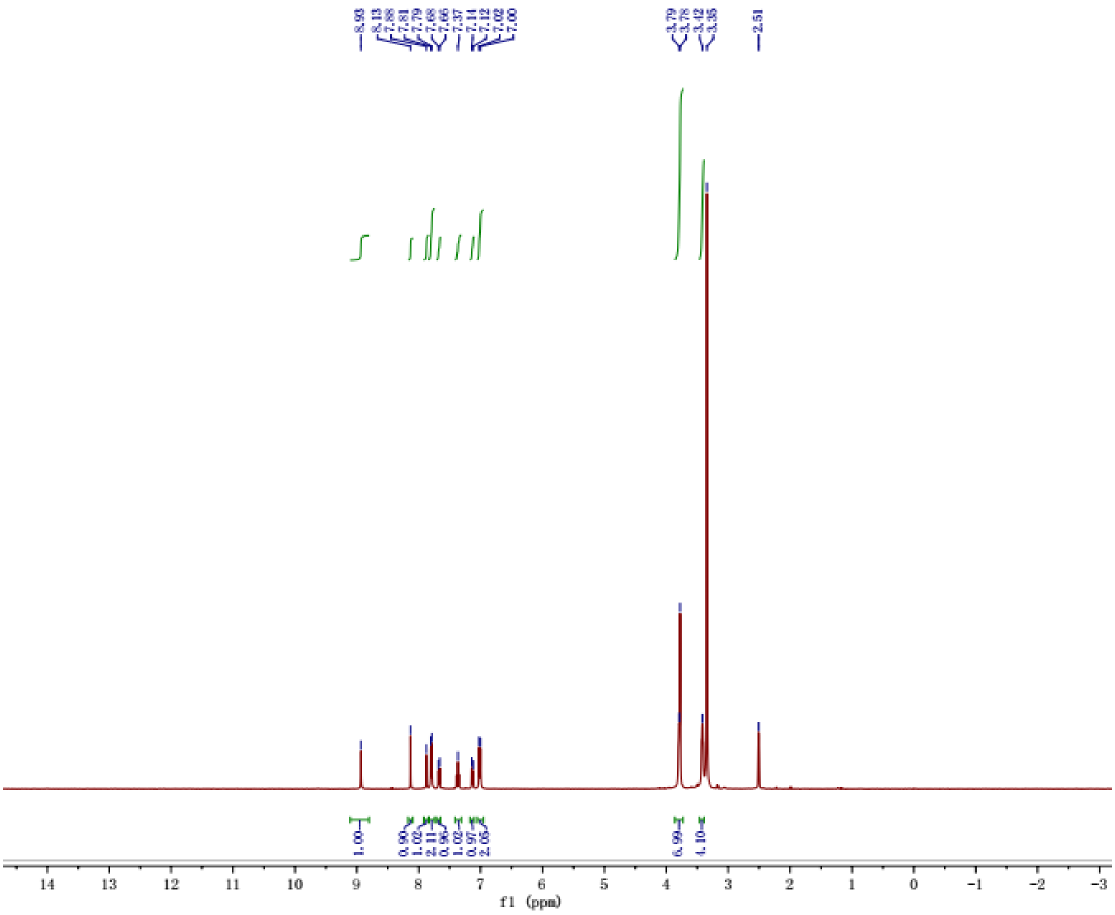


$^1$H-NMR spectrum of c12

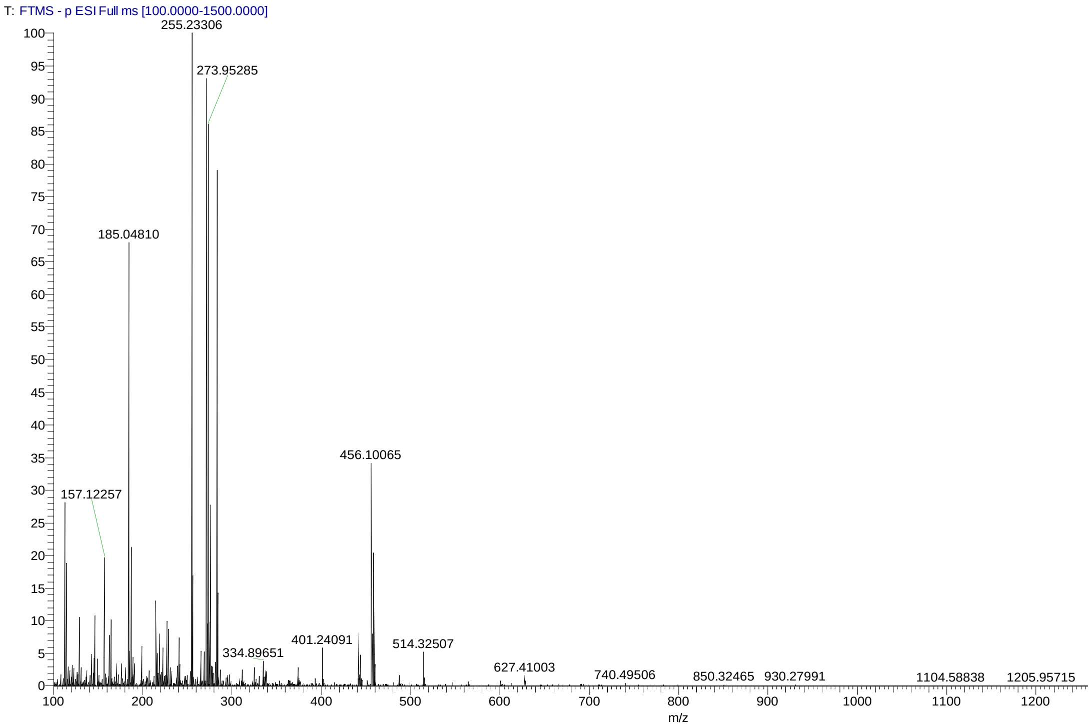


HRMS spectrum of c13

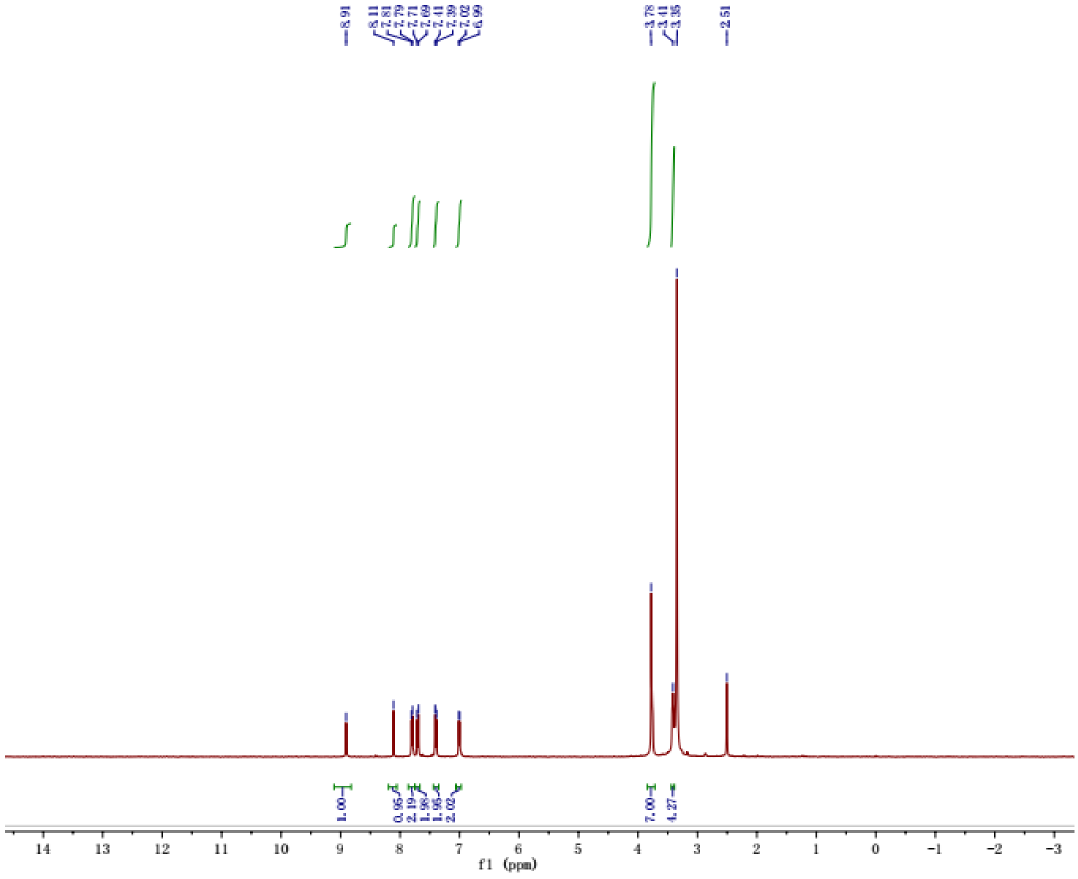


$^{1}$H-NMR spectrum of c13

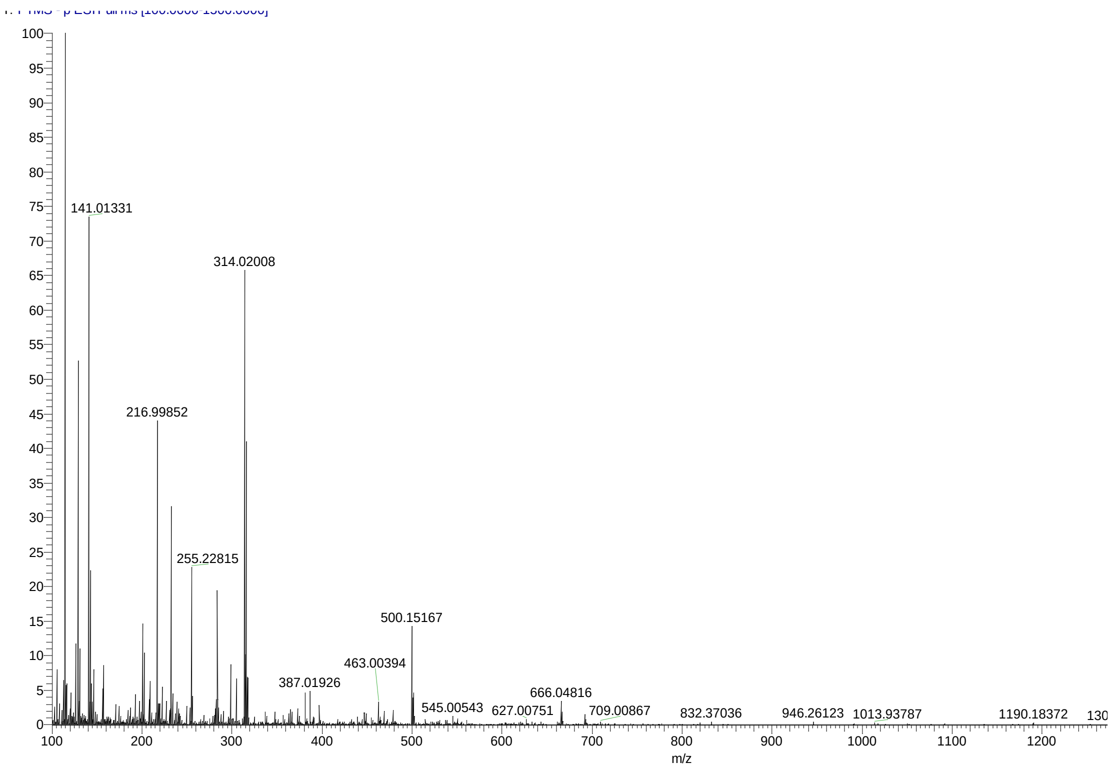


HRMS spectrum of c14

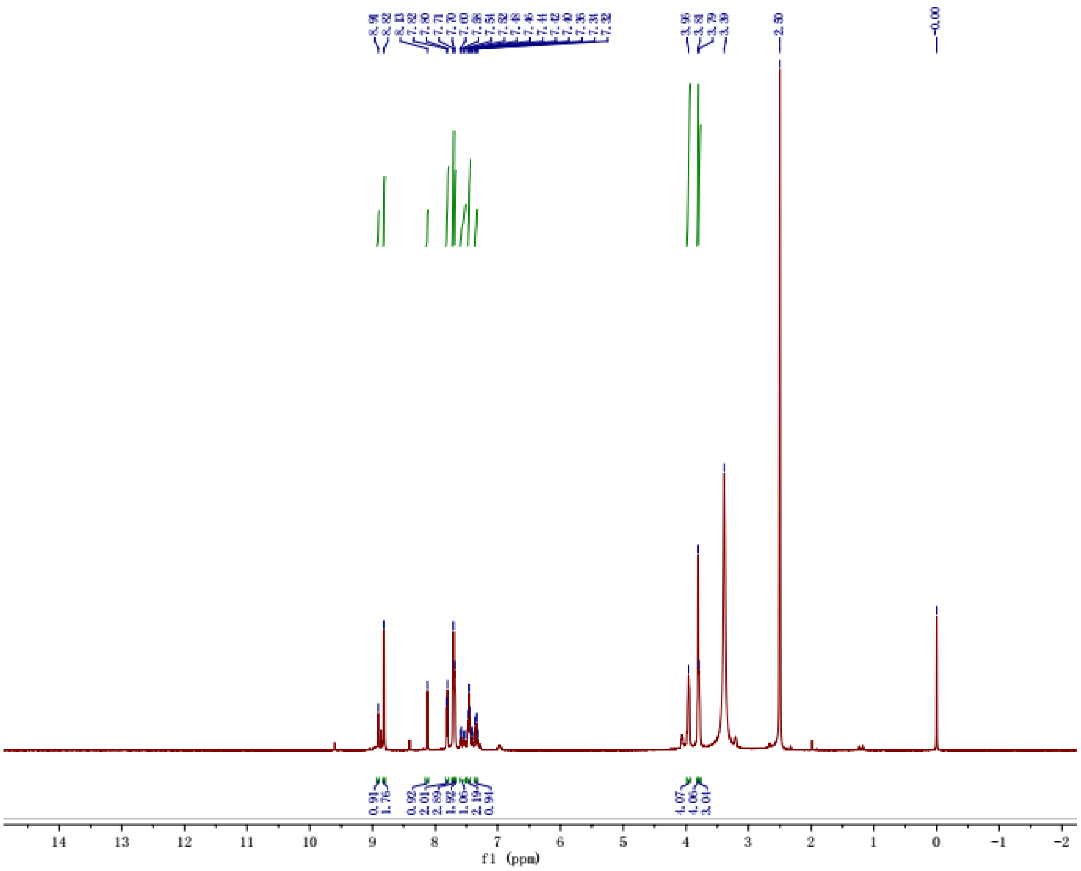


$^{1}$H-NMR spectrum of c14

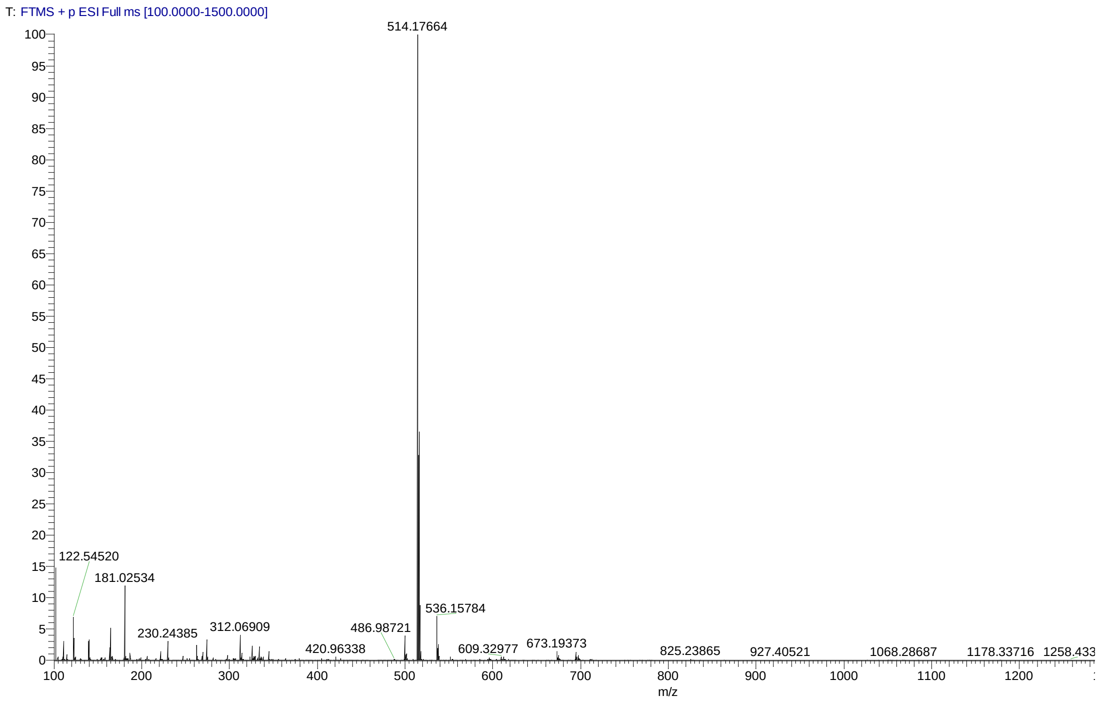


HRMS spectrum of c15

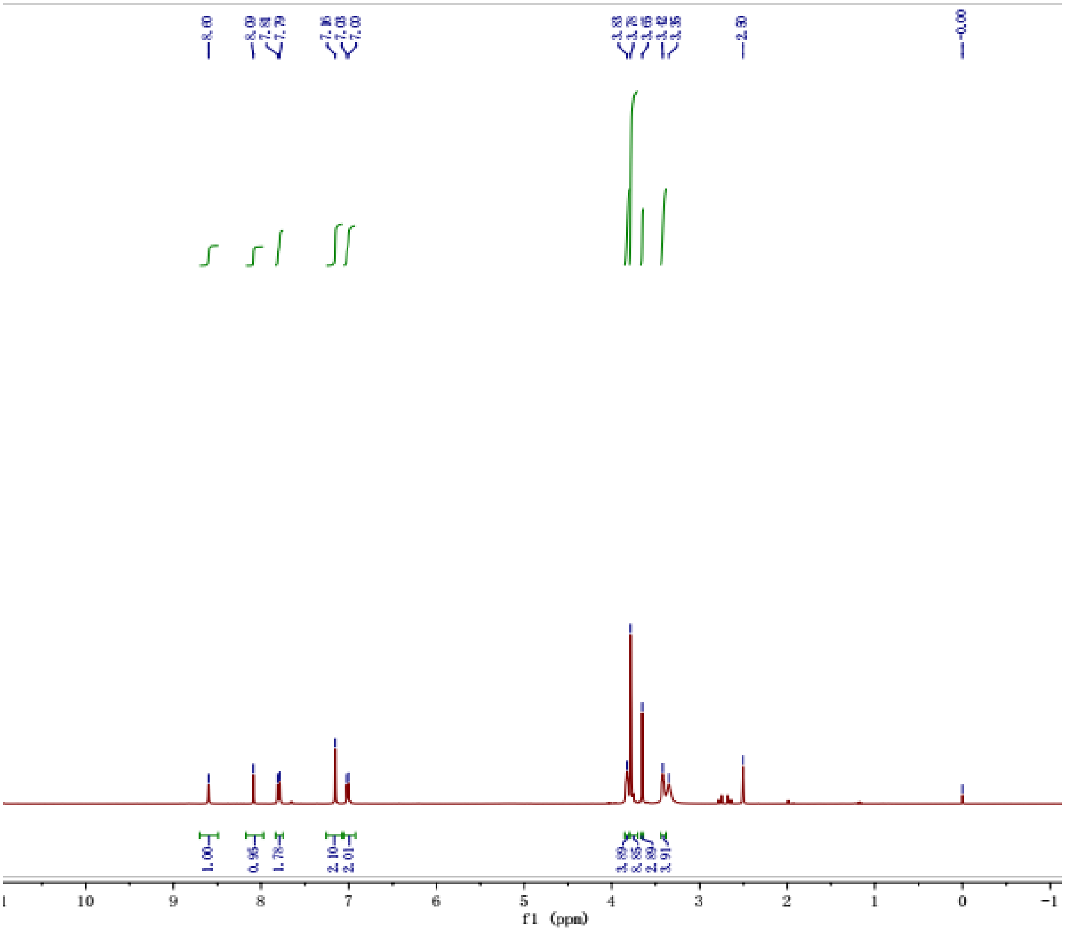


$^{1}$H-NMR spectrum of c15

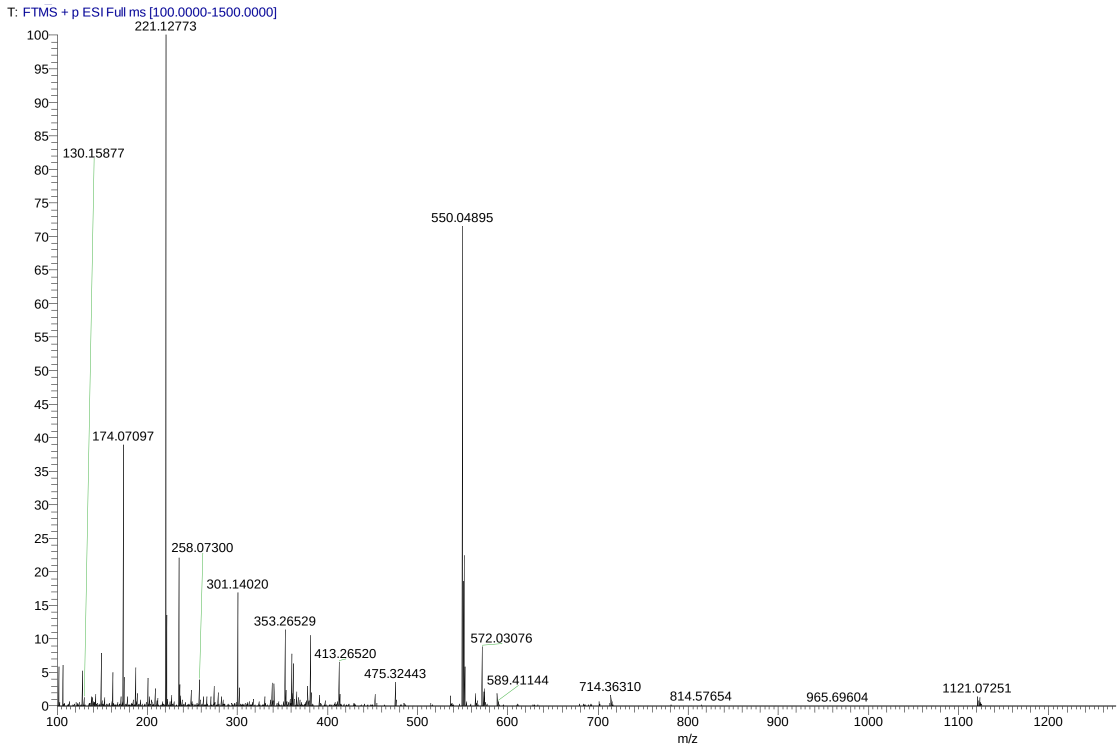


HRMS spectrum of c16

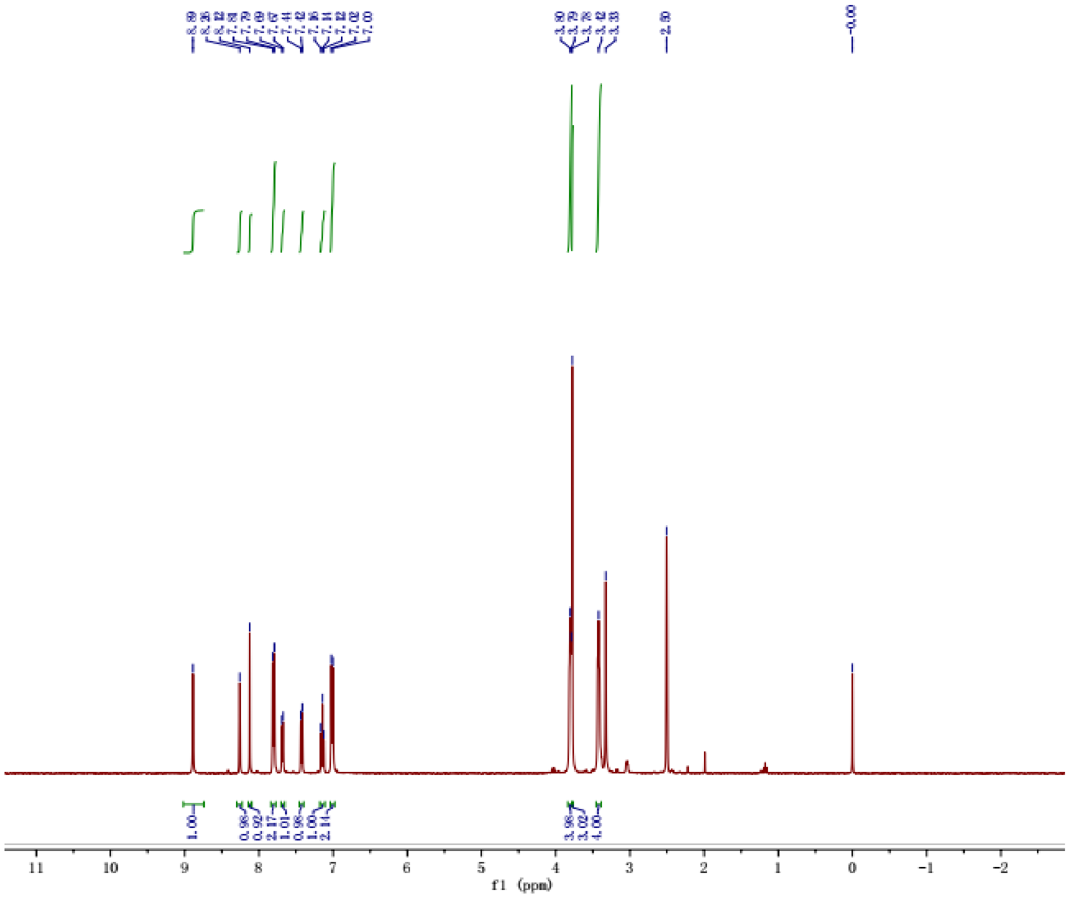


$^{1}$H-NMR spectrum of c16

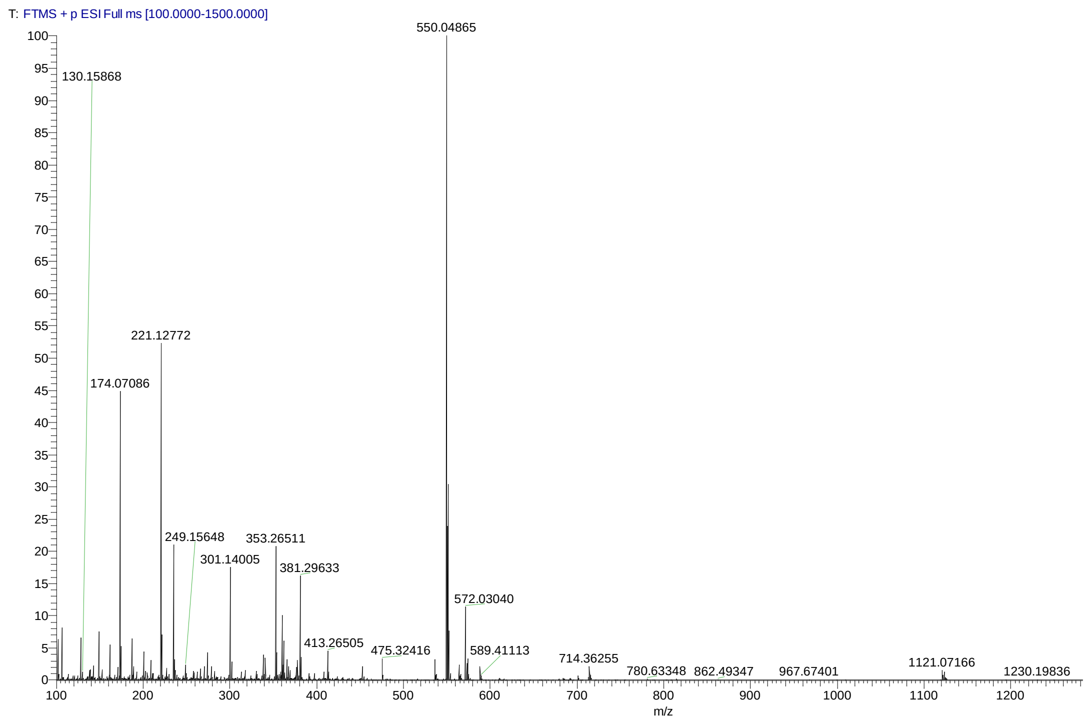


HRMS spectrum of c17

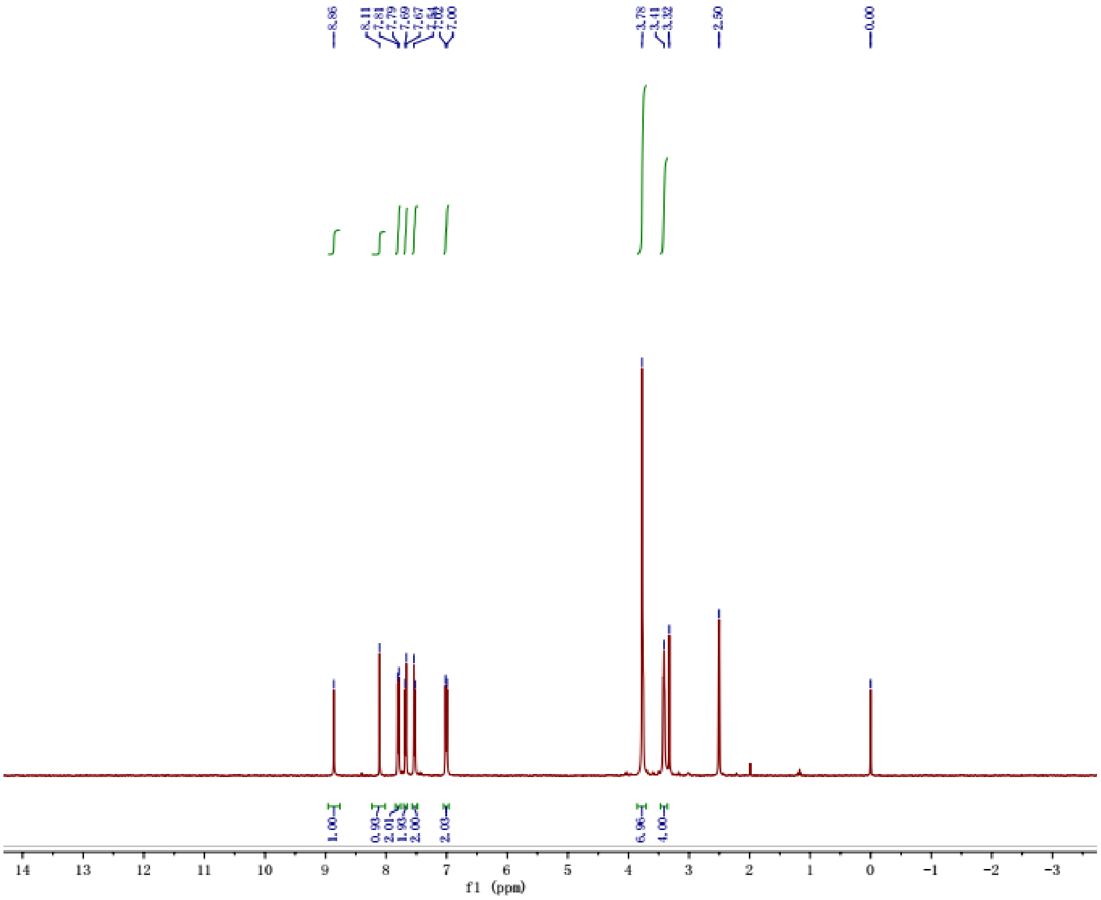


$^{1}$H-NMR spectrum of c17

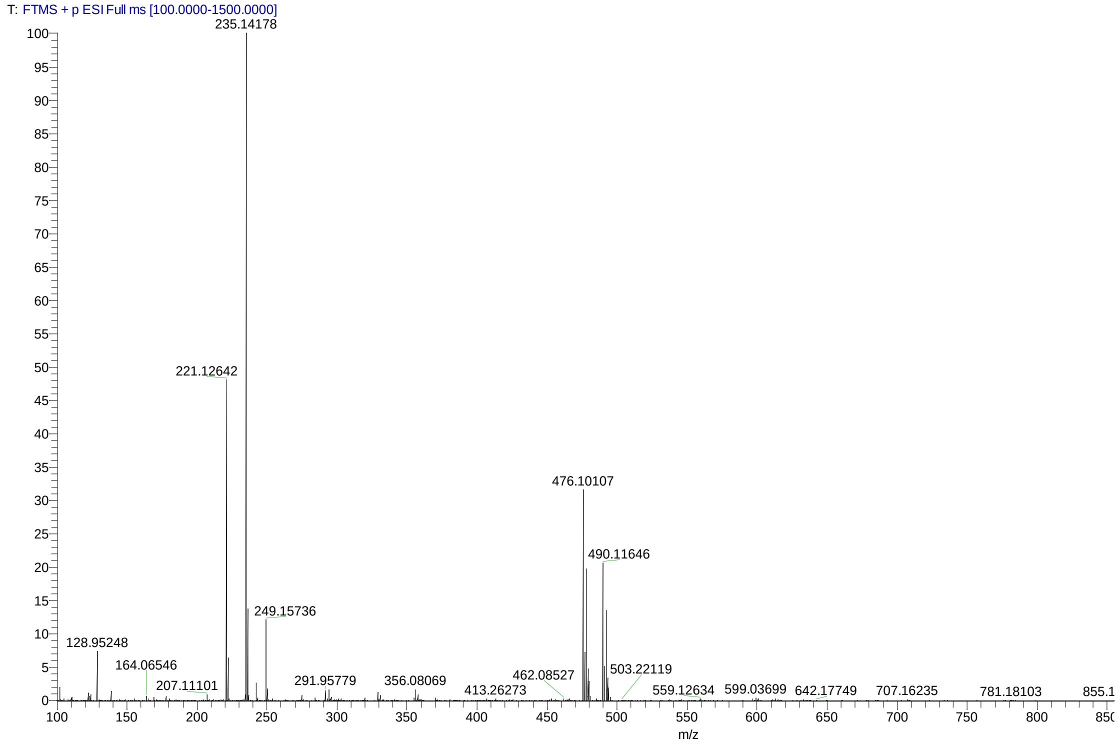


HRMS spectrum of c18

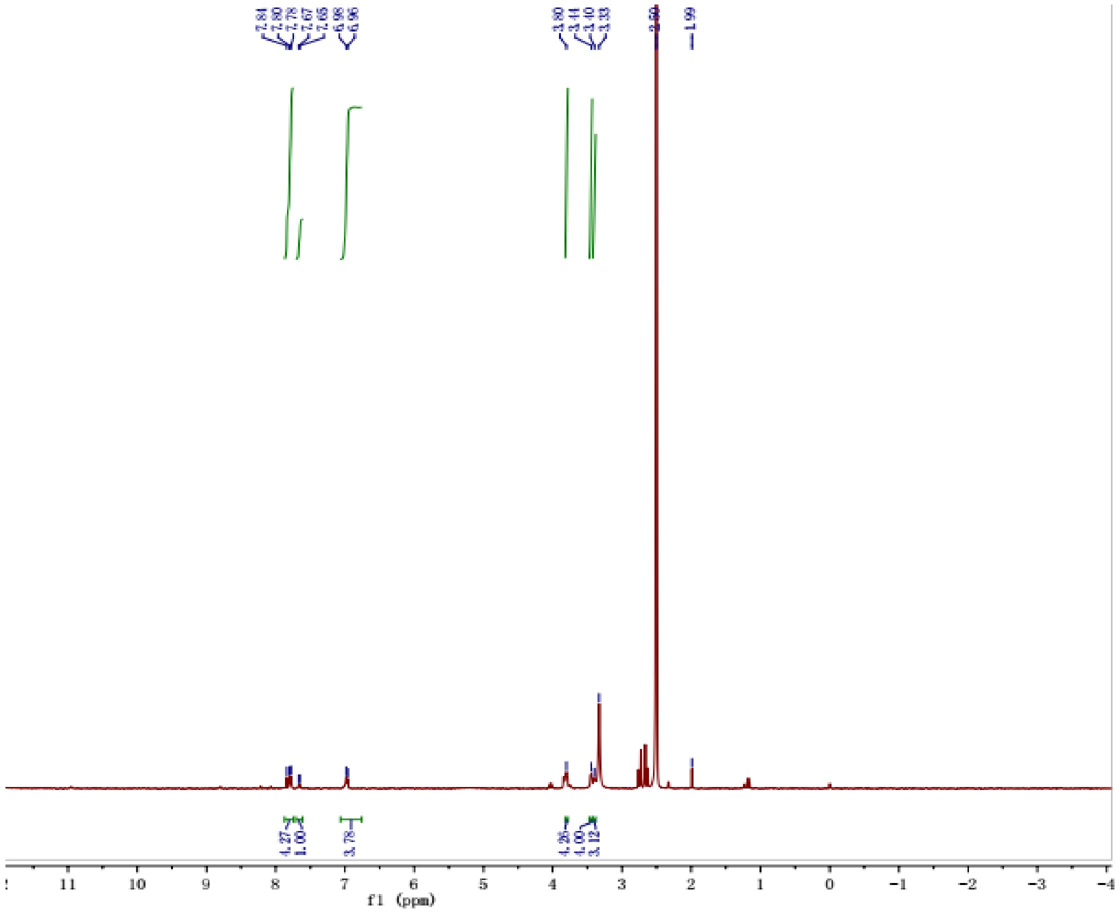


$^1$H-NMR spectrum of c18

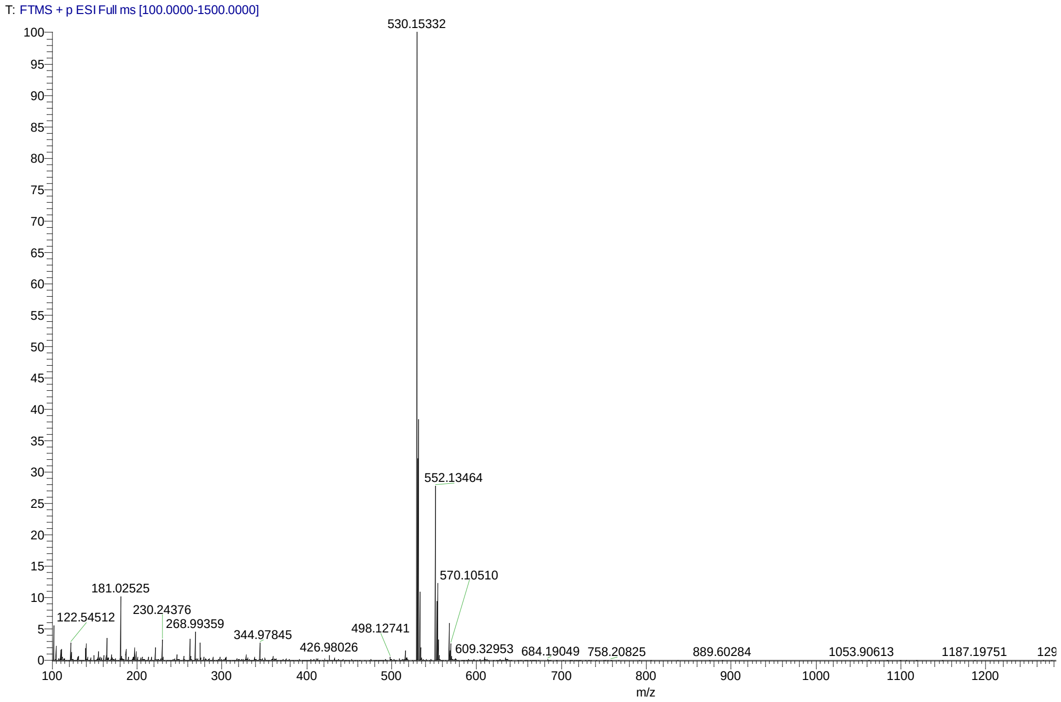


HRMS spectrum of c19

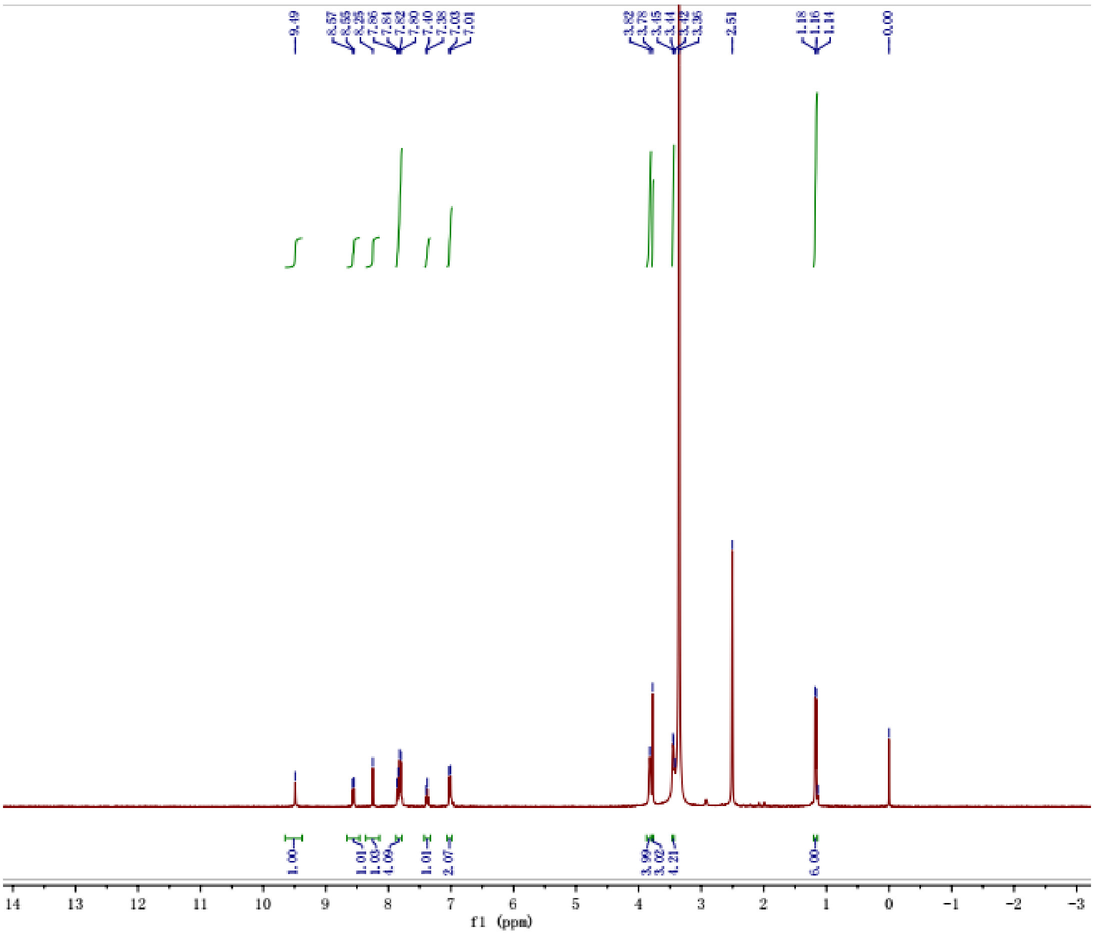


$^{1}$H-NMR spectrum of c19

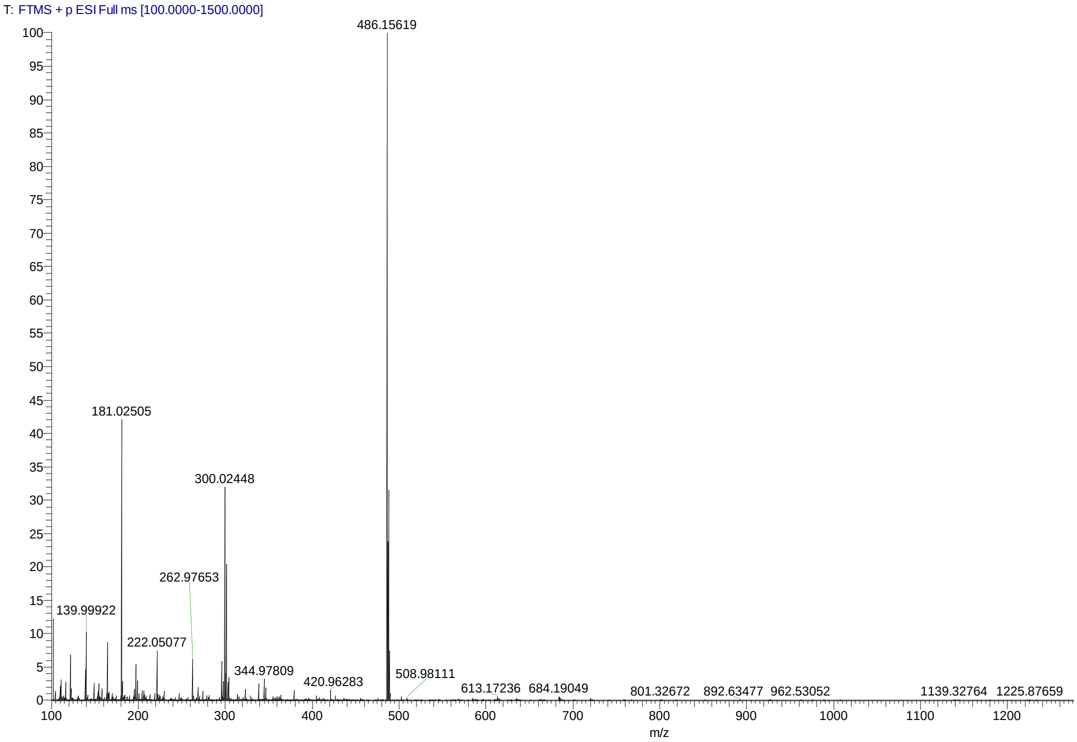


HRMS spectrum of c20

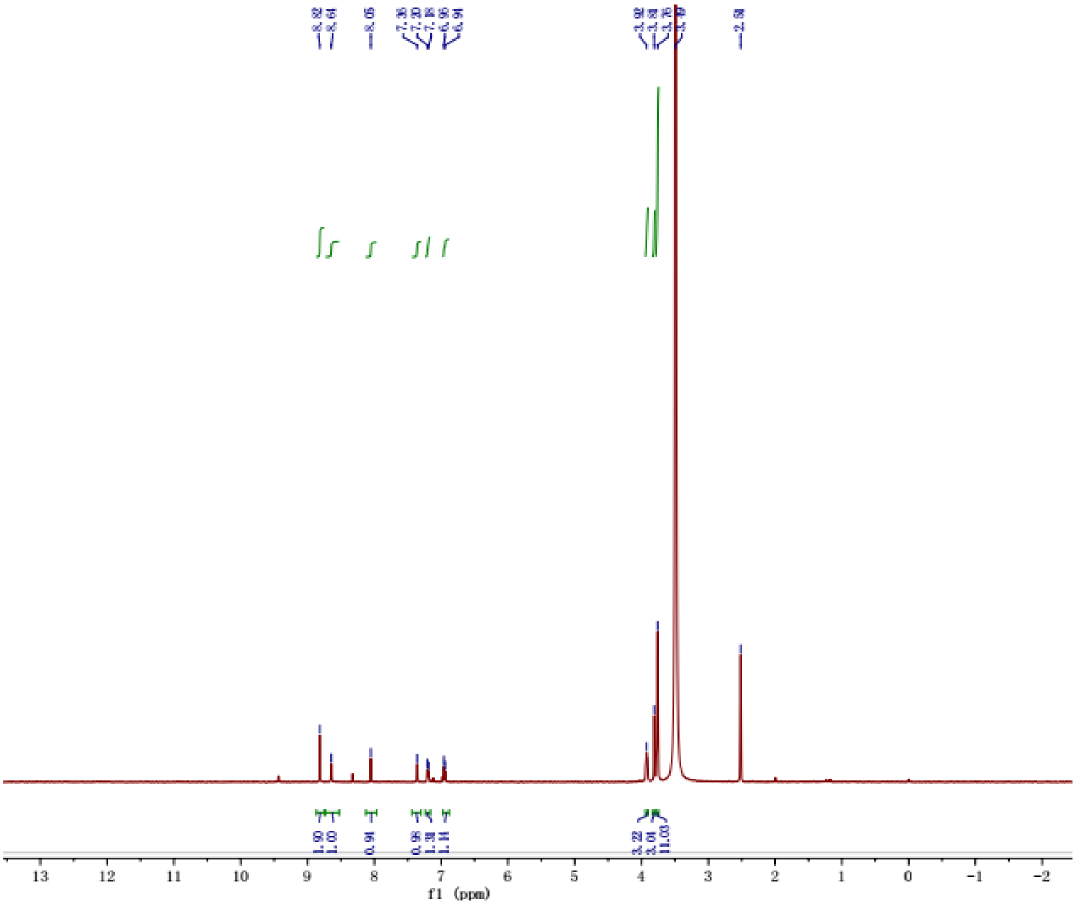


$^1$H-NMR spectrum of c20

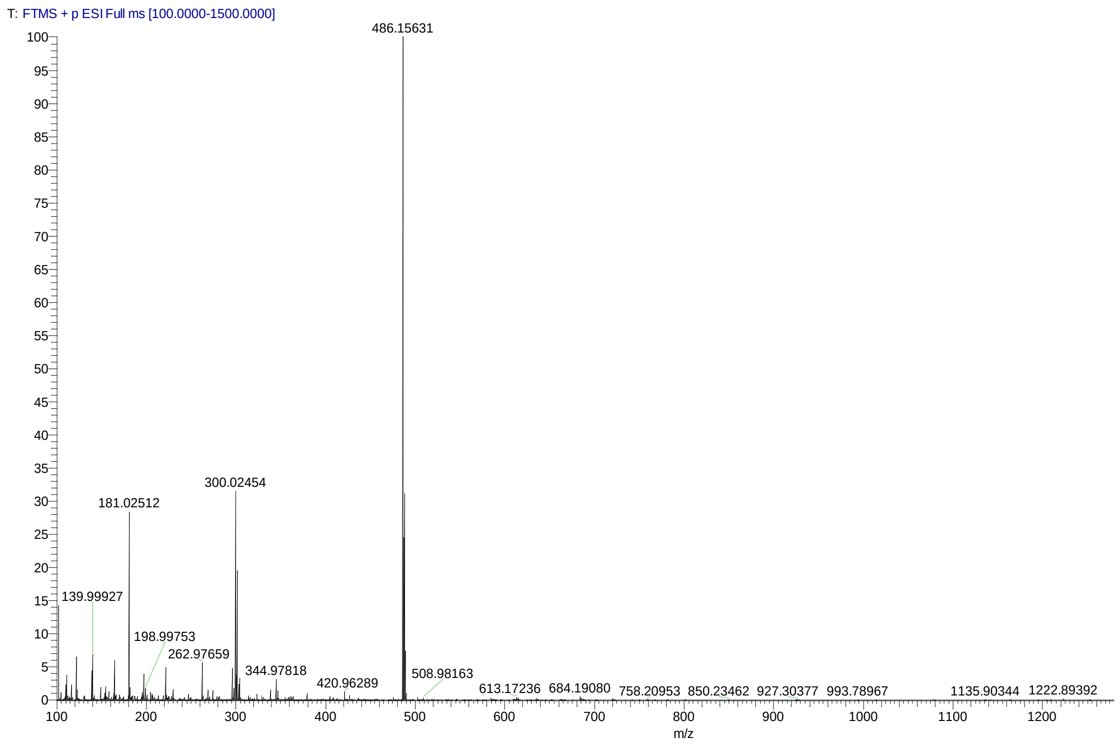


HRMS spectrum of c21

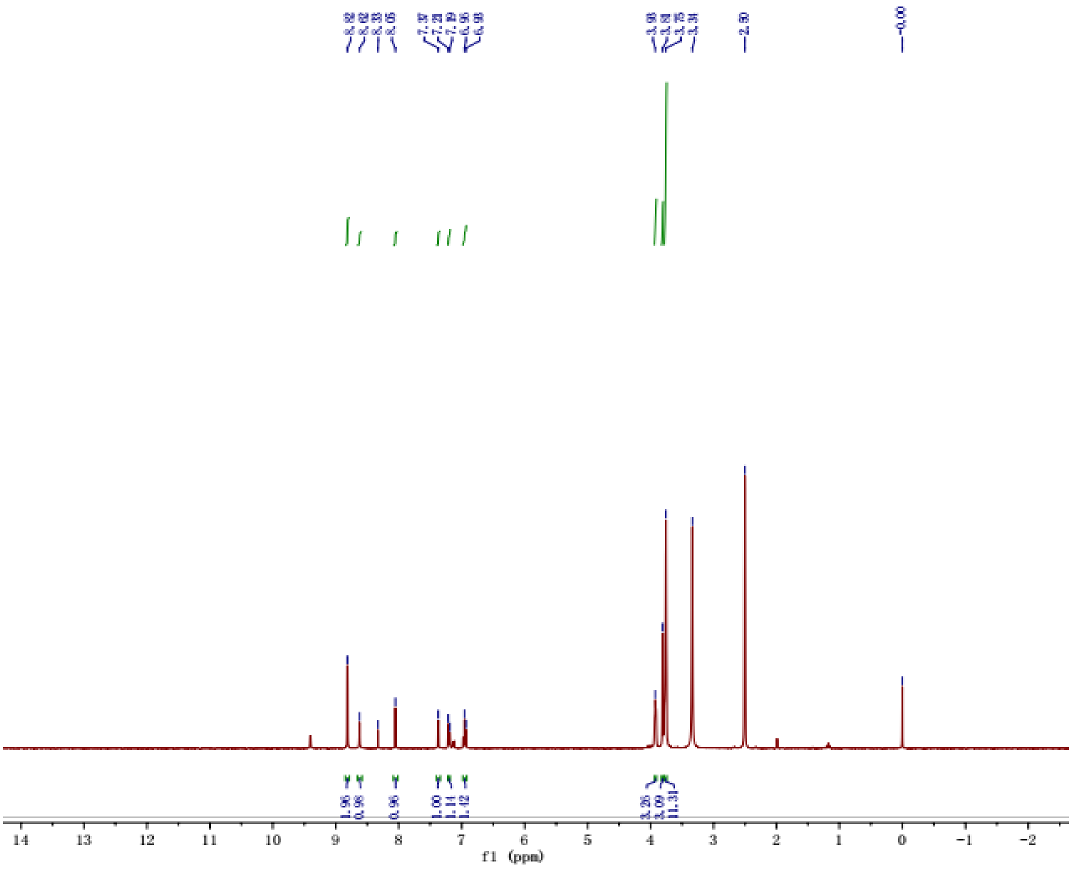


$^1$H-NMR spectrum of c21

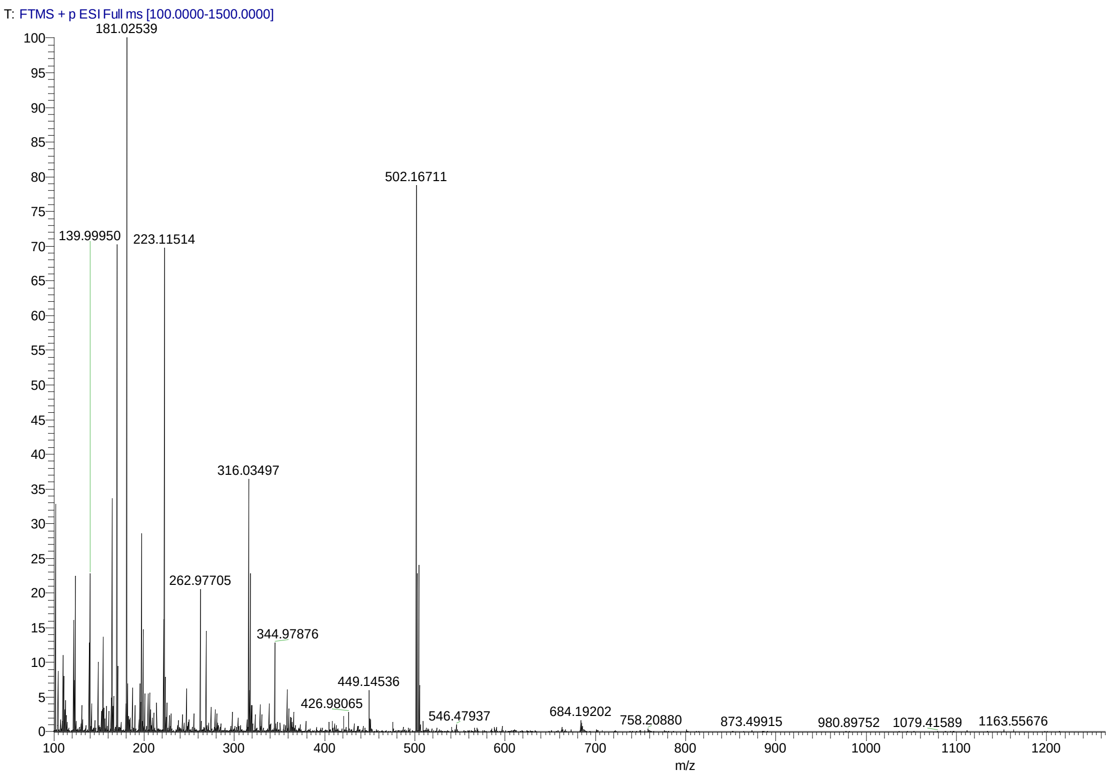


HRMS spectrum of c22

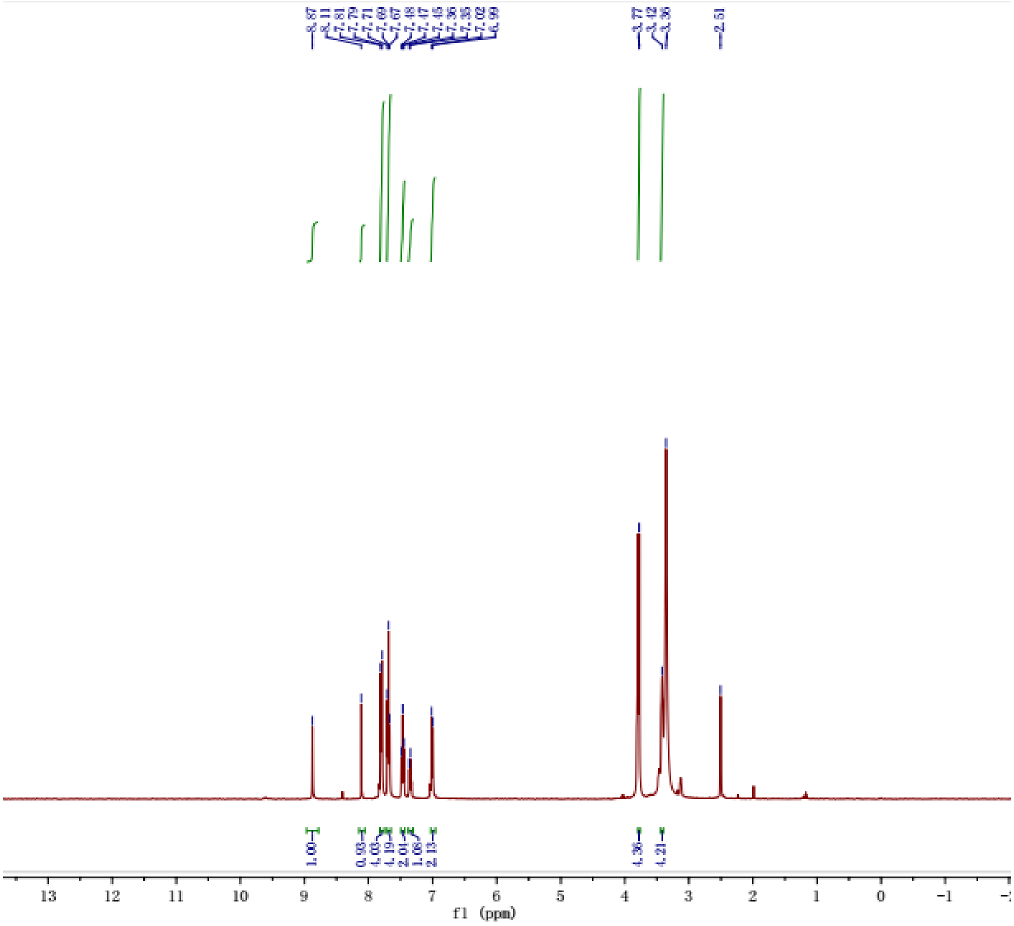


$^{1}$H-NMR spectrum of c22

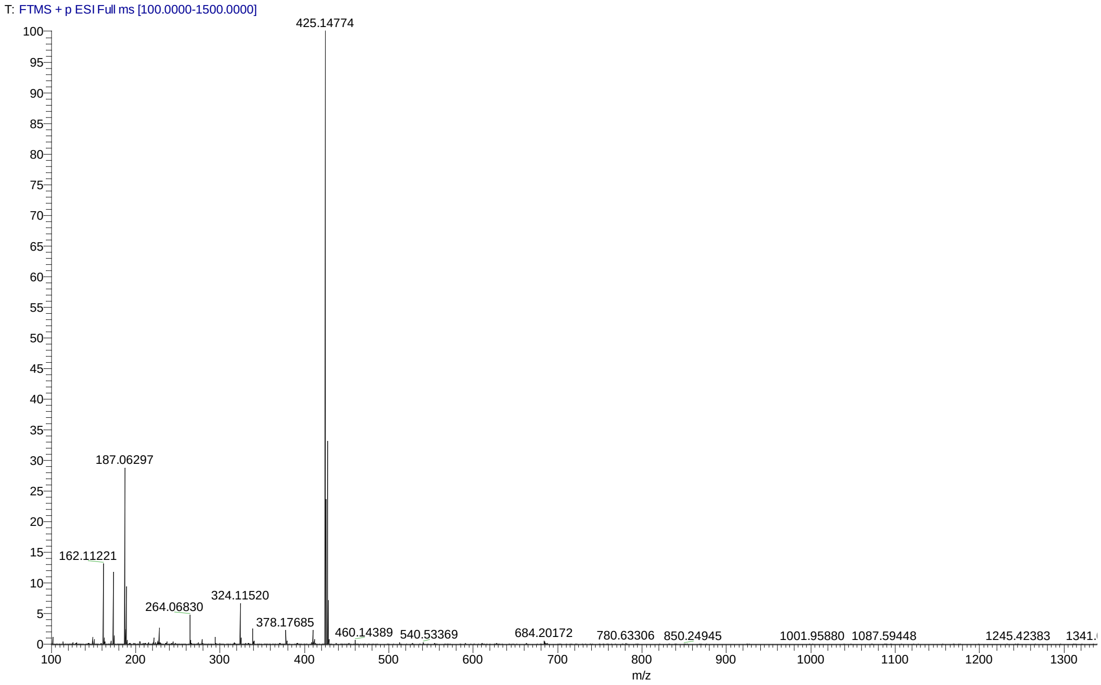


HRMS spectrum of L1

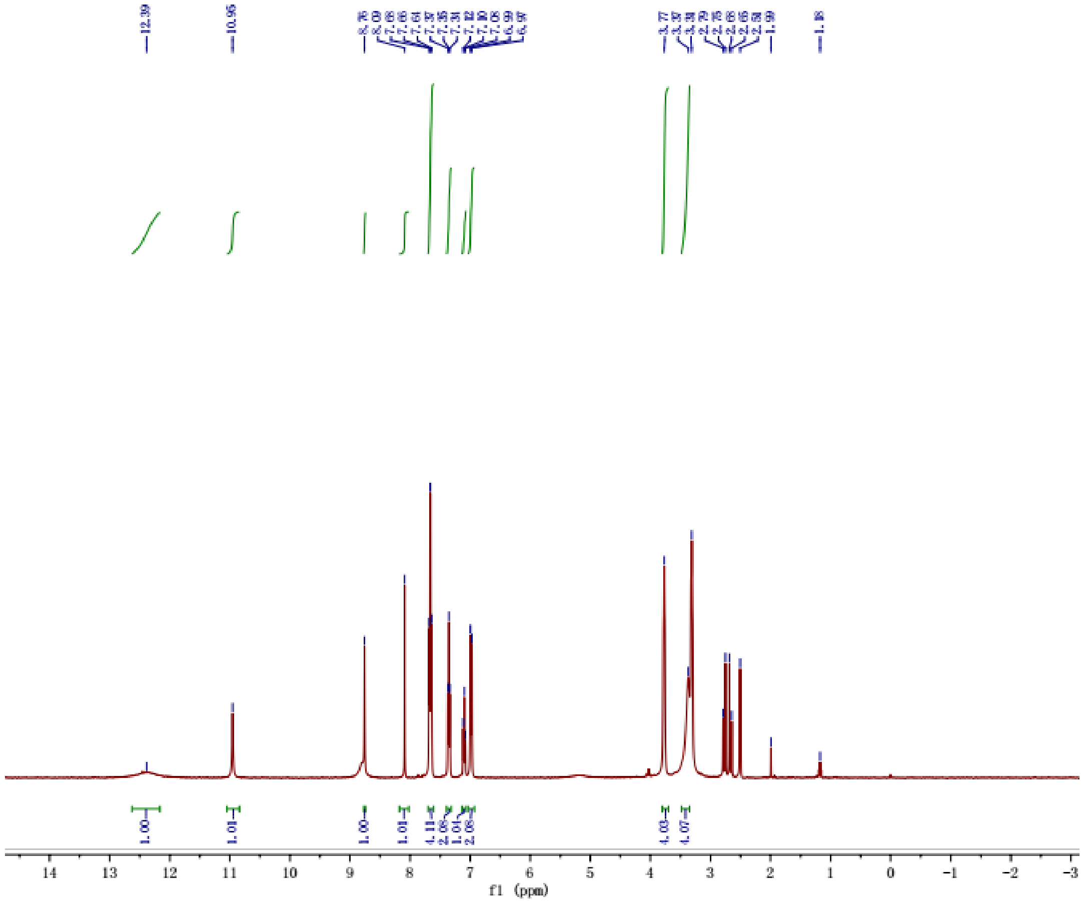


$^{1}$H-NMR spectrum of L1

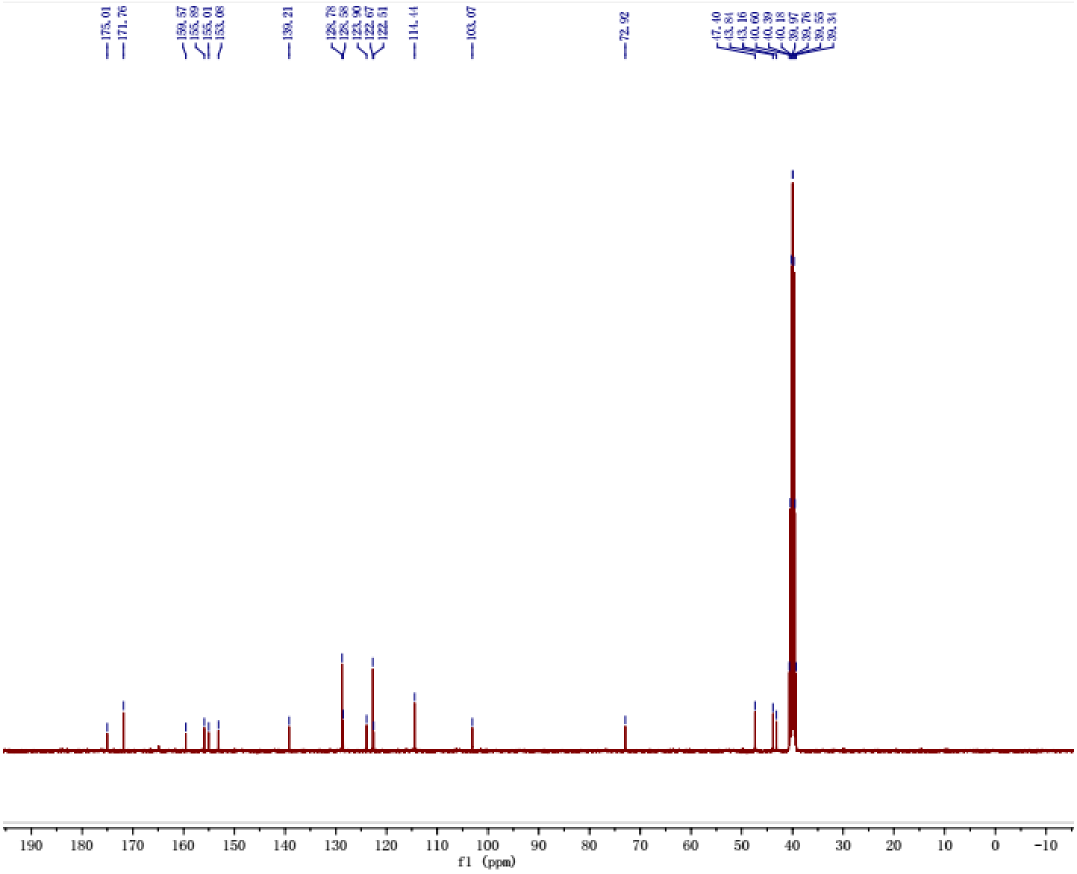


$^{13}$C-NMR spectrum of L1

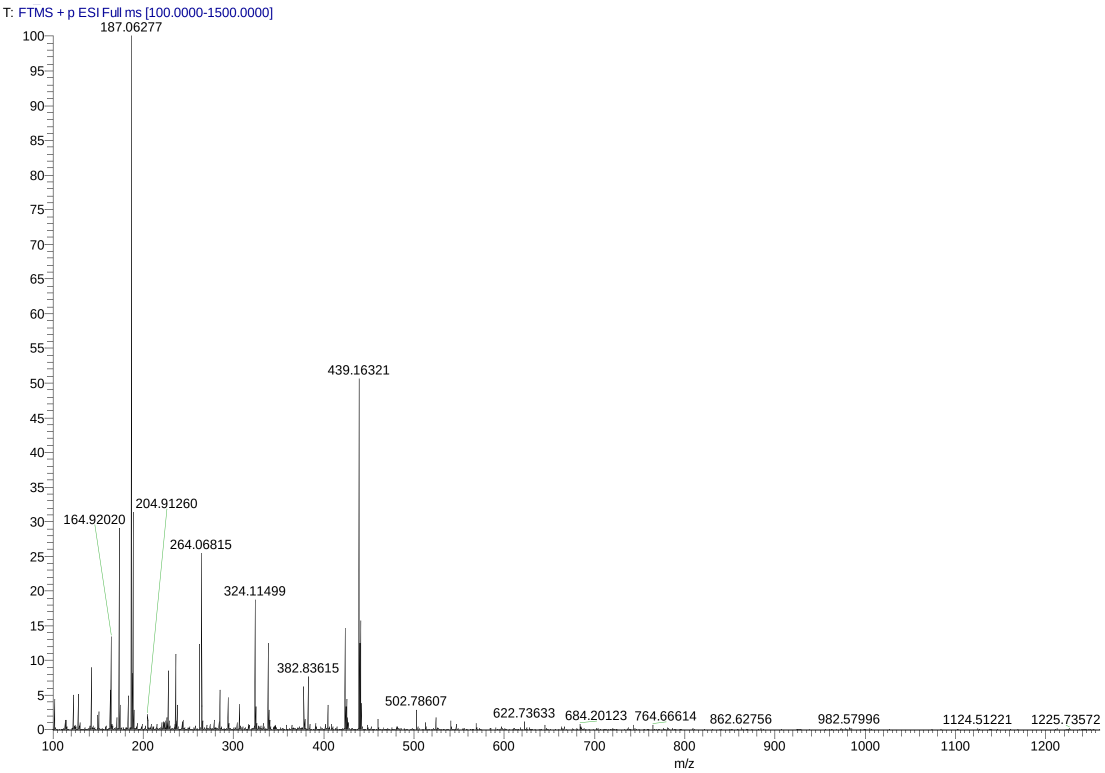


HRMS spectrum of L2

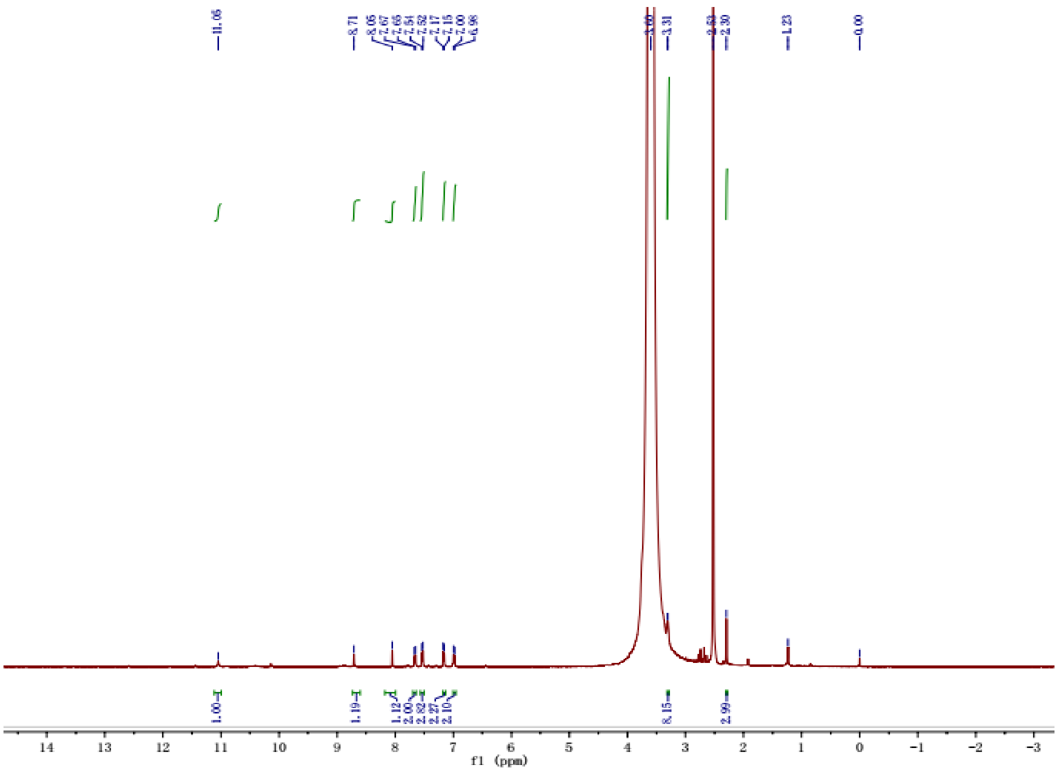

$^{1}$H-NMR spectrum of L2

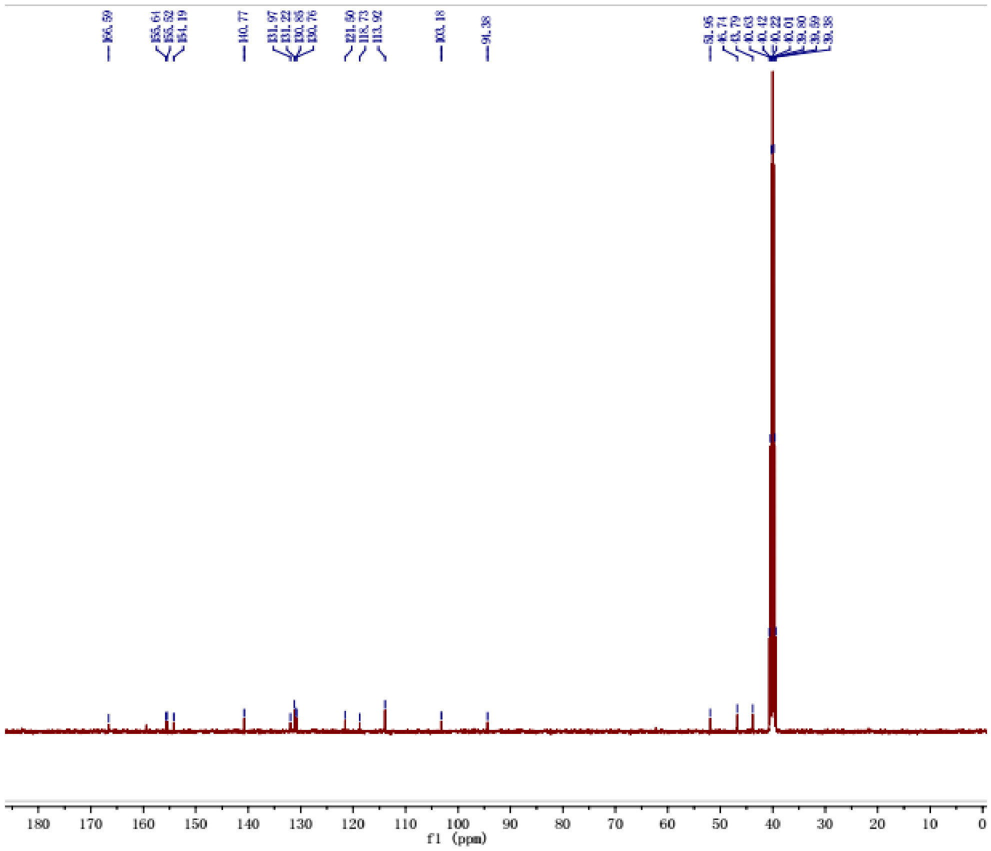

$^{13}$C-NMR spectrum of L2

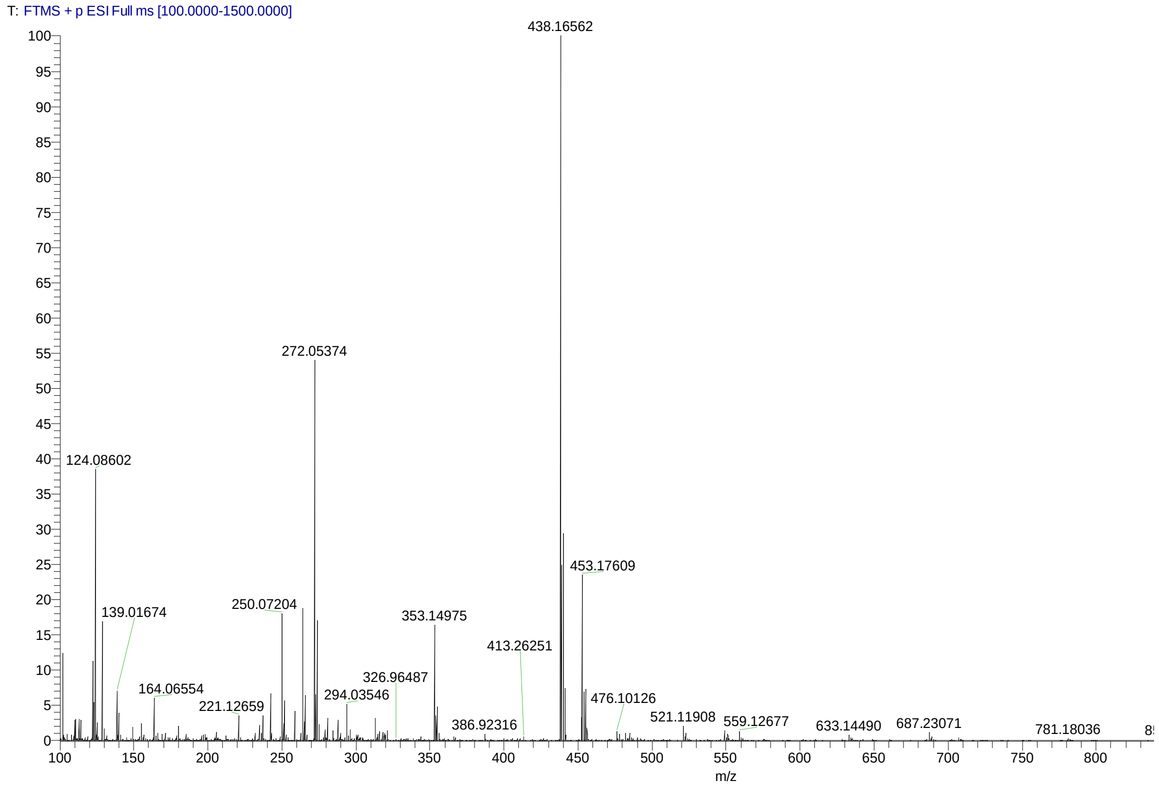


HRMS spectrum of L3

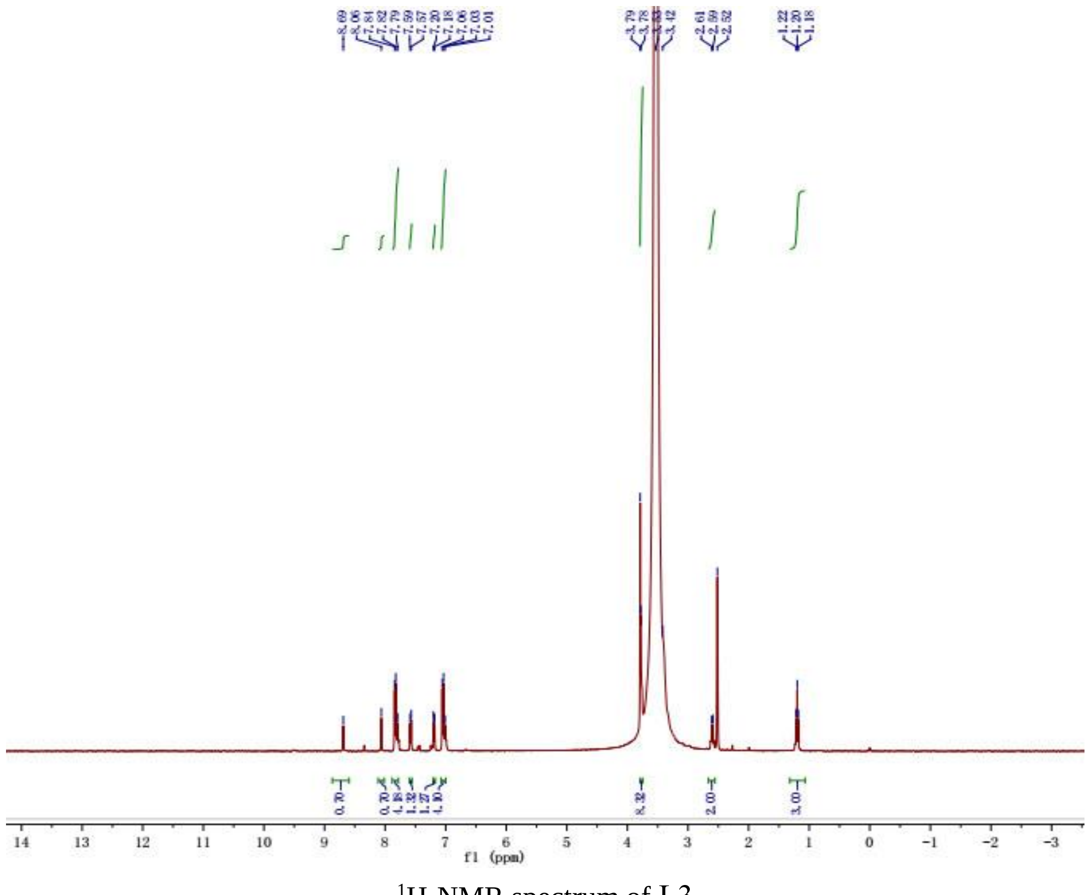


$^{1}$H-NMR spectrum of L3

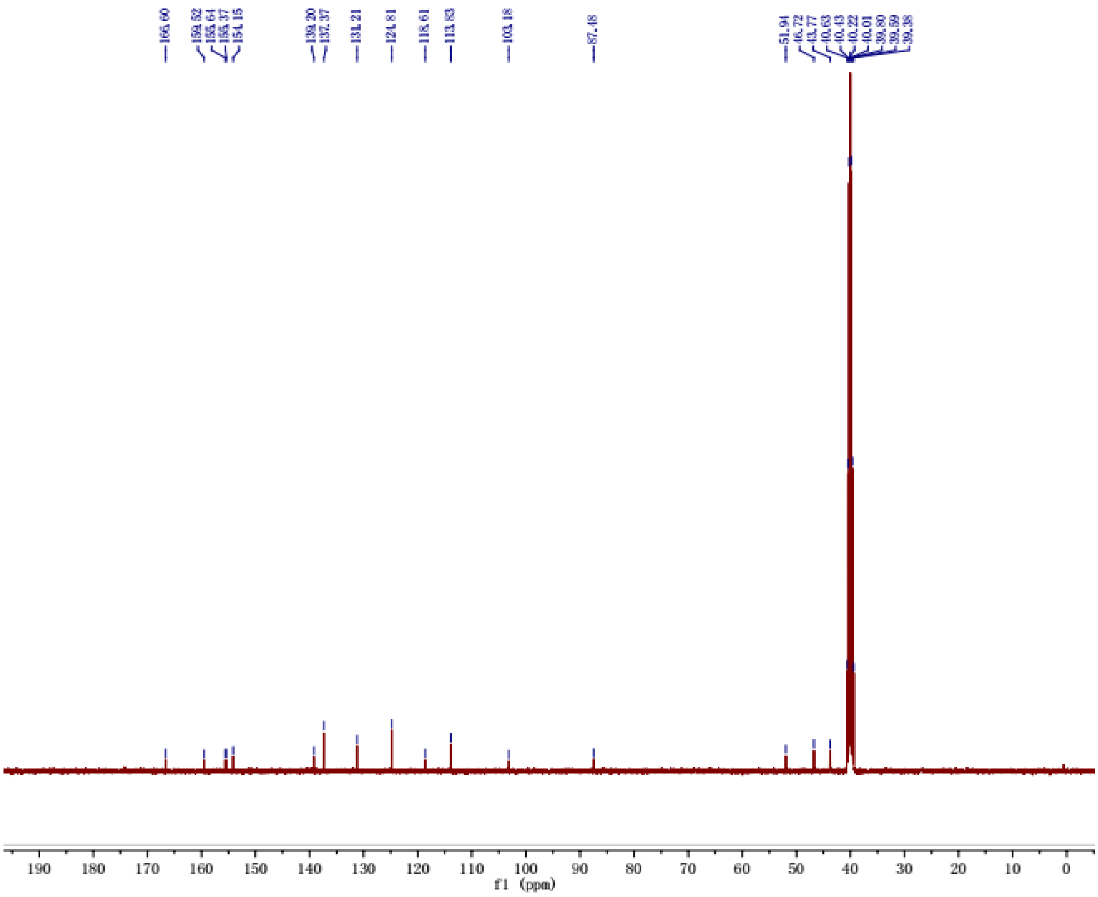


$^{13}$C-NMR spectrum of L3

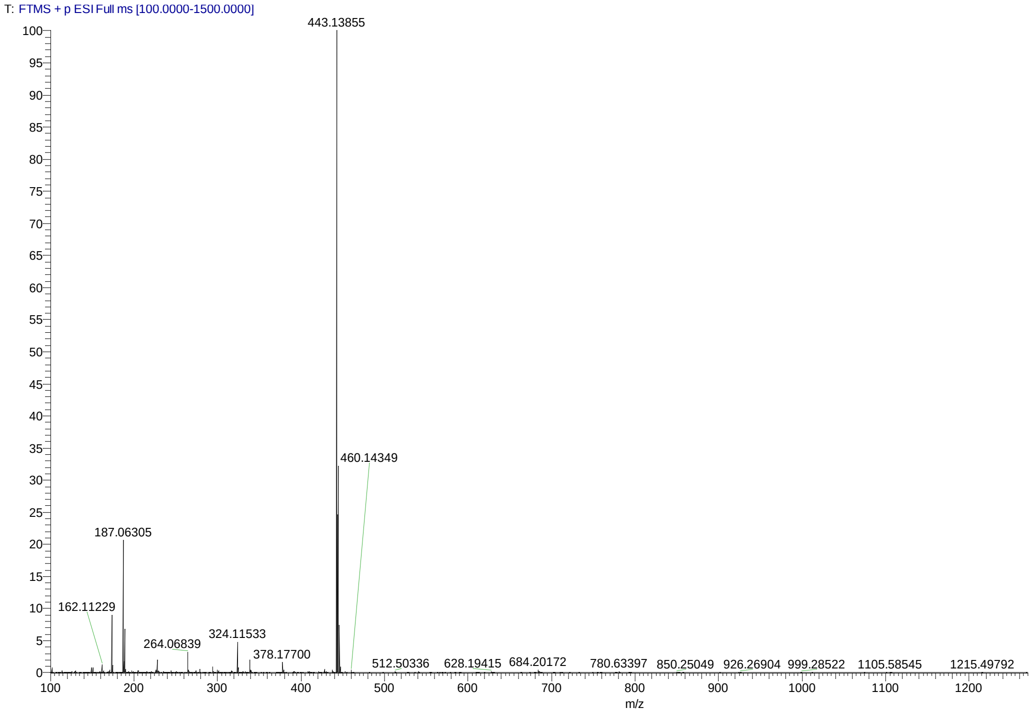


HRMS spectrum of L4

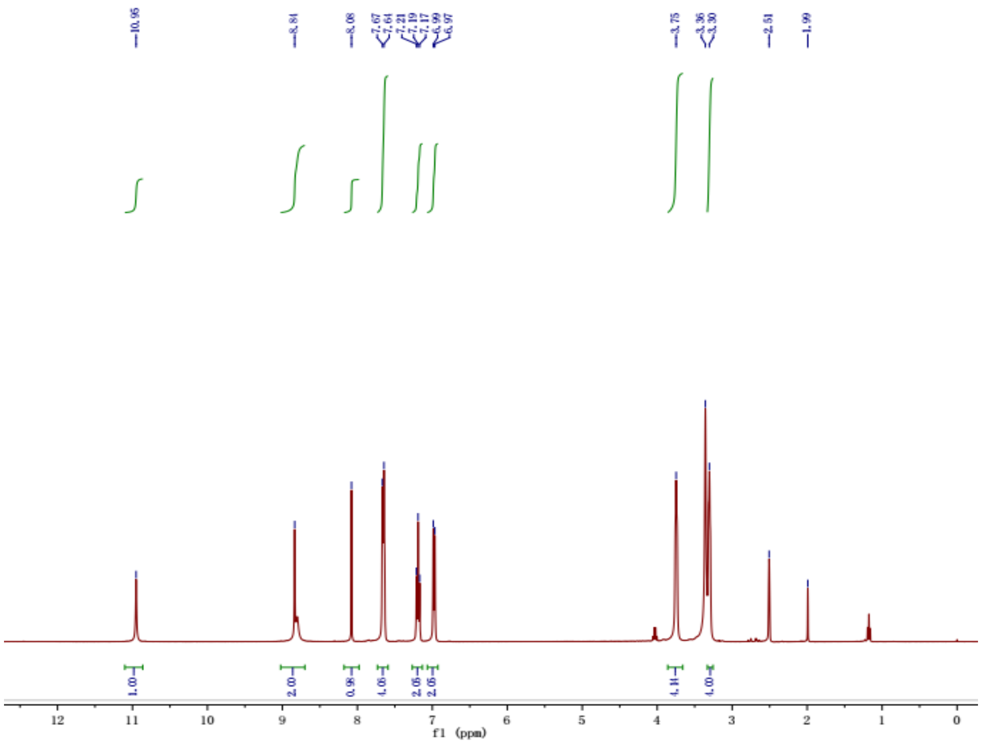

$^{1}$H-NMR spectrum of L4

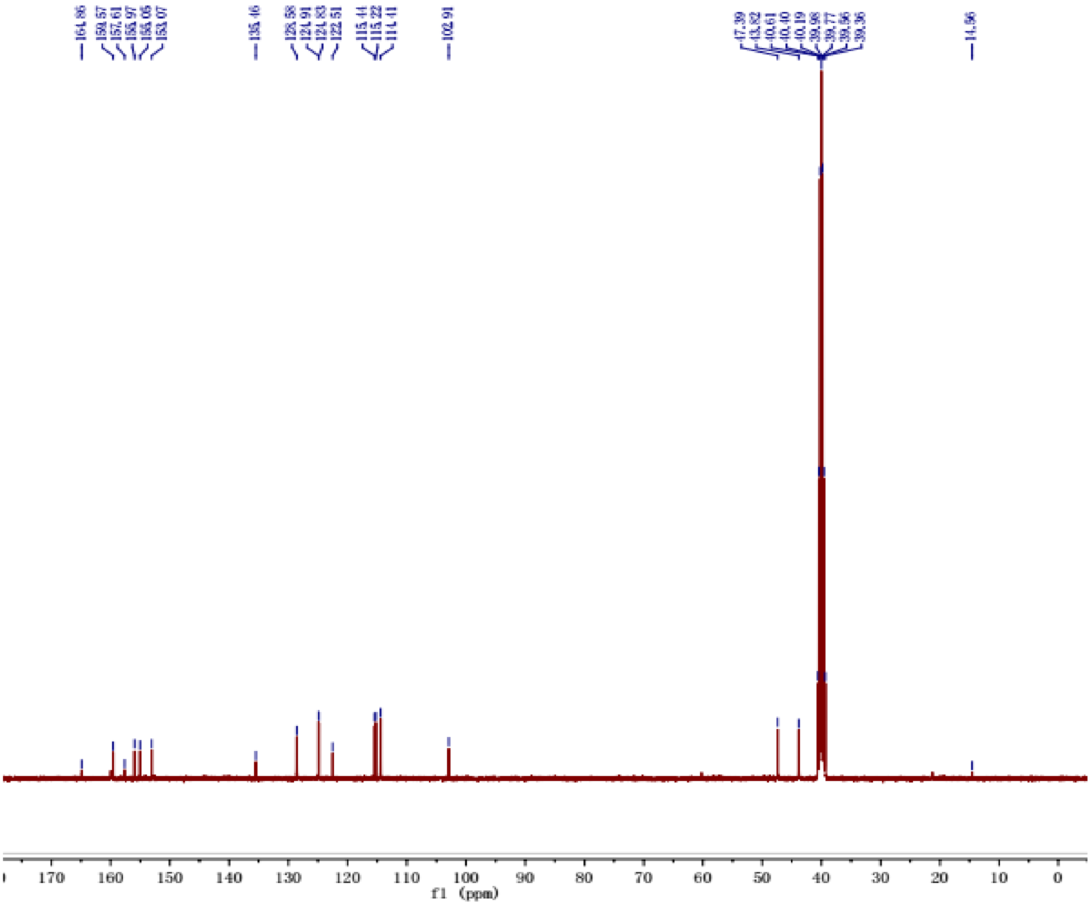

$^{13}$C-NMR spectrum of L4

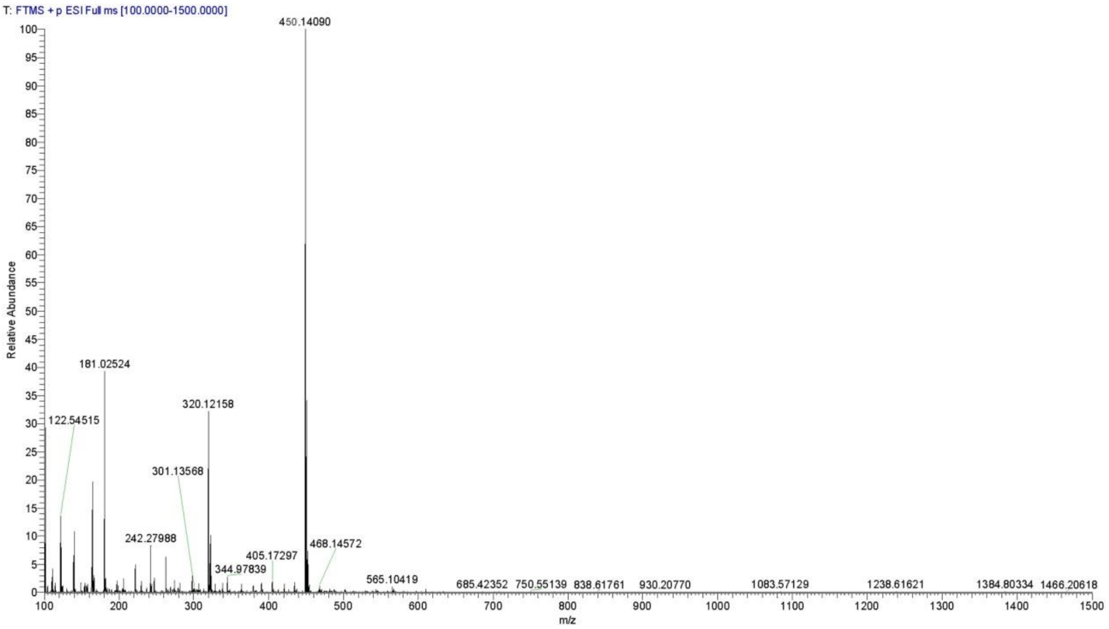


HRMS spectrum of L5

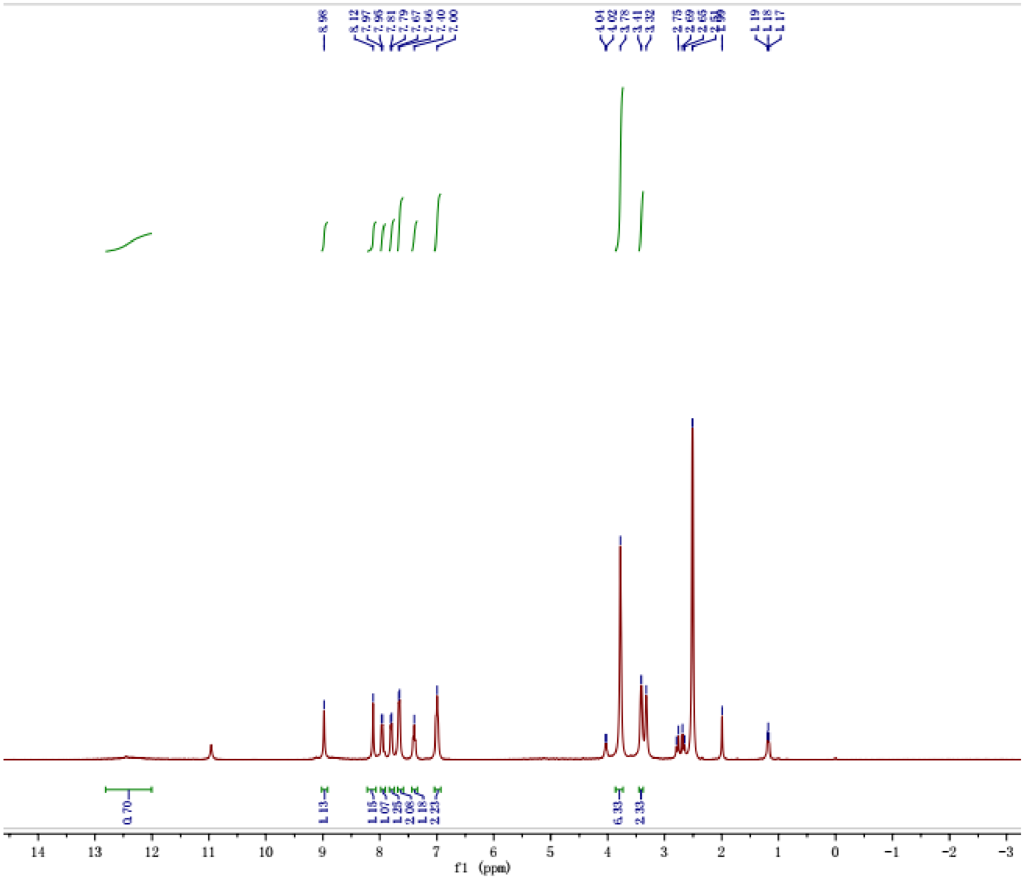


$^1$H-NMR spectrum of L5

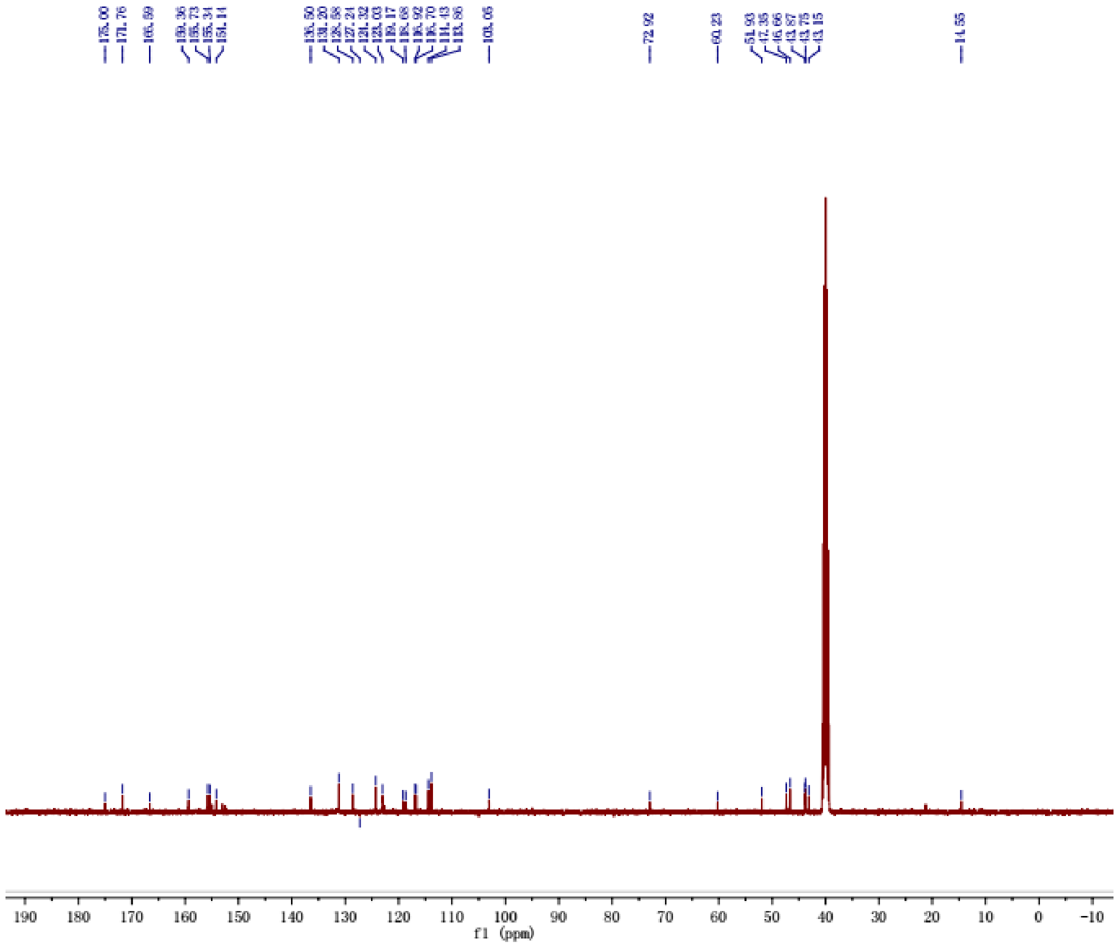


$^{13}$C-NMR spectrum of L5

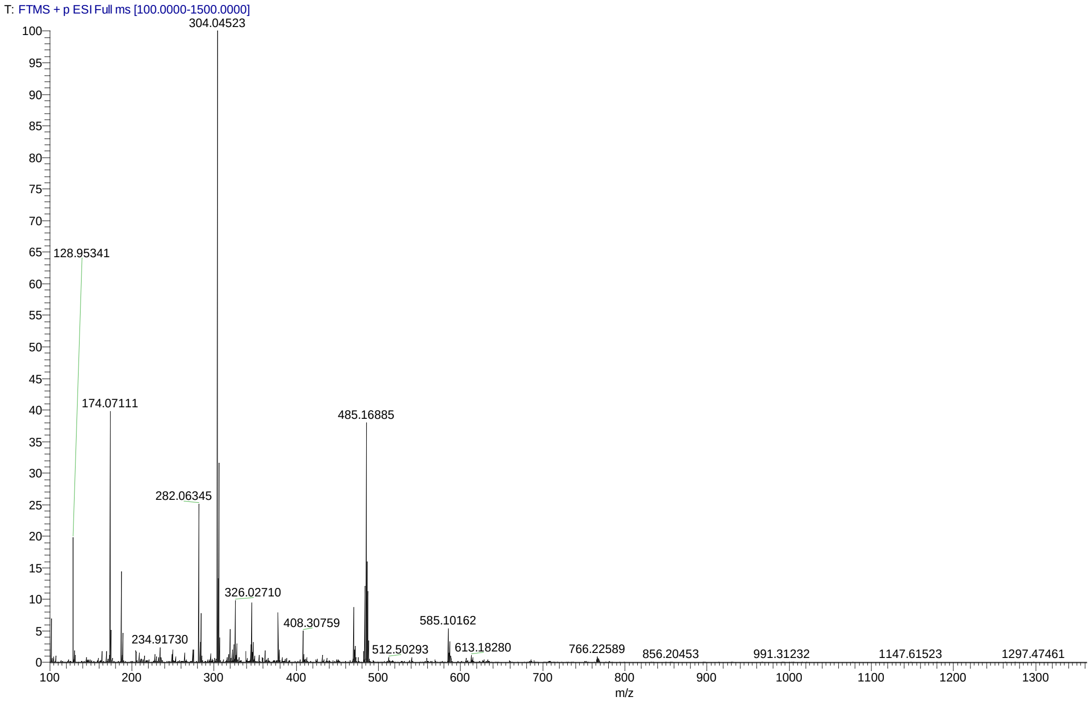


HRMS spectrum of L6

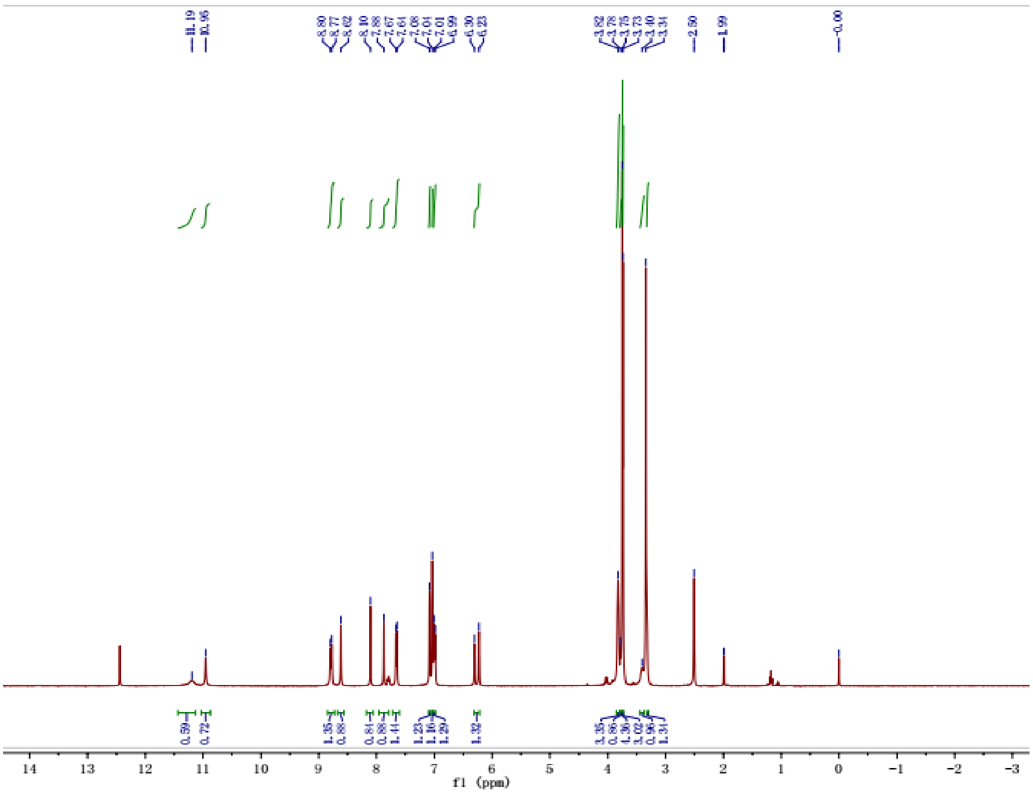

$^{1}$H-NMR spectrum of L6

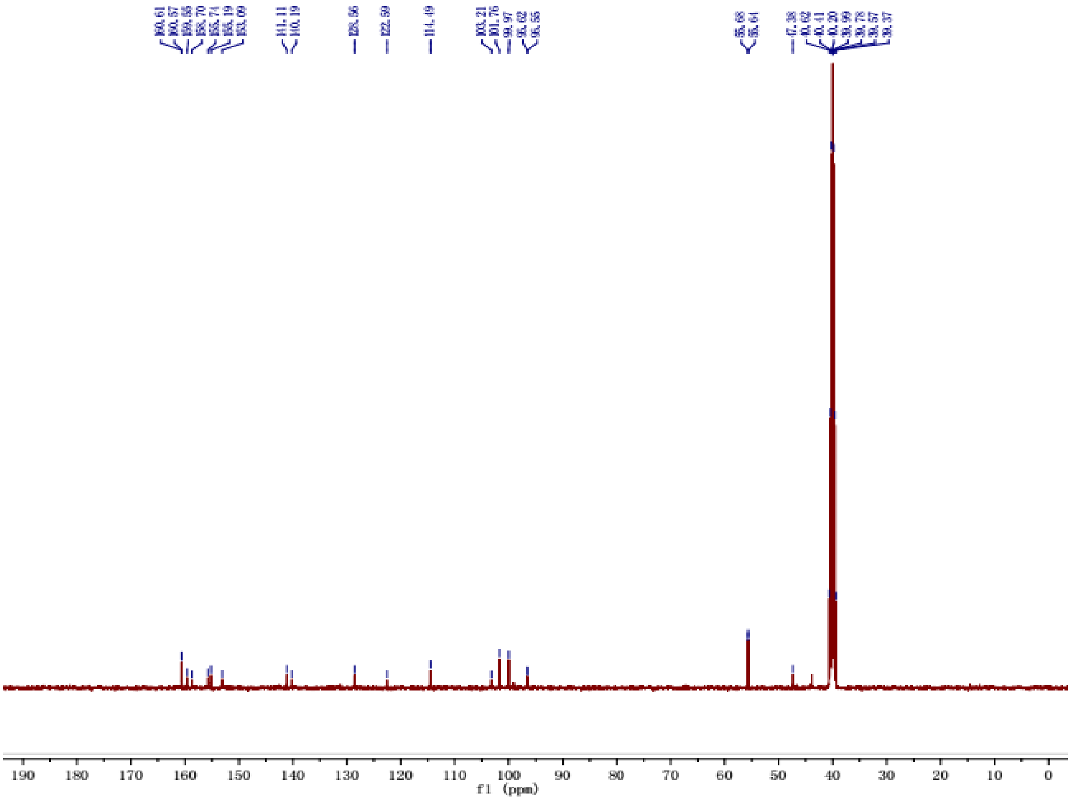

$^{13}$C-NMR spectrum of L6

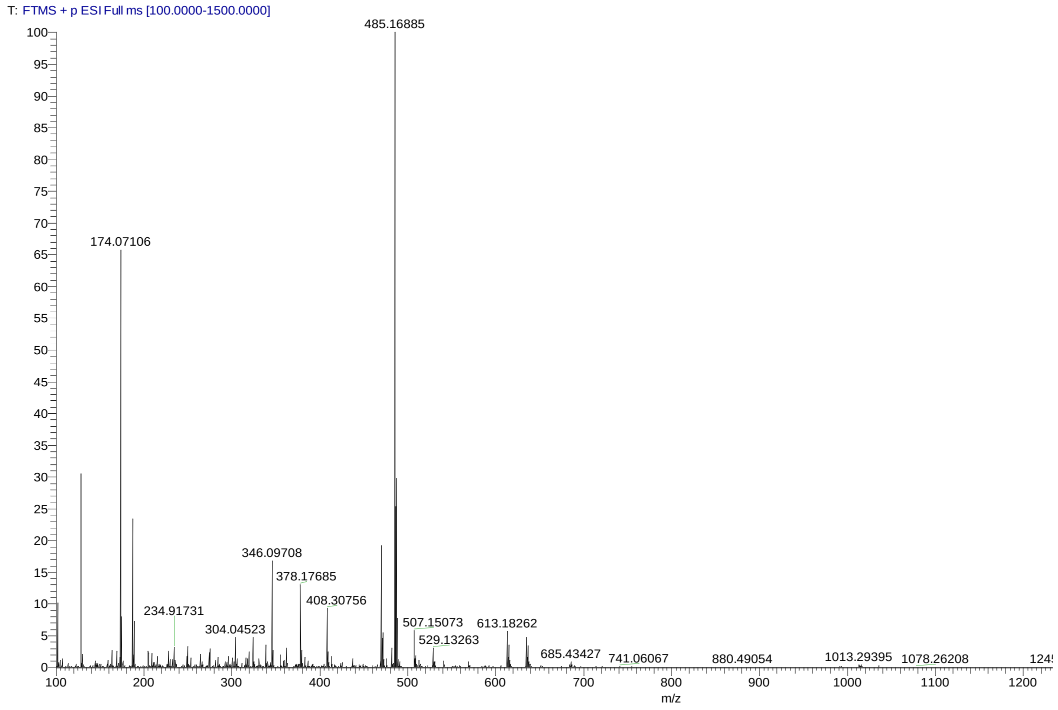


HRMS spectrum of L7

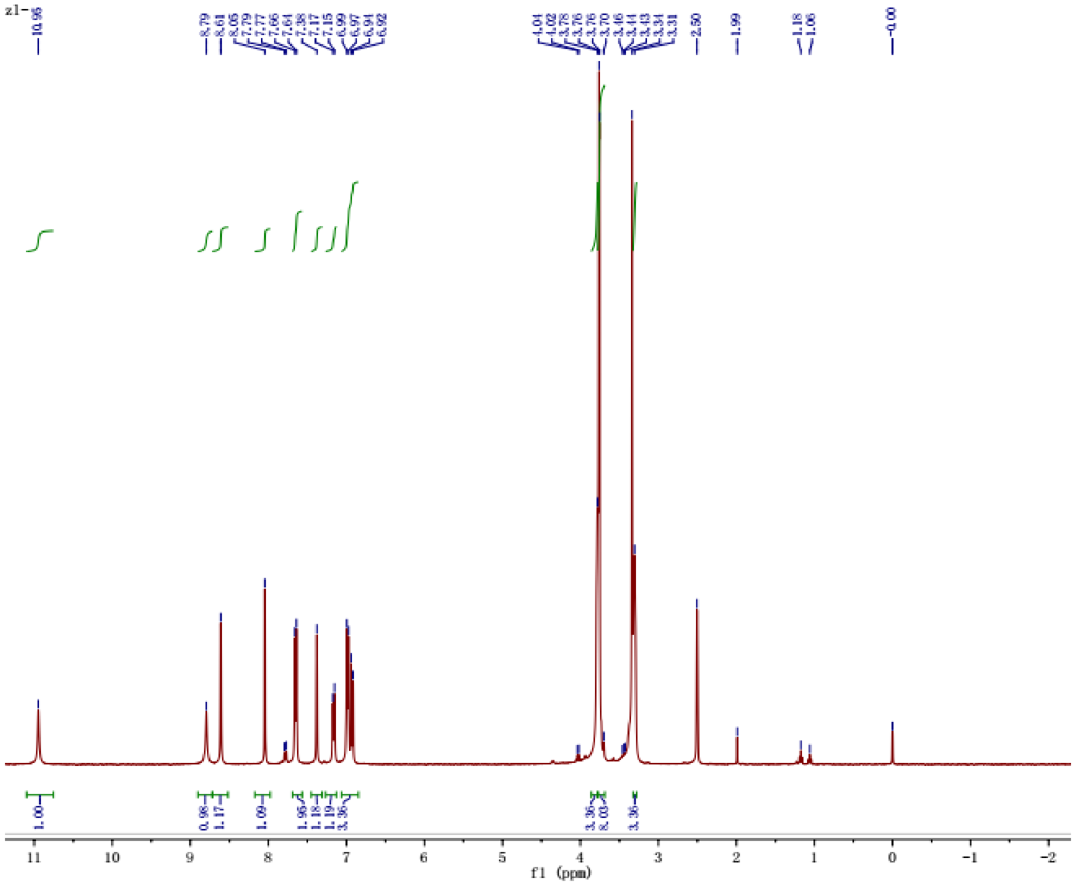


$^{1}$H-NMR spectrum of L7

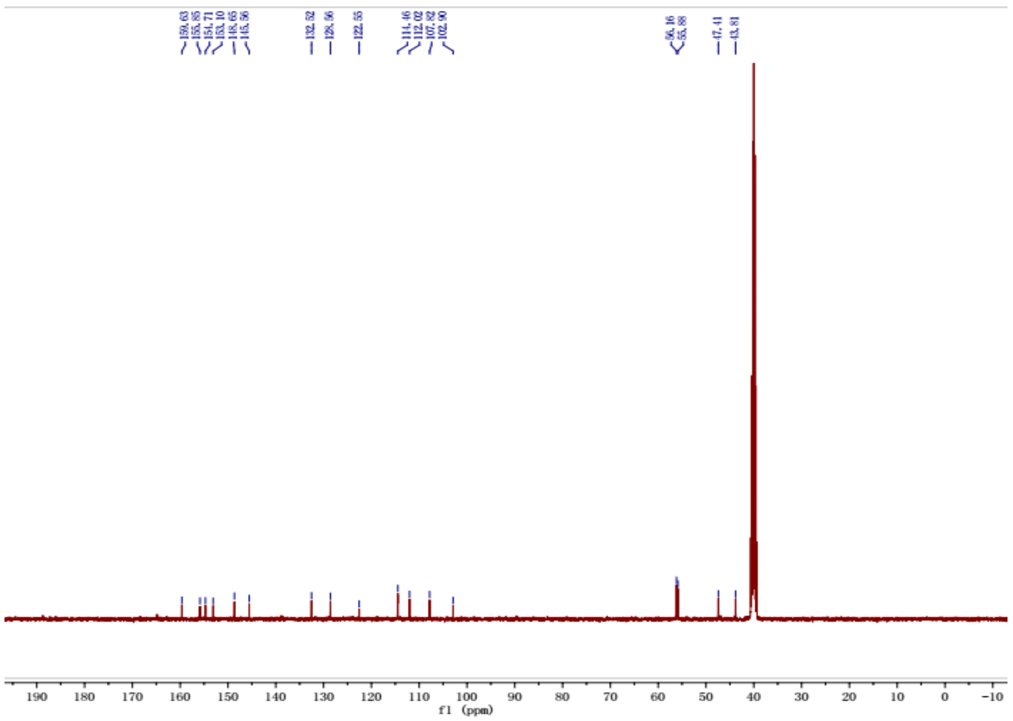


$^{13}$C-NMR spectrum of L7

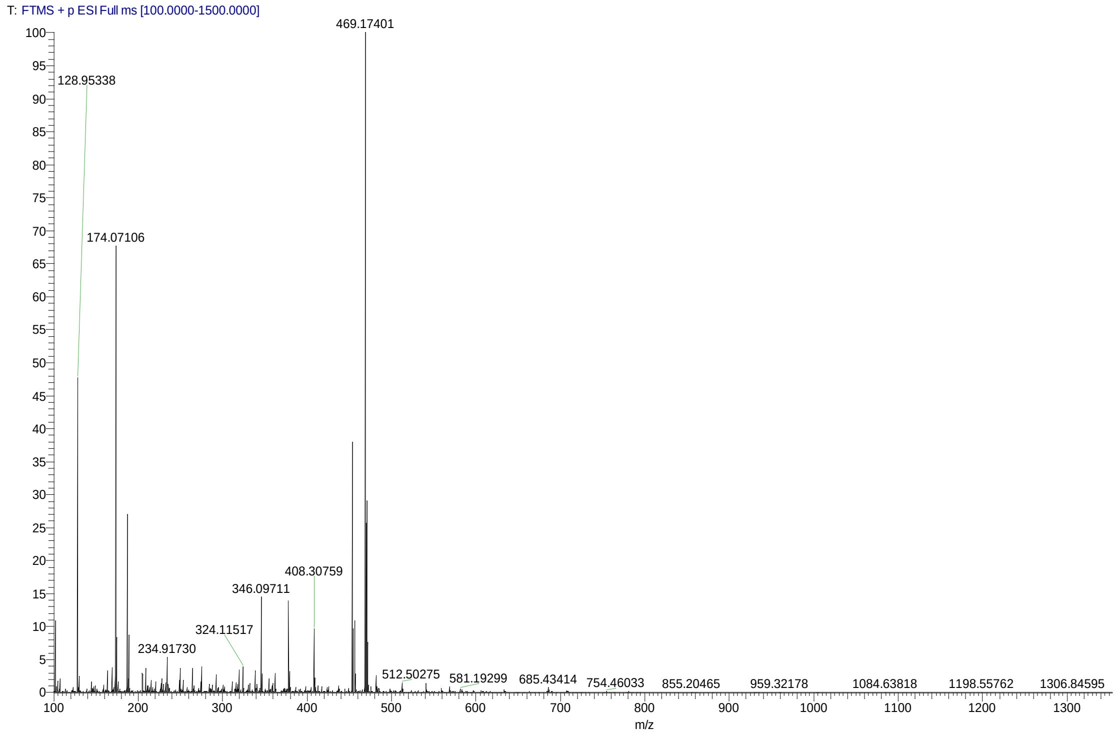


HRMS spectrum of L8

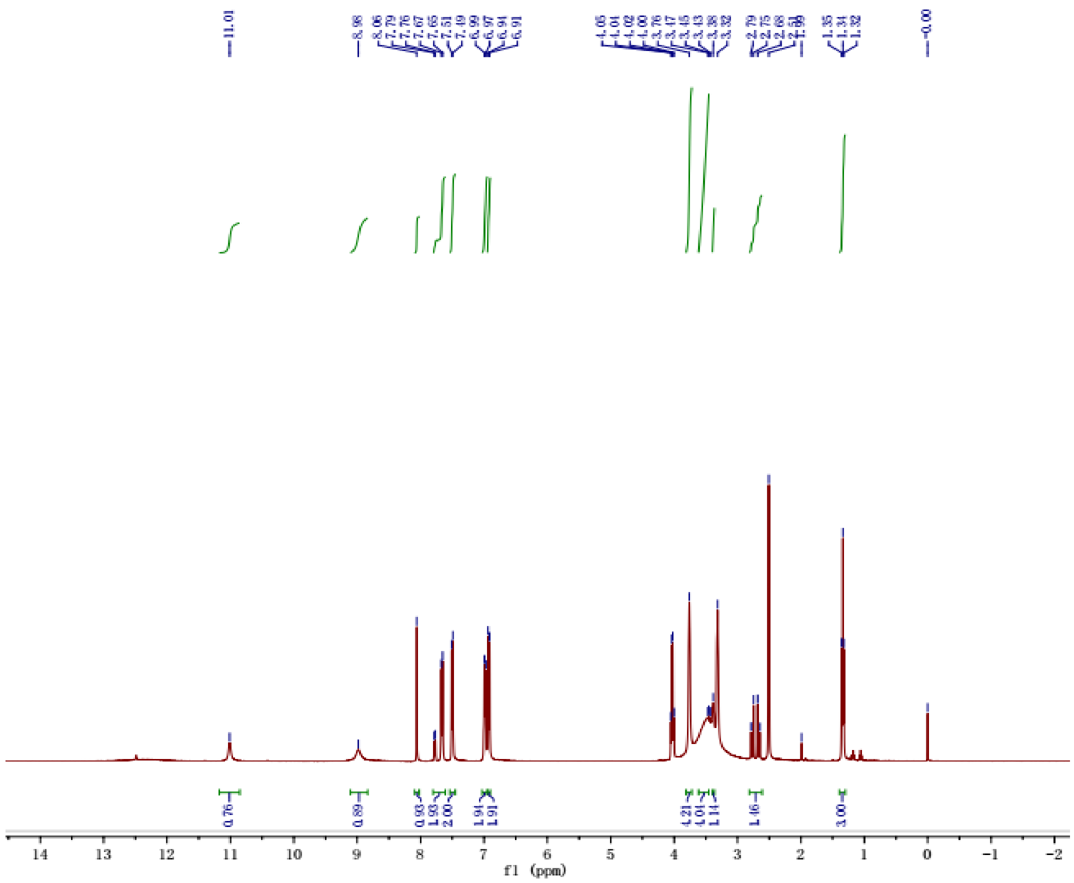

$^{1}$H-NMR spectrum of L8

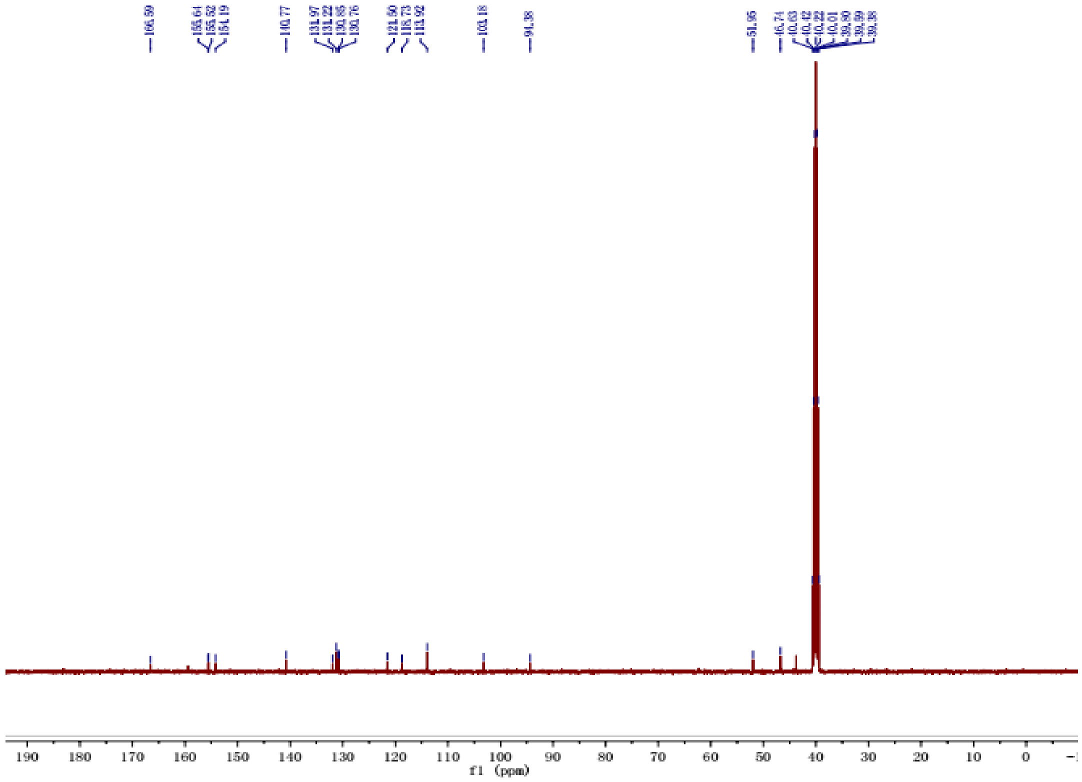

$^{13}$C-NMR spectrum of L8

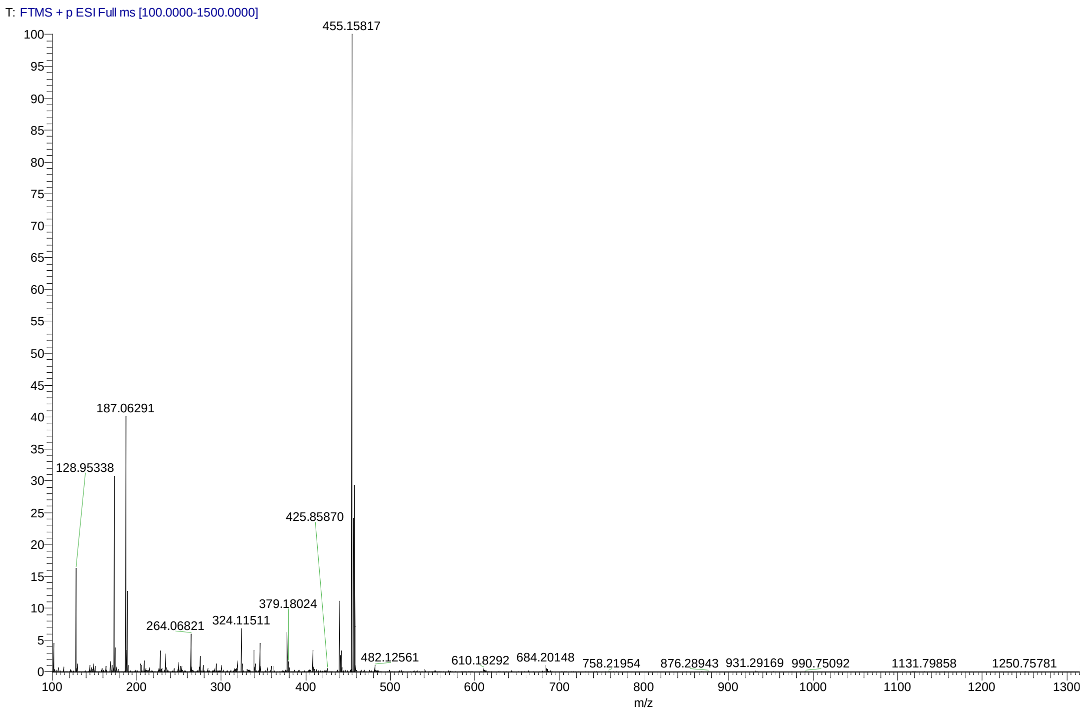


HRMS spectrum of L9

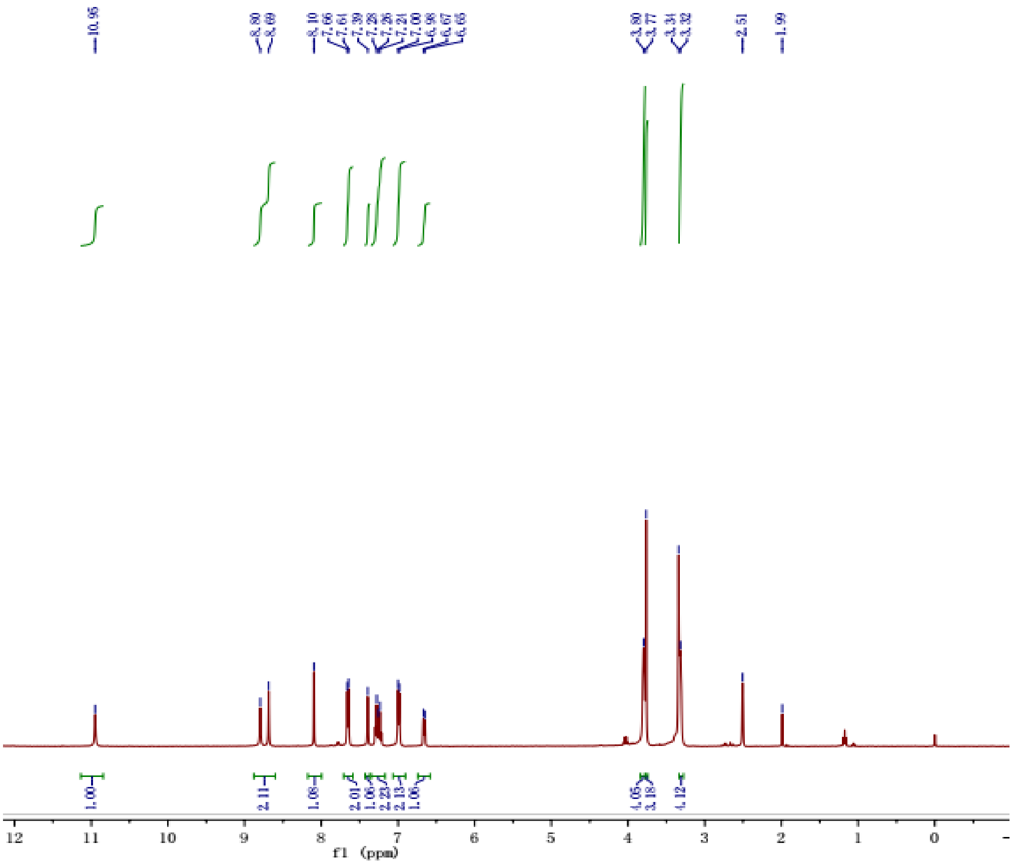


$^{1}$H-NMR spectrum of L9

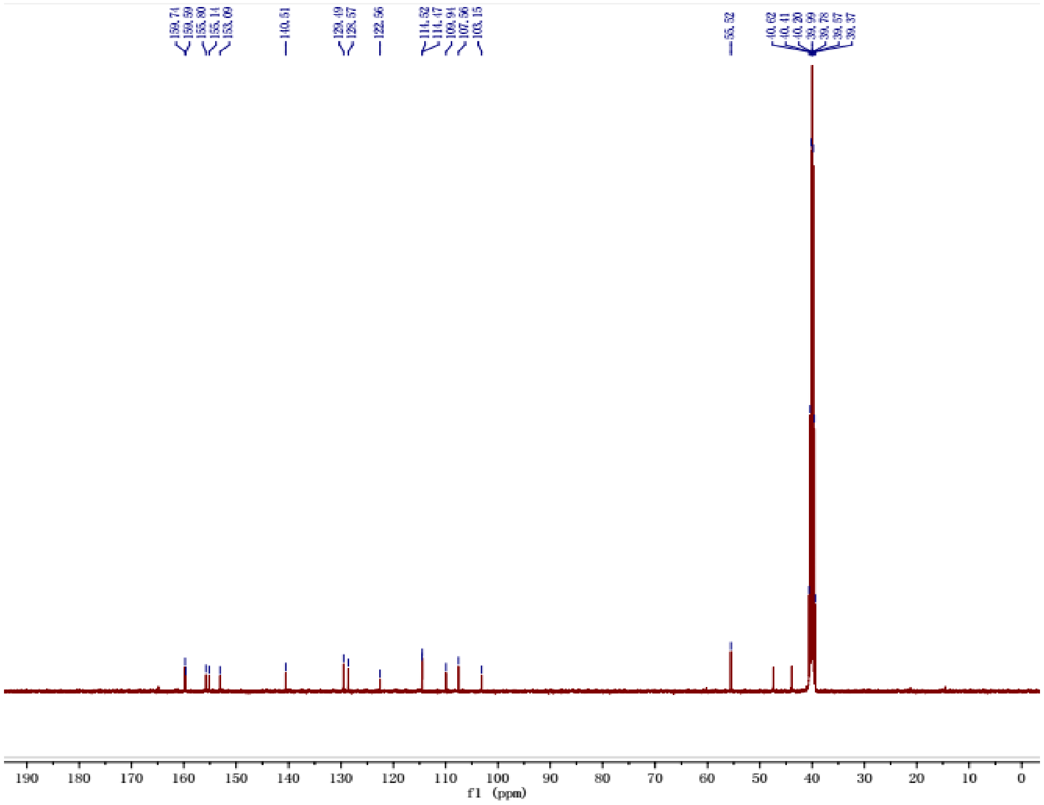


$^{13}$C-NMR spectrum of L9

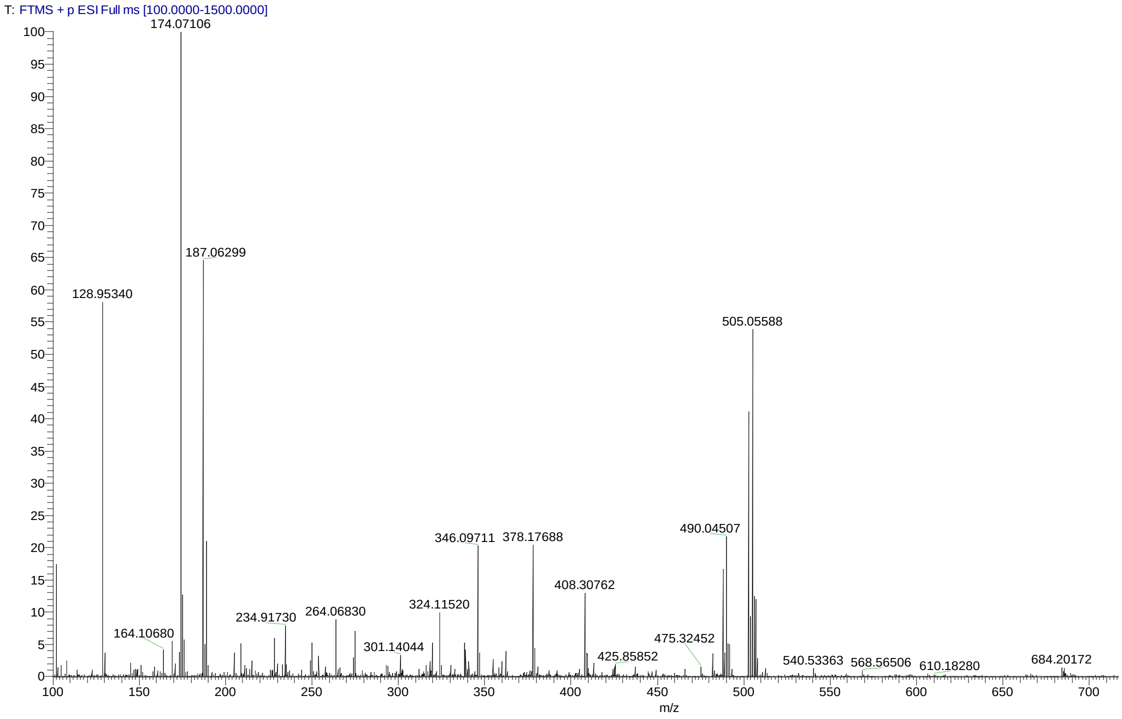


HRMS spectrum of L10

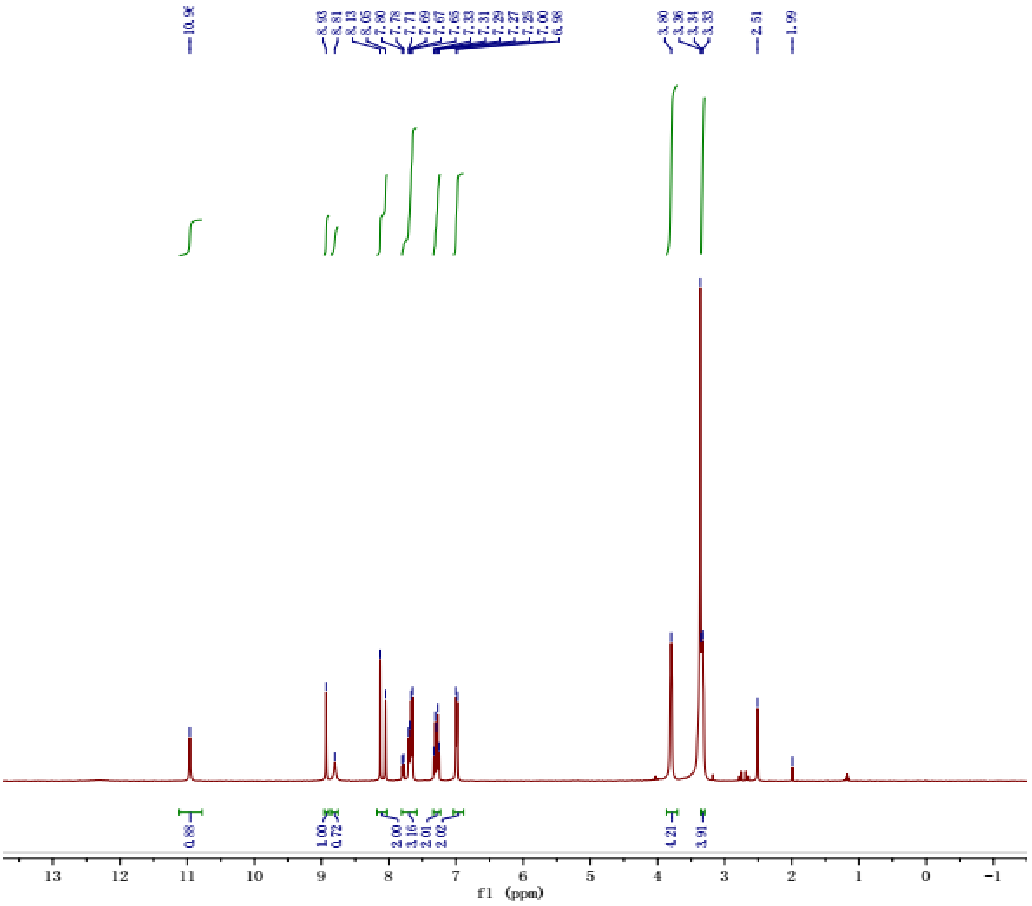

$^{1}$H-NMR spectrum of L10

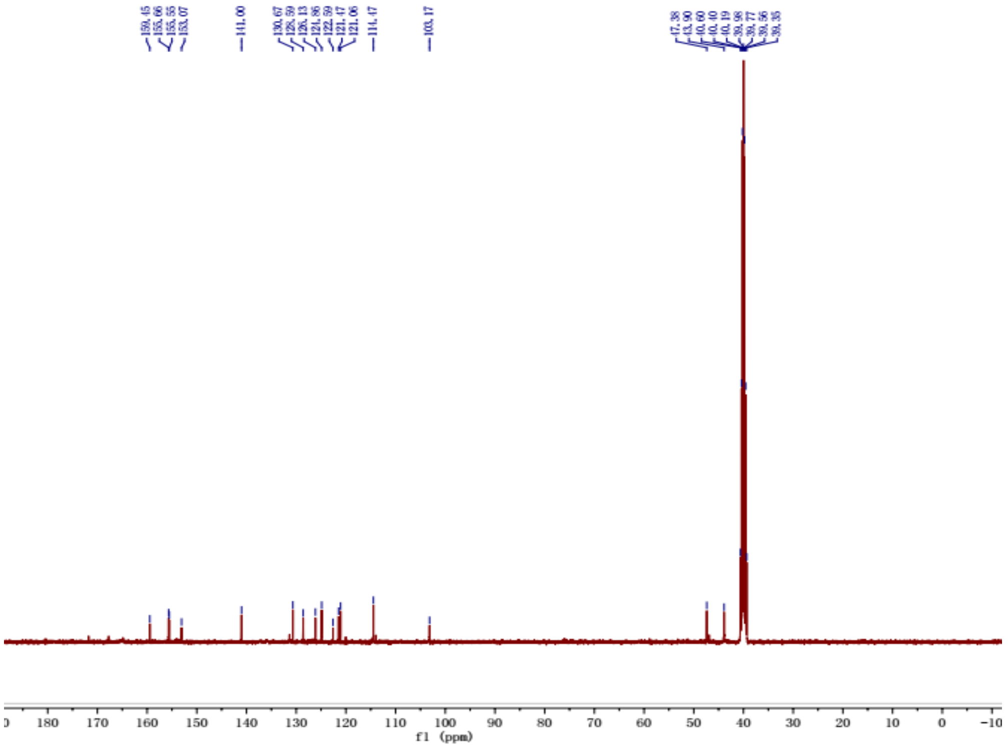

$^{13}$C-NMR spectrum of L10

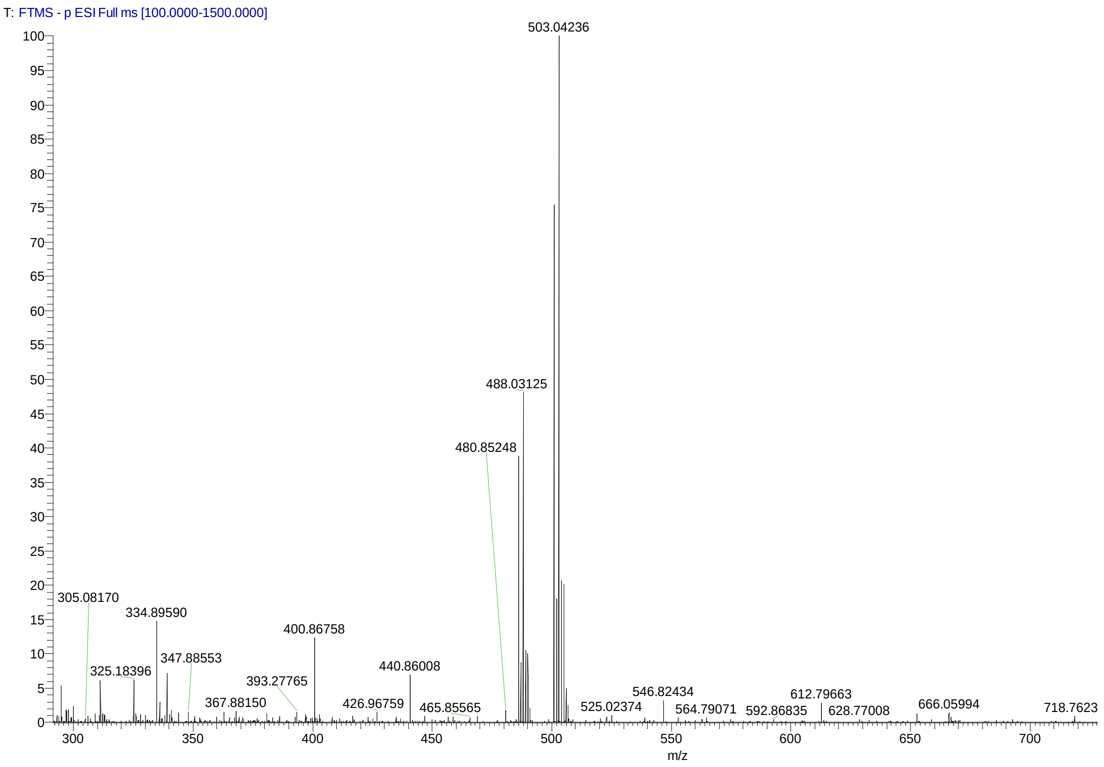


HRMS spectrum of L11

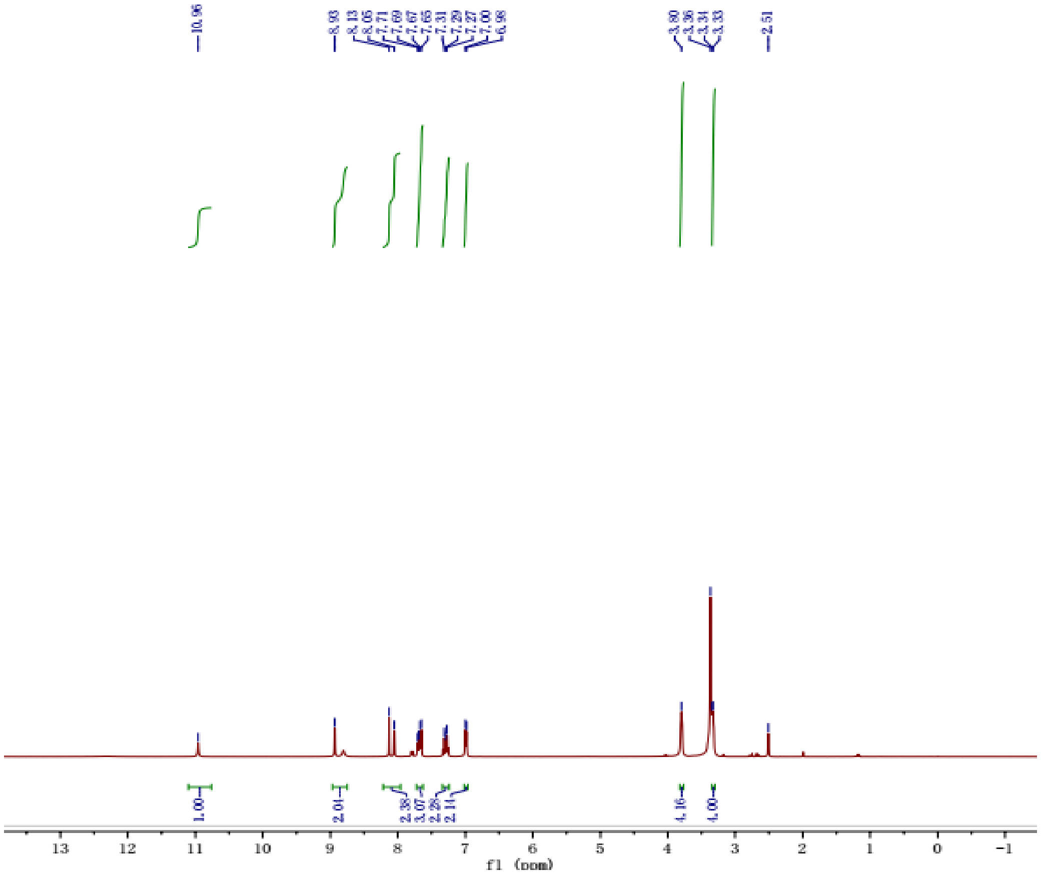


$^{1}$H-NMR spectrum of L11

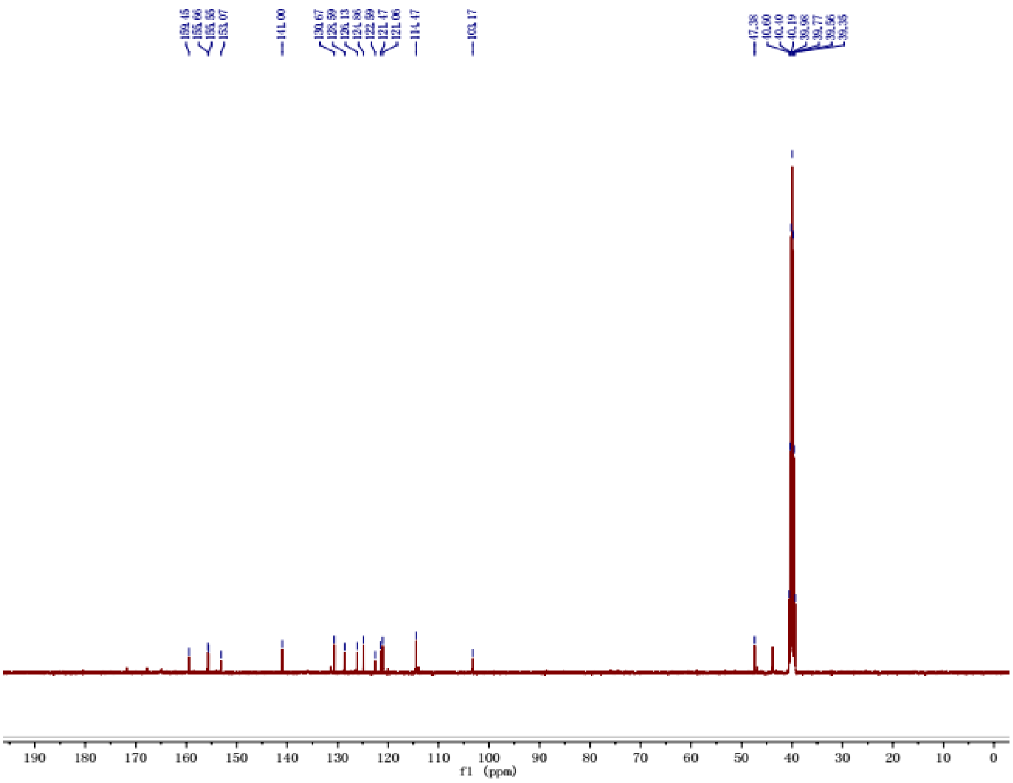


$^{13}$C-NMR spectrum of L11

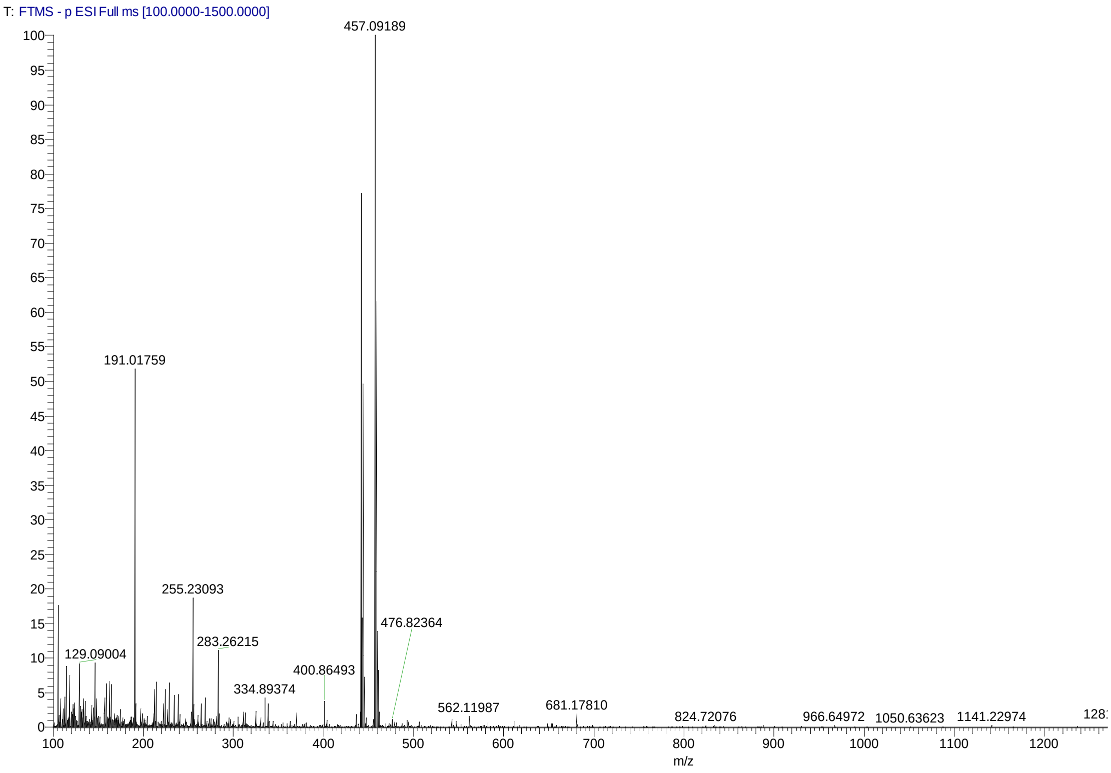


HRMS spectrum of L12

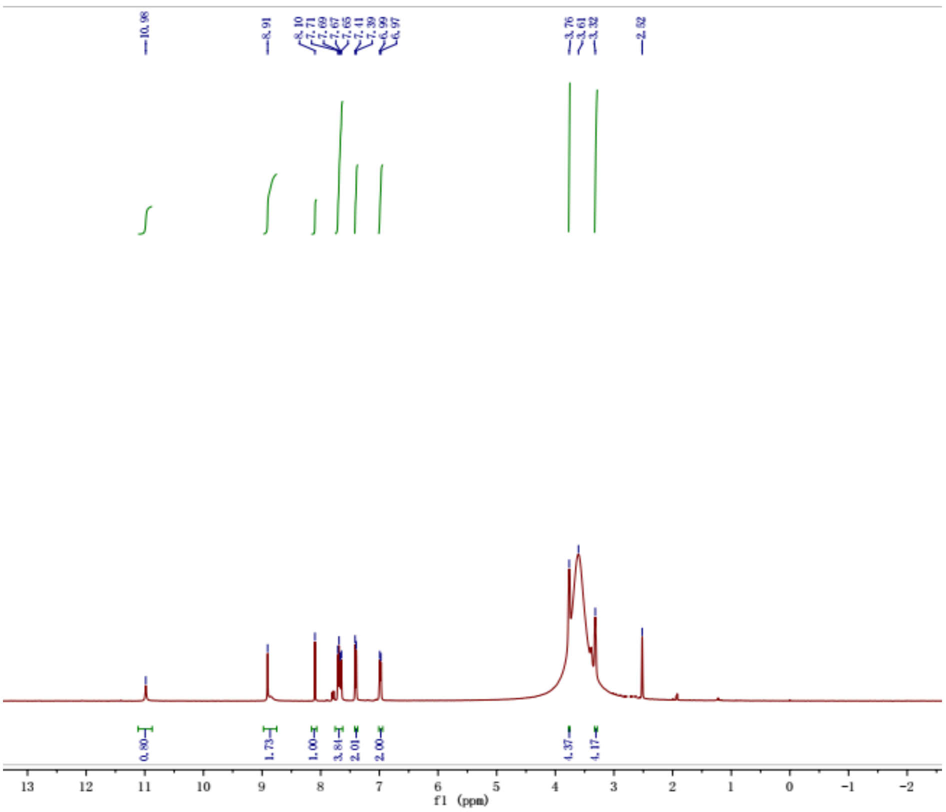


$^{1}$H-NMR spectrum of L12

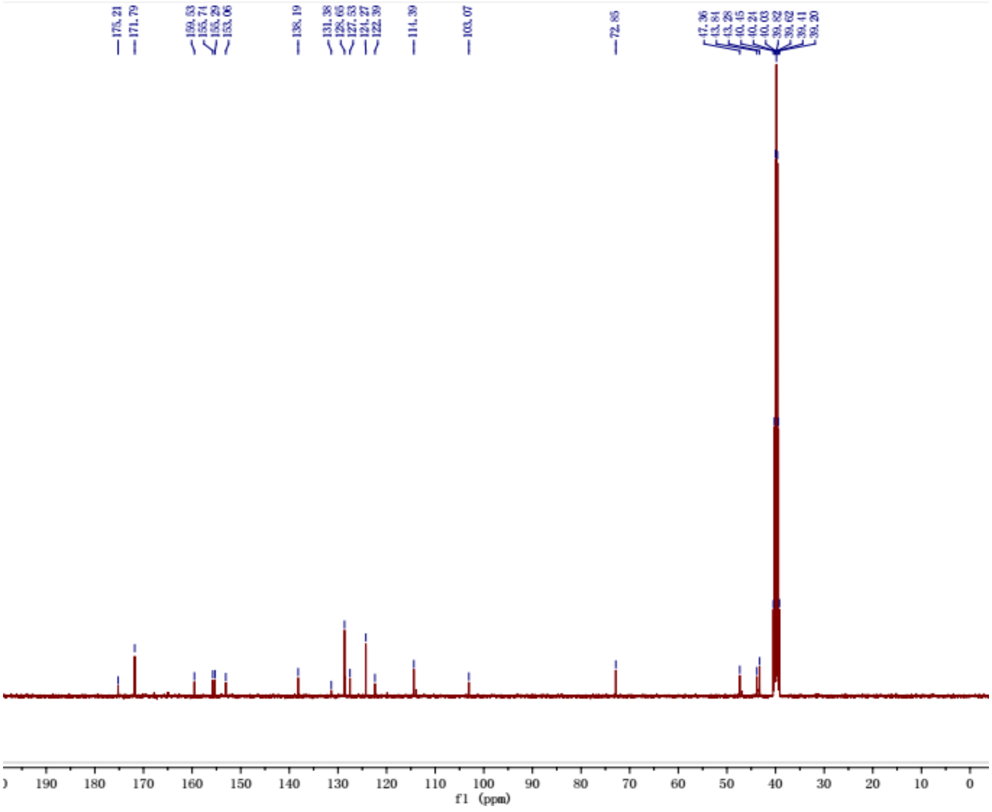


$^{13}$C-NMR spectrum of L12

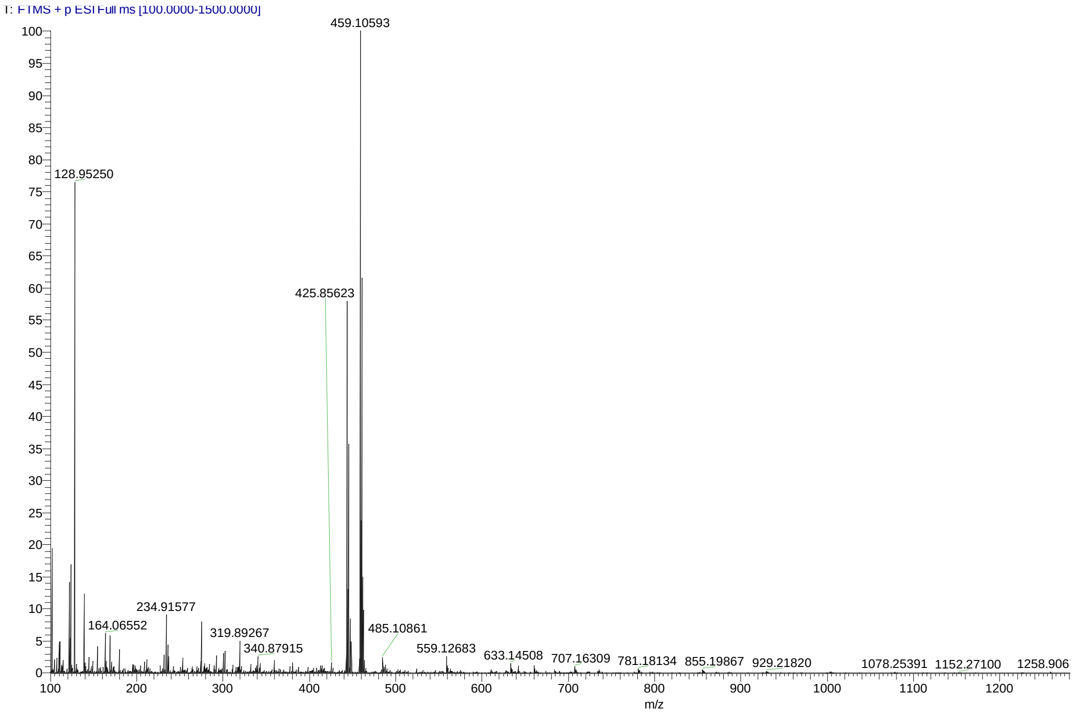


HRMS spectrum of L13

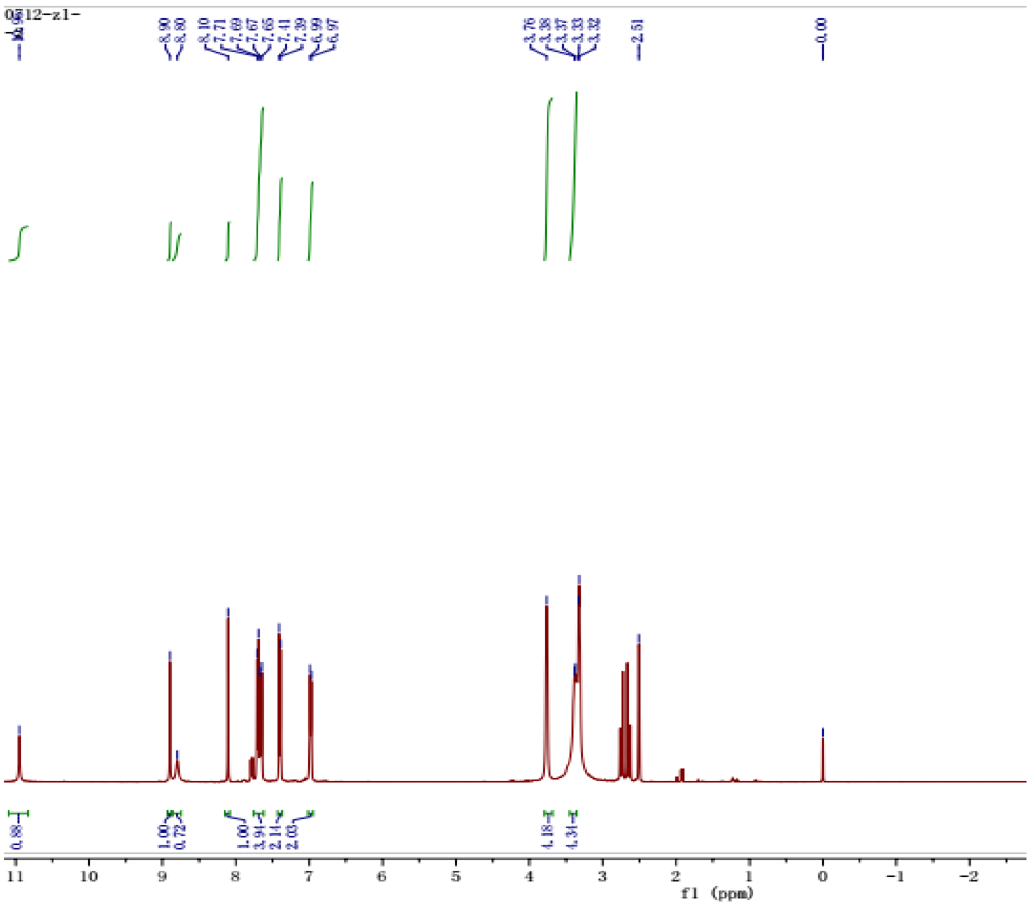


$^{1}$H-NMR spectrum of L13

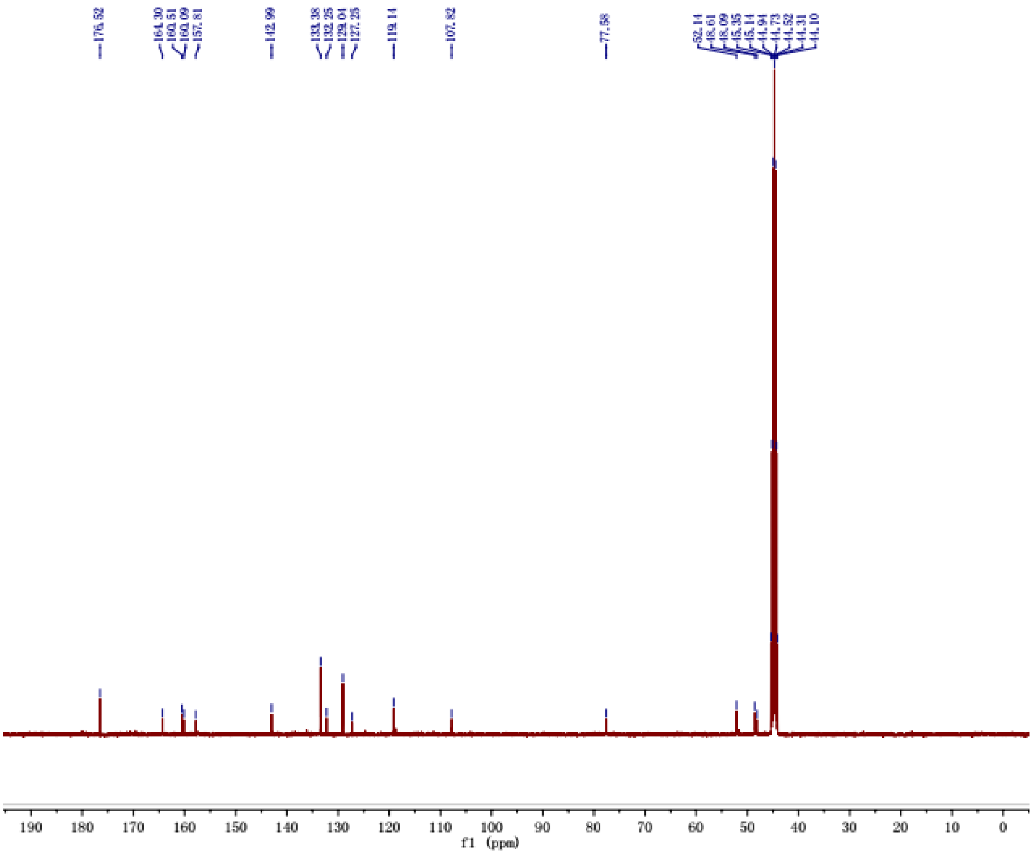


$^{13}$C-NMR spectrum of L13

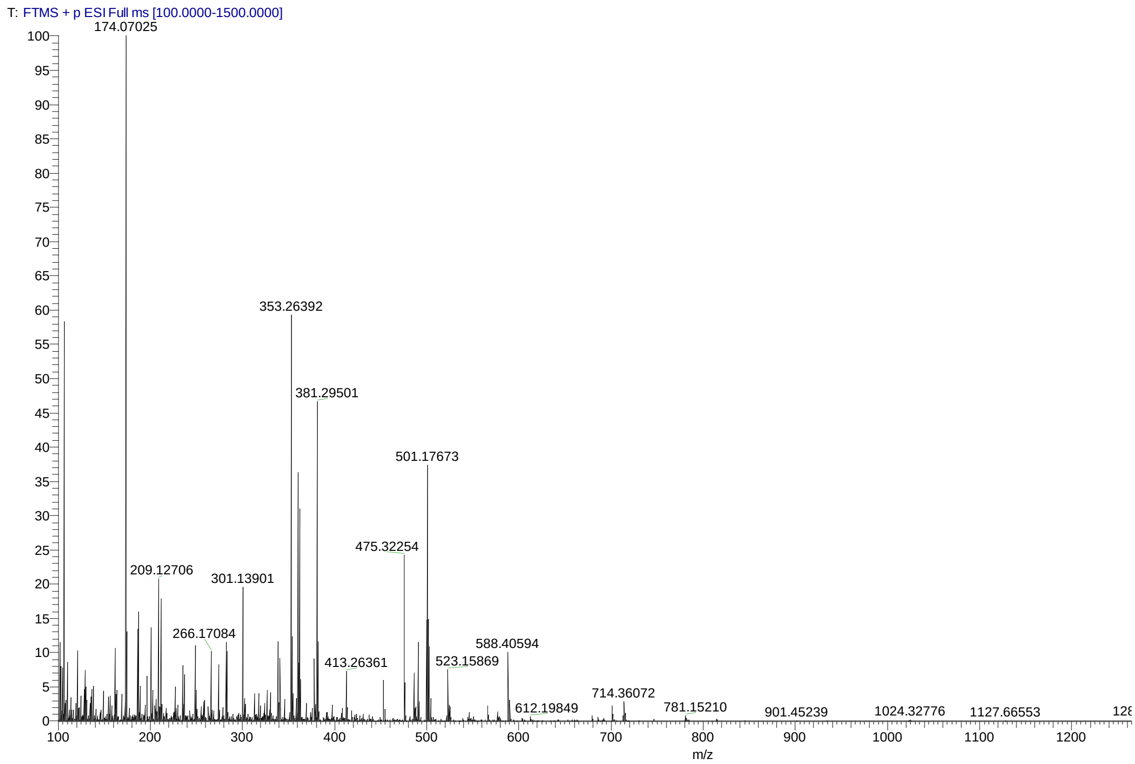


HRMS spectrum of L14

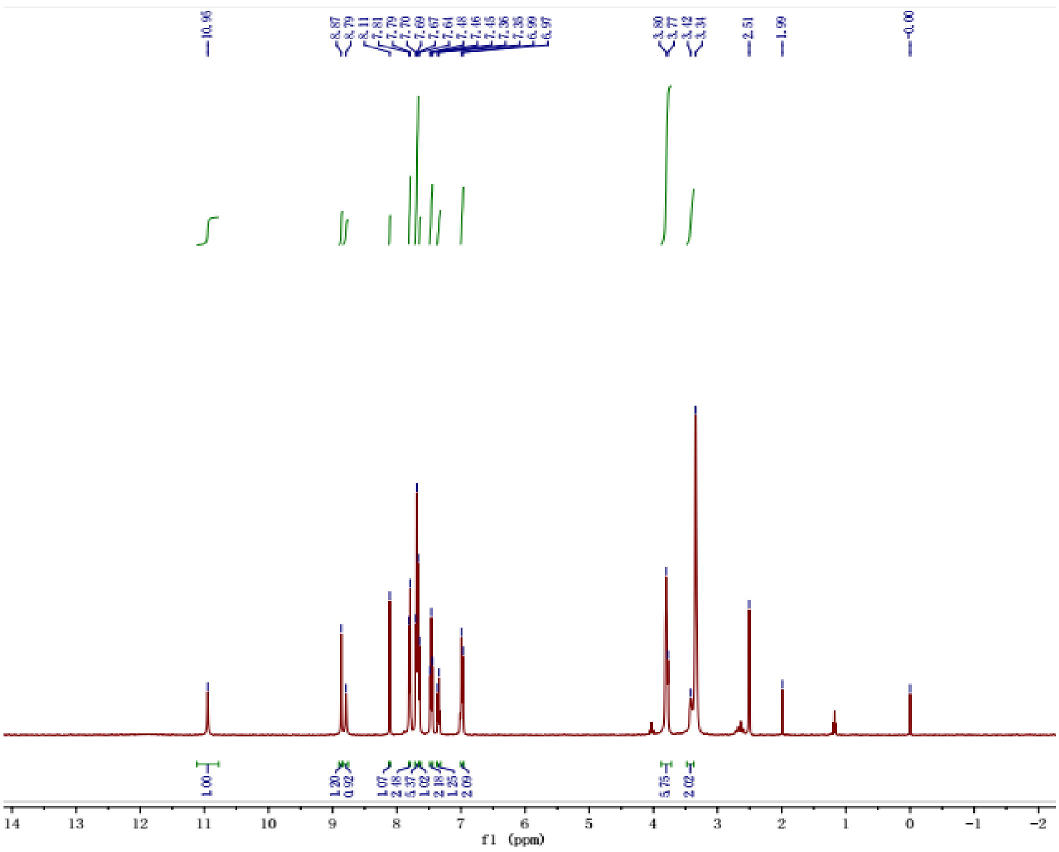

$^{1}$H-NMR spectrum of L14

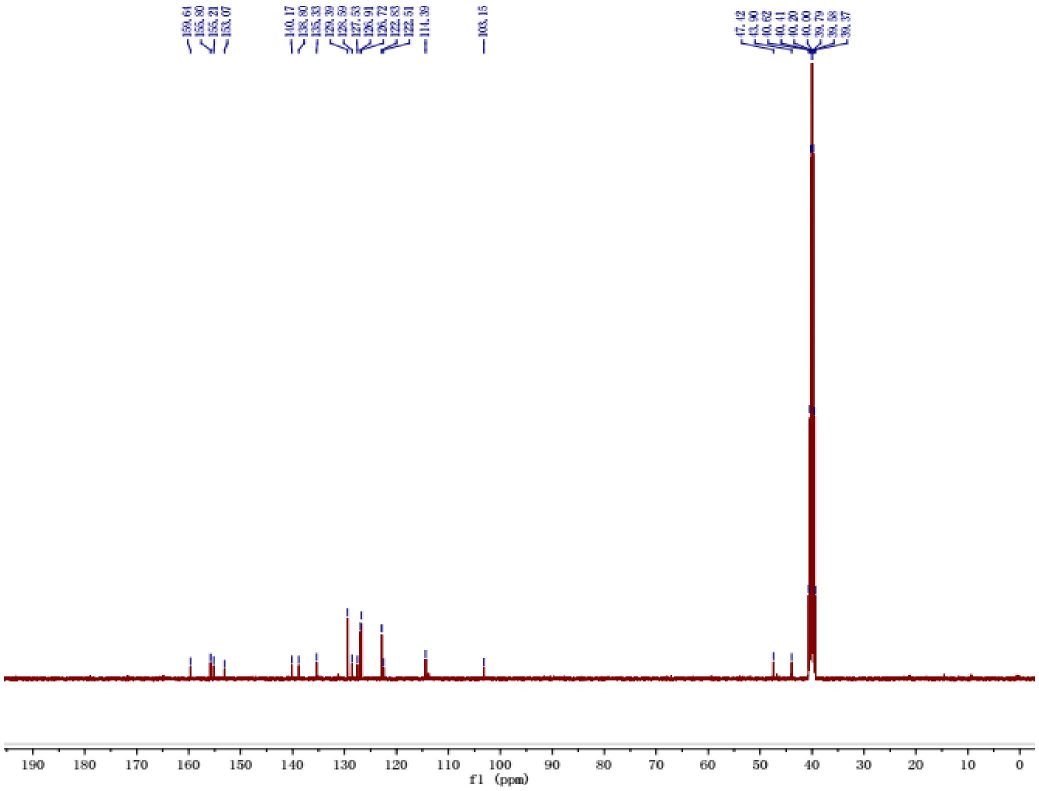

$^{13}$C-NMR spectrum of L14

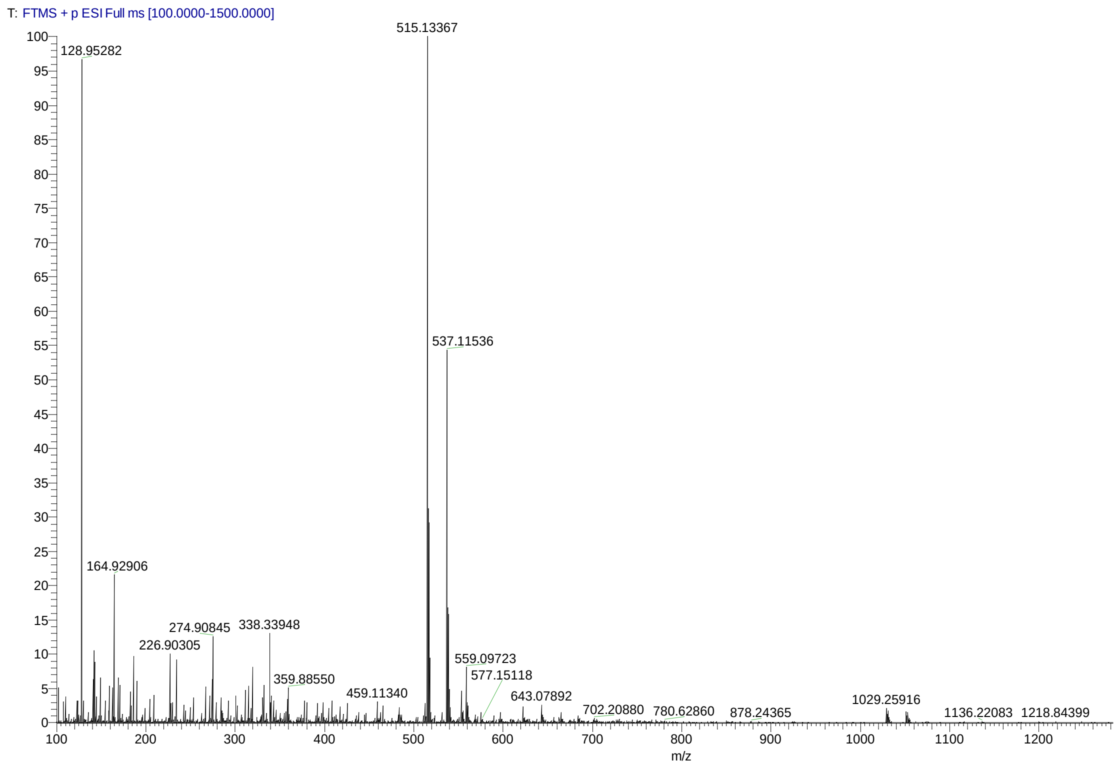


HRMS spectrum of L15

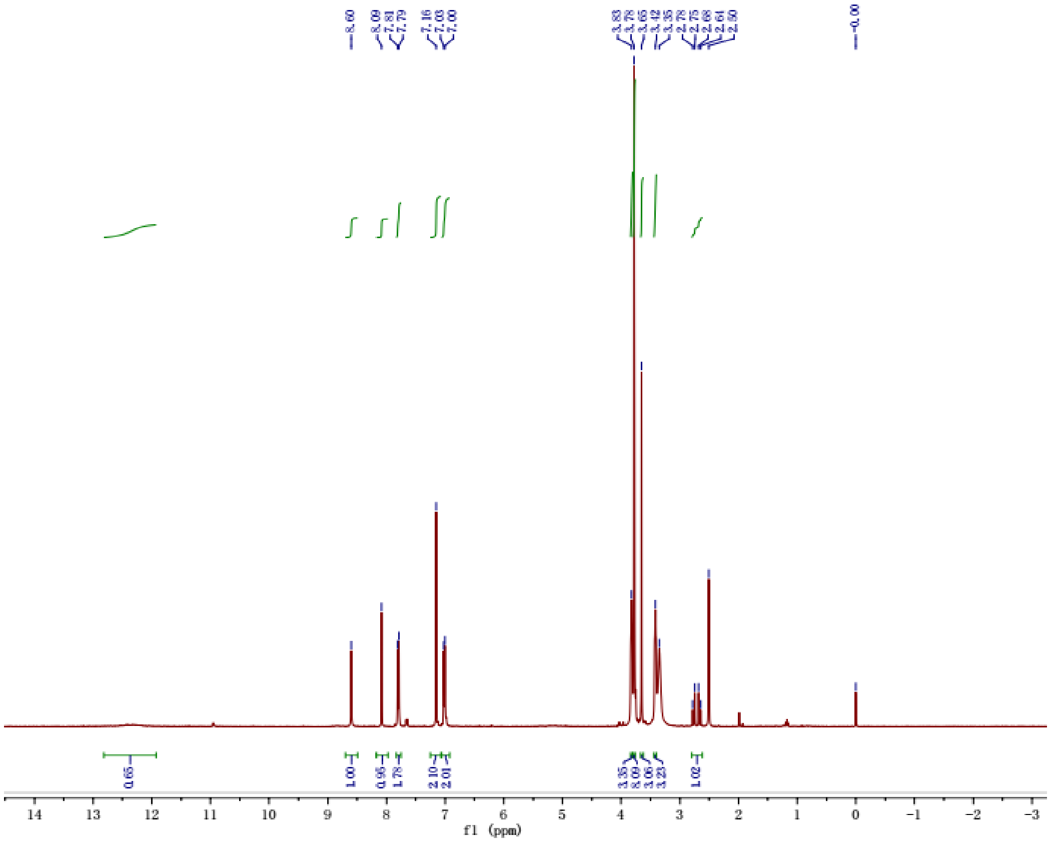


$^{1}$H-NMR spectrum of L15

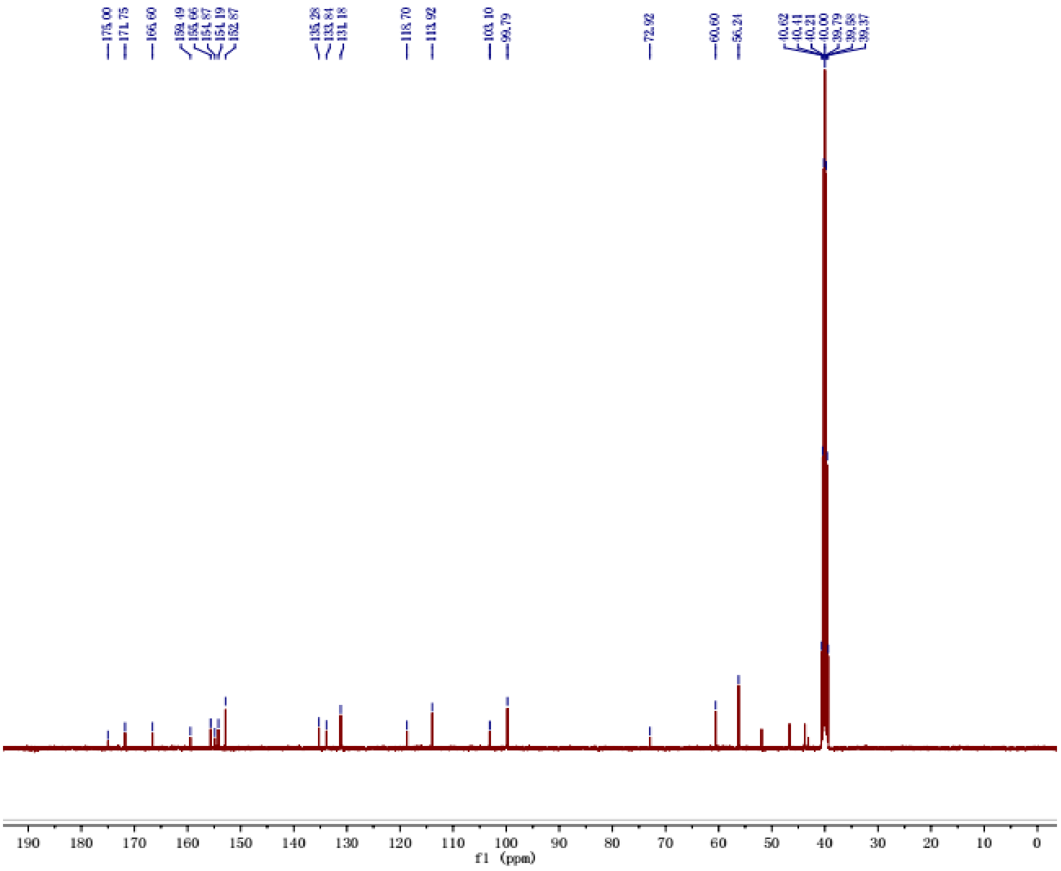


$^{13}$C-NMR spectrum of L15

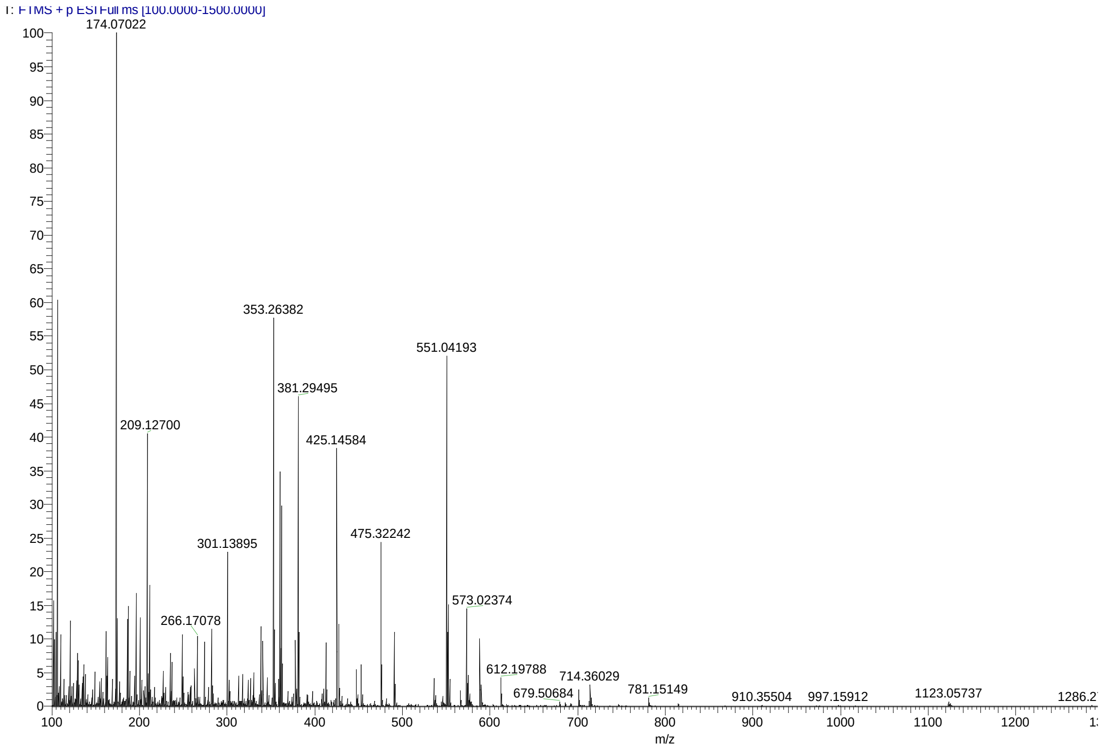


HRMS spectrum of L16

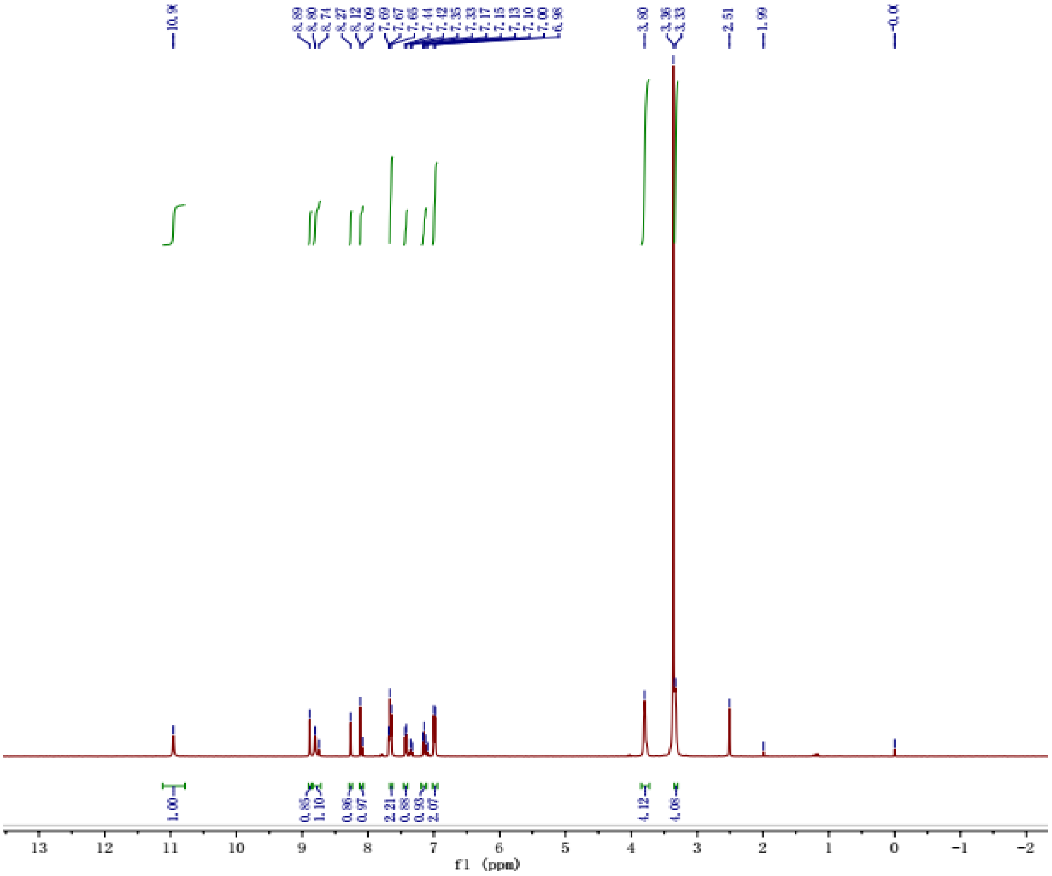

$^{1}$H-NMR spectrum of L16

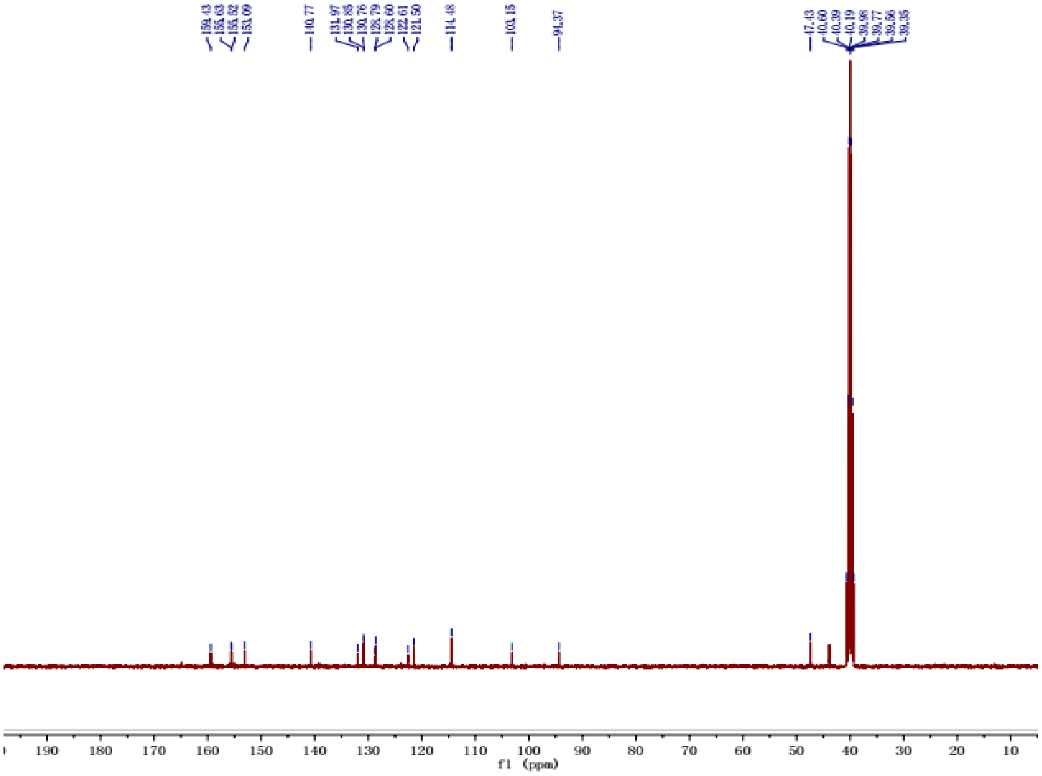

$^{13}$C-NMR spectrum of L16

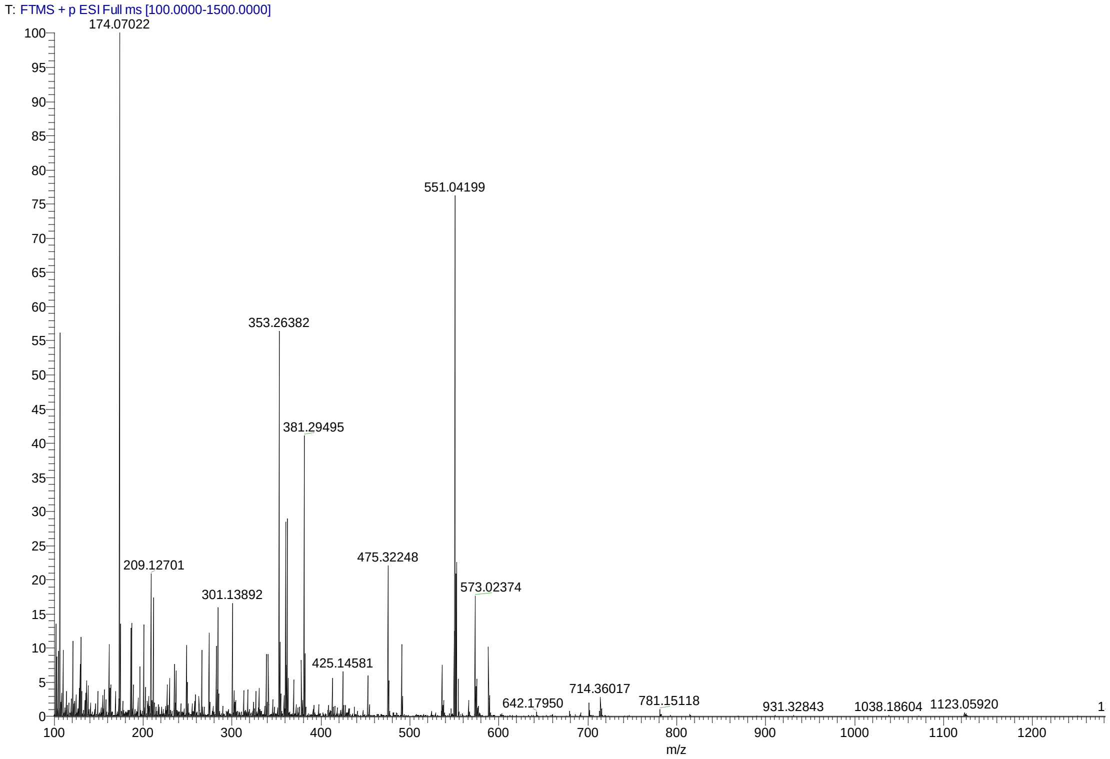


HRMS spectrum of L17

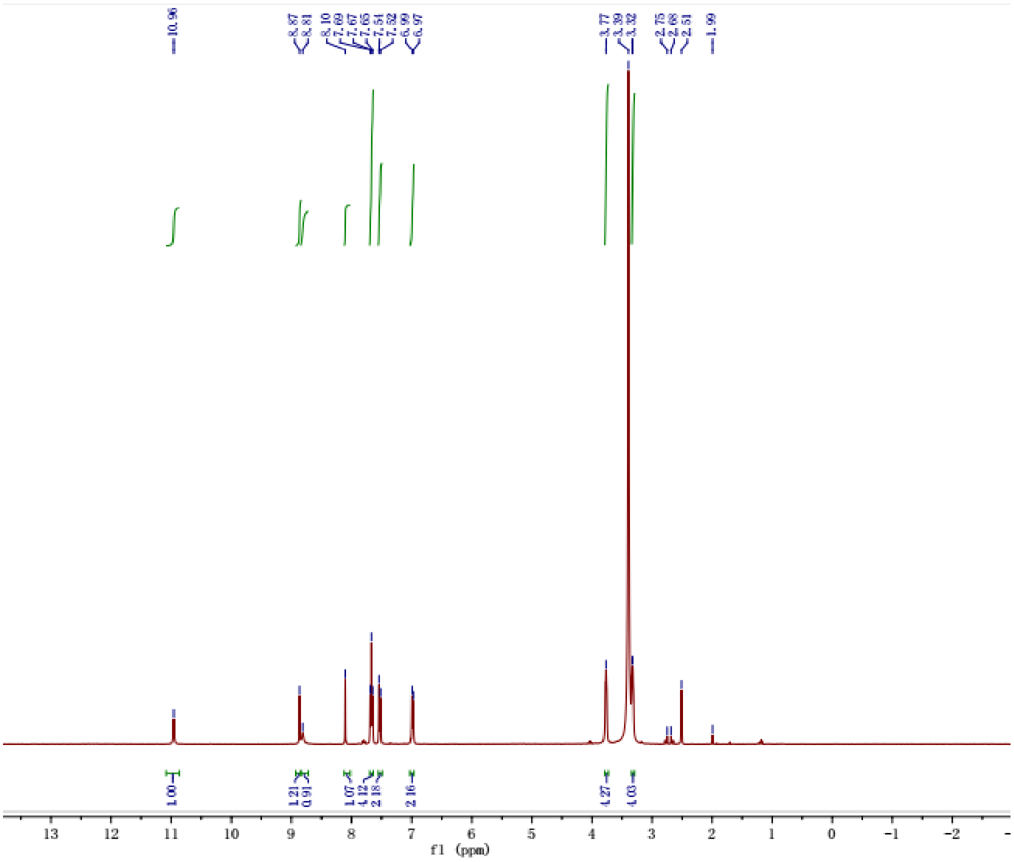


$^{1}$H-NMR spectrum of L17

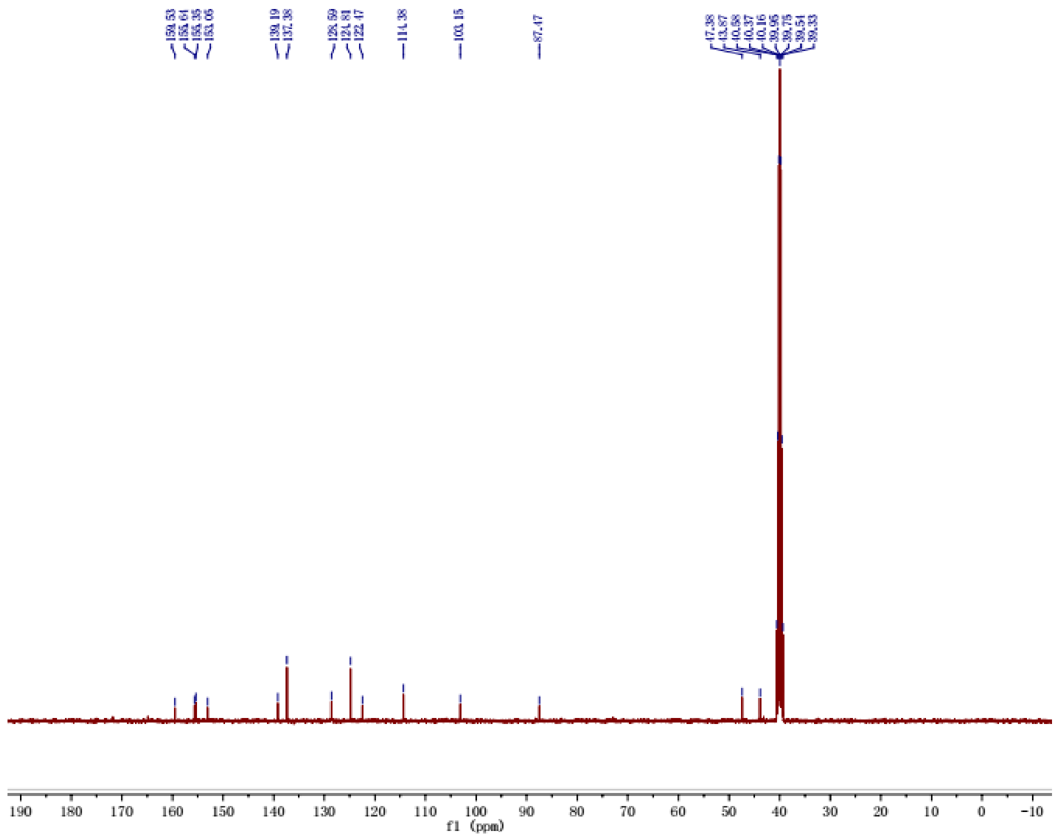


$^{13}$C-NMR spectrum of L17

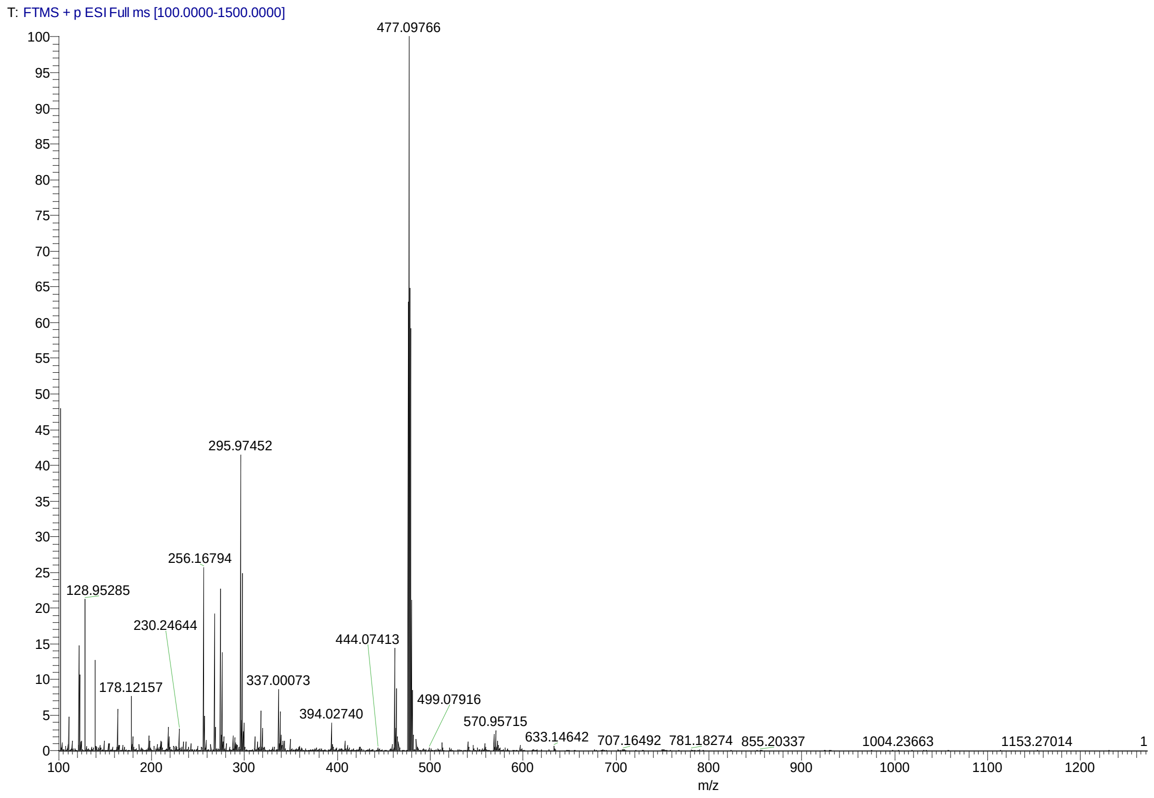


HRMS spectrum of L18

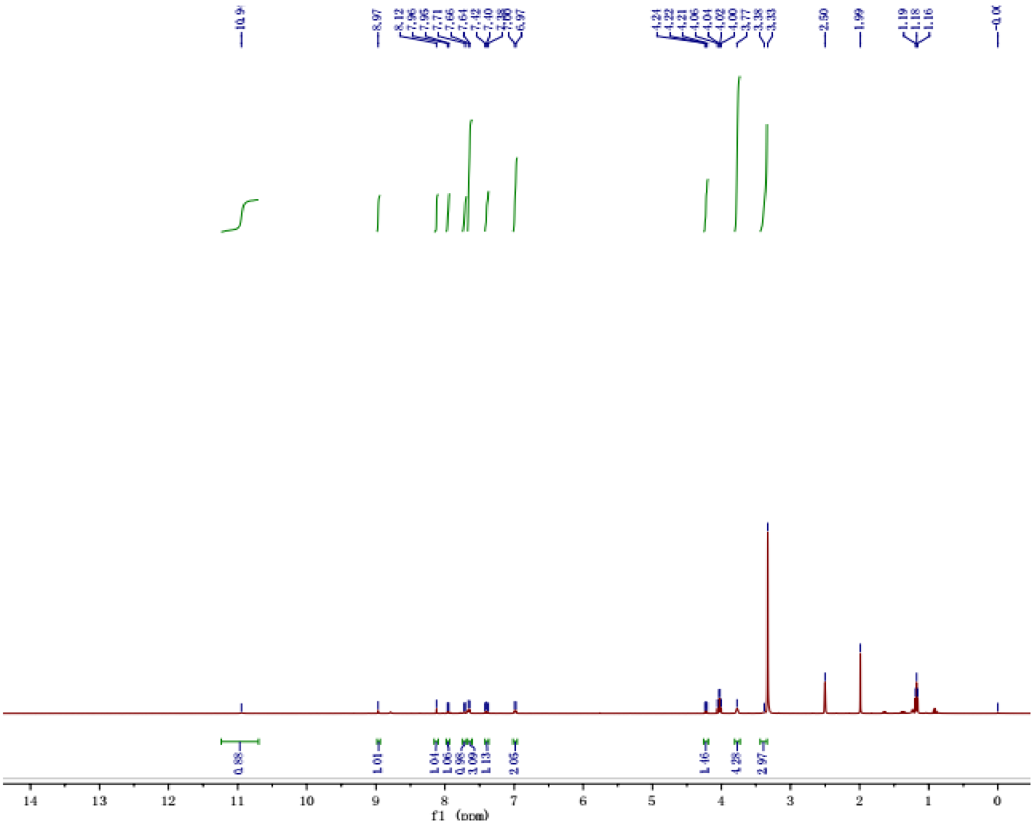

$^{1}$H-NMR spectrum of L18

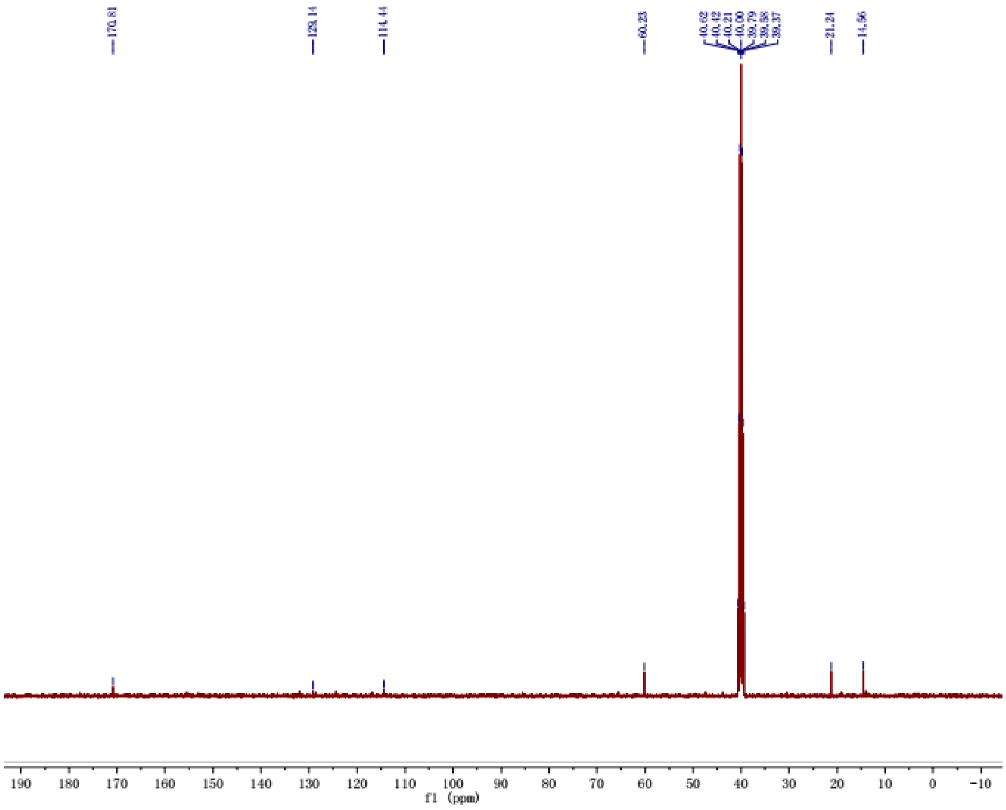

$^{13}$C-NMR spectrum of L18

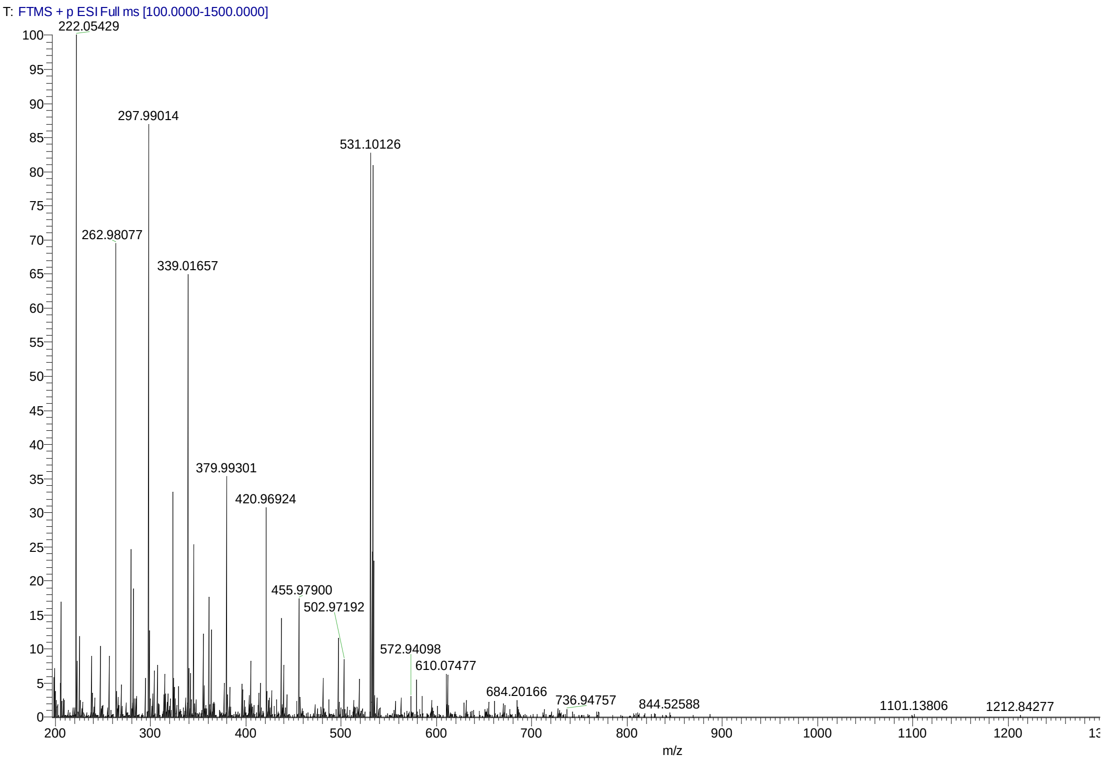


HRMS spectrum of L19

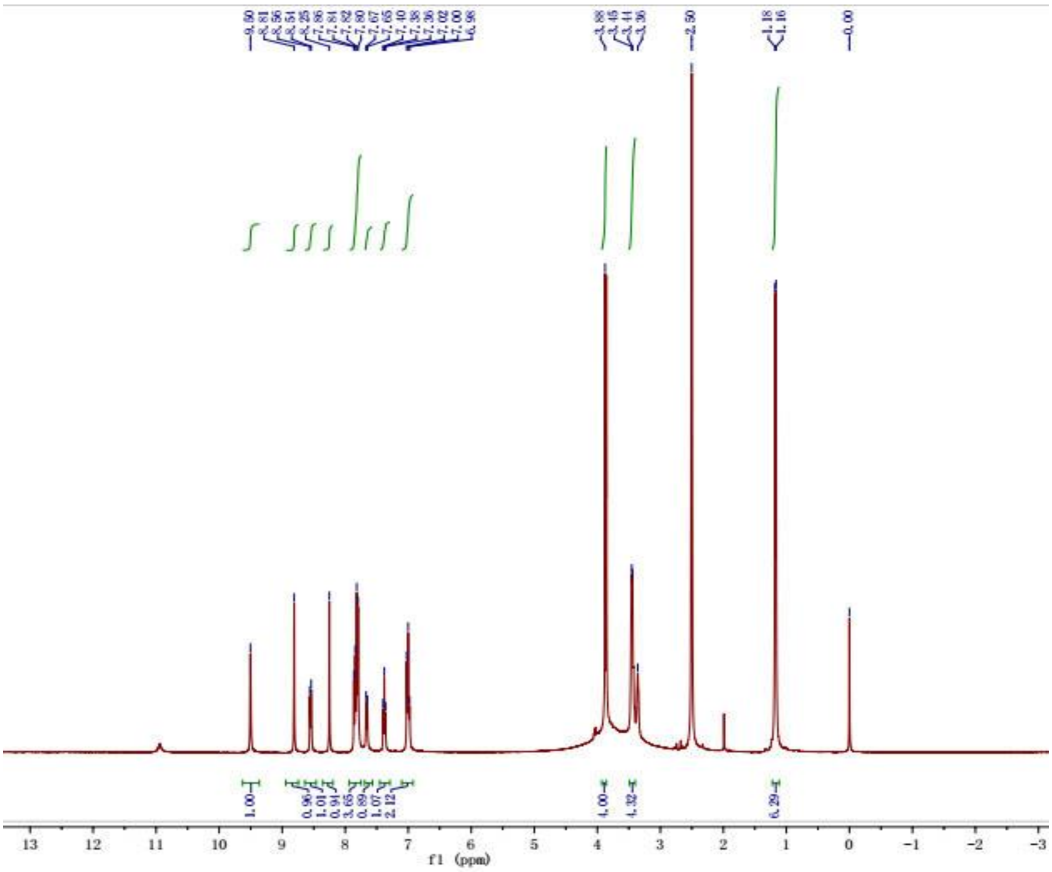


$^1$H-NMR spectrum of L19

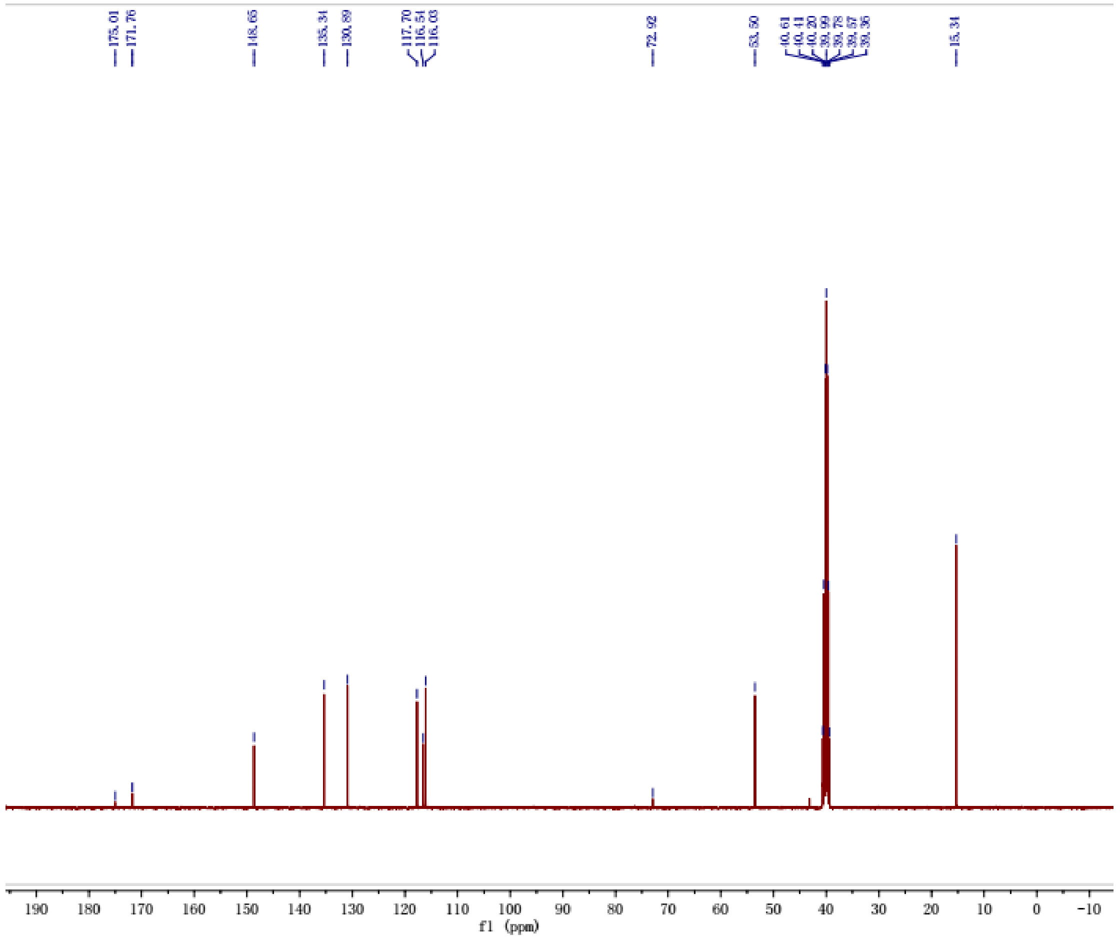


$^{13}$C-NMR spectrum of L19

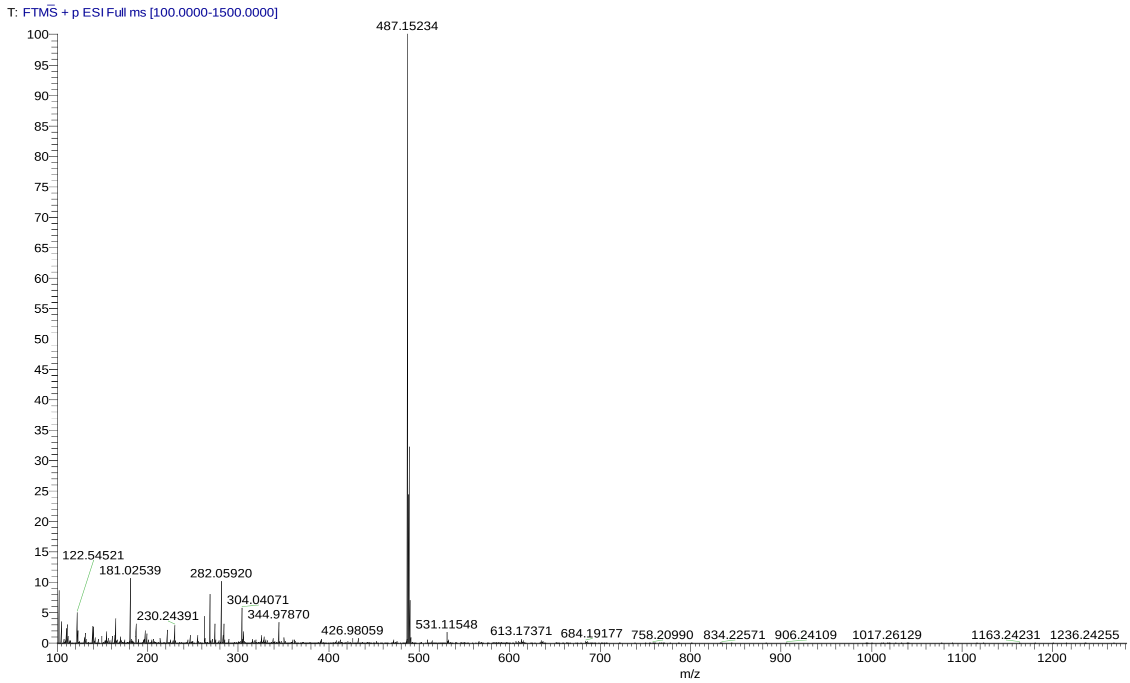


HRMS spectrum of L20

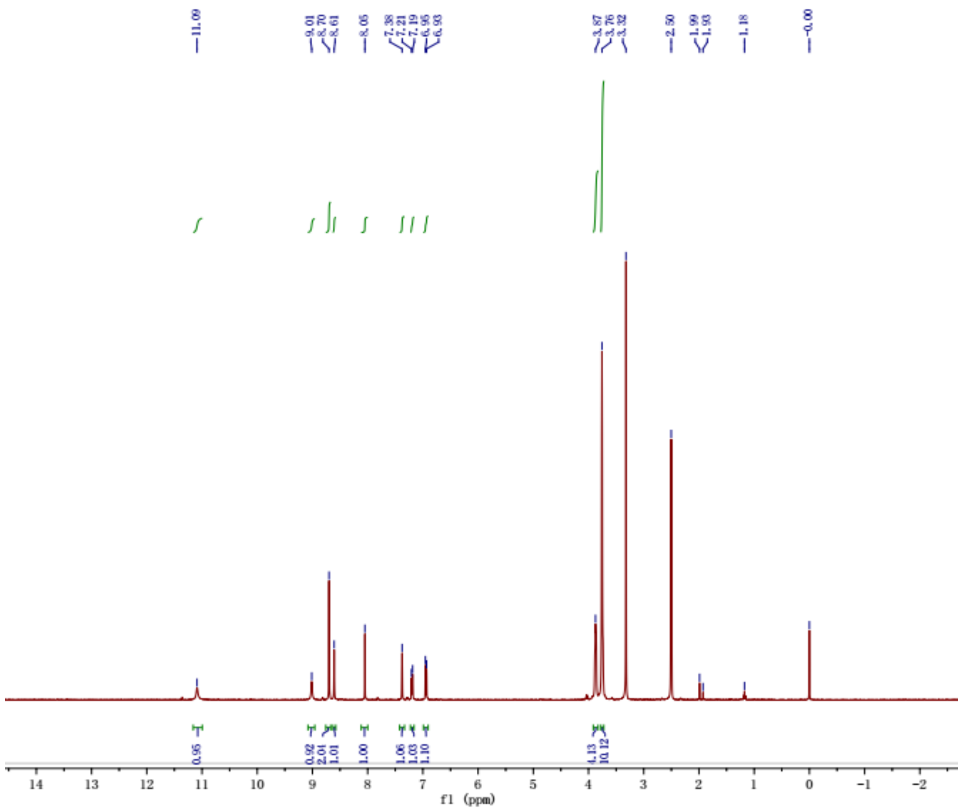


$^{1}$H-NMR spectrum of L20

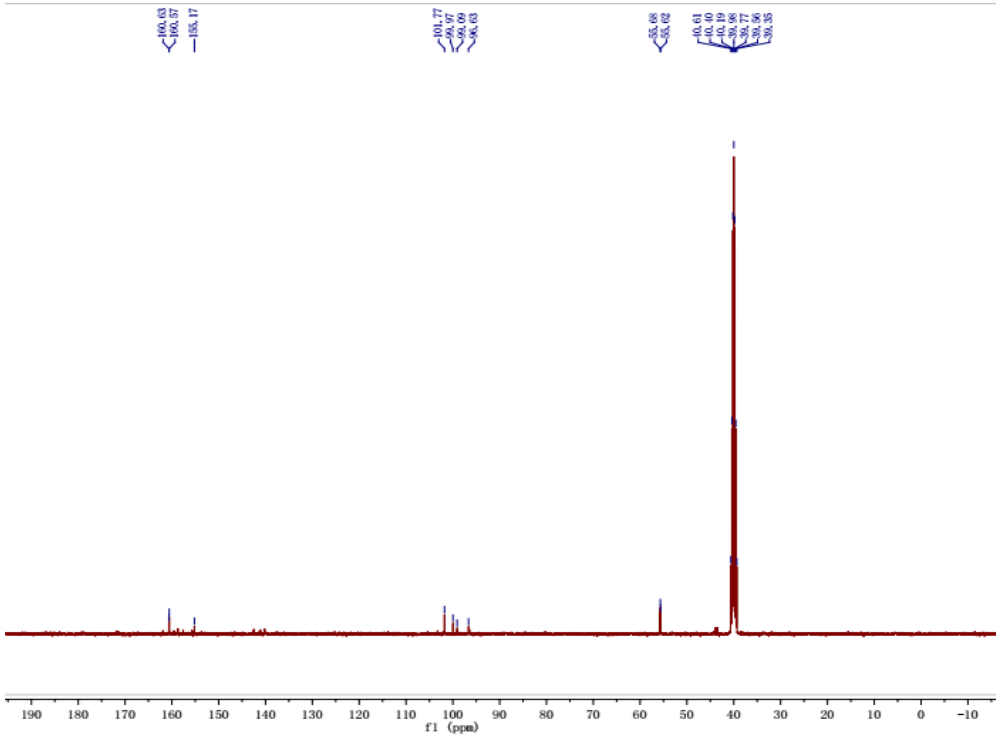


$^{13}$C-NMR spectrum of L20

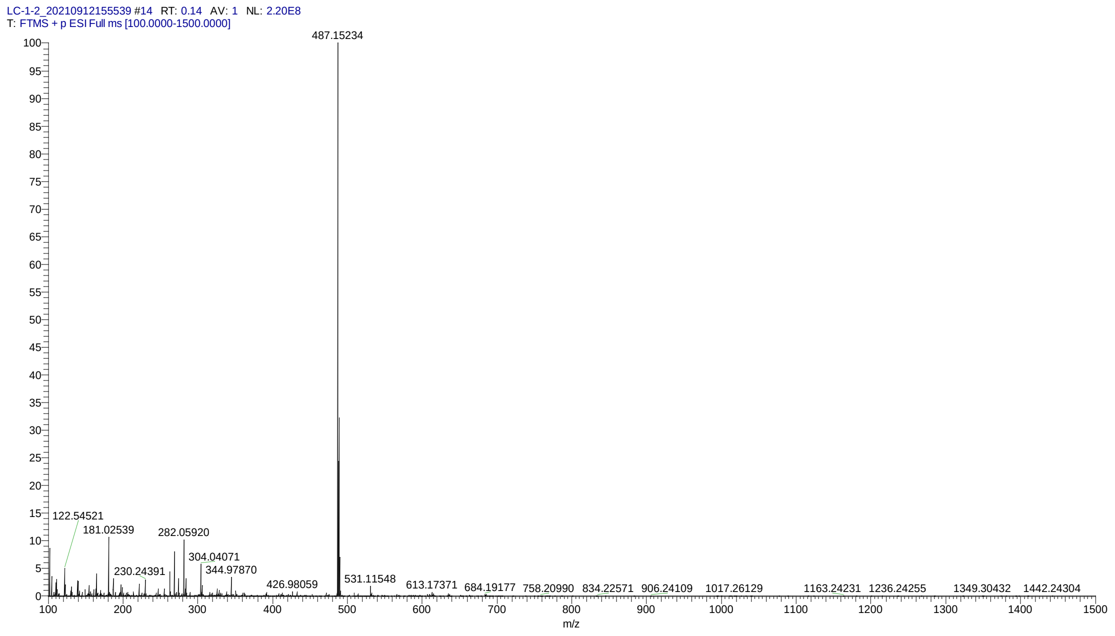


HRMS spectrum of L21

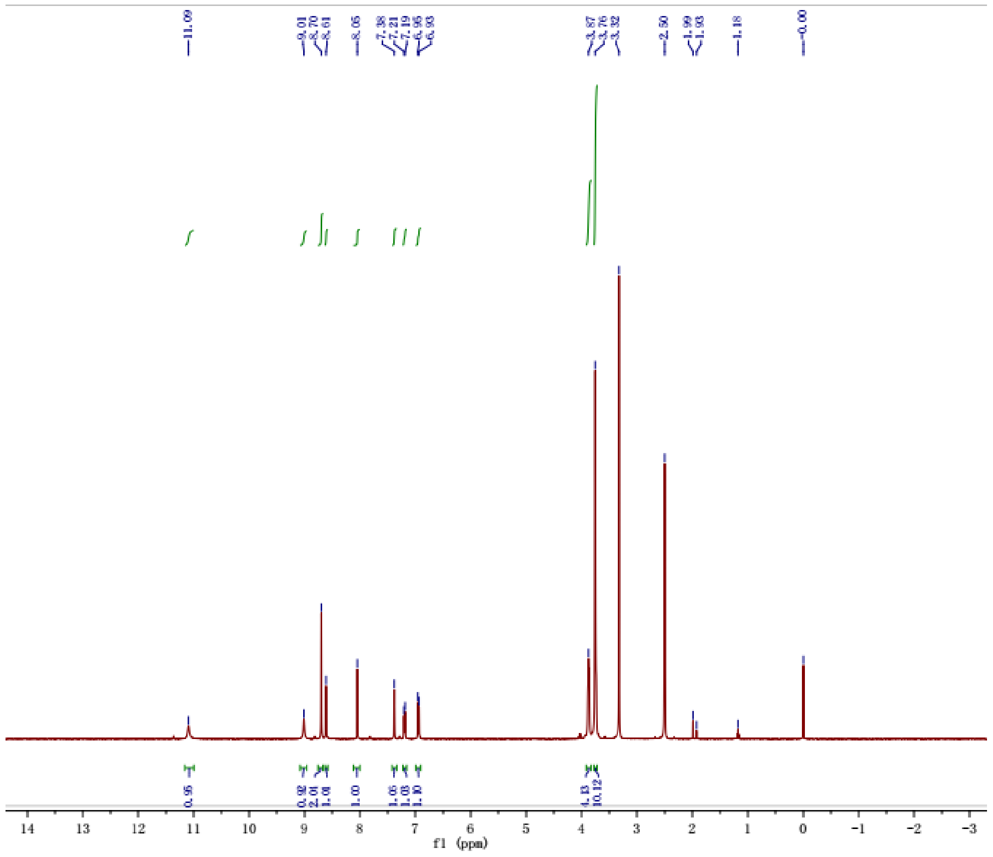


$^{1}$H-NMR spectrum of L21

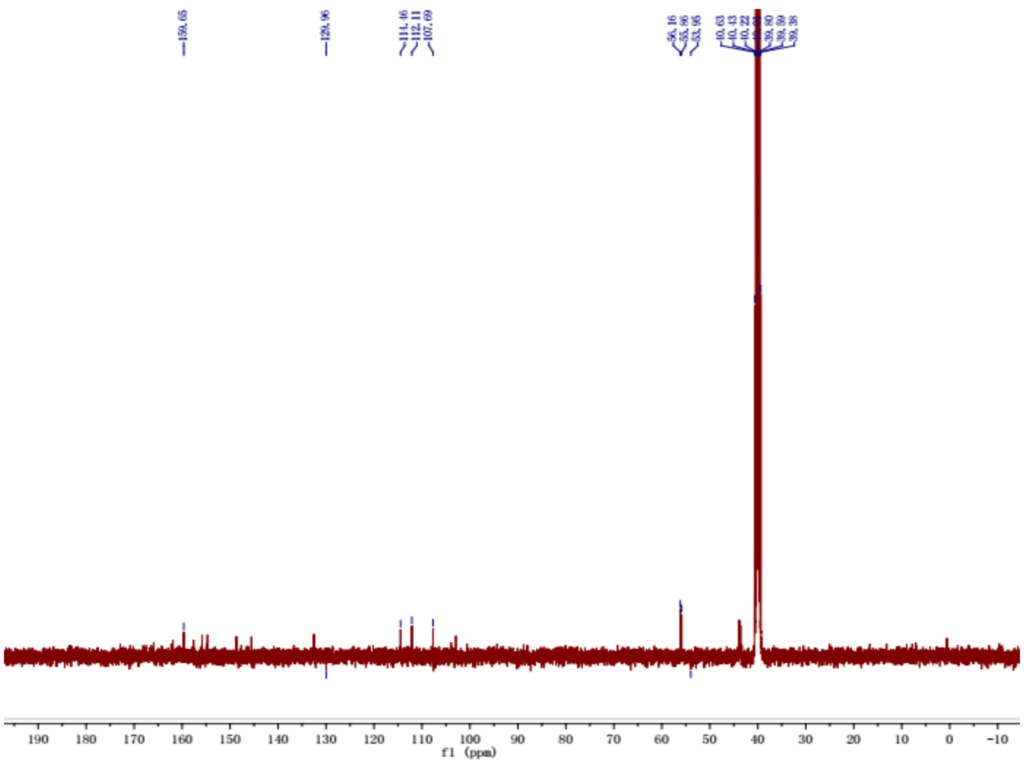


$^{13}$C-NMR spectrum of L21

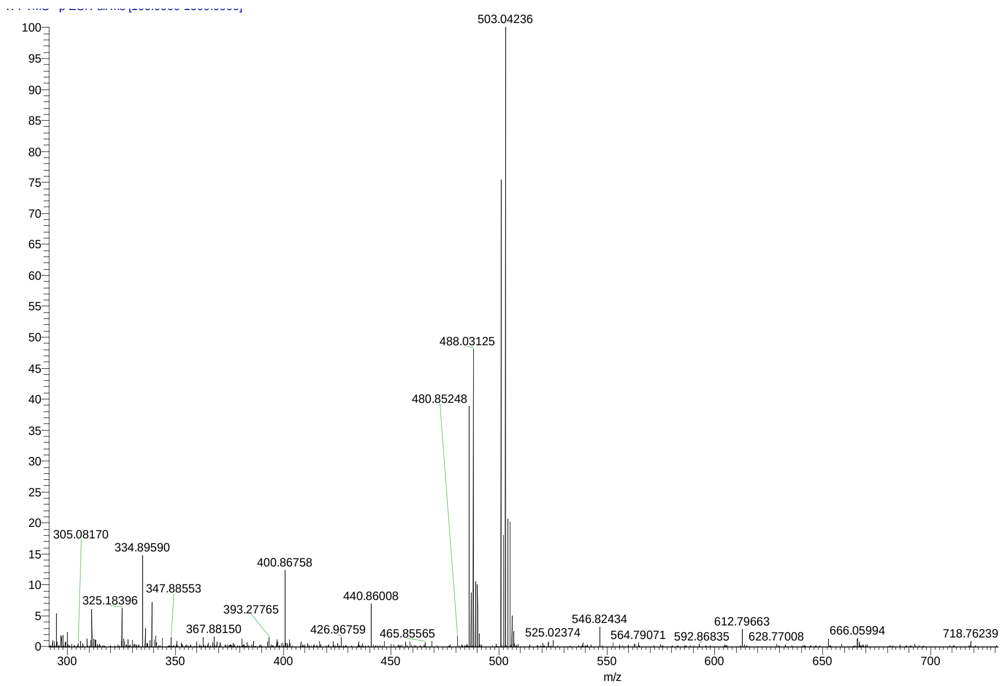


HRMS spectrum of L22

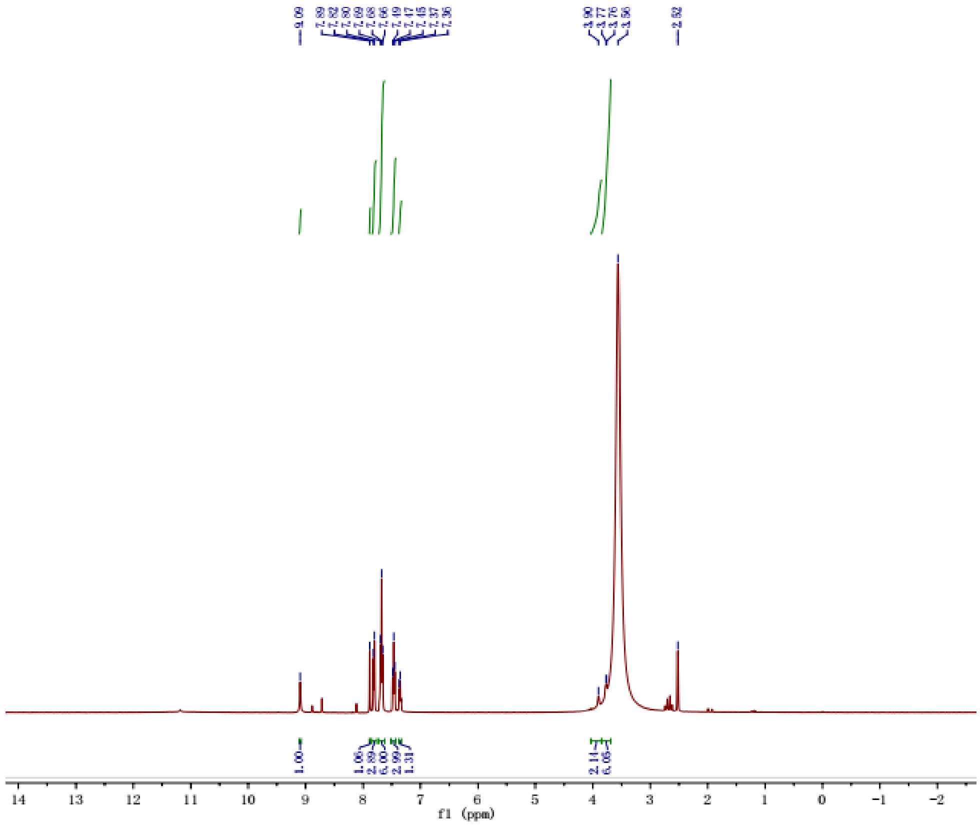

$^{1}$H-NMR spectrum of L22

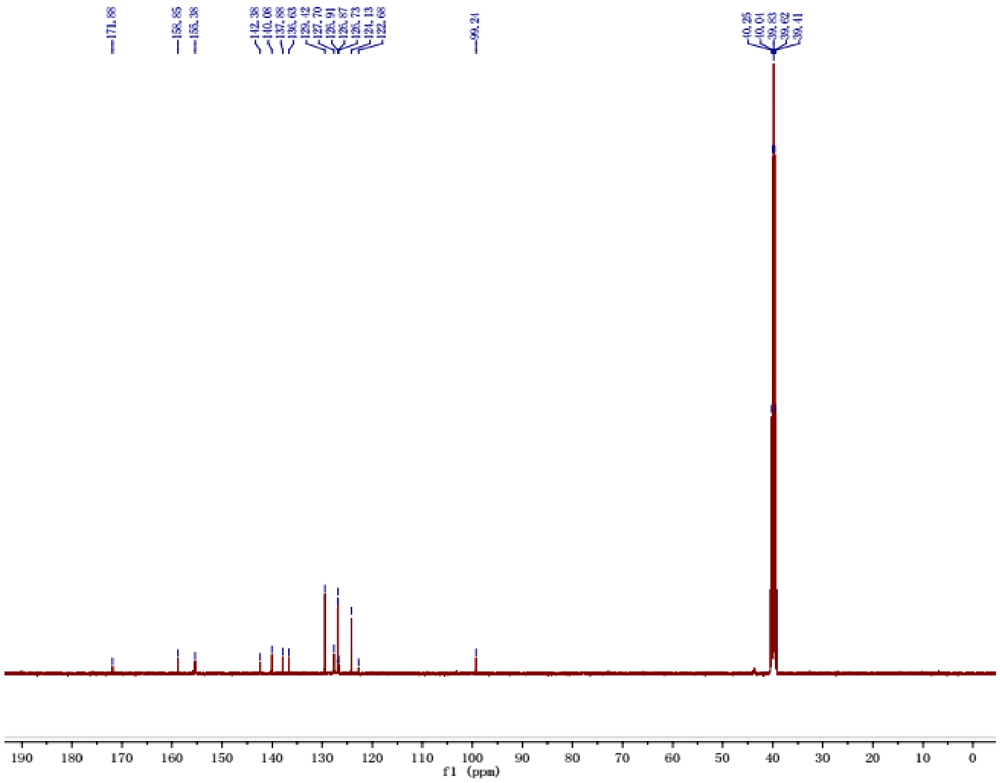

$^{13}$C-NMR spectrum of L22
